# Body fluid volume homeostasis is abnormal in pregnancies complicated with hypertension and/or poor fetal growth

**DOI:** 10.1371/journal.pone.0206257

**Published:** 2018-11-01

**Authors:** Wilfried Gyselaers, Sharona Vonck, Anneleen Simone Staelens, Dorien Lanssens, Kathleen Tomsin, Jolien Oben, Pauline Dreesen, Liesbeth Bruckers

**Affiliations:** 1 Faculty of Medicine and Life Sciences, Hasselt University, Diepenbeek, Belgium; 2 Department of Obstetrics & Gynaecology, Ziekenhuis Oost-Limburg, Genk, Belgium; 3 Department of Physiology, Hasselt University, Diepenbeek, Belgium; 4 Interuniversity Institute for Biostatistics and statistical Bioinformatics, Hasselt University, Diepenbeek, Belgium; University Medical Center Utrecht, NETHERLANDS

## Abstract

**Objectives:**

To evaluate body water volumes and cardiac output in each trimester of pregnancies complicated with hypertension and/or poor fetal growth, relative to uncomplicated pregnancy.

**Methods:**

In this semi-longitudinal cohort study, a standardised non-invasive maternal hemodynamics assessment in first, second or third trimester was performed in 1068 women with uncomplicated pregnancy (UP), 75 with early onset (EPE) and 117 with late onset preeclampsia (LPE), 139 with gestational hypertension (GH), 129 with small for gestational age (SGA) neonates and 43 with essential hypertension (EH). Women with hypertension or SGA were included prior to onset of symptoms or at diagnosis of disease; 46% of women (758/1631) were assessed in ≥ 2 trimesters. Impedance cardiography and spectrum analysis were used to measure cardiac output, total body water (TBW), extracellular (ECW) and intracellular water (ICW). A linear mixed model was used for inter-trimestrial comparison of parity-, age- and BMI-corrected values within and between groups.

**Results:**

For all pregnancies, TBW is higher in each consecutive trimester, mainly due to increasing fraction of ECW (ECW%). Compared to first trimester UP, ECW and ECW% are higher in EPE whereas TBW, ECW and ICW are lower in SGA. Compared to inter-trimestrial differences in UP, abnormal changes for body water volumes are observed in GH, EPE and LPE and for CO in EPE and LPE. Changes in EH are not different from UP.

**Conclusions:**

This study is the first to show that concomitant gestational changes of ECW and CO are different from UP already in preclinical stages of pregnancies complicated with hypertension and/or poor fetal growth, except EH. This finding highlights the relevance of early gestational assessment of maternal body fluid status in pregnancies at risk for hypertension or poor fetal growth.

## Introduction

A woman’s total body water volume is estimated at around 55% of her body weight. Two third of this volume is intracellular fluid and the remaining third is located extracellularly in the intravascular, interstitial and transcellular compartments [1]. All fluid compartments function interdependently, reaching steady states via a continuous exchange of water volume and constituents under physiologic and pathophysiologic conditions [2]. There are several techniques available to measure volumes of the different compartments in vivo [3], however not all are safely applicable during pregnancy. Non-invasive bio-impedance technologies, such as bio-impedance spectroscopy (BIS) or bio-impedance analysis (BIA) are based on the conductivity by human body tissues for imperceptible electrical currents, that varies according to the amount of accumulated fluids [4]. BIA can safely be used in pregnant women, but its reliability has been questioned for technical, mathematical or algorithmic limitations [5] or inconsistencies of BIA body composition measurements with hydrostatic weighing [6] or other gold standard methods [5, 7]. When using bioelectrical impedance technology, it is recommended to consider the measured values not interchangeable with those obtained by other technologies [8], and to use population- and device-specific reference ranges [9]. Despite these limitations, several strengths of the BIA technology have also been reported, such as (a) its simplicity and low cost, (b) its poor interference by dietary or activity variations [10], (c) its high repeatability and reproducibility when applied under standardised conditions in a variety of selected populations of healthy and chronically ill children/adolescents [11–13], healthy adults [14, 15], critically ill [16] and oncologic patients [17], in obese individuals [18] and also in pregnant subjects [19, 20]. In the latter group, maternal measurements are associated with neonatal birth weight [21, 22] and maternal outcome [20, 23–25].

A rise of maternal cardiac output and plasma volume, in association with a generalised vasodilation and reduced total peripheral resistance, are well known features of normal gestational physiology. It is also known that pregnancies, complicated with hypertension or poor fetal growth, present with inadequate maternal cardiovascular adaptations.

Associations between maternal cardiac function and volume homeostasis have been reported in both normotensive and hypertensive pregnant women [26], and abnormal values of cardiac output have been observed in both latent [27] and clinical phase of preeclampsia [28]. However today, no information exists on the concomitant changes of cardiac output and body water volumes in each trimester of pregnancies complicated with early-onset (EPE) or late onset preeclampsia (LPE), gestational hypertension (GH), essential hypertension (EH) or in normotensive women carrying neonates small for gestational age (SGA). We hypothesized that these complicated pregnancies present with phenotype-specific differences of total body water, extracellular fluid and cardiac output, which either (a) are present already from the first trimester onward, or (b) develop during the course of pregnancy. Identification of these phenotype-specific differences might be helpful in the prediction and/or identification of gestational hypertensive diseases at early stages of pregnancy.

## Materials and methods

### Population

**Fig 1:**
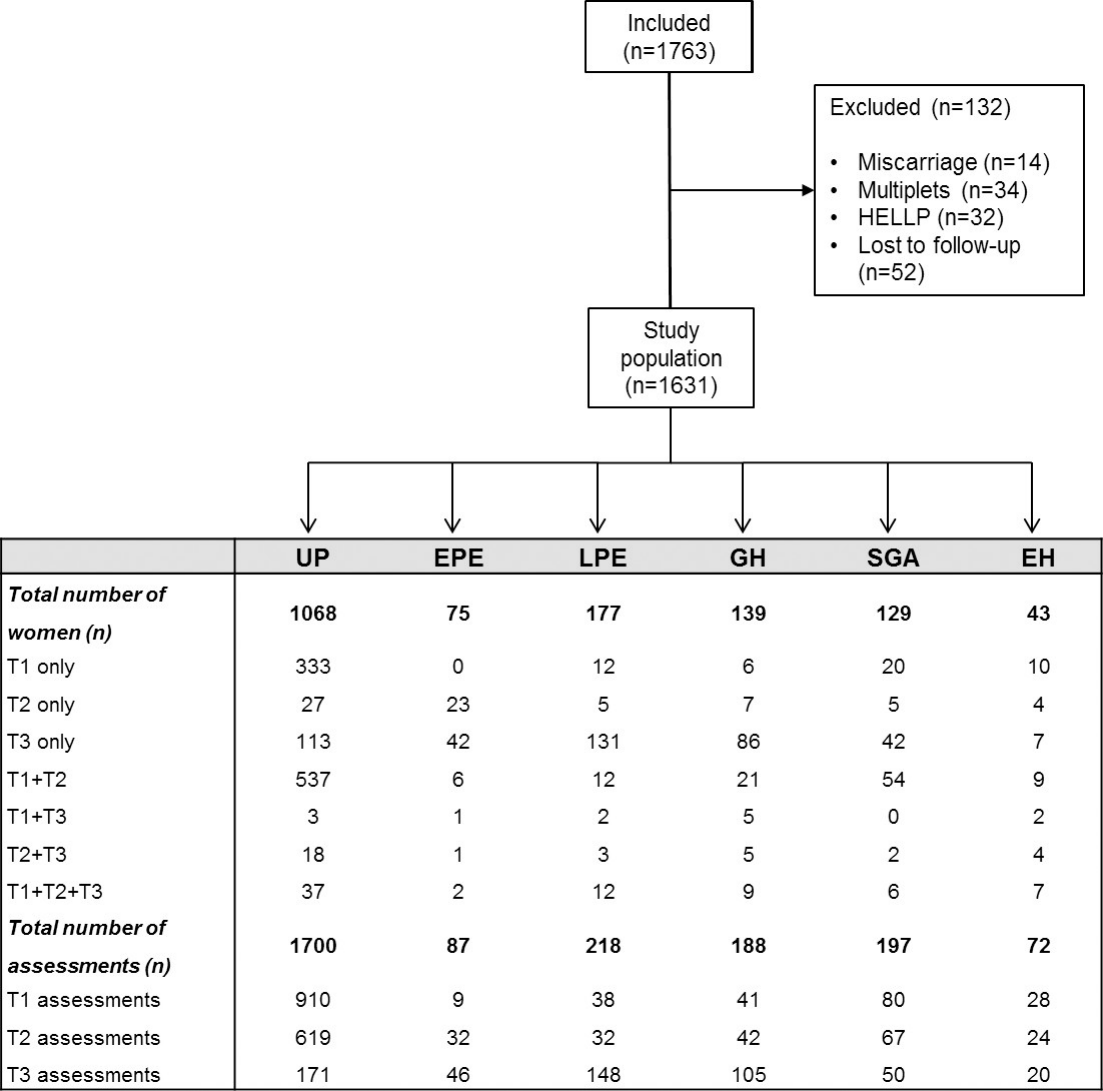
Flowchart from pregnancies included in the observational study as part of the Hasselt University Study Project on Maternal Venous Hemodynamics. 1620 pregnant women were classified based upon diagnosis in gestational hypertension (GH), late preeclampsia (LPE), early preeclampsia (EPE), essential hypertension (EH), uncomplicated pregnancy (UP) with or without small for gestational age (SGA) neonates. Assessments per patient were done in the first, second or third trimester (1T, 2T, 3T resp.) alone or in multiple trimesters.

Ethical approval by the Comité Medische Ethiek Ziekenhuis Oost Limburg, Genk was obtained before study onset (reference: 06/043, 08/049, 13/090U) and informed consent was obtained before inclusion. Women with apparently normotensive singleton pregnancies at random gestational ages, presenting at the obstetric ultrasound scanning clinic of Ziekenhuis Oost-Limburg Genk, Belgium, as well as women referred to the Maternal-Feal-Medicine Department of the same hospital with suspected new-onset hypertension in ambulatory or hospital setting, were invited to participate in an observational study on maternal cardiovascular functioning between 2011–2017, as part of the ongoing Hasselt University Research Project of Maternal Venous Hemodynamics. When included in first or second trimester, women were asked for repeat measurements in consecutive trimesters. Three periods of assessment were considered in the statistical analysis: women included in the first trimester (< 15 weeks), second trimester (15+0 to 27+6 weeks) and third trimester (≥ 28 weeks). Per trimester, only 1 measurement per pregnancy was included. At birth, data on gestational outcome was categorized according to the criteria revised by the International Society for Studies of Hypertension in Pregnancy (ISSHP) [29]. The categories contained women with (1) uncomplicated pregnancies and neonates appropriate or large for gestational age (UP), (2) early-onset preeclampsia (EPE), defined as new-onset hypertension or superimposed on chronic hypertension, associated with proteinuria ≥300mg/24h, other organ dysfunction or foetal growth restriction, and symptomatic at gestation < 34 weeks, (3) late-onset preeclampsia (LPE), defined as new-onset hypertension or superimposed on chronic hypertension, associated with proteinuria ≥300mg/24h, other organ dysfunction or foetal growth restriction, and symptomatic at gestation ≥ 34 weeks, (4) gestational hypertension (GH), defined as new-onset hypertension > 20 weeks, without proteinuria or organ dysfunction, with or without fetal growth restriction, (5) neonates small for gestational age (SGA), born after normotensive pregnancies, and (6) essential hypertension (EH) with high blood pressure before 20 weeks of gestation but without evolution to superimposed preeclampsia. Multiplet pregnancies or women with pre-existing cardiac, renal, endocrine, hematologic or auto-immune diseases were excluded, as well as women with miscarriage, HELLP syndrome or incomplete follow up (Fig 1). Demographic details were maternal age (years), pregestational BMI as per medical history, gestational age at assessment and at delivery, parity, smoking, medication, neonatal birth weight and birth weight percentile.

Bioimpedance cardiography and spectrum analaysis were applied according to a standardized protocol with known inter- and intra-observer correlations as reported in previous publications [30, 31], in combination with population- and technology specific reference ranges [20, 31].

### Impedance Cardiography (ICG)

The Non-Invasive Continuous Cardiac Output Monitor (NICCOMO, Medis Medizinische Messtechnik GmbH, Ilmenau, Germany) was used for automated blood pressure measurements on the right arm and with an appropriate cuff width at standard time points as reported [31]. ICG analysis was performed with four electrodes (two on the axillary line under the thorax and two in the neck) eliminating skin resistance. The examination was performed after stabilization of cardiovascular function in standing position. Parameters used in this study were: blood pressures [systolic (SBP), diastolic (DBP), mean arterial pressure (MAP), pulse pressure (PP)], and cardiac output parameters [heart rate (HR), stroke volume (SV), cardiac output (CO) and cardiac index (CO/body surface area (BSA)].

### Bio-impedance spectrum analysis

The body composition and fluid balance were measured by a multiple frequency bioelectrical impedance analyser (Maltron Bioscan 920-II, Maltron International LTD, Essex, UK) in supine position with stretched arms and legs, without socks or shoes [20]. Two electrodes, receiving the electrical signal, were placed on the dorsal surfaces of the wrist and ankle at the level of the process of the radial and ulnar resp. fibular and tibial bones. Two other electrodes, sending the electrical signal, were attached to the third metacarpal bone of the right hand and right foot. The applied current was 0.6 mA with a frequency of 5, 50, 100 and 200 kHz during 5 seconds. The estimated Total Body Water (TBW) volume totals intracellular water (ICW) and extracellular water (ECW), which in turn is the sum of interstitial water, transcellular water and plasma volume. ECW was expressed as a fraction of total body water (ECW%) to correct for body constitution.

### Statistics

An independent t-test at 5% significance level was used for intergroup comparison of continuous demographic data. Chi-square test was used for categorical demographic variables. Normality was checked via Shapiro-Wilk. Data were presented as mean ± SD or n (%).

Linear Mixed Models for repeated measurements were used to calculate differences in cardiovascular and volume parameters between UP and EPE/LPE/GH/EH/SGA. A random patient effect was used to correct for the correlation between trimestrial measurements of a pregnancy, due to intrasubject repeated measures. Fixed effects of trimester and outcome, as well as their interaction term were specified. The fixed effects structure was simplified by using a significance level of 5%. Analyses were done in SAS (SAS 9.4, Institute Inc., Cary, NC, USA). A correction for demographical influences (BMI, nulliparity and age) on the cardiovascular parameters was implemented in the linear mixed model. Corrections for multiple testing were not implemented.

## Results

In the ongoing Hasselt University Research Project of Maternal Venous Hemodynamics, a total of 1763 women were assessed between 2011–2017, and categorized according to gestational outcome as shown in the flow chart (Fig 1). In these groups, BIA measurements in first and/or second and/or third trimester were available as presented (Fig 1).

Patient demographics are shown in Table 1. Differences between complicated pregnancies and UP were observed for gestational age at assessment, maternal BMI and parity, and in neonatal outcome parameters. Maternal age was higher in EP, smoking rate was higher in SGA and lower in LPE. Medication use was higher in all hypertension groups. At assessment, four women with uncomplicated pregnancy were using Acetyl Acetic Acid or Low Molecular Weight Heparins, whereas 1 in 4 women with EH did not take the antihypertensive medication as prescribed (last intake ≥ 24h before assessment).

**Table 1:** Patient and outcome characteristics of the study population.

	UP (n = 1068)	EPE (n = 75)	LPE (n = 177)	GH (n = 139)	SGA (n = 129)	EH (n = 43)
**Characteristics at inclusion**						
Maternal age (years)	30.4±5.9	30.3±5.3	30.0±5.2	30.2±4.4	29.8±4.9	32.9±5.1*
Gestational age at assessment (weeks)						
*First trimester*	12.2±0.8	12.9±0.5*	11.8±0.8*	12.2±0.7	12.1±0.7	12.4±1.1
*Second trimester*	20.7±1.6	25.2±2.3*	22.0±2.3*	21.5±2.7*	20.8±1.8	21.9±3.9*
*Third trimester*	33.4±3.2	31.5±1.5*	36.7±2.4*	36.8±2.7*	33.4±2.9	34.1±3.0
Pre-pregnancy BMI (kg/m^2^)	24.6±4.9	26.9±6.2*	26.1±5.7*	26.4±6.0*	23.9±5.2	27.1±5.7*
Nulliparity (n,% women)	502 (47)	51 (68)*	129 (73)*	88 (63)*	64 (50)	21 (49)
Cigarette smoker (n,% women)	203 (19)	7 (10)	12 (7)*	21 (15)	48 (37)*	6 (15)
Medication (n, % assessments)						
• non-cardiovascular	201 (12)	8 (9)	15 (7)	16 (9)	26 (13)	2 (3)
• antihypertensive/anticoagulants	69 (4)	22 (25)*	60 (28)*	47 (25)*	4 (2)	54 (75)*
**Outcome characteristics**						
Birth weight, g	3380±535	1280±536*	2915±652*	3069±744*	2384±597*	2941±914*
Birth weight, percentile	55±27	27±26*	43±31	45±32	4.4±2*	45±32
Gestational age at delivery (weeks)	39.2±1.8	30.4±2.8*	37.7±1.8*	38.4±2.6*	38.0±3.4*	37.6±3.8*

UP, uncomplicated pregnancy; EPE, early-onset preeclampsia; LPE, late-onset preeclampsia; GH, gestational hypertension; SGA, small for gestational age neonates; EH, essential hypertension. Data are presented as means ± SD or n (%).

*p<0.05 = significantly different from UP.

Table 2 shows the inter-trimestrial differences of fluid parameters within each group. Irrespective of gestational outcome, TBW, ECW, ICW and ECW% increase significantly in all groups from first-to-second trimester and from second-to-third trimester, with a borderline exception for first-to-second trimester ICW increase in LPE (p = 0,06), and ECW% in EPE (p = 0,08). In each group, the relative increase of ECW is higher than ICW, as is illustrated by growing trimestrial ECW% in each group.

**Table 2:** Average hemodynamic evolution throughout pregnancy of total body water (TBW), extracellular water (ECW), intracellular water (ICW) and ECW/TBW (ECW%).

	*Pregnancy outcome*	*First trimester*	*ΔT1-T2* *(P-value)*	*Second trimester*	*ΔT2-T3* *(P-value)*	*Third trimester*
***TBW (L)***						
	**UP**	33.2(32.8–33.6)	0.001	34.2(33.8–34.5)	0.001	35.3(34.8–35.9)
	**EPE**	35.0(33.0–36.9)	0.012	37.2(35.7–38.7)	0.010	40.9(39.3–42.6)
	**LPE**	33.0(31.8–34.1)	0.044	34.0(32.9–35.1)	0.001	40.1(39.2–40.9)
	**GH**	33.1(32.0–34.2)	0.020	34.1(33.1–35.2)	0.001	37.1(36.2–38.1)
	**SGA**	32.0(31.3–32.8)	0.001	33.0(32.3–33.7)	0.001	34.2(33.4–34.9)
	**EH**	33.1(32.8–33.5)	0.001	34.1(33.8–34.5)	0.001	35.4(34.8–35.9)
***ECW (L)***						
	**UP**	14.2(14.0–14.4)	0.001	14.9(14.7–15.1)	0.001	15.9(15.6–16.2)
	**EPE**	15.5(14.6–16.5)	0.005	16.7(15.9–17.5)	0.001	20.1(19.2–20.9)
	**LPE**	14.2(13.6–14.8)	0.002	14.9(14.3–15.5)	0.001	19.1(18.6–19.5)
	**GH**	14.3(13.7–14.8)	0.001	14.9(14.4–15.5)	0.001	16.8(16.3–17.3)
	**SGA**	13.7(13.3–14.1)	0.001	14.4(13.9–14.7)	0.001	15.3(14.9–15.7)
	**EH**	14.2(14.0–14.4)	0.001	14.8(14.7–15.0)	0.001	15.9(15.6–16.1)
***ICW (L)***						
	**UP**	18.9(18.8–19.1)	0.001	19.2(19.1–19.4)	0.001	19.7(19.5–19.9)
	**EPE**	19.6(18.9–20.4)	0.001	20.5(19.8–21.1)	0.023	21.1(20.4–21.8)
	**LPE**	18.6(18.2–19.1)	0.062	19.0(18.5–19.4)	0.001	21.1(20.8–21.5)
	**GH**	18.9(18.5–19.4)	0.005	19.3(18.8–19.7)	0.001	20.3(19.9–20.6)
	**SGA**	18.4(18.0–18.7)	0.001	18.7(18.3–19.0)	0.001	19.1(18.8–19.4)
	**EH**	18.9(18.8–19.1)	0.001	19.2(19.1–19.4)	0.001	19.7(19.5–19.9)
***ECW%***						
	**UP**	43.0(42.8–43.1)	0.001	43.7(43.5–43.8)	0.001	44.6(44.3–44.9)
	**EPE**	44.4(43.5–45.4)	0.078	45.1(44.4–45.9)	0.001	48.6(47.9–49.4)
	**LPE**	43.3(42.8–43.8)	0.002	44.0(43.5–44.5)	0.001	47.2(46.8–47.6)
	**GH**	43.3(42.7–43.8)	0.002	43.8(43.4–44.4)	0.001	45.6(45.2–46.1)
	**SGA**	42.9(42.7–43.1)	0.001	43.6(43.4–43.8)	0.001	44.5(44.2–44.7)
	**EH**	42.9(42.8–43.1)	0.001	43.6(43.4–43.8)	0.001	44.5(44.3–44.8)

UP, uncomplicated pregnancy; EPE, early-onset preeclampsia; LPE, late-onset preeclampsia; GH, gestational hypertension; SGA, small for gestational age neonates; EH, essential hypertension; TBW, total body water; ECW, extracellular water; ICW, intracellular water; ECW%, fraction of extracellular water. Data are presented as corrected least-square means ± SD, representative for a nulliparous woman aged 30 years with BMI 23 kg/m^2^. Numbers of assessments per group per trimester are presented in Fig 1.

Table 3 shows the inter-trimestrial differences of cardiac output (CO), both in absolute values (L/min) corrected for parity, age and BMI, or indexed to body surface area (CI in L/min/m^2^). CO is higher in each consecutive trimester of UP, and this is not observed in any of the other groups. A similar trend is observed for CI in EPE, LPE and GH, but not in GH and SGA. An overall lack of rise in CO or CI is observed in EPE and LPE.

**Table 3:** Average evolution throughout pregnancy of cardiac output (CO) and cardiac index (CI).

	***Pregnancy outcome***	***First trimester***	***ΔT1-T2*** ***(P-value)***	***Second trimester***	***ΔT2-T3*** ***(P-value)***	***Third trimester***
***CO (L/min)***						
	**UP**	6.9(6.8–7.0)	0.001	7.7(7.6–7.8)	0.613	7.6(7.5–7.8)
	**EPE**	6.5(5.9–7.1)	0.764	6.4(6.0–6.8)	0.087	6.8(6.5–7.2)
	**LPE**	6.8(5.8–7.2)	0.139	7.1(6.8–7.5)	0.963	7.1(6.9–7.3)
	**GH**	6.6(6.3–6.8)	0.001	7.3(7.1–7.6)	0.313	7.3(7.0–7.5)
	**SGA**	6.5(6.3–6.8)	0.004	7.3(7.1–7.5)	0.600	7.2(7.0–7.5)
	**EH**	6.9(6.8–7.0)	0.001	7.7(7.6–7.8)	0.800	7.7(7.5–7.9)
***CI (L/min/m^2^)***						
	**UP**	4.0(3.9–4.1)	0.001	4.4(4.3–4.4)	0.001	4.2(4.1–4.3)
	**EPE**	3.7(3.4–4.1)	0.705	3.7(3.5–3.9)	0.543	3.6(3.4–3.8)
	**LPE**	4.0(3.7–4.1)	0.383	4.0(3.9–4.2)	0.020	3.8(3.7–3.9)
	**GH**	3.9(3.7–4.1)	0.001	4.3(4.1–4.4)	0.001	3.9(3.7–4.0)
	**SGA**	3.9(3.8–4.0)	0.001	4.2(4.1–4.3)	0.001	4.1(4.0–4.2)
	**EH**	3.9(3.6–4.1)	0.001	4.3(4.0–4.5)	0.090	4.5(4.2–4.7)

UP, uncomplicated pregnancy; EPE, early-onset preeclampsia; LPE, late-onset preeclampsia; GH, gestational hypertension; SGA, small for gestational age neonates; EH, essential hypertension; CO, cardiac output; CI, cardiac index. CO is expressed as corrected least-square means ± SD, representative for a nulliparous woman aged 30 years with BMI 23 kg/m^2^. CI is expressed as CO/body surface area, and corrected values representative for a nulliparous woman aged 30 years. Numbers of assessments per group per trimester are presented in Fig 1.

Table 4 presents the comparison between UP and other groups for first trimester values and for gestational evolution of body water volumes and cardiac output. In EPE, first trimester ECW and ECW% are high, and gestational changes of all body water volumes and cardiac output are different. In SGA, first trimester TBW, ECW, ICW, CO and CI are low, whereas gestational changes are not abnormal. In LPE and GH, first trimester values of TBW, ECW, ICW and ECW% are normal, but gestational changes are not. In LPE, but not GH, this is associated with abnormal gestational change of CO. Finally for EH, no differences with UP were observed.

**Table 4:** Positive or negative differences for first trimester values of total body water (TBW), extracellular water (ECW), intracellular water (ICW), ECW/TBW (ECW%), cardiac output (CO) and cardiac index (CI) as well as their inter-trimestrial changes relative to uncomplicated pregnancies.

**Pregnancy outcome**	**Parameter**	**First trimester values**		**Gestational evolution**
		Difference compared to UP	P-value	P-value
***EPE***				
	**TBW**	1,78	0,078	0,001
	**ECW**	1,3	0,01	<0,001
	**ICW**	0,66	0,086	0,039
	**ECW%**	1,48	0,002	<0,0001
	**CO**	-0,42	0,173	0,012
	**CI**	-0,27	0,0119	0,045
***LPE***				
	**TBW**	0,006	0,991	<0,001
	**ECW**	0,099	0,746	<0,001
	**ICW**	-0,154	0,524	<0,001
	**ECW%**	0,385	0,179	<0,001
	**CO**	-0,079	0,647	0,015
	**CI**	-0,064	0,501	0,0021
***GH***				
	**TBW**	-0,024	0,967	0,005
	**ECW**	0,107	0,707	0,009
	**ICW**	-0,0004	0,998	0,019
	**ECW%**	0,366	0,176	0,0359
	**CO**	-0,364	0,001	0,628
	**CI**	-0,147	0,122	0,0479
***EH***				
	**TBW**	0,236	0,731	0,6781
	**ECW**	0,106	0,779	0,957
	**ICW**	0,158	0,6312	0,51
	**ECW%**	0,326	0,339	0,213
	**CO**	0,026	0,887	0,068
	**CI**	-0,169	0,161	0,0072
***SGA***				
	**TBW**	-1,13	0,001	0,8987
	**ECW**	-0,51	0,007	0,212
	**ICW**	-0,59	<0,001	0,48
	**ECW%**	-0,15	0,397	0,1214
	**CO**	-0,38	<0,001	0,596
	**CI**	-0,13	0,012	0,789

UP, uncomplicated pregnancy; EPE, early-onset preeclampsia; LPE, late-onset preeclampsia; GH, gestational hypertension; EH, essential hypertension; SGA, small for gestational age neonates; TBW, total body water; ECW, extracellular water; ICW, intracellular water; ECW%, fraction of extracellular water; CO, cardiac output; CI, cardiac index. Numbers of assessments per group per trimester are presented in Fig 1.

**Fig 2:**
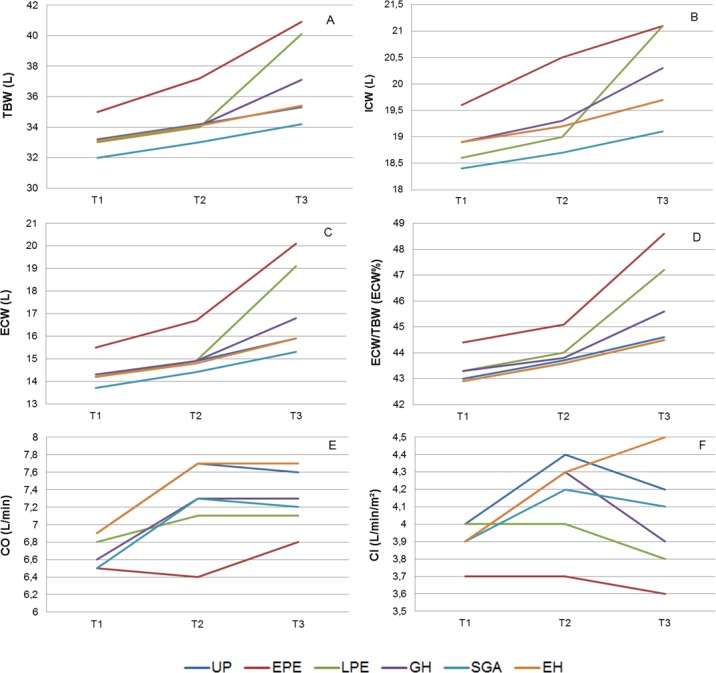
Trimestrial values of total body water (TBW, A), intracellular water (ICW, B), extracellular water (ECW, C), fraction of extracellular water (ECW%, D), cardiac output (CO, E) and cardiac index (CI, F) in uncomplicated pregnancies (UP), early onset preeclampsia (EPE), late onset preeclampsia (LPE), gestational hypertension (GH), essential hypertension (EH) and normotensive women with neonates small for gestational age (SGA).

Fig 2 presents graphically the inter-trimestrial differences of TBW (A), ECW% (C), CO (E) and CI (F) within and between groups.

**Fig 3:**
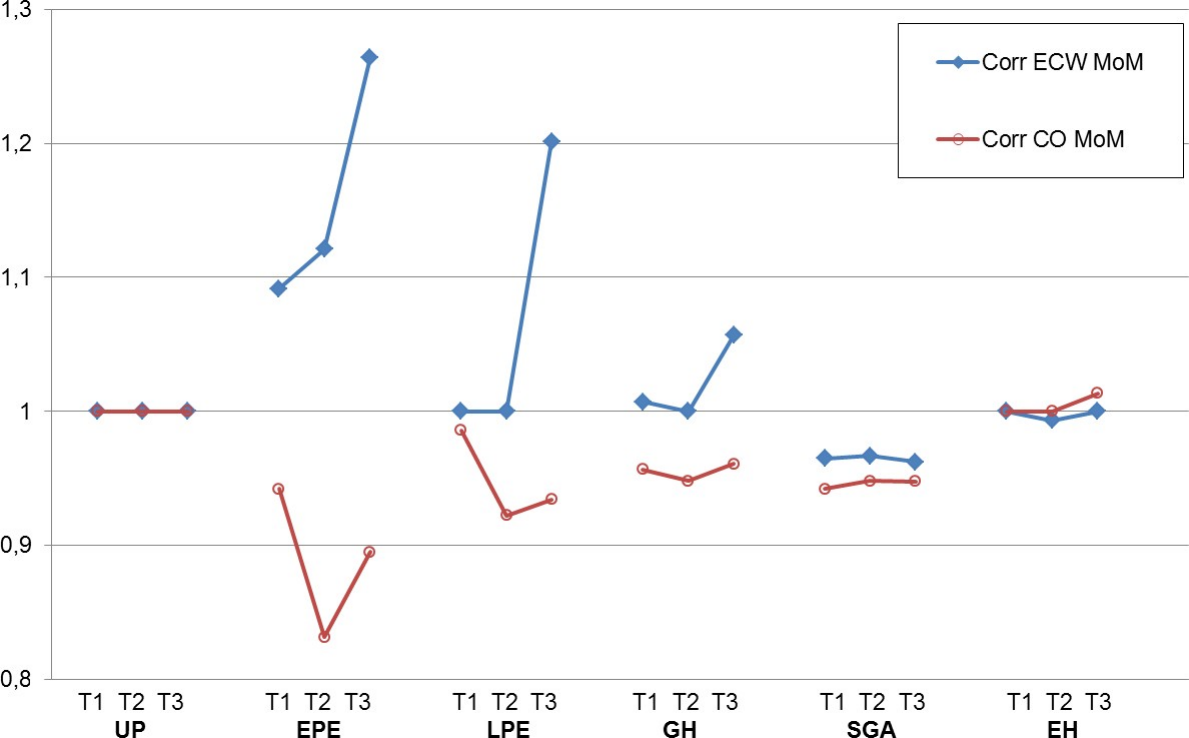
Combined presentation of trimestrial values of extracellular water (ECW) and cardiac output (CO), expressed as multiples of the mean (MoM), in uncomplicated pregnancies (UP), early onset preeclampsia (EPE), late onset preeclampsia (LPE), gestational hypertension (GH), essential hypertension (EH) and normotensive women with neonates small for gestational age (SGA). Severely, moderately and mildly opposite values and/or changes of ECW MoM and CO MoM (MoM < or > 1) are present in EPE, LPE and GH respectively.

Fig 3 shows the inter-trimestrial differences for both CO and ECW, expressed as multiples of the mean (MoM). This presentation clearly illustrates the phenotype-specific differences of volume homeostasis in the pathology groups. Evolutions of CO and ECW are severely, moderately and mildly discrepant in EPE, LPE and GH respectively (MoM <1 or > 1). SGA MoM values are lower than UP and show a similar inter-trimestrial evolution. No differences are present between UP and EH.

Detailed information on patient’s demographics and impedance measurements of volumes and cardiac output are attached as supplement in S1 Table.

## Discussion

The most important findings of this study are that (1) all pregnancies show an increase of body water volumes, irrespective of outcome and body composition, and (2) typical features of abnormal volume regulation are observed in pregnancies with abnormal outcome, such as a persistently low fraction of TBW, ECW and ICW in SGA pregnancies, a lack of CO increase in EPE and LPE, and severely, moderately and mildly discrepant evolutions of CO and ECW MoM values in EPE, LPE and GH respectively. No differences are found between UP and EH.

The strengths of this study are the large numbers of included subjects and assessments, all performed according to a rigid standard protocol [32], and the low number of missing outcome data. Next to this, all included subjects also had an extensive maternal hemodynamics assessment including all major parts of the circulation, using combined ECG-Doppler and impedance cardiography, which are or will be reported elsewhere, and which allow interpretation of volume measurements within the global functioning of the maternal cardiovascular circuit. In our analysis, device- and population-specific reference values were used, and measurements were corrected for age, parity and BMI [33] or indexed for body surface area [34]. We acknowledge that the semi-longitudinal observations in our cohorts is a limitation, and confirmation from a large prospective study is required. Our study population represents a large fraction of women with complicated third trimester pregnancies, relating to the inclusion of pregnancies at diagnosis of disease, where ideally every women should have had 1 measurement per trimester: this bias may limit the statistical power of our analysis. The lack of data on serum or urine electrolyte concentrations and haematocrit values is another weakness, as electrical conduction is known to depend mainly on electrolytes and water [4,23]. Confirmation of our BIA results by so-called golden standard technologies for assessment of body fluid compartments is warranted [3].

Several reports mention an increase of body water volume in every pregnant woman, a process in which the relative rise of ECW rise is larger than of ICW [20, 24]. The well-known gestational plasma volume expansion [35] is part of this process. This plasma volume change is generally considered to result from an early gestational generalized vasodilatation, triggering activation of volume retaining mechanisms towards increase of intravascular volume [36] and aiming for an adequate perfusion of the uteroplacental circulation in the growing uterus. Our results add to these data that gestational body water expansion is not associated with increase of cardiac output in pregnancies destined to develop preeclampsia (Tables 2 and 3).

Our data show that first trimester TBW volume is abnormally low in SGA pregnancies. This observation is in line with other reports on plasma volume, a component of TBW, that repeatedly has been reported lower in SGA pregnancies [37, 38]. This condition is known to be associated with lower preload, SV and CO [37, 39–41], important maternal contributors to neonatal birth weight [39, 42].

Reduced plasma volume expansion in EPE has been reported [43], as well as a more pronounced rise of ECW than ICW [20]. Our results add to this that higher ECW and ECW% than UP are already present in EPE from the first trimester onwards, and this co-exists with a lack of increase of CO. In combination, these observations suggest an increased extravasation of intravascular fluids into the interstitial and transcellular compartments throughout every stage of EPE pregnancy. In first trimester pathophysiology of EPE, increase of mean arterial pressure, uterine arterial resistance, sympathetic tone and serum concentrations of endothelial dysfunction have been reported [43, 44]. Our data suggest to add the phenomenon of extravasation of intravascular fluids to the early gestational features of abnormal maternal hemodynamics of EPE, which could perhaps be useful in screening or prediction of this serious gestational complication, as already mentioned by others [25]. Similarly, the evaluation of body fluid volumes from second to third trimester could be potentially useful in prediction of late onset preeclampsia or gestational hypertension (Figs 2 and 3). Finally, a rationalized management of abnormal body fluid volume changes may also be a new target for prevention and/or follow up of pregnancies, complicated with GHD and/or SGA [45].

Another important observation of this study is the striking similarity of body water volumes and cardiac output in every gestational trimester of UP and EH without superimposed PE. As such, body water homeostasis may be an important predictive discriminator between normal and abnormal outcome of pregnancies in women with essential hypertension. This observation needs confirmation from more targeted research.

Finally, our data add to the growing body of evidence that, despite the reported limitations, the bio-impedance technology is a very simple method to assess body volume changes at every stage of pregnancy, which offers perspectives towards a potentially valuable and easily applicable technology in screening and management of GHD.

We conclude from our observations presented in this report that body water volume increases in every pregnant woman, starting from first trimester abnormal values in EPE and SGA, or becoming abnormal in later stages of LPE and GH. BIA is a useful and easy to perform method to evaluate these body volume changes. These data invite to further explore BIA volume measurements as a new tool in screening and management of GHD and/or SGA.

## Supporting information

S1 Table Details on patient’s demographics and impedance measurements of volumes and cardiac output. (PDF)
