# Supplementary material for: Body fluid volume homeostasis is abnormal in pregnancies complicated with hypertension and/or poor fetal growth
Source: PLoS One. 2018 Nov 1;13(11):e0206257. doi: 10.1371/journal.pone.0206257 (PMC6211673; doi:10.1371/journal.pone.0206257)
Supplement: S1 Table — (PDF) [file pone.0206257.s001.pdf]

ID Code	Abdo Surgery	Hypertension	Diabetes	Thrombofilia	Allergy	Race	Parity (0=nulli, 1=multi)	G	P	A	Start weight	Length	BMI	History IU death
1	0	0	0	0	0		1	2	1	0	55	1,65	20,2	0
2	0	0	0	0	0		0	1	0	0	93	1,68	32,95	0
3	0	0	0	0	0		0	1	0	0	53	1,56	21,78	0
4	0	1	0	0	0		1	2	1	0	44	1,58	17,63	0
5	0	0	0	0	0		1	9	4	4	65	1,65	23,88	0
6	0	0	0	0	0		1	5	1	3	58	1,64	21,56	0
7	0	0	0	0	0		0	1	0	0	80	1,7	27,68	0
8	0	0	0	0	0		0	2	0	1	48	1,49	21,62	0
9	0	0	0	0	0		0	1	0	0	76	1,68	26,93	0
10	0	0	0	0	0		1	5	2	2	51	1,68	18,07	0
11	0	0	0	0	0		1	3	1	1	56	1,5	24,89	0
12	0	1	0	0	0		1	7	4	2	49	1,54	20,66	0
13	0	0	0	0	0		0	1	0	0	60	1,68	21,26	0
14	0	0	1	0	0		0	1	0	0	47	1,62	17,91	0
15	0	0	0	0	0	1	0	1	0	1	55	1,68	19,49	0
16	0	0	0	0	0	1	0	1	0	1	55	1,68	19,49	0
17	2	0	0	0	0	3	1	2	1	0	63	1,72	21,3	0
18	0	0	0	0	0	1	1	2	1	0	96	1,72	32,45	0
19	0	0	0	0	0	1	1	2	1	0	96	1,72	32,45	0
20	1	0	0	0	0	3	0	2	0	1	42	1,54	17,71	0
21	1	0	0	0	0	3	0	2	0	1	42	1,54	17,71	0
22	0	0	0	0	1	1	1	2	1	0	67	1,57	27,18	0
23	0	0	0	0	0	6	1	3	1	1	73	1,6	28,52	0
24	1	1	0	0	0	1	1	2	1	0	72	1,55	29,97	0
25	1	1	0	0	0	1	1	2	1	0	72	1,55	29,97	0
26	2	0	0	0	0	3	1	3	2	0	85	1,5	37,78	0
27	2	0	0	0	0	3	1	3	2	0	85	1,5	37,78	0
28	0	0	0	0	0	1	0	1	0	0	60	1,67	21,51	0
29	0	0	0	0	0	1	0	1	0	0	60	1,67	21,51	0
30	1	0	0	0	1	1	1	4	1	2	80	1,72	27,04	0
31	1	0	0	0	1	1	1	4	1	2	80	1,72	27,04	0
32	1	0	0	0	1	3	0	1	0	0	66	1,58	26,44	0

33	1	0	0	0	1	3	0	1	0	0	66	1,58	26,44	0
34	0	1	0	0	0		1	4	2	1	59	1,47	27,30	0
35	0	0	0	0	0		1	2	1	0	60	1,67	21,51	0
36	0	0	0	0	0		0	1	0	0	68	1,78	21,46	0
37	0	0	0	0	0		0	1	0	0	49	1,64	18,22	0
38	0	0	0	0	0		0	1	0	0	68	1,68	24,09	0
39	0	0	0	0	0		0	1	0	0	68	1,68	24,09	0
40	0	0	0	0	0		0	2	0	1	72	1,65	26,45	0
41	0	0	0	0	0		0	2	0	1	57	1,6	22,27	0
42	0	0	0	0	0		0	1	0	0	53	1,6	20,7	0
43	0	0	0	0	0		0	1	0	0	86	1,65	31,59	0
44	0	0	0	0	0		0	1	0	0	51	1,58	20,43	0
45	0	0	0	0	0		1	2	1	0	63	1,6	24,61	0
46	0	0	0	0	0		0	2	0	1	64	1,72	21,63	0
47	0	0	0	0	0		0	1	0	0	59	1,6	23,05	0
48	0	0	0	0	0		0	1	0	0	67	1,65	24,61	0
49	0	0	0	0	0		1	2	1	0	63	1,6	24,61	0
50	0	0	0	0	1	1	1	2	1	0	52,3	1,53	22,34	0
51	0	0	0	0	0		0	1	0	0	64	1,61	24,69	0
52	0	0	0	0	0		1	5	3	1	55	1,68	19,49	0
53	0	0	0	0	0		0	1	0	0	61	1,77	19,47	0
54	0	0	0	0	0		0	1	0	0	45	1,53	19,22	0
55	0	0	0	0	0		0	1	0	0	49	1,64	18,22	0
56	0	0	0	0	0		0	1	0	0	86	1,72	29,07	0
57	0	0	0	0	0		0	1	0	0	86	1,72	29,07	0
58	0	0	0	0	0		1	2	1	0	45	1,6	17,58	0
59	0	0	0	0	0		0	1	0	0	64	1,7	22,15	0
60	0	0	0	0	0		0	1	0	0	68	1,64	25,28	0
61	0	0	0	0	0		0	1	0	0	64	1,7	22,15	0
62	0	0	0	0	0		0	1	0	0	86	1,72	29,07	0
63	0	0	0	0	0		0	1	0	0	63	1,68	22,32	0
64	0	0	0	0	0		0	2	0	1	69	1,57	27,99	0
65	0	0	0	0	0		1	3	1	1	99	1,65	36,36	0

66	0	0	0	0	0			1	2	1	0	57	1,63	21,45	0
67	0	0	0	0	0			0	2	0	1	49	1,65	18	0
68	0	1	0	0	0			1	2	1	0	58	1,73	19,38	0
69	0	1	0	0	0			1	2	1	0	58	1,73	19,38	0
70	0	0	0	0	0			1	3	2	0	75	1,65	27,55	0
71	0	0	0	0	0			0	1	0	0	58	1,68	20,55	0
72	0	0	0	0	0			0	1	0	0	53	1,55	22,06	0
73	0	0	0	0	0			1	2	1	0	58	1,61	22,38	0
74	0	0	0	0	0			1	4	2	1	89	1,76	28,73	0
75	0	0	0	0	0			0	1	0	0	45	1,6	17,58	0
76	0	0	0	0	0			1	3	2	0	88	1,68	31,18	0
77	0	0	0	0	0			1	3	1	1	55	1,65	20,2	0
78	0	0	0	0	0			1	3	1	1	58	1,85	16,95	0
79	0	0	0	0	0			1	2	1	0	60	1,63	22,58	0
80	0	0	0	0	0			0	1	0	0	65	1,73	21,72	0
81	0	0	0	0	0			0	1	0	0	53	1,54	22,35	0
82	0	0	0	0	0			0	1	0	0	65	1,73	21,72	0
83	0	0	0	0	0			1	3	2	0	86	1,68	30,47	0
84	0	0	0	0	1	2		0	1	0	0	94	1,65	34,53	0
85	0	0	0	0	1	2		0	1	0	0	94	1,65	34,53	0
86	0	0	0	0	0			0	2	0	1	66	1,66	23,95	0
87	0	0	0	0	0			1	2	1	0		1,68		0
88	0	0	0	0	0			0	1	0	0	58	1,73	19,38	0
89	0	0	0	0	0			1	4	3	0	64	1,64	23,8	0
90	0	0	0	0	0			1	4	3	0	64	1,64	23,8	0
91	0	1	0	0	0			1	2	1	0	58	1,63	21,83	0
92	0	1	0	0	0			1	2	1	0	58	1,63	21,83	0
93	0	0	0	0	0			0	1	0	0	68	1,58	27,24	0
94	0	0	0	0	0			1	2	1	0	80	1,65	29,38	0
95	0	0	0	0	0			0	1	0	0	58	1,55	24,14	0
96	0	0	0	0	0			1	2	1	0	71	1,6	27,73	0
97	0	0	0	0	0			0	1	0	0	83	1,68	29,41	0
98	0	0	0	0	0			1	2	1	0	68	1,6	26,56	0

99	0	0	0	0	0		0	1	0	0	60	1,63	22,58	0
100	0	0	0	0	0		1	2	1	0	68	1,73	22,72	0
101	0	0	0	0	0		1	3	1	1	69	1,66	25,04	1
102	0	0	0	0	0		0	2	0	1	68	1,7	23,53	0
103	0	0	0	0	0		0	1	0	0	62	1,63	23,34	0
104	0	0	0	0	0		0	1	0	0	54	1,65	19,83	0
105	0	0	0	0	0		0	1	0	0	52	1,51	22,81	0
106	0	0	0	0	0		1	2	1	0	45	1,6	17,58	0
107	0	0	0	0	0		0	1	0	0	52	1,51	22,81	0
108	0	0	0	0	0		0	1	0	0	53	1,67	19	0
109	0	0	0	0	0		1	4	3	0	94	1,6	36,72	0
110	0	0	0	0	0		1	4	3	0	94	1,6	36,72	0
111	0	0	0	0	0		1	4	2	1	59	1,61	22,76	0
112	0	0	0	0	0		1	4	2	1	59	1,61	22,76	0
113	0	0	0	0	0		0	1	0	0	64	1,67	22,95	0
114	0	0	0	0	0		0	1	0	0	60	1,7	20,76	0
115	0	0	0	0	0		0	3	0	2	52	1,68	18,42	0
116	0	0	0	0	0		1	4	3	0	64	1,57	25,96	1
117	0	0	0	0	0		0	2	0	1	59	1,67	21,16	0
118	0	1	0	0	0		1	2	1	0	73	1,65	26,81	0
119	0	0	0	0	0		0	1	0	0	79	1,68	27,99	0
120	0	0	0	0	0		0	2	0	1	75	1,7	25,95	0
121	0	0	0	0	0		1	2	1	0	55	1,67	19,72	0
122	0	0	0	0	0		0	1	0	0	108	1,74	35,67	0
123	0	0	0	0	0		1	2	1	0				1
124	0	0	0	0	0		0	2	0	1	75	1,7	25,95	0
125	0	0	0	0	0	1	1	9	7	1	82	1,7	28,37	0
126	0	0	0	0	0		0	1	0	0	108	1,74	35,67	0
127	0	0	0	0	0		1	2	1	0				1
128	0	0	0	0	0		1	2	1	0				1
129	0	1	0	0	0		1	2	1	0	84	1,72	28,39	0
130	0	0	0	0	0		1	2	1	0	73	1,72	24,68	0
131	0	0	0	0	0		1	2	1	0	64	1,67	22,95	0

132	0	0	0	0	0		1	2	1	0	73	1,72	24,68	0
133	0	0	0	0	0		1	2	1	0	69	1,64	25,65	0
134	0	1	0	0	0		1	4	2	1	77	1,68	27,28	0
135	0	0	0	0	0		0	1	0	0	56	1,5	24,89	0
136	0	0	0	0	0		1	9	1	5	69	1,73	23,05	0
137	0	0	0	0	0		0	1	0	0	81	1,73	27,06	0
138	0	0	0	0	0		1	4	1	2	65	1,69	22,76	0
139	0	0	0	0	0	1	0	1	0	0	80	1,72	27,04	0
140	0	0	0	0	0		1	2	1	0	70	1,66	25,4	0
141	0	0	0	0	0		0	2	0	1	59	1,7	20,42	0
142	0	0	0	0	0		1	4	3	0	71	1,58	28,44	0
143	0	0	0	0	0		1	3	1	1	80	1,74	26,42	0
144	0	0	0	0	0		0	4	0	3	63	1,66	22,86	0
145	0	0	0	0	0		0	4	0	3	63	1,66	22,86	0
146	0	0	0	0	0		0	1	0	0	60	1,68	21,26	0
147	0	0	0	0	0		0	1	0	0	68	1,74	22,46	0
148	0	0	0	0	0		1	4	3	0	84	1,72	28,39	0
149	0	0	0	0	0		1	4	2	1	109	1,7	37,72	0
150	0	0	0	0	0		0	2	0	1	66	1,68	23,38	0
151	0	0	0	0	0		1	2	1	0	83	1,73	27,73	0
152	0	1	0	0	0		1	2	1	0	80	1,7	27,68	0
153	0	0	0	0	0		0	1	0	0	61	1,58	24,44	0
154	0	1	0	0	0		1	2	1	0	80	1,7	27,68	0
155	0	0	0	0	0		1	3	1	1	52	1,58	20,83	0
156	0	0	0	0	0		1	3	2	2	70	1,7	24,22	0
157	2	0	0	0	0	1	1	2	1	0	79	1,65	29,02	0
158	2	0	0	0	1	1	0	1	0	0	52	1,59	20,57	0
159	2	0	0	0	1	1	0	1	0	0	52	1,59	20,57	0
160	2	0	0	0	0	1	1	2	1	0	60	1,63	22,58	0
161	2	0	0	0	0	1	1	2	1	0	60	1,63	22,58	0
162	1	0	0	0	0	1	0	2	0	1	71	1,78	22,41	0
163	1	0	0	0	0	1	0	2	0	1	71	1,78	22,41	0
164	1	0	0	0	0	1	0	1	0	0	89	1,65	32,69	0

165	1	0	0	0	0	1	0	1	0	0	89	1,65	32,69	0
166	1	0	0	0	1	2	1	5	1	3	72	1,72	24,34	0
167	1	0	0	0	1	2	1	5	1	3	72	1,72	24,34	0
168	1	1	0	0	0		1	2	1	0	60	1,6	23,44	0
169	2	0	0	0	0	1	0	1	0	0	87	1,62	33,15	0
170	1	1	0	0	1	2	1	2	1	0	53	1,6	20,7	0
171	1	1	0	0	1	2	1	2	1	0	53	1,6	20,7	0
172	0	0	0	0	0		0	1	0	0	68	1,76	21,95	0
173	0	0	0	0	0		1	3	1	1	78	1,68	27,64	0
174	0	1	0	0	0		1	4	1	2	62	1,54	26,14	0
175	0	0	0	0	0		0	2	0	1	70	1,73	23,39	0
176	0	0	0	0	0		1	2	1	0	68	1,65	24,98	1
177	0	0	0	0	0		1	6	3	2	48	1,57	19,47	0
178	0	1	0	0	0		1	2	1	0	67	1,7	23,18	0
179	0	1	0	0	0		1	2	1	0	67	1,7	23,18	0
180	0	1	0	0	0		1	2	1	0	67	1,7	23,18	0
181	2	0	0	0	0	3	1	4	2	1	78	1,55	32,47	0
182	0	1	0	0	0		1	2	1	0	62	1,57	25,15	0
183	0	0	0	0	0		1	2	1	0	53	1,7	18,34	0
184	0	0	0	0	0		1	2	1	0	52	1,58	20,83	0
185	0	0	0	0	0		1	4	2	1				0
186	0	0	0	0	0		0	1	0	0	73	1,71	24,96	0
187	0	0	0	0	0		1	5	2	2	92	1,67	32,99	0
188	0	0	0	0	0		1	3	1	1	93	1,65	34,16	0
189	0	0	0	0	0		1	3	1	1	72	1,68	25,51	0
190	1	1	0	0	0		1	2	1	0	85	1,72	28,73	0
191	1	1	0	0	0		1	2	1	0	85	1,72	28,73	0
192	0	0	0	0	0		1	2	1	0	66	1,67	23,67	0
193	1	0	0	0	1		1	3	1	1	52	1,62	19,81	0
194	0	0	0	0	0		1	2	1	0	59	1,64	21,94	0
195	0	0	0	0	0		1	2	1	0	69	1,67	24,74	0
196	1	0	0	0	1	1	1	3	1	1	51	1,57	20,69	0
197	1	0	0	0	1	1	1	3	1	1	51	1,57	20,69	0

198	1	0	0	0	0		0	1	0	0	72	1,63	27,1	0
199	1	0	0	0	0		1	6	3	2	72	1,74	23,78	0
200	1	0	0	0	0		1	5	3	2	70	1,62	26,67	0
201	2	1	0	1	1	1	1	2	1	0	65	1,71	22,23	0
202	2	1	0	1	1	1	1	2	1	0	65	1,71	22,23	0
203	2	1	0	1	1	1	1	2	1	0	65	1,71	22,23	0
204	0	0	1	0	0		0	1	0	0	104	1,65	38,2	0
205	0	0	1	0	0		0	1	0	0	104	1,65	38,2	0
206	0	0	0	0	0		1	2	1	0	98	1,67	35,14	0
207	0	0	0	0	0		1	2	1	0	98	1,67	35,14	0
208	0	0	0	0	0		0	1	0	0	80	1,72	27,04	0
209	0	0	1	0	0		0	2	0	1	94	1,6	36,72	0
210	0	0	0	0	0		0	1	0	0	56	1,75	18,29	0
211	0	0	0	0	0		0	1	0	0		1,9		0
212	0	0	0	0	0		0	1	0	0	80	1,7	27,68	0
213	0	0	0	0	0		1	4	1	2	60	1,56	24,65	0
214	0	0	0	0	0		1	4	1	2	60	1,56	24,65	0
215	2	0	0	0	0		0	1	0	0	91	1,7	31,49	0
216	2	0	0	0	0		0	1	0	0	91	1,7	31,49	0
217	0	0	0	0	0		1	3	2	0	69	1,68	24,45	0
218	0	1	0	1	0	1	1	3	2	0	62	1,63	23,34	0
219	0	1	0	1	0	1	1	3	2	0	62	1,63	23,34	0
220	0	1	0	1	0	1	1	3	2	0	62	1,63	23,34	0
221	0	0	0	0	0		1	2	1	0	59	1,68	20,9	0
222	0	0	0	0	0		0	1	0	0	92	1,75	30,04	0
223	0	0	0	0	0		1	2	1	0	72	1,6	28,12	1
224	0	0	0	0	0		1	2	1	0	80	1,63	30,11	0
225	0	0	0	0	0		0	1	0	0	58	1,7	20,07	0
226	0	0	0	0	0		1	3	2	0	65	1,75	21,22	0
227	0	0	0	0	0		1	2	1	0	77	1,67	27,61	0
228	0	0	0	0	0		0	1	0	0	72	1,68	25,51	0
229	0	1	1	0	0		1	3	1	1	95	1,75	31,02	0
230	0	1	1	0	0		1	3	1	1	95	1,75	31,02	0

231	1	0	0	0	1	1	1	2	1	0	111	1,68	39,33	1
232	1	0	0	0	1	1	1	2	1	0	111	1,68	39,33	1
233	1	0	0	0	1	1	1	2	1	0	111	1,68	39,33	1
234	2	1	0	0	0	1	1	2	1	0	60	1,6	23,44	0
235	0	0	1	0	0		0	1	0	0	86	1,65	31,59	0
236	0	1	1	0	0		1	4	3	0	91	1,73	30,41	0
237	1	0	1	0	0	1	1	3	1	1	138	1,78	43,56	0
238	1	0	1	0	0	1	1	3	1	1	138	1,78	43,56	0
239	1	0	1	0	1		0	1	0	0	77,4	1,74	25,56	0
240	0	0	1	0	0		0	1	0	0	86	1,59	34,02	0
241	0	0	1	0	0		1	4	2	1	48	1,65	17,63	0
242	0	1	1	0	0		1	2	1	0	105	1,73	35,08	0
243	0	1	1	0	0		1	2	1	0	105	1,73	35,08	0
244	0	0	1	0	0		1	4	3	1	106	1,64	39,41	0
245	0	0	1	0	0		1	5	2	2	68	1,67	24,38	0
246	0	0	1	0	0		1	5	2	2	68	1,67	24,38	0
247	0	0	1	0	0		0	1	0	0	70	1,69	24,51	0
248	0	0	1	0	0		0	1	0	0	70	1,69	24,51	0
249	0	1	0	0	0		1	2	1	0		1,7		0
250	0	0	1	0	0		1	5	3	1	86	1,5	38,22	0
251	0	0	0	0	0	1	1	3	1	1	57	1,62	21,72	0
252	0	0	0	0	0	1	1	3	1	1	57	1,62	21,72	0
253	1	0	0	0	0	1	1	4	3	0	60	1,72	20,28	0
254	1	0	0	0	0	1	1	4	3	0	60	1,72	20,28	0
255	2	0	0	0	0	1	1	2	1	0	63	1,7	21,8	0
256	2	0	0	0	0	1	1	2	1	0	63	1,7	21,8	0
257	1	0	0	0	0	2	1	2	1	0	59	1,62	22,48	0
258	0	0	0	0	1	1	0	1	0	0	92	1,64	34,21	0
259	0	0	0	0	1	1	0	1	0	0	92	1,64	34,21	0
260	0	1	0	0	0		1	2	1	0	52	1,64	19,33	0
261	0	1	0	0	0		1	2	1	0	52	1,64	19,33	0
262	0	0	1	0	0		1	3	1	1	63	1,5	28	0
263	0	0	0	0	0	1	0	1	0	0	76	1,66	27,58	0

264	0	0	0	0	0	1	0	1	0	0	76	1,66	27,58	0
265	0	0	0	0	0	3	1	3	2	0	89	1,73	29,74	0
266	2	0	0	0	0	3	0	2	0	1	59	1,64	21,94	0
267	2	0	0	0	0	3	0	2	0	1	59	1,64	21,94	0
268	0	0	0	1	1	1	1	2	1	0	70	1,66	25,4	0
269	0	0	0	1	1	1	1	2	1	0	70	1,66	25,4	0
270	0	0	0	0	0	2	0	1	0	0	58	1,58	23,23	0
271	0	0	0	0	0	2	0	1	0	0	58	1,58	23,23	0
272	1	0	0	0	0	1	0	1	0	1	68	1,69	23,81	0
273	1	0	0	0	0	1	0	1	0	1	68	1,69	23,81	0
274	0	0	0	0	0	1	0	1	0	0	49	1,6	19,14	0
275	0	0	0	0	0	1	0	1	0	0	49	1,6	19,14	0
276	0	0	0	0	0	1	0	1	0	0	62	1,6	24,22	0
277	0	0	0	0	0	1	0	1	0	0	62	1,6	24,22	0
278	0	0	0	0	0	1	1	3	1	1	72	1,7	24,91	0
279	0	0	0	0	0	1	1	3	1	1	72	1,7	24,91	0
280	0	0	0	0	0	2	1	3	1	1	72	1,7	24,91	0
281	0	0	0	0	0	2	1	3	1	1	72	1,7	24,91	0
282	0	0	0	0	0	3	1	3	2	0	101	1,61	38,96	0
283	0	0	0	0	0	3	1	3	2	0	101	1,61	38,96	0
284	0	0	0	0	0	1	1	2	1	0	74	1,67	26,53	0
285	0	0	0	0	0	1	1	2	1	0	74	1,67	26,53	0
286	0	0	0	0	0	2	1	5	3	1	72	1,75	23,51	0
287	0	0	0	0	0	2	1	5	3	1	72	1,75	23,51	0
288	1	0	0	0	0	3	1	3	2	0	60	1,65	22,04	0
289	1	0	0	0	0	3	1	3	2	0	60	1,65	22,04	0
290	1	0	0	0	0	1	1	4	2	1	79	1,67	28,33	0
291	1	0	0	0	0	1	1	4	2	1	79	1,67	28,33	0
292	1	0	1	0	0	1	0	2	0	1	60	1,62	22,86	0
293	1	0	1	0	0	1	0	2	0	1	60	1,62	22,86	0
294	1	0	0	0	1	2	1	2	1	0	70	1,68	24,8	0
295	1	0	0	0	1	2	1	2	1	0	70	1,68	24,8	0
296	2	0	0	0	0	3	1	2	1	0	69	1,64	25,65	0

297	2	0	0	0	0	3	1	2	1	0	69	1,64	25,65	0
298	0	0	0	1	0	1	0	1	0	0	48	1,63	18,07	0
299	0	0	0	1	0	1	0	1	0	0	48	1,63	18,07	0
300	0	0	0	0	0	2	0	1	0	0	85	1,75	27,76	0
301	0	0	0	0	0	2	0	1	0	0	85	1,75	27,76	0
302	2	0	0	0	0	1	0	4	0	3	64	1,65	23,51	0
303	2	0	0	0	0	1	0	4	0	3	64	1,65	23,51	0
304	1	0	0	0	0	1	0	1	0	0	57	1,67	20,44	0
305	1	0	0	0	0	1	0	1	0	0	57	1,67	20,44	0
306	2	0	0	0	0	1	1	2	1	0	66	1,74	21,8	0
307	2	0	0	0	0	1	1	2	1	0	66	1,74	21,8	0
308	0	0	0	0	1	1	1	3	1	1	57	1,67	20,44	0
309	2	0	0	0	0	1	0	1	0	0	59	1,69	20,66	0
310	2	0	0	0	0	1	0	1	0	0	59	1,69	20,66	0
311	1	0	0	0	0	3	1	4	2	1	60	1,59	23,73	0
312	1	0	0	0	0	3	1	4	2	1	60	1,59	23,73	0
313	1	0	0	0	0	3	1	3	1	1	70	1,62	26,67	0
314	1	0	0	0	0	3	1	3	1	1	70	1,62	26,67	0
315	0	0	0	0	0	3	1	10	6	3	57	1,65	20,94	0
316	0	0	0	0	0	3	1	10	6	3	57	1,65	20,94	0
317	1	0	1	0	0	3	0	1	0	0	74	1,67	26,53	0
318	1	0	1	0	0	3	0	1	0	0	74	1,67	26,53	0
319	1	0	1	0	0	1	1	5	3	1	121	1,64	44,99	0
320	1	0	1	0	0	1	1	5	3	1	121	1,64	44,99	0
321	1	0	1	0	0	3	1	5	1	3	57	1,7	19,72	0
322	0	0	0	0	0	1	0	1	0	0	58	1,55	24,14	0
323	0	0	0	0	0	1	0	1	0	0	58	1,55	24,14	0
324	0	0	0	0	0	1	0	2	0	1	81	1,7	28,03	0
325	0	0	0	0	0	1	0	2	0	1	81	1,7	28,03	0
326	0	0	0	0	0	3	1	2	1	0	70	1,62	26,67	0
327	0	0	0	0	0	1	0	1	0	0	60	1,68	21,26	0
328	0	0	0	0	0	1	0	1	0	0	60	1,68	21,26	0
329	0	0	0	0	0	1	0	1	0	1	55	1,68	19,49	0

330	0	0	0	0	0	3	1	5	3	1	54	1,56	22,19	0
331	0	0	0	0	0	3	1	5	3	1	54	1,56	22,19	0
332	0	0	0	1	0	1	0	1	0	0	54	1,64	20,08	0
333	0	0	0	1	0	1	0	1	0	0	54	1,64	20,08	0
334	2	0	0	0	0	1	0	2	0	1	86	1,65	31,59	0
335	2	0	0	0	0	1	0	2	0	1	86	1,65	31,59	0
336	1	0	0	0	1	1	1	2	1	0	66	1,67	23,67	0
337	1	0	0	0	1	1	1	2	1	0	66	1,67	23,67	0
338	2	0	0	0	0	1	0	2	0	1	116	1,76	37,45	0
339	1	0	0	0	1	1	1	4	2	1	63	1,72	21,3	0
340	1	0	0	0	1	1	1	4	2	1	63	1,72	21,3	0
341	2	0	0	0	0	3	1	3	2	0	65	1,64	24,17	0
342	2	0	0	0	0	3	1	3	2	0	65	1,64	24,17	0
343	2	0	0	0	0	1	1	3	1	1	58	1,71	19,84	0
344	2	0	0	0	0	1	1	3	1	1	58	1,71	19,84	0
345	2	0	0	0	1	1	1	2	1	0	54	1,65	19,83	0
346	2	0	0	0	1	1	1	2	1	0	54	1,65	19,83	0
347	0	0	0	0	0	1	0	1	0	0	67	1,71	22,91	0
348	0	0	0	0	0	1	0	1	0	0	67	1,71	22,91	0
349	2	0	0	0	1	3	1	2	1	0	66	1,6	25,78	0
350	2	0	0	0	1	3	1	2	1	0	66	1,6	25,78	0
351	2	0	0	0	1	2	0	1	0	0	63	1,7	21,8	0
352	2	0	0	0	1	2	0	1	0	0	63	1,7	21,8	0
353	1	0	0	0	1	1	1	2	1	0	52	1,64	19,33	0
354	1	0	0	0	1	1	1	2	1	0	52	1,64	19,33	0
355	0	0	0	0	1	1	0	1	0	0	56	1,56	23,01	0
356	0	0	0	0	1	1	0	1	0	0	56	1,56	23,01	0
357	0	0	0	0	0	2	0	1	0	0	80	1,73	26,73	0
358	1	0	0	1	0	3	0	1	0	0	51	1,65	18,73	0
359	2	1	0	0	0	2	0	1	0	0	78	1,58	31,24	0
360	1	0	0	0	1	3	1	5	1	3	58	1,62	22,10	0
361	1	0	0	0	1	3	1	3	2	0	56	1,6	21,88	0
362	1	0	0	1	1	1	0	2	0	1	81,5	1,78	25,72	0

363	2	0	0	0	0	1	0	1	0	0	110	1,73	36,75	0
364	1	0	0	0	0	1	0	1	0	0	58	1,67	20,80	0
365	1	0	0	0	0	1	0	1	0	0	59	1,65	21,67	0
366	1	0	0	0	0	1	0	1	0	0	63	1,61	24,30	0
367	1	0	0	0	0	1	1	2	1	0	88,8	1,8	27,41	0
368	0	0	0	0	0	1	0	1	0	0	72	1,65	26,45	0
369	2	0	1	0	0	1	0	1	0	0	55	1,76	17,76	0
370	1	0	0	0	0	2	1	3	2	0	65	1,63	24,46	0
371	2	1	1	1	1		1	3	1	1	80	1,68	28,34	0
372	1	0	0	0	0	1	0	1	0	0	69	1,59	27,29	0
373	0	0	0	0	1	3	0	2	0	1	45	1,62	17,15	0
374	2	0	0	0	0	1	0	1	0	0	70	1,74	23,12	0
375	1	0	0	0	1	3	1	2	1	0	58	1,58	23,23	0
376	1	0	1	0	0	2	0	1	0	0	57	1,7	19,72	0
377	1	0	0	0	0		0	1	0	0	65	1,7	22,49	0
378	1	0	0	0	0	1	1	2	1	0	64	1,71	21,89	0
379	2	0	1	0	0	1	0	1	0	0	66,8	1,57	27,10	0
380	0	0	1	0	0	1	0	1	0	0	56	1,69	19,61	0
381	2	0	1	0	0	6	0	1	0	0	79	1,75	25,80	0
382	1	0	1	0	0	3	1	3	2	0	118	1,8	36,42	0
383	1	0	0	0	0	2	0	1	0	0	61	1,65	22,41	0
384	0	0	0	0	0	1	0	1	0	0	68	1,68	24,09	0
385	0	1	0	0	1	1	0	1	0	0	49	1,68	17,36	0
386	0	1	0	0	1	1	0	1	0	0	49	1,68	17,36	0
387	0	0	0	0	1	1	1	1	1	0	64	1,64	23,8	0
388	0	0	0	0	1	1	1	1	1	0	64	1,64	23,8	0
389	1	0	0	0	0	1	1	5	2	2	63	1,72	21,3	0
390	1	0	0	0	0	1	1	5	2	2	63	1,72	21,3	0
391	2	0	0	0	0	1	1	3	1	1	68	1,65	24,98	0
392	2	0	0	0	0	1	1	3	1	1	68	1,65	24,98	0
393	1	0	0	1	0	4	1	4	1	2	56	1,56	23,01	0
394	1	0	0	1	0	4	1	4	1	2	56	1,56	23,01	0
395	2	0	1	0	0	1	1	3	1	1	60	1,68	21,26	0

396	2	0	1	0	0	1	1	3	1	1	60	1,68	21,26	0
397	2	0	0	0	0	3	1	5	3	1	77	1,55	32,05	0
398	2	0	0	0	0	3	1	5	3	1	77	1,55	32,05	0
399	0	1	0	0	0	1	1	2	1	0	62	1,64	23,05	0
400	0	1	0	0	0	1	1	2	1	0	62	1,64	23,05	0
401	2	0	0	0	0	3	1	4	3	0	94	1,63	35,38	0
402	2	0	0	0	0	1	1	4	2	1	80	1,71	27,36	0
403	2	0	0	0	0	1	1	4	2	1	80	1,71	27,36	0
404	1	0	0	0	0	1	1	2	1	0	66	1,6	25,78	0
405	1	1	1	0	0	3	0	1	0	0	75	1,47	34,71	0
406	1	0	0	0	0	1	1	2	1	0		1,63		0
407	1	0	0	0	0	3	0	1	0	0	55	1,7	19,03	0
408	1	0	0	0	0	1	1	3	1	1	80	1,71	27,36	0
409	1	0	0	0	1	1	0	1	0	0	75	1,67	26,89	0
410	2	0	1	0	0	1	1	2	1	0	57	1,58	22,83	0
411	1	0	1	0	0	3	1	3	1	1	64	1,63	24,09	0
412	0	0	1	0	0	1	1	3	2	0	68,5	1,74	22,63	0
413	1	1	0	0	0	1	1	2	1	0	50,4	1,65	18,51	0
414	0	0	0	0	0	1	0	1	0	0	53	1,56	21,78	0
415	0	0	0	0	1	1	1	2	1	0	54	1,62	20,58	0
416	2	0	0	0	0		0	1	0	0	58	1,63	21,83	0
417	0	0	0	0	0		0	1	0	0	77	1,55	32,05	0
418	0	0	0	0	0	2	0	1	0	0	50	1,55	20,81	0
419	0	0	0	0	0	2	0	1	0	0	50	1,55	20,81	0
420	0	0	0	0	0	1	0	2	0	1	54	1,58	21,63	0
421	0	0	0	0	0	3	0	1	0	0	58	1,61	22,38	0
422	0	0	0	0	0	3	0	1	0	0	58	1,61	22,38	0
423	0	0	0	0	0	2	0	1	0	0	75	1,78	23,67	0
424	1	0	0	0	0	2	0	1	0	0	60	1,62	22,86	0
425	1	0	0	0	0	2	0	1	0	0	60	1,62	22,86	0
426	2	1	1	0	1	1	0	1	0	0	65	1,63	24,46	0
427	1	0	1	0	0	1	0	1	0	0	62	1,65	22,77	0
428	1	0	0	0	0	1	1	3	1	1	48	1,6	18,75	0

429	1	0	0	0	0	1	1	3	1	1	65,7	1,69	23,00	0
430	2	0	0	0	1	1	0	1	0	0	78	1,62	29,72	0
431	2	0	0	0	1	1	0	1	0	0	78	1,62	29,72	0
432	2	0	0	0	1	1	0	1	0	0	61	1,7	21,11	0
433	2	1	0	0	1	2	0	1	0	0	64	1,69	22,41	0
434	2	1	0	0	1	2	0	1	0	0	64	1,69	22,41	0
435	1	0	0	1	0	1	0	1	0	0	76	1,7	26,30	0
436	1	0	0	0	0	3	0	1	0	0	52	1,57	21,10	0
437	1	0	0	0	1	1	0	2	0	1	60,4	1,53	25,80	0
438	1	0	1	0	0	6	1	4	2	1	80	1,59	31,64	0
439	1	0	1	0	0	6	1	4	2	1	80	1,59	31,64	0
440	0	0	0	0	0	2	1	2	1	0	113	1,63	42,53	0
441	0	0	0	0	0	2	1	2	1	0	113	1,63	42,53	0
442	1	0	0	0	0	1	0	2	0	1	62	1,77	19,79	0
443	1	0	0	0	0	1	0	2	0	1	62	1,77	19,79	0
444	1	0	1	0	0	1	0	2	0	1	63	1,58	25,24	0
445	1	0	1	0	0	1	0	2	0	1	63	1,58	25,24	0
446	1	0	0	0	0	1	0	2	0	1	113	1,7	39,1	0
447	1	0	0	0	0	1	0	2	0	1	113	1,7	39,1	0
448	1	0	1	0	0	1	1	2	1	0	77	1,66	27,94	0
449	1	1	1	0	0	2	1	2	1	0	74	1,64	27,51	0
450	0	0	0	0	0	1	1	3	1	1	56	1,64	20,82	0
451	0	0	0	0	0	1	1	3	1	1	56	1,64	20,82	0
452	0	0	0	0	0	1	0	1	0	0	42	1,53	17,94	0
453	0	0	0	0	0	2	1	2	1	0	62	1,6	24,22	0
454	0	0	0	0	0	2	1	2	1	0	62	1,6	24,22	0
455	0	0	0	0	0	2	0	1	0	0	73	1,61	28,16	0
456	0	0	0	0	0	2	0	1	0	0	73	1,61	28,16	0
457	1	0	0	0	1	1	0	3	0	2	87	1,73	29,07	0
458	2	0	0	0	0	1	0	1	0	0	60	1,73	20,05	0
459	1	0	1	0	1	1	0	2	0	1	58	1,69	20,31	0
460	1	0	1	0	1	1	0	2	0	1	58	1,69	20,31	0
461	0	0	0	0	0	2	1	4	3	0	55	1,6	21,48	0

462	0	0	0	0	0	2	1	4	3	0	55	1,6	21,48	0
463	2	0	0	0	0	3	0	1	0	0	50	1,6	19,53	0
464	2	0	0	0	0	3	0	1	0	0	50	1,6	19,53	0
465	1	0	0	0	0	1	1	3	1	1	82	1,77	26,17	0
466	1	0	0	0	0	1	1	3	1	1	82	1,77	26,17	0
467	2	0	1	0	0	1	0	2	0	1	96	1,75	31,35	0
468	2	0	0	0	0	1	0	1	0	0	68	1,68	24,09	0
469	2	0	0	0	0	1	0	1	0	0	68	1,68	24,09	0
470	0	0	0	0	0		1	4	3	0	69	1,71	23,6	0
471	0	0	0	0	0		1	4	3	0	69	1,71	23,6	0
472	0	0	0	0	0		0	2	0	1	53	1,7	18,34	0
473	0	0	0	0	0		0	2	0	1	53	1,7	18,34	0
474	0	0	0	0	0	6	1	3	2	0	48	1,63	18,07	0
475	0	0	0	0	0	6	1	3	2	0	48	1,63	18,07	0
476	0	0	0	0	0	1	0	1	0	0	50	1,6	19,53	0
477	0	0	0	0	0	3	0	2	0	1	68	1,54	28,67	0
478	0	0	0	0	0	3	0	2	0	1	68	1,54	28,67	0
479	0	0	0	0	0	1	0	1	0	0	50	1,6	19,53	0
480	0	0	1	0	0	3	1	2	1	0	62	1,62	23,62	0
481	0	0	0	0	0	3	1	3	2	0	49	1,6	19,14	0
482	0	0	0	0	0	3	1	2	1	0	54	1,71	18,47	0
483	0	0	0	0	0	3	1	3	2	0	49	1,6	19,14	0
484	0	0	0	0	0	1	0	1	0	0	64	1,58	25,64	0
485	0	0	0	0	0	1	0	1	0	0	54	1,63	20,32	0
486	0	0	1	0	0	2	1	2	1	0	58	1,59	22,94	0
487	0	0	0	0	0	1	0	1	0	0	54	1,63	20,32	0
488	0	0	0	0	0	1	0	1	0	0	46	1,6	17,97	0
489	0	0	0	0	0	1	0	1	0	0	46	1,6	17,97	0
490	0	0	0	0	0	2	0	2	0	1	70	1,65	25,71	0
491	0	0	0	0	0	2	0	2	0	1	70	1,65	25,71	0
492	0	0	1	0	0	1	1	5	4	1	66	1,7	22,84	0
493	0	0	1	0	0	1	1	5	4	1	66	1,7	22,84	0
494	0	1	0	0	0	1	1	2	1	0	52	1,58	20,83	0

495	0	0	0	0	0	1	1	3	1	1	66	1,63	24,84	0
496	0	0	0	0	0	3	1	2	1	0	57	1,67	20,44	0
497	0	0	1	0	0	1	0	1	0	0	60	1,65	22,04	0
498	0	1	0	0	0	1	1	2	1	0	52	1,58	20,83	0
499	0	0	0	0	0	3	1	2	1	0	57	1,67	20,44	0
500	0	0	0	0	0	1	1	3	1	1	66	1,63	24,84	0
501	0	0	1	0	0	1	0	1	0	0	60	1,65	22,04	0
502	0	0	0	0	0	1	0	1	0	0	53	1,59	20,96	0
503	0	0	0	0	0	1	0	2	0	1	64	1,64	23,8	0
504	0	0	0	0	0	1	0	2	0	1	64	1,64	23,8	0
505	0	0	0	0	0	1	0	1	0	0	53	1,59	20,96	0
506	0	0	0	0	0	1	0	1	0	0	58	1,6	22,66	0
507	0	0	0	0	0	1	0	1	0	0	58	1,6	22,66	0
508	0	0	0	0	0	2	0	1	0	0	60	1,68	21,26	0
509	0	0	0	0	0	2	0	1	0	0	60	1,68	21,26	0
510	0	0	0	0	0	1	0	2	0	1	57	1,69	19,96	0
511	0	0	0	0	1	1	0	1	0	0	68	1,76	21,95	0
512	0	0	0	0	1	1	0	1	0	0	68	1,76	21,95	0
513	0	0	0	0	0	2	1	3	1	1	74	1,61	28,55	0
514	0	0	0	0	0	2	1	3	1	1	74	1,61	28,55	0
515	0	0	0	0	0	1	1	3	1	1	74	1,67	26,53	0
516	0	0	0	0	0	1	1	3	1	1	74	1,67	26,53	0
517	0	0	0	0	0	1	1	2	1	0	52	1,56	21,37	0
518	0	0	0	0	0	2	0	1	0	0	67	1,67	24,02	0
519	0	0	0	0	0	2	1	2	1	0	70	1,68	24,8	0
520	0	0	0	0	0	2	1	2	1	0	70	1,68	24,8	0
521	0	0	0	0	0	2	1	3	2	0	57	1,59	22,55	0
522	0	0	0	0	0	2	1	3	2	0	57	1,59	22,55	0
523	0	0	0	0	0	1	1	2	1	0	58	1,6	22,66	0
524	0	0	0	0	0	1	1	2	1	0	58	1,6	22,66	0
525	0	0	0	0	0	2	1	4	3	0	57	1,6	22,27	0
526	0	0	0	0	0	1	1	2	1	0	63	1,73	21,05	0
527	0	0	0	0	0	1	0	1	0	0	94	1,59	37,18	0

528	0	0	0	0	0	2	1	4	3	0	57	1,6	22,27	0
529	0	0	0	0	0	1	1	2	1	0	63	1,73	21,05	0
530	0	0	0	0	1	1	0	2	0	1	57	1,63	21,45	0
531	0	0	0	0	0	6	0	1	0	0	53	1,62	20,2	0
532	0	0	0	0	1	1	0	2	0	1	57	1,63	21,45	0
533	0	0	0	0	0	6	0	1	0	0	53	1,62	20,2	0
534	0	0	0	0	1	1	0	1	0	0	60	1,76	19,37	0
535	0	0	0	0	0	1	1	2	1	0	84	1,68	29,76	0
536	0	0	0	0	0	1	1	2	1	0	84	1,68	29,76	0
537	0	0	0	0	0	1	0	1	0	0		1,6		0
538	0	0	0	0	0	1	0	2	0	1	60	1,68	21,26	0
539	0	0	0	0	0	1	1	3	2	0	56	1,6	21,88	0
540	0	0	0	0	0	1	0	2	0	1	60	1,68	21,26	0
541	0	0	0	0	0	1	0	2	0	1	58,8	1,72	19,88	0
542	0	0	0	0	0	1	0	2	0	1	49	1,55	20,40	0
543	0	0	0	0	0	1	1	2	1	0	56	1,65	20,57	0
544	0	0	0	0	0	1	1	2	1	0	56	1,65	20,57	0
545	0	0	0	0	0	2	1	3	2	0	50	1,55	20,81	0
546	0	0	0	0	0	2	1	3	2	0	50	1,55	20,81	0
547	0	0	0	0	0	1	0	1	0	0	51	1,65	18,73	0
548	0	0	0	0	1	1	1	2	1	0	74	1,65	27,18	0
549	0	0	0	0	0	4	1	6	4	1	58	1,58	23,23	0
550	0	0	0	0	0	1	0	1	0	0	60	1,7	20,76	0
551	0	0	0	0	0	4	0	3	0	2	44	1,58	17,63	0
552	0	0	0	0	0	1	0	1	0	0	60	1,7	20,76	0
553	0	0	0	0	0	2	1	2	1	0	87	1,7	30,1	0
554	0	0	0	0	0	2	1	2	1	0	87	1,7	30,1	0
555	0	0	0	0	0	1	1	3	1	1	63	1,67	22,59	0
556	0	0	0	0	0	1	1	3	1	1	63	1,67	22,59	0
557	0	0	0	0	0	1	0	1	0	0	75,2	1,75	24,56	0
558	0	0	0	0	1	1	1	3	1	1	64	1,75	20,9	0
559	0	0	0	0	0	1	1	2	1	0	56	1,68	19,84	0
560	0	0	0	0	0	1	1	2	1	0	56	1,68	19,84	0

561	0	0	0	0	0	1	0	1	0	0	64	1,6	25	0
562	0	0	0	0	0	1	0	1	0	0	64	1,6	25	0
563	0	1	0	0	0	3	1	2	1	0	93	1,68	32,95	0
564	0	1	0	0	0	3	1	2	1	0	93	1,68	32,95	0
565	0	0	0	0	0	3	1	3	2	0	53	1,68	18,78	0
566	0	0	0	0	0	2	1	2	1	0	70	1,65	25,71	0
567	0	0	0	0	0	3	1	3	2	0	53	1,68	18,78	0
568	0	0	0	0	0	2	1	2	1	0	70	1,65	25,71	0
569	0	0	0	0	0	1	1	2	1	0	68	1,6	26,56	0
570	0	0	0	0	0	1	0	1	0	0	69	1,63	25,97	0
571	0	0	0	0	0	1	0	1	0	0	64,5	1,75	21,06	0
572	0	0	0	0	0	1	0	1	0	0	69	1,63	25,97	0
573	0	0	0	0	0	4	0	2	0	1	60	1,6	23,44	0
574	0	0	0	0	0	1	1	5	1	3	46	1,62	17,53	0
575	0	0	0	0	0	3	1	3	1	1	69	1,53	29,48	0
576	0	0	0	0	0	1	1	5	1	3	46	1,62	17,53	0
577	0	0	0	0	0	2	1	4	2	1	49	1,51	21,49	0
578	0	0	0	0	0	3	1	3	1	1	69	1,53	29,48	0
579	0	0	0	0	0	1	1	3	1	1	57	1,59	22,55	0
580	0	0	0	0	0	1	1	3	1	1	57	1,59	22,55	0
581	0	0	0	0	0	2	1	4	2	1	49	1,51	21,49	0
582	0	0	0	0	1	1	1	3	2	0	51	1,61	19,68	0
583	0	0	0	0	1	1	1	3	2	0	51	1,61	19,68	0
584	0	0	0	0	0	1	0	1	0	0	80	1,65	29,38	0
585	0	0	0	0	0	1	0	1	0	0	80	1,65	29,38	0
586	0	0	0	0	0	1	0	1	0	0	80	1,65	29,38	0
587	0	0	0	0	1	1	0	1	0	0	56	1,66	20,32	0
588	0	0	0	0	1	1	0	1	0	0	56	1,66	20,32	0
589	0	0	0	0	0	1	1	3	1	1	47	1,58	18,83	0
590	0	0	0	0	0	1	1	2	1	0	61	1,67	21,87	0
591	0	0	0	0	0	1	1	2	1	0	61	1,67	21,87	0
592	0	0	0	0	0	1	0	1	0	0	65	1,68	23,03	0
593	0	0	0	0	1	1	1	2	1	0	71	1,71	24,28	0

594	0	0	0	0	0	1	1	3	1	1	69	1,65	25,34	0
595	0	0	0	0	0	1	1	3	1	1	69	1,65	25,34	0
596	0	0	0	0	0	1	1	2	1	0	80	1,81	24,42	0
597	0	0	0	0	0	1	1	3	1	1	47	1,58	18,83	0
598	0	0	0	0	0	1	0	1	0	0	65	1,68	23,03	0
599	0	0	0	0	0	2	1	2	1	0	63	1,74	20,81	0
600	0	0	0	0	1	1	1	2	1	0	71	1,71	24,28	0
601	0	0	0	0	0	1	1	2	1	0	115	1,69	40,26	0
602	0	0	0	0	0	2	1	2	1	0	63	1,74	20,81	0
603	0	0	0	0	0	1	1	2	1	0	115	1,69	40,26	0
604	0	0	0	0	0	6	1	5	3	1	60	1,66	21,77	0
605	0	0	1	0	0	2	0	1	0	0	56	1,68	19,84	0
606	0	0	0	0	0	1	0	1	0	0	69	1,7	23,88	0
607	0	0	0	0	0	6	1	5	3	1	60	1,66	21,77	0
608	0	0	1	0	0	2	0	1	0	0	56	1,68	19,84	0
609	0	0	0	0	0	1	0	1	0	0		1,69		0
610	0	0	0	0	0	1	0	1	0	0	69	1,7	23,88	0
611	0	0	0	0	0	1	0	1	0	0	76	1,69	26,61	0
612	0	0	0	0	0	1	0	1	0	0	76	1,69	26,61	0
613	0	0	0	0	1	1	0	1	0	0	71	1,7	24,57	0
614	0	0	0	0	1	1	0	1	0	0	71	1,7	24,57	0
615	0	0	0	0	0	1	0	1	0	0	86	1,7	29,76	0
616	0	0	0	0	0	1	1	3	1	1	92	1,75	30,04	0
617	0	0	0	0	0	1	1	3	2	0	62	1,72	20,96	0
618	0	0	0	0	0	1	1	3	2	0	62	1,72	20,96	0
619	0	0	0	0	0	1	0	1	0	0	86	1,7	29,76	0
620	0	0	0	0	0	6	0	1	0	0	57	1,65	20,94	0
621	0	0	0	0	0	6	1	3	2	0	80	1,6	31,25	0
622	0	0	0	0	1	1	1	3	2	0	72	1,69	25,21	0
623	0	0	0	0	0	6	1	3	2	0	80	1,6	31,25	0
624	0	1	0	0	0	1	1	2	1	0	88	1,58	35,25	0
625	0	1	0	0	0	1	1	2	1	0	88	1,58	35,25	0
626	0	0	0	0	0	1	1	2	1	0	85	1,74	28,08	0

627	0	0	0	0	1	1	1	3	2	0	72	1,69	25,21	0
628	0	0	0	0	0	1	1	5	1	3	106	1,74	35,01	0
629	0	0	0	0	0	1	0	1	0	0	66	1,61	25,46	0
630	0	0	0	0	0	1	0	1	0	0	78	1,76	25,18	0
631	0	0	0	0	0	1	0	1	0	0	66	1,61	25,46	0
632	0	0	0	0	0	1	1	5	1	3	106	1,74	35,01	0
633	0	0	0	0	0	1	1	5	1	3	106	1,74	35,01	0
634	0	0	0	0	0	2	0	1	0	0	72	1,7	24,91	0
635	0	0	0	0	0	5	0	1	0	0	68	1,5	30,22	0
636	0	0	0	0	0	2	0	1	0	0	72	1,7	24,91	0
637	0	1	1	0	0	3	1	3	2	0	89	1,67	31,91	0
638	0	1	1	0	0	3	1	3	2	0	89	1,67	31,91	0
639	0	0	0	0	0	1	1	3	1	1	102	1,78	32,19	0
640	0	0	1	0	1	3	0	1	0	0	66	1,58	26,44	0
641	0	0	0	0	0	1	1	3	1	1	102	1,78	32,19	0
642	0	0	1	0	1	3	0	1	0	0	66	1,58	26,44	0
643	0	0	0	0	0	1	0	1	0	0	62	1,81	18,92	0
644	2	1	0	0	0	1	0	1	0	0	72	1,61	27,78	0
645	2	1	0	0	0	1	0	1	0	0	72	1,61	27,78	0
646	2	0	0	0	0	1	0	1	0	0	72	1,6	28,12	0
647	2	0	0	0	0	1	0	1	0	0	72	1,6	28,12	0
648	1	0	0	0	1	1	1	2	1	0	72	1,68	25,51	0
649	1	0	0	0	1	1	1	2	1	0	72	1,68	25,51	0
650	1	0	0	0	0	1	1	2	1	0	64	1,69	22,41	0
651	1	0	1	0	0	6	1	2	1	0	67	1,6	26,17	0
652	1	0	0	0	0	1	0	1	0	0	70	1,69	24,51	0
653	1	0	0	0	0	1	0	1	0	0	70	1,69	24,51	0
654	1	0	0	0	0	1	0	1	0	0	49	1,63	18,44	0
655	1	0	0	0	0	1	0	1	0	0	49	1,63	18,44	0
656	1	0	0	0	0	1	0	1	0	0	65	1,7	22,49	0
657	1	0	0	0	0	2	1	2	1	0	54	1,56	22,19	0
658	2	0	0	0	0	3	1	3	1	1	49	1,63	18,44	0
659	2	0	0	0	0	3	1	3	1	1	49	1,63	18,44	0

660	2	0	0	0	1	1	1	3	1	1	65,5	1,75	21,39	0
661	1	0	1	0	0	6	1	3	2	0	70	1,65	25,71	0
662	1	0	1	0	0	6	1	3	2	0	70	1,65	25,71	0
663	2	0	0	0	1	1	0	1	0	0	70	1,7	24,22	0
664	2	0	0	0	1	1	0	1	0	0	70	1,7	24,22	0
665	1	0	0	0	0	1	1	6	2	4	90	1,74	29,73	1
666	1	0	0	0	0	1	0	3	0	2	68	1,7	23,53	0
667	1	0	0	0	0	1	0	3	0	2	68	1,7	23,53	0
668	1	0	0	0	0	1	0	4	0	3	64	1,75	20,9	0
669	1	0	0	0	0	1	0	4	0	3	64	1,75	20,9	0
670	1	0	1	0	0	1	0	1	0	0	72,5	1,73	24,22	0
671	1	0	0	0	1	3	0	1	0	0	58	1,69	20,31	0
672	1	0	0	0	1	3	0	1	0	0	58	1,69	20,31	0
673	2	0	1	0	0	1	1	2	1	0	83	1,61	32,02	0
674	1	0	0	0	0	3	0	1	0	1	60	1,7	20,76	0
675	1	0	0	0	0	3	0	1	0	1	60	1,7	20,76	0
676	1	0	0	0	0	1	0	1	0	0	58	1,72	19,61	0
677	2	0	1	0	1	1	0	1	0	0	59	1,68	20,9	0
678	2	1	0	0	0	1	1	4	1	2	80	1,65	29,38	0
679	2	1	0	0	0	1	1	4	1	2	80	1,65	29,38	0
680	2	0	0	0	0	1	1	3	2	0	62	1,69	21,71	0
681	1	0	0	0	0	3	1	2	1	0	83	1,62	31,63	0
682	1	0	0	0	0	3	1	3	2	0	77	1,65	28,28	0
683	2	1	0	0	1	1	0	1	0	0	65	1,62	24,77	0
684	2	1	0	0	1	1	0	1	0	0	65	1,62	24,77	0
685	1	0	0	0	0	1	1	3	1	1	85	1,62	32,39	0
686	1	0	0	0	0	1	1	3	1	1	85	1,62	32,39	0
687	1	0	0	0	0	3	0	2	0	1	60	1,72	20,28	0
688	1	0	0	0	0	3	0	2	0	1	60	1,72	20,28	0
689	2	0	1	0	0	3	1	3	2	0	51	1,66	18,51	0
690	2	0	0	0	0	1	0	1	0	0	55	1,6	21,48	0
691	2	0	0	0	0	1	0	1	0	0	55	1,6	21,48	0
692	1	0	0	0	1	2	0	3	0	2	63	1,72	21,3	0

693	1	0	0	0	1	2	0	3	0	2	63	1,72	21,3	0
694	1	0	0	0	1	2	0	3	0	2	63	1,72	21,3	0
695	1	0	0	0	0	2	1	2	1	0	56	1,57	22,72	0
696	1	0	1	0	0		0	4	0	3	52	1,59	20,57	0
697	1	0	0	0	0	3	1	5	4	0	65	1,67	23,31	0
698	2	1	0	0	0	1	0	1	0	0	52	1,71	17,78	0
699	2	1	0	0	0	1	0	1	0	0	52	1,71	17,78	0
700	2	0	0	0	0	1	1	2	1	0	55	1,66	19,96	0
701	2	0	0	0	0	1	1	2	1	0	55	1,66	19,96	0
702	2	0	0	0	0	1	0	1	0	0	64	1,57	25,96	0
703	1	1	0	0	0	1	0	1	0	0	63	1,64	23,42	0
704	1	0	0	0	0	1	0	1	0	0	73	1,76	23,57	0
705	1	0	0	0	1	1	1	2	1	0	92	1,76	29,70	0
706	1	1	0	0	0	1	0	1	0	0	89	1,65	32,69	0
707	1	0	0	0	0	3	1	4	3	0	100	1,59	39,56	0
708	1	0	0	0	1	5	1	2	1	0	62	1,67	22,23	0
709	1	0	1	0	0	1	0	1	0	0	63	1,64	23,42	0
710	1	0	0	0	0	1	1	2	1	0	52	1,73	17,37	0
711	1	1	1	0	0	1	0	1	0	0	60,5	1,67	21,69	0
712	1	0	0	0	0	1	0	1	0	0	56	1,63	21,08	0
713	1	0	1	0	0	3	1	3	2	0	57	1,63	21,45	0
714	1	0	0	0	0	1	1	2	1	0	56	1,7	19,38	0
715	1	0	0	0	0	1	1	2	1	0	62	1,67	22,23	0
716	1	0	0	0	0	2	0	1	0	0	64	1,62	24,39	0
717	1	0	0	0	0	5	1	4	3	0	71	1,61	27,39	0
718	1	0	0	0	0	5	1	4	3	0	71	1,61	27,39	0
719	1	0	0	0	0	3	0	1	0	0	47,5	1,53	20,29	0
720	1	0	0	0	1	1	1	2	1	0	82	1,76	26,47	0
721	1	0	0	0	0	3	0	1	0	0	62	1,62	23,62	0
722	1	0	0	0	0	2	0	1	0	0	60	1,68	21,26	0
723	1	0	0	0	0	1	1	4	1	2	75	1,65	27,55	0
724	1	1	0	0	0	1	1	2	1	0	73,5	1,68	26,04	0
725	1	1	0	0	0	6	1	2	1	0	61	1,58	24,44	0

726	1	0	0	0	0	2	0	1	0	0	48	1,55	19,98	0
727	1	0	0	0	0	1	0	1	0	0	63,8	1,75	20,83	0
728	1	0	0	0	0	1	0	1	0	0	53	1,65	19,47	0
729	1	0	0	0	0	1	0	1	0	0	69,5	1,68	24,62	0
730	1	0	0	0	0	1	1	2	1	0	69	1,69	24,16	0
731	1	0	0	0	0	2	1	2	1	0	78	1,7	26,99	0
732	1	0	0	0	0	1	1	4	2	1	62	1,61	23,92	0
733	2	0	0	0	0	1	1	2	1	0	56	1,57	22,72	0
734	2	0	0	0	0	1	1	2	1	0	56	1,57	22,72	0
735	2	0	0	0	1	1	1	3	2	0	71	1,58	28,44	0
736	2	0	0	0	1	1	1	3	2	0	71	1,58	28,44	0
737	1	0	0	0	0	1	0	1	0	0	50,5	1,63	19,01	0
738	2	0	0	0	0	1	1	2	1	0	53	1,67	19	0
739	2	0	0	0	0	1	0	1	0	0	57	1,61	21,99	0
740	2	0	0	0	0	1	0	1	0	0	57	1,61	21,99	0
741	1	0	0	0	1	1	0	1	0	0	51	1,66	18,51	0
742	1	0	0	0	1	1	0	1	0	0	75	1,68	26,57	0
743	1	0	0	0	1	1	0	1	0	0	75	1,68	26,57	0
744	1	0	0	0	0	1	0	1	0	0	81,5	1,79	25,44	0
745	2	1	0	0	0	1	0	1	0	0	102	1,67	36,57	0
746	1	0	0	0	0	1	1	2	1	0	66	1,57	26,78	0
747	1	0	0	0	0	1	1	4	2	1	75	1,63	28,23	0
748	1	0	0	0	0	1	1	4	2	1	75	1,63	28,23	0
749	1	0	0	0	0	2	0	1	0	0	54	1,58	21,63	0
750	1	0	0	0	0	2	0	1	0	0	54	1,58	21,63	0
751	1	0	0	0	0	1	1	6	3	2	58	1,6	22,66	1
752	1	0	0	0	0	6	1	2	1	0	52	1,6	20,31	0
753	1	0	1	0	0	2	1	2	1	0	71	1,6	27,73	0
754	1	0	0	0	0	1	1	5	1	3	71,5	1,83	21,35	0
755	1	1	0	0	1	1	0	1	0	0	88	1,65	32,32	0
756	1	0	0	0	0	6	1	4	2	1	60	1,65	22,04	0
757	1	0	0	0	0	6	1	4	2	1	60	1,65	22,04	0
758	1	0	0	0	0	1	1	2	1	0	65	1,68	23,03	0

759	1	0	0	0	0	1	1	2	1	0	65	1,68	23,03	0
760	2	0	0	0	0	1	0	1	0	0	78	1,8	24,07	0
761	2	0	0	0	0	1	0	1	0	0	78	1,8	24,07	0
762	2	0	0	0	1	1	0	1	0	0	73	1,63	27,48	0
763	2	0	0	0	1	1	0	1	0	0	73	1,63	27,48	0
764	2	0	0	0	1	1	1	2	1	0	65	1,68	23,03	0
765	2	0	0	0	1	1	1	2	1	0	65	1,68	23,03	0
766	2	1	1	0	0	1	0	2	0	1	83	1,67	29,76	0
767	2	1	1	0	0	1	0	2	0	1	83	1,67	29,76	0
768	2	1	0	0	1	1	0	1	0	0	116	1,67	41,59	0
769	2	1	0	0	1	1	0	1	0	0	116	1,67	41,59	0
770	2	1	0	0	0	1	0	1	0	0	58	1,69	20,31	0
771	2	1	0	0	0	1	0	1	0	0	58	1,69	20,31	0
772	1	0	0	0	1	1	0	1	0	0	54	1,57	21,91	0
773	1	0	0	0	1	1	0	1	0	0	54	1,57	21,91	0
774	1	0	0	0	1	1	1	2	1	1	53	1,59	20,96	0
775	1	0	0	0	1	1	1	2	1	1	53	1,59	20,96	0
776	2	0	1	0	1		0	1	0	0	58	1,58	23,23	0
777	2	0	0	0	0	3	0	1	0	0	75	1,6	29,30	0
778	2	0	0	0	0	1	0	2	0	1	50	1,59	19,78	0
779	2	1	0	0	1	1	1	2	1	0	98	1,69	34,31	0
780	2	1	0	0	1	1	1	2	1	0	98	1,69	34,31	0
781	2	0	0	0	1	1	0	1	0	0	70	1,67	25,1	0
782	2	0	0	0	1	1	0	1	0	0	70	1,67	25,1	0
783	2	0	0	0	1	1	0	1	0	0	58	1,67	20,8	0
784	2	0	0	0	0	3	1	2	1	0	57	1,6	22,27	0
785	2	0	0	0	1	1	0	1	0	0	58	1,67	20,8	0
786	2	0	0	0	0	1	1	2	1	0	69	1,5	30,67	0
787	2	0	0	0	0	1	1	2	1	0	69	1,5	30,67	0
788	2	0	0	0	0	1	0	1	0	0	106	1,7	36,68	0
789	2	0	0	0	0	1	0	1	0	0	60	1,68	21,26	0
790	2	0	0	0	0	1	0	1	0	0	60	1,68	21,26	0
791	2	0	0	0	0	1	0	1	0	0	64	1,7	22,15	0

792	2	0	0	0	0	1	0	1	0	0	64	1,7	22,15	0
793	2	0	0	0	0	1	1	2	1	0	71,5	1,66	25,95	0
794	2	0	0	0	1	1	0	1	0	0	56	1,69	19,61	0
795	2	0	0	0	1	1	0	1	0	0	56	1,69	19,61	0
796	2	1	1	0	0	1	0	1	0	0	50	1,58	20,03	0
797	2	1	1	0	0	1	0	1	0	0	50	1,58	20,03	0
798	2	0	0	0	0	2	0	1	0	0	80	1,71	27,36	0
799	2	0	0	0	0	2	0	1	0	0	80	1,71	27,36	0
800	2	0	0	0	0	1	1	3	1	1	70	1,7	24,22	0
801	2	0	0	0	0	1	1	3	1	1	70	1,7	24,22	0
802	1	0	0	0	0	1	1	4	1	2	56	1,67	20,08	0
803	1	0	0	0	0	1	1	4	1	2	56	1,67	20,08	0
804	1	0	0	0	0	1	1	3	1	1	61	1,6	23,83	0
805	1	0	0	0	0	1	1	3	1	1	61	1,6	23,83	0
806	2	0	0	0	0	1	1	4	2	1	89	1,62	33,91	0
807	2	0	0	0	0	1	1	4	2	1	89	1,62	33,91	0
808	2	0	0	0	0	1	0	1	0	0	64	1,65	23,51	0
809	1	0	1	0	0	2	0	1	0	0	54	1,6	21,09	0
810	1	0	1	0	0	2	0	1	0	0	54	1,6	21,09	0
811	1	1	0	0	0	1	0	2	0	1	56	1,68	19,84	0
812	1	1	0	0	0	1	0	2	0	1	56	1,68	19,84	0
813	1	0	0	0	0	1	0	1	0	0	77	1,66	27,94	0
814	2	0	0	0	0	3	1	4	1	2	74	1,7	25,61	0
815	2	0	0	0	0	3	1	4	1	2	74	1,7	25,61	0
816	2	0	0	0	0	1	0	1	0	0	49	1,65	18	0
817	2	0	0	0	1	1	0	1	0	0	60	1,62	22,86	0
818	2	0	0	0	1	1	0	1	0	0	60	1,62	22,86	0
819	1	0	0	0	1	2	0	1	0	0	50	1,6	19,53	0
820	2	0	0	0	0	1	1	2	1	0	60	1,64	22,31	0
821	2	0	0	0	0	1	1	2	1	0	60	1,64	22,31	0
822	1	0	0	0	1	1	0	3	0	2	72	1,64	26,77	0
823	1	0	1	0	0	2	0	2	0	1	66	1,6	25,78	0
824	0	0	0	0	0	3	1	2	1	0	54	1,71	18,47	0

825	1	1	0	0	0	1	1	2	1	0	70	1,6	27,34	0
826	1	1	0	0	0	1	1	2	1	0	70	1,6	27,34	0
827	1	1	0	0	0	1	1	2	1	0	70	1,6	27,34	0
828	1	1	0	0	0	1	1	2	1	0	69,5	1,72	23,49	0
829	1	0	0	0	0	1	1	4	1	2	53	1,71	18,13	0
830	1	1	0	0	0	1	1	5	2	2	59	1,7	20,42	0
831	0	0	0	1	0	1	1	4	1	2	72	1,65	26,45	0
832	0	0	0	1	0	1	1	4	1	2	72	1,65	26,45	0
833	2	0	0	0	0	1	0	1	0	0	50	1,56	20,55	0
834	2	0	0	0	0	1	0	1	0	0	50	1,56	20,55	0
835	1	1	1	0	0	6	1	2	1	0	52	1,5	23,11	0
836	1	0	0	0	0	3	1	4	2	1	75	1,68	26,57	0
837	1	0	0	0	1	1	1	2	1	0	57	1,61	21,99	0
838	1	0	0	0	1	1	1	2	1	0	57	1,61	21,99	0
839	1	0	0	0	0	1	1	2	1	0	68	1,7	23,53	0
840	1	0	0	0	0	1	1	2	1	0	68	1,7	23,53	0
841	2	0	1	0	0	1	0	2	0	1	75	1,66	27,22	0
842	2	0	1	0	0	1	0	2	0	1	75	1,66	27,22	0
843	2	1	0	0	1	1	0	1	0	0	75	1,81	22,89	0
844	2	1	0	0	1	1	0	1	0	0	75	1,81	22,89	0
845	1	0	0	0	1	1	1	3	1	1	92	1,68	32,6	0
846	1	0	0	0	1	1	1	3	1	1	92	1,68	32,6	0
847	0	0	0	0	0	1	1	2	1	0	67	1,82	20,23	0
848	0	0	0	0	0	1	1	2	1	0	67	1,82	20,23	0
849	2	0	0	0	0	1	0	1	0	0	60	1,7	20,76	0
850	2	0	0	0	0	1	0	1	0	0	60	1,7	20,76	0
851	1	0	0	0	0	5	0	2	0	1	64	1,54	26,99	0
852	1	0	0	0	0	5	0	2	0	1	64	1,54	26,99	0
853	1	0	0	0	0	2	1	3	2	0	61	1,72	20,62	0
854	1	1	0	1	1	1	1	5	1	3	70	1,78	22,09	0
855	1	1	0	1	1	1	1	5	1	3	70	1,78	22,09	0
856	1	0	0	0	0	1	0	3	0	2	67	1,73	22,39	0
857	0	0	0	0	1	1	1	2	1	0	52	1,58	20,83	0

858	0	0	0	0	1	1	1	2	1	0	52	1,58	20,83	0
859	0	1	0	0	0	6	1	3	2	0	50	1,62	19,05	0
860	0	1	0	0	0	6	1	3	2	0	50	1,62	19,05	0
861	2	1	1	0	1	1	1	2	1	0	90	1,65	33,06	0
862	2	1	1	0	1	1	1	2	1	0	90	1,65	33,06	0
863	2	1	1	0	1	1	1	2	1	0	90	1,65	33,06	0
864	1	0	0	0	1	1	0	3	0	2	64	1,73	21,38	0
865	1	0	0	0	1	1	0	3	0	2	64	1,73	21,38	0
866	1	1	1	0	1	1	1	3	2	0	119	1,76	38,42	0
867	1	1	1	0	1	1	1	3	2	0	119	1,76	38,42	0
868	0	0	0	0	1	1	1	2	1	0	72	1,59	28,48	0
869	0	0	0	0	1	1	1	2	1	0	72	1,59	28,48	0
870	0	0	0	0	1	1	1	3	1	1	56	1,6	21,88	0
871	0	0	0	0	1	1	1	3	1	1	56	1,6	21,88	0
872	1	0	0	0	0	1	1	3	1	1	75	1,76	24,21	0
873	1	0	0	0	0	1	1	3	1	1	75	1,76	24,21	0
874	1	0	0	0	0	1	0	1	0	0	55	1,6	21,48	0
875	1	0	0	0	0	1	0	1	0	0	55	1,6	21,48	0
876	1	0	0	0	0	1	1	2	1	0	62	1,65	22,77	0
877	1	0	0	0	1	1	0	1	0	0	62	1,7	21,45	0
878	1	0	0	0	1	1	0	1	0	0	62	1,7	21,45	0
879	0	0	0	0	0	1	0	2	0	1	65	1,65	23,88	0
880	0	0	0	0	0	1	0	2	0	1	65	1,65	23,88	0
881	2	0	0	0	0	1	0	1	0	0	96	1,84	28,36	0
882	2	0	0	0	0	1	0	1	0	0	96	1,84	28,36	0
883	2	0	0	0	0	1	1	3	1	1	76	1,68	26,93	0
884	2	0	0	0	0	1	1	3	1	1	76	1,68	26,93	0
885	1	0	0	1	0	2	1	2	1	0	59	1,72	19,94	0
886	1	0	0	1	0	2	1	2	1	0	59	1,72	19,94	0
887	1	0	0	0	1	1	0	3	0	2	66,2	1,7	22,91	0
888	2	0	0	0	0	1	1	2	1	0	62	1,64	23,05	0
889	2	0	0	0	0	1	1	2	1	0	62	1,64	23,05	0
890	1	0	0	1	0	2	1	3	2	0	78	1,57	31,64	0

891	1	0	0	0	0	3	0	2	0	1	109	1,73	36,42	0
892	1	0	0	0	0	3	0	2	0	1	109	1,73	36,42	0
893	1	0	0	0	0	2	0	2	0	1	56	1,65	20,57	0
894	1	0	0	1	0	1	0	3	0	2	106	1,78	33,46	0
895	1	0	0	1	0	1	0	3	0	2	106	1,78	33,46	0
896	1	1	1	0	1	1	1	4	2	1	96	1,65	35,26	0
897	1	0	0	0	1	1	1	2	1	0	62	1,7	21,45	0
898	1	0	0	0	1	1	1	2	1	0	62	1,7	21,45	0
899	1	0	0	0	1	1	0	1	0	0	50	1,61	19,29	0
900	1	0	0	0	1	1	0	1	0	0	50	1,61	19,29	0
901	2	0	0	0	0		1	2	1	0	55	1,66	19,96	0
902	2	0	0	0	1	1	0	1	0	0	59	1,63	22,21	0
903	2	0	0	0	1	1	0	1	0	0	59	1,63	22,21	0
904	2	0	1	0	0	1	1	4	1	2	70	1,76	22,60	0
905	2	0	0	0	1	1	0	1	0	0	68	1,8	20,99	0
906	2	0	0	0	1	1	0	1	0	0	68	1,8	20,99	0
907	1	0	0	0	0	1	1	2	1	0	60	1,67	21,51	0
908	1	0	0	0	0	1	1	2	1	0	60	1,67	21,51	0
909	1	1	0	0	0	2	1	4	1	2	107	1,64	39,78	0
910	0	0	1	0	1	1	0	1	0	0	87	1,58	34,85	0
911	1	1	0	0	0	1	0	1	0	0	62	1,64	23,05	0
912	2	0	0	0	0		1	2	1	0	54	1,68	19,13	0
913	0	0	0	0	0	1	0	1	0	0	64	1,78	20,2	0
914	0	0	0	0	0	1	0	1	0	0	64	1,78	20,2	0
915	1	0	0	0	0	1	1	5	1	3	85	1,66	30,85	0
916	1	0	0	0	0	1	1	5	1	3	85	1,66	30,85	0
917	1	0	1	0	0	2	1	3	1	1	56	1,6	21,88	0
918	0	1	0	1	0	1	1	2	1	0	73	1,68	25,86	0
919	0	1	0	1	0	1	1	2	1	0	73	1,68	25,86	0
920	2	0	0	0	0	1	1	2	1	0	59	1,76	19,05	0
921	2	0	0	0	0	3	1	4	1	2	66	1,64	24,54	0
922	1	0	0	0	0	4	1	5	2	2	53	1,49	23,87	0
923	1	0	0	0	0	4	1	5	2	2	53	1,49	23,87	0

924	1	1	0	0	0	2	0	1	0	0	90,4	1,7	31,28	0
925	2	1	0	0	0	3	1	2	1	0	74	1,58	29,64	0
926	2	1	0	0	0	3	1	2	1	0	74	1,58	29,64	0
927	0	0	0	0	1	1	1	2	1	0	56	1,68	19,84	0
928	0	0	0	0	1	1	1	2	1	0	56	1,68	19,84	0
929	0	0	0	0	0	1	1	2	1	0	58	1,7	20,07	0
930	0	0	0	0	0	1	1	2	1	0	58	1,7	20,07	0
931	1	0	0	0	0		1	2	1	0	64	1,67	22,95	0
932	0	0	0	0	1	1	0	1	0	0	73	1,82	22,04	0
933	0	0	0	0	1	1	0	1	0	0	73	1,82	22,04	0
934	1	0	0	0	1	1	1	2	1	0	69	1,64	25,65	0
935	1	0	0	0	1	1	1	2	1	0	69	1,64	25,65	0
936	2	0	0	0	1	1	0	1	0	0	106	1,69	37,11	0
937	2	0	0	0	1	1	0	1	0	0	106	1,69	37,11	0
938	2	0	1	0	1	3	0	1	0	0	73	1,5	32,44	0
939	2	0	1	0	1	3	0	1	0	0	73	1,5	32,44	0
940	0	0	0	0	0	1	0	1	0	0	88	1,69	30,81	0
941	0	0	0	0	0	1	0	1	0	0	88	1,69	30,81	0
942	0	1	0	0	0	2	0	1	0	0	77	1,81	23,5	0
943	0	1	0	0	0	2	0	1	0	0	77	1,81	23,5	0
944	1	1	0	0	0	3	1	2	1	0	68	1,7	23,53	0
945	0	0	0	0	1	1	0	1	0	0	58	1,68	20,55	0
946	0	0	0	0	1	1	0	1	0	0	58	1,68	20,55	0
947	1	0	0	0	0	3	0	1	0	0	75	1,57	30,43	0
948	1	0	0	0	1	1	1	2	1	0	100	1,58	40,06	0
949	1	0	0	0	1	1	1	2	1	0	100	1,58	40,06	0
950	1	1	0	0	0	6	0	1	0	0	81	1,68	28,7	0
951	1	1	0	0	0	6	0	1	0	0	81	1,68	28,7	0
952	0	0	0	0	0	3	0	1	0	0	61	1,65	22,41	0
953	0	0	0	0	0	3	0	2	0	1	66	1,68	23,38	0
954	0	0	1	0	0	2	0	1	0	0	58	1,72	19,61	0
955	0	0	1	0	0	2	0	1	0	0	58	1,72	19,61	0
956	2	0	0	0	1	1	0	1	0	0	83	1,61	32,02	0

957	2	0	0	0	1	1	0	1	0	0	83	1,61	32,02	0
958	0	0	0	0	0	3	1	2	1	0	65	1,65	23,88	0
959	0	0	0	0	0	6	0	1	0	0	63	1,72	21,3	0
960	0	0	0	0	0	6	0	1	0	0	63	1,72	21,3	0
961	1	0	1	0	1	1	0	2	0	1	91	1,63	34,25	0
962	1	0	1	0	1	1	0	2	0	1	91	1,63	34,25	0
963	0	0	0	0	1	1	0	1	0	0	62	1,75	20,24	0
964	0	0	0	0	1	1	0	1	0	0	62	1,75	20,24	0
965	1	0	1	1	0	3	0	4	0	3	71	1,59	28,08	0
966	1	0	1	1	0	3	0	4	0	3	71	1,59	28,08	0
967	1	0	1	1	0	3	0	4	0	3	71	1,59	28,08	0
968	0	0	0	1	1	1	0	2	0	1	58	1,68	20,55	0
969	0	0	0	1	1	1	0	2	0	1	58	1,68	20,55	0
970	1	0	0	0	0	1	1	3	1	1	80	1,73	26,73	0
971	1	0	0	0	0	1	1	3	1	1	80	1,73	26,73	0
972	2	0	0	0	1	3	0	1	0	0	77	1,57	31,24	0
973	1	0	0	0	0	1	1	3	1	1	63,5	1,75	20,73	0
974	0	0	0	0	1	1	0	1	0	0	64	1,58	25,64	0
975	0	0	0	0	1	1	0	1	0	0	64	1,58	25,64	0
976	2	0	0	0	1	1	1	3	1	1	97	1,71	33,17	0
977	2	0	0	0	1	1	1	3	1	1	97	1,71	33,17	0
978	1	0	0	0	0	1	1	2	1	0	60	1,68	21,26	0
979	2	0	0	0	0	2	1	2	1	0	54	1,6	21,09	0
980	1	0	0	0	0		0	1	0	0	55,6	1,6	21,72	0
981	0	0	0	1	1	3	1	3	2	0	69	1,7	23,88	1
982	0	0	0	1	1	3	1	3	2	0	69	1,7	23,88	1
983	0	1	0	0	0	1	1	2	1	0	60	1,68	21,26	0
984	0	1	0	0	0	1	1	2	1	0	60	1,68	21,26	0
985	0	0	0	0	0	1	1	2	1	0	78	1,6	30,47	0
986	0	0	0	0	0	1	1	2	1	0	78	1,6	30,47	0
987	1	1	0	0	1	1	0	1	0	0	49	1,68	17,36	0
988	1	1	0	0	1	1	0	1	0	0	49	1,68	17,36	0
989	1	1	0	0	0	3	1	2	1	0	64	1,56	26,3	0

990	1	1	0	0	0	3	1	2	1	0	64	1,56	26,3	0
991	2	0	0	0	0	1	0	1	0	0	73	1,69	25,56	0
992	2	0	0	0	0	1	0	1	0	0	73	1,69	25,56	0
993	1	0	0	0	0	3	1	3	2	0	75	1,58	30,04	0
994	0	1	0	0	1	1	1	2	1	0	86	1,72	29,07	0
995	0	1	0	0	1	1	1	2	1	0	86	1,72	29,07	0
996	0	1	0	0	1	1	1	2	1	0	86	1,72	29,07	0
997	0	1	0	0	0	1	1	2	1	0	81	1,65	29,75	0
998	0	1	0	0	0	1	1	2	1	0	81	1,65	29,75	0
999	2	1	0	1	0	1	1	2	1	0	65,5	1,7	22,66	0
1000	1	0	0	1	0	1	0	2	0	1	56,3	1,68	19,95	0
1001	2	0	0	0	0	1	0	2	0	1	60	1,84	17,72	0
1002	1	0	0	0	0	2	1	2	1	0	100	1,68	35,43	0
1003	2	1	1	0	1	1	0	1	0	0	132	1,66	47,9	0
1004	2	1	1	0	1	1	0	1	0	0	132	1,66	47,9	0
1005	2	1	1	0	1	1	0	1	0	0	132	1,66	47,9	0
1006	1	0	0	0	1	1	1	4	2	1	81	1,84	23,92	0
1007	1	0	0	0	1	1	1	4	2	1	81	1,84	23,92	0
1008	1	0	0	0	1	1	1	4	2	1	81	1,84	23,92	0
1009	1	0	0	0	0	1	1	2	1	0	82	1,6	32,03	0
1010	1	0	0	0	0	1	1	2	1	0	82	1,6	32,03	0
1011	1	0	0	0	0	1	1	3	2	1	85	1,75	27,76	0
1012	1	0	0	0	0	1	1	3	2	1	85	1,75	27,76	0
1013	2	0	0	0	0	1	1	3	1	1	68	1,62	25,91	0
1014	2	0	0	0	0	1	1	3	1	1	68	1,62	25,91	0
1015	0	0	0	0	0	3	1	3	1	1	72	1,65	26,45	1
1016	0	1	1	0	0	1	0	1	0	0	48	1,54	20,24	0
1017	0	1	1	0	0	1	0	1	0	0	48	1,54	20,24	0
1018	0	0	0	0	0	1	1	2	1	0	53	1,63	19,95	0
1019	1	0	0	0	0	1	1	2	1	0	58	1,63	21,83	0
1020	1	0	0	0	0	1	1	2	1	0	58	1,63	21,83	0
1021	0	0	0	0	0	2	0	1	0	0	60	1,7	20,76	0
1022	2	0	0	0	1	1	1	2	1	0	70	1,81	21,37	0

1023	2	0	0	0	1	1	1	2	1	0	70	1,81	21,37	0
1024	1	0	1	0	0	1	1	2	1	0	108	1,69	37,81	0
1025	1	1	1	0	0	2	0	1	0	0	61,5	1,65	22,59	0
1026	0	0	1	0	1	1	0	1	0	0	60	1,63	22,58	0
1027	0	0	1	0	0	2	1	3	2	0	53	1,6	20,7	0
1028	0	0	1	0	0	2	1	3	2	0	53	1,6	20,7	0
1029	0	0	1	0	0	3	1	4	1	2	56	1,67	20,08	0
1030	0	0	1	0	0	3	1	4	1	2	56	1,67	20,08	0
1031	0	0	1	0	0	1	1	2	1	0	83	1,56	34,11	0
1032	0	0	1	0	0	1	1	2	1	0	83	1,56	34,11	0
1033	0	0	1	0	0	1	1	2	1	0	83	1,56	34,11	0
1034	0	0	1	0	0	1	1	2	1	0	85	1,75	27,76	0
1035	0	0	1	0	0	1	1	2	1	0	85	1,75	27,76	0
1036	0	0	1	0	1	1	0	1	0	0	98	1,64	36,44	0
1037	0	0	1	0	0	1	1	2	1	0	79	1,79	24,66	0
1038	1	0	1	0	0	1	0	1	0	0	74,5	1,6	29,10	0
1039	1	0	1	0	1	1	0	2	0	1	76	1,75	24,82	0
1040	1	0	1	0	1	1	0	2	0	1	76	1,75	24,82	0
1041	1	0	1	0	0	1	0	1	0	0	59	1,74	19,49	0
1042	1	0	1	0	0	1	0	1	0	0	59	1,74	19,49	0
1043	1	0	1	0	0	1	1	2	1	0	71	1,64	26,4	0
1044	1	0	1	0	0	1	1	2	1	0	71	1,64	26,4	0
1045	1	0	1	0	0	2	1	2	1	0	87	1,7	30,1	0
1046	1	0	1	0	0	2	1	2	1	0	87	1,7	30,1	0
1047	1	0	1	0	0	2	1	2	1	0	87	1,7	30,1	0
1048	1	0	1	0	0	2	1	3	2	0	68	1,62	25,91	0
1049	1	0	1	0	0	2	1	3	1	1	56	1,55	23,31	0
1050	1	0	1	0	0	2	1	3	1	1	56	1,55	23,31	0
1051	2	0	1	0	0	1	0	1	0	0	77	1,6	30,08	0
1052	1	0	1	0	0	1	1	3	1	1	45	1,55	18,73	0
1053	1	0	1	0	0	1	1	3	1	1	45	1,55	18,73	0
1054	1	0	1	0	0	2	0	4	0	3	57	1,58	22,83	0
1055	1	0	1	0	0	2	0	4	0	3	57	1,58	22,83	0

1056	2	0	1	0	1	1	0	1	0	0	57	1,62	21,72	0
1057	2	0	1	0	1	1	0	1	0	0	71	1,76	22,92	0
1058	2	0	1	0	1	1	0	1	0	0	71	1,76	22,92	0
1059	2	1	1	0	0	1	1	3	2	0	65	1,6	25,39	0
1060	2	1	1	0	0	1	1	3	2	0	65	1,6	25,39	0
1061	2	1	1	0	0	1	1	3	2	0	65	1,6	25,39	0
1062	1	0	1	1	0	1	1	4	1	2	80	1,65	29,38	0
1063	1	0	1	1	0	1	1	4	1	2	80	1,65	29,38	0
1064	1	0	1	0	0	1	1	2	1	0	108	1,69	37,81	0
1065	2	0	1	0	1	2	1	3	1	1	71	1,55	29,55	1
1066	2	0	1	0	1	2	1	3	1	1	71	1,55	29,55	1
1067	2	0	1	0	1	2	1	3	1	1	71	1,55	29,55	1
1068	2	1	1	0	1	1	1	3	1	1	77	1,5	34,22	0
1069	2	1	1	0	1	1	1	3	1	1	77	1,5	34,22	0
1070	2	1	1	0	1	1	1	3	1	1	77	1,5	34,22	0
1071	0	0	1	0	0	3	1	4	3	0	72	1,57	29,21	0
1072	0	0	1	0	0	3	1	4	3	0	72	1,57	29,21	0
1073	0	0	1	0	0	4	1	2	1	0	66	1,6	25,78	0
1074	1	0	1	0	0	1	1	2	1	0	58	1,6	22,66	0
1075	2	0	1	0	0	1	1	5	3	1	70	1,78	22,09	0
1076	2	0	1	0	0	1	1	5	3	1	70	1,78	22,09	0
1077	2	0	1	0	0	3	1	2	1	0	51	1,5	22,67	0
1078	1	1	1	0	0	1	1	3	1	1	59	1,67	21,16	0
1079	2	1	1	1	1	1	1	2	1	0	111	1,78	35,03	0
1080	2	1	1	1	1	1	1	2	1	0	111	1,78	35,03	0
1081	1	0	0	0	1	1	0	1	0	0	88	1,74	29,07	0
1082	1	0	0	1	0	1	0	2	0	1	64	1,69	22,41	0
1083	1	0	0	1	0	1	0	2	0	1	64	1,69	22,41	0
1084	1	0	0	0	0	5	1	4	3	0	74	1,63	27,85	0
1085	1	0	0	0	0	5	1	4	3	0	74	1,63	27,85	0
1086	1	1	0	0	1	1	0	2	0	1	92	1,5	40,89	0
1087	1	1	0	0	1	1	0	2	0	1	92	1,5	40,89	0
1088	0	0	0	0	0	4	0	1	0	0	45	1,6	17,58	0

1089	1	0	0	0	0	1	1	4	3	0	96,5	1,65	35,45	0
1090	2	0	0	0	1	3	1	2	1	0	54	1,6	21,09	0
1091	2	0	0	0	1	3	1	2	1	0	54	1,6	21,09	0
1092	2	0	0	0	0	1	0	1	0	0	91	1,64	33,83	0
1093	0	0	0	0	0	2	0	1	0	0	43	1,54	18,13	0
1094	2	0	1	1	0	3	1	4	1	2	53,5	1,64	19,89	1
1095	1	0	0	0	0	2	1	3	1	1	64	1,78	20,2	0
1096	1	0	0	0	0	2	1	3	1	1	64	1,78	20,2	0
1097	1	0	0	0	1	1	0	3	0	2	78	1,69	27,31	0
1098	1	0	0	0	1	1	0	3	0	2	78	1,69	27,31	0
1099	2	0	0	0	1	1	0	1	0	0	72	1,68	25,51	0
1100	2	0	0	0	1	1	0	1	0	0	72	1,68	25,51	0
1101	2	0	0	0	1	1	0	1	0	0	72	1,68	25,51	0
1102	1	0	0	0	0	2	1	6	1	4	67,5	1,68	23,92	1
1103	1	1	0	0	0	1	1	5	1	3	75	1,7	25,95	0
1104	1	1	0	0	0	1	1	5	1	3	75	1,7	25,95	0
1105	1	1	0	0	0	1	1	5	1	3	75	1,7	25,95	0
1106	0	0	0	0	0	6	0	1	0	0	62	1,62	23,62	0
1107	0	0	0	0	0	6	0	1	0	0	62	1,62	23,62	0
1108	1	1	1	0	0	6	0	2	0	1	78	1,55	32,47	0
1109	1	1	1	0	0	6	0	2	0	1	78	1,55	32,47	0
1110	1	0	0	0	1	1	0	2	0	1	65	1,74	21,47	0
1111	1	0	0	0	1	1	0	2	0	1	65	1,74	21,47	0
1112	1	0	0	0	1	1	0	2	0	1	65	1,74	21,47	0
1113	0	1	0	1	0	1	1	2	1	0	49	1,68	17,36	0
1114	1	0	0	0	0	1	0	1	0	0	68	1,74	22,46	0
1115	1	0	0	0	0	1	0	1	0	0	68	1,74	22,46	0
1116	2	0	0	0	1	2	0	1	0	0	65	1,71	22,23	0
1117	1	1	0	0	0	1	1	3	1	1	89	1,68	31,53	0
1118	1	1	0	0	0	1	1	3	1	1	89	1,68	31,53	0
1119	1	1	0	0	0	1	1	3	1	1	89	1,68	31,53	0
1120	0	0	0	1	1	1	0	1	0	0	52	1,65	19,10	0
1121	0	0	0	0	0		1	6	3	2	52	1,62	19,81	0

1122	0	0	0	0	0		1	6	3	2	52	1,62	19,81	0
1123	1	0	0	0	0	1	1	5	2	2	92	1,8	28,4	0
1124	1	0	0	0	0	1	1	5	2	2	92	1,8	28,4	0
1125	1	0	0	0	0	2	0	2	0	1	47	1,58	18,83	0
1126	1	0	0	0	0	2	0	2	0	1	47	1,58	18,83	0
1127	0	0	0	0	0	1	0	1	0	0	67	1,64	24,91	0
1128	0	0	0	0	0	1	1	2	1	0	92	1,7	31,83	0
1129	0	0	0	0	0	1	0	2	0	1	59	1,64	21,94	0
1130	0	0	0	0	0	1	1	2	1	0	92	1,7	31,83	0
1131	0	0	0	0	0	3	0	1	0	0	64	1,6	25	0
1132	0	0	0	0	1	3	1	2	1	0	50	1,63	18,82	0
1133	0	0	0	0	1	3	1	2	1	0	50	1,63	18,82	0
1134	0	0	0	0	0	3	0	1	0	0	64	1,6	25	0
1135	0	0	0	0	0	2	1	2	1	0	69	1,58	27,64	0
1136	0	0	0	0	0	2	1	2	1	0	69	1,58	27,64	0
1137	0	0	0	0	0	2	1	2	1	0	69	1,58	27,64	0
1138	0	0	0	0	0	3	1	2	1	0	62	1,6	24,22	0
1139	0	0	0	0	0	3	1	2	1	0	62	1,6	24,22	0
1140	0	0	0	0	0	1	0	1	0	0	57,5	1,73	19,21	0
1141	0	0	0	0	0	1	0	1	0	0	65	1,64	24,17	0
1142	0	0	0	0	1	1	1	2	1	0	66	1,7	22,84	0
1143	0	0	0	0	0	3	1	2	1	0	59	1,69	20,66	0
1144	0	0	0	0	0	1	1	2	1	0	54	1,67	19,36	0
1145	0	0	0	0	1	1	1	2	1	0	66	1,7	22,84	0
1146	0	0	0	0	0	1	0	3	0	2	72	1,73	24,06	0
1147	0	0	0	0	0	1	1	2	1	0	54	1,67	19,36	0
1148	0	0	0	0	0	1	0	3	0	2	72	1,73	24,06	0
1149	0	0	0	0	0	1	0	2	0	1	61	1,72	20,62	0
1150	0	0	0	0	0	1	0	2	0	1	61	1,72	20,62	0
1151	0	0	0	0	0	1	0	2	0	1	56	1,69	19,61	0
1152	0	0	0	0	0	1	0	2	0	1	56	1,69	19,61	0
1153	0	0	1	0	1	1	1	2	1	0	57	1,6	22,27	0
1154	0	0	0	0	0	3	0	1	0	0	50	1,7	17,30	0

1155	0	0	1	0	0	2	0	2	0	1	61	1,75	19,92	0
1156	0	0	0	0	0	3	1	4	2	1	69	1,67	24,74	0
1157	0	0	1	0	0	2	0	2	0	1	61	1,75	19,92	0
1158	0	0	0	0	0	3	1	4	2	1	69	1,67	24,74	0
1159	0	0	0	0	0	1	0	1	0	0	68	1,7	23,53	0
1160	0	0	0	0	0	1	0	1	0	0	68	1,7	23,53	0
1161	0	0	0	0	0	3	0	1	0	0	58	1,66	21,05	0
1162	0	0	0	0	0	1	1	2	1	0	65	1,63	24,46	0
1163	0	0	0	0	0	1	1	2	1	0	65	1,63	24,46	0
1164	0	0	0	0	0	1	0	2	0	1	63,5	1,63	23,90	0
1165	0	0	0	0	1	1	1	2	1	0	62	1,58	24,84	0
1166	0	0	0	0	0		0	1	0	0	79	1,64	29,37	0
1167	0	0	0	0	0	3	1	2	1	0	52	1,61	20,06	0
1168	0	0	0	0	0	1	0	1	0	0	69	1,71	23,60	0
1169	0	0	0	0	0	1	1	2	1	0	62	1,73	20,72	0
1170	0	0	0	0	0	1	1	2	1	0	62	1,73	20,72	0
1171	0	0	0	0	0	1	0	1	0	0	63	1,7	21,8	0
1172	0	0	1	0	1	3	0	2	0	1	63	1,6	24,61	0
1173	0	0	0	0	0	1	0	1	0	0	63	1,7	21,8	0
1174	0	0	1	0	1	3	0	2	0	1	63	1,6	24,61	0
1175	0	0	0	0	0	1	1	2	1	0	78	1,61	30,09	0
1176	0	0	0	0	0	2	1	3	2	0	82	1,65	30,12	0
1177	0	0	0	0	0	2	0	1	0	0	60	1,6	23,44	0
1178	0	0	0	0	0	2	0	1	0	0	60	1,6	23,44	0
1179	0	0	0	0	0	1	1	2	1	0	78	1,61	30,09	0
1180	0	0	0	0	0	1	0	1	0	0	60	1,68	21,26	0
1181	0	0	0	0	0	1	0	1	0	0	69	1,73	23,05	0
1182	0	0	0	0	0	1	0	1	0	0	69	1,73	23,05	0
1183	0	0	0	0	0	3	1	2	1	0	57	1,67	20,44	0
1184	0	0	0	0	0	1	1	2	1	0	68	1,73	22,72	0
1185	0	0	0	0	0	1	0	1	0	0	62	1,67	22,23	0
1186	0	0	0	0	0	3	1	2	1	0	57	1,67	20,44	0
1187	0	0	0	0	0	1	0	1	0	0	88	1,68	31,18	0

1188	0	0	0	0	0	1	1	2	1	0	68	1,73	22,72	0
1189	0	0	0	0	0	1	1	3	1	1	50	1,62	19,05	0
1190	0	0	0	0	0	1	0	1	0	0	88	1,68	31,18	0
1191	0	0	0	0	0	1	0	1	0	0	62	1,67	22,23	0
1192	0	0	0	0	0	1	1	2	1	0	91	1,78	28,72	0
1193	0	0	0	0	0	1	1	2	1	0	72	1,75	23,51	0
1194	0	0	0	0	0	1	1	2	1	0	91	1,78	28,72	0
1195	0	0	0	0	0	1	1	2	1	0	72	1,75	23,51	0
1196	0	0	0	0	0	1	1	2	1	0	91	1,78	28,72	0
1197	0	0	0	0	0	2	1	3	1	1	59,5	1,73	19,88	0
1198	0	0	0	1	0	1	0	1	0	0	64	1,61	24,69	0
1199	0	0	0	1	0	1	0	1	0	0	64	1,61	24,69	0
1200	0	0	1	0	0	1	0	1	0	0	64	1,63	24,09	0
1201	0	1	0	0	0	2	1	2	1	0	85	1,68	30,12	1
1202	0	0	0	0	0	1	1	2	1	0	86	1,73	28,73	0
1203	0	0	0	0	1	1	0	1	0	0	69	1,6	26,95	0
1204	0	0	0	0	1	1	0	1	0	0	69	1,6	26,95	0
1205	0	0	0	0	1	1	0	1	0	0	69	1,6	26,95	0
1206	0	0	0	0	0	1	1	3	2	0	66	1,68	23,38	0
1207	0	0	0	0	0	1	1	3	2	0	66	1,68	23,38	0
1208	0	0	0	0	0	1	0	1	0	0	59	1,6	23,05	0
1209	0	0	0	0	0	4	0	1	0	0	45	1,55	18,73	0
1210	0	0	0	0	0	2	0	1	0	0	72	1,75	23,51	0
1211	0	0	0	0	0	4	0	1	0	0	45	1,55	18,73	0
1212	0	1	0	0	0	1	1	3	2	0	69	1,65	25,34	0
1213	0	0	0	0	0	1	0	1	0	0	75	1,6	29,3	0
1214	0	0	0	0	0	6	1	3	1	1	69	1,63	25,97	0
1215	0	0	0	0	0	2	1	3	1	1	71	1,76	22,92	0
1216	0	0	0	0	0	1	0	1	0	0	75	1,6	29,3	0
1217	0	0	0	0	0	1	1	2	1	0	52	1,7	17,99	0
1218	0	0	0	0	0	2	1	3	1	1	71	1,76	22,92	0
1219	0	1	0	0	1	1	0	1	0	0	81	1,73	27,06	0
1220	0	0	0	0	0	6	1	3	1	1	69	1,63	25,97	0

1221	0	0	0	0	0	1	0	1	0	0	98	1,76	31,64	0
1222	0	0	0	0	1	1	0	1	0	0	84,8	1,78	26,76	0
1223	0	0	0	0	0	1	0	1	0	0	98	1,76	31,64	0
1224	0	1	0	0	0	1	1	3	2	0	84	1,69	29,41	0
1225	0	0	0	0	0	1	1	2	1	0	72	1,68	25,51	0
1226	0	1	0	0	0	1	1	3	2	0	84	1,69	29,41	0
1227	0	0	0	0	0	6	0	1	0	0	62	1,6	24,22	0
1228	0	0	0	0	0	1	1	2	1	0	73	1,72	24,68	0
1229	0	0	0	0	0	1	1	2	1	0	73	1,72	24,68	0
1230	1	1	1	0	0	3	1	3	2	0	49	1,57	19,88	0
1231	1	1	0	0	1	1	1	2	1	0	77	1,6	30,08	0
1232	1	1	0	0	1	1	1	2	1	0	77	1,6	30,08	0
1233	1	1	0	0	0	2	1	2	1	0	69	1,7	23,88	0
1234	2	0	0	0	0	1	1	2	1	0	48,5	1,59	19,18	0
1235	1	0	0	0	1	3	1	3	2	0	88	1,65	32,32	0
1236	1	0	0	0	1	3	1	3	2	0	88	1,65	32,32	0
1237	1	0	0	0	0	1	0	2	0	1	68	1,77	21,71	0
1238	1	0	0	0	0	1	0	2	0	1	68	1,77	21,71	0
1239	1	0	0	0	1	1	1	3	1	1	70	1,6	27,34	0
1240	1	0	0	0	1	1	1	3	1	1	70	1,6	27,34	0
1241	1	0	0	0	0		1	5	2	2	53	1,63	19,95	0
1242	1	0	0	0	0	1	1	5	3	1	62	1,68	21,97	0
1243	1	0	0	0	0	1	1	5	3	1	62	1,68	21,97	0
1244	1	0	0	0	0	1	0	3	0	2	59	1,7	20,42	0
1245	1	0	0	0	0	2	0	2	0	1	58	1,71	19,84	0
1246	1	0	0	0	0	2	1	7	4	2	59	1,7	20,42	0
1247	1	0	0	0	0	2	1	7	4	2	59	1,7	20,42	0
1248	1	0	0	0	0	2	0	4	0	3	77	1,7	26,64	0
1249	1	0	0	0	0	3	1	4	1	2	62	1,65	22,77	0
1250	2	0	0	0	1	1	0	3	0	2	47	1,65	17,26	0
1251	2	0	0	0	1	1	0	3	0	2	47	1,65	17,26	0
1252	2	0	0	0	0	3	0	1	0	0	67	1,72	22,65	0
1253	2	0	0	0	0	3	0	1	0	0	67	1,72	22,65	0

1254	1	0	0	0	1	1	1	2	1	0	70	1,76	22,6	0
1255	1	0	0	0	1	1	1	2	1	0	70	1,76	22,6	0
1256	1	0	0	0	0	3	0	1	0	0	51,5	1,59	20,37	0
1257	2	0	1	0	0	1	1	3	2	0	85	1,61	32,79	0
1258	2	0	1	0	0	1	1	3	2	0	85	1,61	32,79	0
1259	1	0	0	0	0	1	0	2	0	1	60,5	1,7	20,93	0
1260	1	0	0	0	1	3	1	3	1	1	65	1,58	26,04	0
1261	1	0	0	0	1	3	1	3	1	1	65	1,58	26,04	0
1262	1	0	0	0	0	3	0	1	0	0	75	1,68	26,57	0
1263	1	0	1	0	0	1	0	2	0	1	79,9	1,7	27,65	0
1264	1	0	0	0	0	6	1	2	1	0	151	1,7	52,25	0
1265	1	0	0	0	0	6	1	2	1	0	151	1,7	52,25	0
1266	2	0	0	0	0	2	1	2	1	0	86	1,7	29,76	0
1267	2	0	0	0	0	1	0	1	0	0	74	1,7	25,61	0
1268	2	0	0	0	0	1	0	1	0	0	74	1,7	25,61	0
1269	1	0	0	0	0	1	1	3	2	0	68,5	1,8	21,14	0
1270	1	0	0	0	0	1	1	2	1	0	48	1,57	19,47	0
1271	1	0	0	0	0	1	1	3	1	1	97	1,6	37,89	0
1272	1	0	0	0	0	1	0	1	0	0	67	1,82	20,23	0
1273	1	0	0	0	0	1	0	1	0	0	67	1,82	20,23	0
1274	2	0	0	0	0	1	1	2	1	0	57	1,63	21,45	0
1275	2	0	0	0	0	1	1	2	1	0	57	1,63	21,45	0
1276	2	1	0	0	0	1	0	1	0	0	77	1,65	28,28	0
1277	1	0	0	0	0	1	0	1	0	0	50	1,57	20,28	0
1278	1	0	0	0	0	1	0	1	0	0	50	1,57	20,28	0
1279	2	0	0	0	0	1	1	3	1	1	68	1,79	21,22	0
1280	2	0	0	0	0	1	1	3	1	1	68	1,79	21,22	0
1281	2	0	0	0	0	1	1	3	1	1	68	1,79	21,22	0
1282	2	0	0	0	0	1	1	2	1	0	56	1,69	19,61	0
1283	2	0	0	0	0	1	1	2	1	0	56	1,69	19,61	0
1284	1	0	0	0	0	3	1	2	1	0	59	1,68	20,90	0
1285	2	0	0	0	1	1	0	1	0	0	62	1,68	21,97	0
1286	2	0	0	0	1	1	0	1	0	0	62	1,68	21,97	0

1287	1	0	0	0	0	1	1	2	1	0	74	1,73	24,73	0
1288	1	0	0	0	0	1	1	2	1	0	74	1,73	24,73	0
1289	1	0	0	0	0	1	0	2	0	1	56	1,77	17,87	0
1290	1	0	0	0	0	1	0	2	0	1	56	1,77	17,87	0
1291	1	0	0	0	0	1	0	1	0	0	53	1,68	18,78	0
1292	1	0	0	0	0	1	0	1	0	0	53	1,68	18,78	0
1293	1	0	0	0	0	1	0	1	0	0	53	1,68	18,78	0
1294	1	0	0	0	0	1	1	3	1	1	60	1,66	21,77	0
1295	1	0	0	0	0	1	1	3	1	1	60	1,66	21,77	0
1296	1	0	0	0	1	1	1	3	1	1	92	1,66	33,39	0
1297	1	1	0	0	0	3	0	1	0	0	65	1,63	24,46	0
1298	1	0	0	0	0	1	1	3	1	1	63	1,58	25,24	0
1299	1	0	0	0	0	1	1	3	1	1	63	1,58	25,24	0
1300	2	0	0	0	1	1	0	1	0	0	80	1,68	28,34	0
1301	2	0	0	0	0	1	0	1	0	0	50	1,65	18,37	0
1302	2	0	1	0	1	1	1	2	1	0	85	1,72	28,73	0
1303	2	0	1	0	1	1	1	2	1	0	85	1,72	28,73	0
1304	2	0	0	0	0	1	0	1	0	0	50	1,67	17,93	0
1305	2	0	0	0	0	1	0	1	0	0	50	1,67	17,93	0
1306	2	0	0	0	0	1	0	1	0	0	61	1,69	21,36	0
1307	2	0	0	0	0	1	0	1	0	0	61	1,69	21,36	0
1308	2	0	0	0	0	1	0	1	0	0	61	1,69	21,36	0
1309	2	0	0	0	0	1	0	1	0	0	87	1,82	26,26	0
1310	2	0	0	0	0	1	0	1	0	0	87	1,82	26,26	0
1311	2	0	0	0	1	1	0	1	0	0	65	1,69	22,76	0
1312	2	0	0	0	0	1	0	2	0	1	72	1,68	25,51	0
1313	2	0	0	0	0	1	0	2	0	1	72	1,68	25,51	0
1314	1	1	1	0	1	1	1	4	1	2	107	1,65	39,3	0
1315	1	1	1	0	1	1	1	4	1	2	107	1,65	39,3	0
1316	2	0	0	0	0	1	0	3	0	2	73	1,72	24,68	0
1317	2	0	0	0	0	1	0	3	0	2	73	1,72	24,68	0
1318	0	1	0	0	0	2	1	4	2	1	84	1,68	29,76	1
1319	0	1	0	0	0	2	1	4	2	1	84	1,68	29,76	1

1320	1	0	0	0	0	1	1	2	1	0	72	1,73	24,06	0
1321	1	0	0	0	0	1	1	2	1	0	72	1,73	24,06	0
1322	1	0	0	0	0	3	1	4	1	2	59	1,65	21,67	0
1323	0	0	0	0	0	3	1	2	1	0	64	1,58	25,64	0
1324	0	0	0	0	1	3	0	1	0	0	61	1,62	23,24	0
1325	0	0	0	0	1	3	0	1	0	0	61	1,62	23,24	0
1326	0	0	0	0	0	6	1	2	1	0	64	1,68	22,68	0
1327	0	0	0	0	0	6	1	2	1	0	64	1,68	22,68	0
1328	0	0	0	0	1	1	0	1	0	0	65	1,7	22,49	0
1329	0	0	0	0	1	1	1	2	1	0	83	1,62	31,63	0
1330	0	0	0	0	0	1	0	1	0	0	74	1,76	23,89	0
1331	1	0	0	0	1	2	1	2	1	0	60	1,67	21,51	0
1332	1	0	0	0	0	1	1	3	1	1	57	1,57	23,12	0
1333	1	0	0	0	0	1	1	3	1	1	57	1,57	23,12	0
1334	1	0	0	0	1	1	1	3	2	0	78	1,68	27,64	0
1335	0	0	0	0	0	3	1	4	3	1	55	1,57	22,31	0
1336	0	0	0	0	0	3	1	4	3	1	55	1,57	22,31	0
1337	1	0	0	0	0	1	1	2	1	0	70	1,7	24,22	0
1338	1	0	0	0	0	1	1	2	1	0	70	1,7	24,22	0
1339	0	0	0	0	0	1	1	2	1	0	76	1,76	24,54	0
1340	0	0	0	0	0	1	1	2	1	0	76	1,76	24,54	0
1341	0	0	0	0	1	2	0	1	0	0	44	1,55	18,31	0
1342	0	0	0	0	1	2	0	1	0	0	44	1,55	18,31	0
1343	1	0	0	0	0	2	1	2	1	0	56	1,65	20,57	0
1344	1	0	0	0	0	2	1	2	1	0	56	1,65	20,57	0
1345	0	1	0	0	1	2	1	3	2	0	59	1,65	21,67	0
1346	0	1	0	0	1	2	1	3	2	0	59	1,65	21,67	0
1347	1	0	0	0	0	1	0	1	0	0	58	1,67	20,8	0
1348	1	0	0	0	0	1	0	1	0	0	58	1,67	20,8	0
1349	1	1	0	0	0	1	1	2	1	0	98	1,63	36,89	0
1350	0	0	0	0	0	3	0	1	0	0	63	1,57	25,56	0
1351	0	0	0	0	0	3	0	1	0	0	63	1,57	25,56	0
1352	1	1	0	1	0	1	1	2	1	0	53	1,66	19,23	0

1353	1	1	0	1	0	1	1	2	1	0	53	1,66	19,23	0
1354	1	1	0	1	0	1	1	2	1	0	53	1,66	19,23	0
1355	1	1	0	0	0	1	1	2	1	0	65	1,58	26,04	0
1356	1	1	0	0	0	1	1	2	1	0	65	1,58	26,04	0
1357	1	1	0	0	0	1	1	2	1	0	65	1,58	26,04	0
1358	1	0	0	0	1	1	1	3	1	1	81	1,7	28,03	0
1359	1	0	0	0	1	1	1	3	1	1	81	1,7	28,03	0
1360	0	0	0	0	0	1	0	1	0	0	69	1,74	22,79	0
1361	0	0	0	0	0	1	0	1	0	0	69	1,74	22,79	0
1362	1	0	0	0	1	1	1	3	2	0	53	1,63	19,95	0
1363	0	0	0	0	1	1	0	1	0	0	58	1,65	21,3	0
1364	0	0	0	0	1	1	0	1	0	0	58	1,65	21,3	0
1365	1	0	0	0	1	2	1	4	1	2	58	1,65	21,3	0
1366	1	0	0	0	1	2	1	4	1	2	58	1,65	21,3	0
1367	0	0	0	0	0	1	1	2	1	0	77	1,74	25,43	0
1368	0	0	0	0	0	1	1	2	1	0	77	1,74	25,43	0
1369	0	0	0	0	1	6	1	2	1	0	64	1,65	23,51	0
1370	0	0	0	0	1	6	1	2	1	0	64	1,65	23,51	0
1371	0	0	0	0	0	1	1	2	1	0	69	1,62	26,29	0
1372	0	0	0	0	0	6	1	2	1	0	60	1,57	24,34	0
1373	0	0	0	0	0	6	1	2	1	0	60	1,57	24,34	0
1374	1	0	0	0	1	1	1	2	1	0	72	1,66	26,13	0
1375	1	0	0	0	1	1	1	2	1	0	72	1,66	26,13	0
1376	0	0	0	0	0	1	1	3	1	1	62	1,68	21,97	0
1377	0	0	0	0	0	1	1	3	1	1	62	1,68	21,97	0
1378	1	0	0	0	0	2	1	2	1	0	53	1,58	21,23	0
1379	1	0	0	0	0	2	1	2	1	0	53	1,58	21,23	0
1380	1	0	0	0	0	1	1	3	1	1	68	1,71	23,26	0
1381	0	0	0	0	0	3	1	2	1	0	59,5	1,68	21,08	0
1382	1	0	0	0	1	2	0	1	0	0	58	1,72	19,61	0
1383	1	0	0	0	1	2	0	1	0	0	58	1,72	19,61	0
1384	0	0	0	0	0	1	1	3	2	1	72	1,66	26,13	1
1385	0	0	0	0	0	1	1	3	2	1	72	1,66	26,13	1

1386	0	1	0	1	0	1	1	2	1	0	63	1,65	23,14	0
1387	0	1	0	1	0	1	1	2	1	0	63	1,65	23,14	0
1388	0	1	0	1	0	1	1	2	1	0	63	1,65	23,14	0
1389	2	0	0	0	0	6	0	1	0	0	77	1,68	27,28	0
1390	2	0	0	0	0	6	0	1	0	0	77	1,68	27,28	0
1391	1	0	0	0	1	1	0	1	0	0	90	1,63	33,87	0
1392	1	0	0	0	1	1	0	1	0	0	90	1,63	33,87	0
1393	2	0	0	0	0	3	0	1	0	0	69	1,63	25,97	0
1394	2	0	0	0	0	3	0	1	0	0	69	1,63	25,97	0
1395	1	0	0	0	0	1	0	2	0	1	76	1,73	25,39	0
1396	1	0	0	0	1	1	0	3	0	2	74	1,63	27,85	0
1397	1	0	0	0	1	1	0	3	0	2	74	1,63	27,85	0
1398	1	0	0	0	1	1	0	3	0	2	74	1,63	27,85	0
1399	1	0	0	0	0	2	1	3	2	0	64	1,58	25,64	0
1400	1	0	0	0	0	2	1	3	2	0	64	1,58	25,64	0
1401	0	0	0	0	0	1	0	2	0	1	62,5	1,68	22,14	0
1402	0	0	0	0	0	2	1	4	3	0	66	1,56	27,12	0
1403	0	0	0	0	0	2	1	4	3	0	66	1,56	27,12	0
1404	1	0	1	0	0	2	0	1	0	0	66	1,58	26,44	0
1405	1	0	0	0	1	2	1	2	1	0	56	1,59	22,15	0
1406	0	0	0	0	0	1	0	1	0	0	58	1,68	20,55	0
1407	2	0	0	0	1	1	0	1	0	0	77	1,65	28,28	0
1408	2	0	0	0	1	1	0	1	0	0	77	1,65	28,28	0
1409	2	0	1	0	0	1	1	2	1	0	59	1,67	21,16	0
1410	2	0	1	0	0	1	1	2	1	0	59	1,67	21,16	0
1411	1	0	0	0	1	1	0	1	0	0	52	1,53	22,21	0
1412	1	0	0	0	0	1	1	2	1	0	60	1,61	23,15	0
1413	1	0	0	0	0	1	1	2	1	0	60	1,61	23,15	0
1414	2	0	0	0	1	1	1	2	1	0	57	1,68	20,2	0
1415	2	0	0	0	1	1	1	2	1	0	57	1,68	20,2	0
1416	0	0	0	0	0	1	0	1	0	0	62	1,7	21,45	0
1417	1	0	0	0	0	2	1	3	1	1	63	1,6	24,61	0
1418	1	0	0	0	0	2	1	3	1	1	63	1,6	24,61	0

1419	0	0	0	0	1	1	0	1	0	0	72,8	1,73	24,32	0
1420	0	0	0	0	0	3	0	1	0	0	55	1,68	19,49	0
1421	1	0	0	0	0	1	1	2	1	0	64	1,7	22,15	0
1422	2	0	0	0	0	1	0	1	0	0	64	1,52	27,7	0
1423	2	0	0	0	0	1	0	1	0	0	64	1,52	27,7	0
1424	1	0	0	0	0	1	0	1	0	0	58	1,7	20,07	0
1425	1	0	0	0	0	1	0	1	0	0	58	1,7	20,07	0
1426	0	0	0	0	0	3	1	4	3	0	58	1,5	25,78	0
1427	0	0	0	0	0	3	1	4	3	0	58	1,5	25,78	0
1428	0	0	0	0	1	1	0	1	0	0	75	1,68	26,57	0
1429	0	0	0	0	1	1	0	1	0	0	75	1,68	26,57	0
1430	2	0	0	1	0	1	0	1	0	0	57	1,63	21,45	0
1431	2	0	0	1	0	1	0	1	0	0	57	1,63	21,45	0
1432	1	0	0	0	0	1	0	1	0	0	80	1,68	28,34	0
1433	1	0	0	0	0	1	0	1	0	0	80	1,68	28,34	0
1434	1	1	0	0	0	3	0	1	0	0	75	1,68	26,57	0
1435	1	1	0	0	0	3	0	1	0	0	75	1,68	26,57	0
1436	2	0	0	0	1	3	0	2	0	1	85	1,52	36,79	0
1437	2	0	0	0	1	3	0	2	0	1	85	1,52	36,79	0
1438	2	0	0	0	1	3	0	2	0	1	85	1,52	36,79	0
1439	0	1	0	0	0	1	0	2	0	1	73	1,49	32,88	0
1440	0	1	0	0	0	1	0	2	0	1	73	1,49	32,88	0
1441	1	0	0	0	0	1	0	1	0	0	57	1,67	20,44	0
1442	1	0	0	0	0	1	0	1	0	0	57	1,67	20,44	0
1443	1	0	0	0	1	1	1	3	1	1	58	1,68	20,55	0
1444	1	0	0	0	1	1	1	3	1	1	58	1,68	20,55	0
1445	2	0	0	0	1	1	0	2	0	1	80	1,73	26,73	0
1446	2	0	0	0	1	1	0	2	0	1	80	1,73	26,73	0
1447	0	0	0	0	0	2	0	1	0	0	52	1,65	19,1	0
1448	0	0	0	0	0	2	0	1	0	0	52	1,65	19,1	0
1449	0	1	0	0	1	1	0	1	0	0	72	1,73	24,06	0
1450	0	1	0	0	1	1	0	1	0	0	72	1,73	24,06	0
1451	1	0	0	0	0	3	0	2	0	1	55	1,54	23,19	0

1452	1	0	0	0	0	3	0	2	0	1	55	1,54	23,19	0
1453	1	0	0	0	0	1	0	1	0	0	60	1,69	21,01	0
1454	2	0	0	0	1	1	0	1	0	0	89	1,65	32,69	0
1455	1	0	0	0	0	2	1	2	1	0	75	1,65	27,55	1
1456	1	0	0	0	0	2	1	2	1	0	75	1,65	27,55	1
1457	1	0	0	0	0	2	1	2	1	0	75	1,65	27,55	1
1458	0	0	0	0	1	3	1	2	1	0	51	1,63	19,2	0
1459	0	0	0	0	1	3	1	2	1	0	51	1,63	19,2	0
1460	1	0	0	0	0	2	0	2	0	1	57	1,7	19,72	0
1461	1	0	0	0	0	2	0	2	0	1	57	1,7	19,72	0
1462	1	0	0	0	0	1	1	5	3	1	54	1,63	20,32	0
1463	2	0	0	0	0	3	0	1	0	0	73	1,59	28,88	0
1464	2	0	0	0	0	3	0	1	0	0	73	1,59	28,88	0
1465	0	0	0	0	0	1	1	2	1	0	57	1,65	20,94	0
1466	0	0	0	0	0	1	1	2	1	0	57	1,65	20,94	0
1467	1	1	0	0	1	1	1	3	1	1	62	1,68	21,97	0
1468	1	1	0	0	1	1	1	3	1	1	62	1,68	21,97	0
1469	1	1	0	0	1	1	1	3	1	1	62	1,68	21,97	0
1470	0	0	0	0	0		0	1	0	0	65	1,69	22,76	0
1471	0	0	0	0	0		0	1	0	0	48	1,55	19,98	0
1472	0	0	0	0	0		0	1	0	0	75	1,56	30,82	0
1473	0	1	0	0	0		1	2	1	0	69,5	1,72	23,49	0
1474	0	0	0	0	0		1	2	1	0	63	1,72	21,3	0
1475	0	0	1	0	0		1	3	1	1	70	1,65	25,71	0
1476	0	0	1	0	0		1	3	1	1	70	1,65	25,71	0
1477	0	0	1	0	0		1	2	1	0	84	1,6	32,81	0
1478	0	0	1	0	0		1	3	2	0	55	1,52	23,81	0
1479	0	0	1	0	0		0	3	0	2	75	1,68	26,57	0
1480	0	0	1	0	0		1	3	2	0	55	1,52	23,81	0
1481	0	0	1	0	0		1	4	3	1	62	1,6	24,22	1
1482	0	0	1	0	0		1	4	3	1	62	1,6	24,22	1
1483	0	0	1	0	0		1	3	2	0	68	1,57	27,59	0
1484	0	0	1	0	0		1	3	2	0	68	1,57	27,59	0

1485	2	1	0	0	0		1	4	3	0	69	1,6	26,95	0
1486	2	0	0	0	0		1	3	2	0	53	1,6	20,7	0
1487	2	0	0	0	0		1	3	2	0	53	1,6	20,7	0
1488	1	0	0	0	0		1	2	1	0	52	1,59	20,57	0
1489	1	0	0	0	0		1	5	3	1	85	1,68	30,12	0
1490	0	0	0	0	0		1	4	1	2	56	1,6	21,88	0
1491	0	0	0	0	0		1	4	1	2	56	1,6	21,88	0
1492	0	0	0	0	0		1	3	2	0	69	1,6	26,95	0
1493	0	0	0	0	0		1	2	1	0	53	1,67	19	0
1494	0	0	0	0	0		1	2	1	0	53	1,67	19	0
1495	0	0	0	0	0		1	3	2	0	69	1,6	26,95	0
1496	0	0	0	0	0		1	2	1	0	71	1,52	30,73	0
1497	0	0	0	0	0		1	2	1	0	71	1,52	30,73	0
1498	0	0	0	0	0		1	2	1	0	60	1,6	23,44	0
1499	0	0	0	0	0		1	2	1	0	60	1,6	23,44	0
1500	0	0	0	0	0		0	1	0	0	71	1,62	27,05	0
1501	0	0	0	0	0		1	2	1	0	63	1,72	21,3	0
1502	0	0	0	0	0		0	1	0	0	71	1,62	27,05	0
1503	0	0	0	0	0		0	2	0	1	66	1,78	20,83	0
1504	0	0	0	0	0		1	2	1	0	50	1,63	18,82	0
1505	0	0	0	0	0		0	2	0	1	66	1,78	20,83	0
1506	0	0	0	0	0		1	2	1	0	57	1,63	21,45	0
1507	0	0	0	0	0		1	2	1	0	57	1,63	21,45	0
1508	0	0	0	0	0		0	2	0	1	57	1,66	20,69	0
1509	0	0	0	0	0		0	1	0	0	64	1,73	21,38	0
1510	0	0	0	0	0		0	1	0	0	60	1,73	20,05	0
1511	0	0	0	0	0		0	1	0	0	64	1,73	21,38	0
1512	0	0	0	0	0		0	2	0	1	57	1,66	20,69	0
1513	0	0	0	0	0		0	1	0	0	76	1,79	23,72	0
1514	0	0	0	0	0		0	1	0	0	66	1,63	24,84	0
1515	0	0	0	0	0		0	1	0	0	66	1,63	24,84	0
1516	0	0	0	0	0		0	2	0	1	78	1,58	31,24	0
1517	0	0	0	0	0		0	1	0	0	76	1,79	23,72	0

1518	0	0	0	0	0		0	2	0	1	78	1,58	31,24	0
1519	0	0	0	0	0		0	2	0	1	71	1,74	23,45	0
1520	0	0	0	0	0		0	1	0	0	63	1,65	23,14	0
1521	0	0	0	0	0		0	1	0	0	63	1,65	23,14	0
1522	0	0	0	0	0		1	3	2	0	79	1,73	26,4	0
1523	0	0	0	0	0		1	3	2	0	56	1,8	17,28	0
1524	0	0	0	0	0		1	3	2	0	56	1,8	17,28	0
1525	0	0	0	0	0		1	2	1	0	54	1,63	20,32	0
1526	0	0	0	0	0		0	1	0	0	66	1,6	25,78	0
1527	0	0	0	0	0		1	2	1	0	54	1,63	20,32	0
1528	0	0	0	0	0		0	1	0	0	66	1,6	25,78	0
1529	0	0	0	0	0		1	2	1	0	54	1,76	17,43	0
1530	0	0	0	0	0		1	2	1	0	54	1,76	17,43	0
1531	0	0	0	0	0		1	2	1	0	52	1,59	20,57	0
1532	0	0	0	0	0		1	3	2	0	64	1,63	24,09	0
1533	0	0	0	0	0		1	3	2	0	64	1,63	24,09	0
1534	0	0	0	0	0		0	1	0	0	55	1,7	19,03	0
1535	0	0	0	0	0		0	1	0	0	55	1,7	19,03	0
1536	0	0	0	0	0		0	1	0	0	96	1,68	34,01	0
1537	0	0	0	0	0		1	2	1	0	68	1,63	25,59	0
1538	0	0	0	0	0		1	2	1	0	68	1,63	25,59	0
1539	0	1	0	0	0		1	5	3	1	50	1,7	17,3	0
1540	0	0	0	0	0		1	5	2	2	77	1,76	24,86	0
1541	0	0	0	0	0		1	5	2	2	77	1,76	24,86	0
1542	0	1	0	0	0		1	5	3	1	50	1,7	17,3	0
1543	0	0	0	0	0		0	1	0	0	96	1,68	34,01	0
1544	0	0	0	0	0		0	2	0	1	56	1,61	21,6	0
1545	0	0	0	0	0		1	2	1	0	60	1,66	21,77	0
1546	0	0	0	0	0		0	2	0	1	56	1,61	21,6	0
1547	0	0	0	0	0		0	1	0	0	54	1,58	21,63	0
1548	0	0	0	0	0		0	1	0	0	54	1,58	21,63	0
1549	0	0	0	0	0		1	2	1	0	63	1,76	20,34	0
1550	0	0	0	0	0		0	1	0	0	59	1,68	20,9	0

1551	0	0	0	0	0		0	1	0	0	59	1,68	20,9	0
1552	0	0	0	0	0		0	2	0	1	66	1,76	21,31	0
1553	0	0	0	0	0		0	2	0	1	66	1,76	21,31	0
1554	0	0	0	0	0		0	1	0	0	70	1,6	27,34	0
1555	0	0	0	0	0		0	1	0	0	70	1,6	27,34	0
1556	0	0	0	0	0		1	3	1	1	60	1,72	20,28	0
1557	0	0	0	0	0		0	1	0	0	64	1,68	22,68	0
1558	0	0	0	0	0		0	1	0	0	64	1,68	22,68	0
1559	0	0	0	0	0		1	3	1	1	60	1,72	20,28	0
1560	0	1	0	0	0		1	4	3	0	69	1,6	26,95	0
1561	0	0	0	0	0		1	3	1	1	53	1,68	18,78	0
1562	0	0	0	0	0		1	2	1	0	60	1,7	20,76	0
1563	0	0	0	0	0		1	2	1	0	60	1,7	20,76	0
1564	0	0	0	0	0		1	3	1	1	53	1,68	18,78	0
1565	0	0	0	0	0		0	2	0	1	68	1,78	21,46	0
1566	0	0	0	0	0		0	2	0	1	68	1,78	21,46	0
1567	0	0	0	0	0		0	1	0	0	69	1,68	24,45	0
1568	0	0	0	0	0		0	1	0	0	69	1,68	24,45	0
1569	0	0	0	0	0		1	2	1	0	60	1,76	19,37	0
1570	0	0	0	0	0		0	1	0	0	70	1,6	27,34	0
1571	0	0	0	0	0		0	2	0	1	64	1,7	22,15	0
1572	0	0	0	0	0		1	3	1	1	57	1,67	20,44	0
1573	0	0	0	0	0		0	2	0	1	58	1,63	21,83	0
1574	0	0	0	0	0		0	1	0	0	70	1,6	27,34	0
1575	0	0	0	0	0		1	3	1	1	57	1,67	20,44	0
1576	0	0	0	0	0		0	2	0	1	64	1,7	22,15	0
1577	0	0	0	0	0		0	2	0	1	58	1,63	21,83	0
1578	0	0	0	0	0		0	1	0	0	92	1,78	29,04	0
1579	0	0	0	0	0		0	1	0	0	92	1,78	29,04	0
1580	0	0	0	0	0		0	1	0	0	95	1,7	32,87	0
1581	0	0	0	0	0		0	1	0	0	95	1,7	32,87	0
1582	0	0	0	0	0		1	5	2	2	68	1,68	24,09	0
1583	0	0	0	0	0		1	2	1	0	58	1,7	20,07	0

1584	0	0	0	0	0			1	2	1	0	58	1,7	20,07	0
1585	0	0	0	0	0			1	3	1	1	57	1,58	22,83	0
1586	0	0	0	0	0			1	5	2	2	68	1,68	24,09	0
1587	0	0	0	0	0			1	3	1	1	57	1,58	22,83	0
1588	0	0	0	0	0			0	1	0	0	64	1,52	27,7	0
1589	0	0	0	0	0			0	1	0	0	64	1,52	27,7	0
1590	0	0	0	0	0			0	1	0	0	66	1,79	20,6	0
1591	0	0	0	0	0			0	1	0	0	66	1,79	20,6	0
1592	0	0	0	0	0			0	1	0	0	68	1,68	24,09	0
1593	0	0	0	0	0			0	1	0	0	68	1,68	24,09	0
1594	0	0	0	0	0			1	2	1	0	56	1,6	21,88	0
1595	0	0	0	0	0			1	2	1	0	56	1,6	21,88	0
1596	0	0	0	0	0			1	2	1	0	61	1,7	21,11	0
1597	0	0	0	0	0			1	5	3	1	85	1,68	30,12	0
1598	0	0	0	0	0			1	2	1	0	49	1,58	19,63	0
1599	0	0	0	0	0			0	1	0	0	57	1,65	20,94	0
1600	0	0	0	0	0			0	1	0	0	57	1,65	20,94	0
1601	0	0	0	0	0			1	2	1	0	61	1,69	21,36	0
1602	0	0	0	0	0			1	2	1	0	61	1,69	21,36	0
1603	0	0	0	0	0			1	2	1	0	49	1,58	19,63	0
1604	0	0	0	0	0			1	8	4	3	114	1,75	37,22	0
1605	0	0	0	0	0			0	1	0	0	92	1,62	35,06	0
1606	0	0	0	0	0			0	1	0	0	81	1,68	28,7	0
1607	0	0	0	0	0			1	6	3	2	95	1,76	30,67	0
1608	0	0	0	0	0			0	1	0	0	92	1,62	35,06	0
1609	0	0	0	0	0			1	4	1	2	56	1,7	19,38	0
1610	0	1	0	0	0			1	2	1	0	72	1,62	27,43	0
1611	0	1	0	0	0			1	2	1	0	72	1,62	27,43	0
1612	0	0	0	0	0			1	5	1	3	62	1,58	24,84	0
1613	0	0	0	0	0			0	1	0	0	81	1,68	28,7	0
1614	0	0	0	0	0			1	4	1	2	56	1,7	19,38	0
1615	0	0	0	0	0			1	6	3	2	95	1,76	30,67	0
1616	0	0	0	0	0			1	5	1	3	62	1,58	24,84	0

1617	0	0	0	0	0		0	1	0	0	58	1,62	22,1	0
1618	0	0	0	0	0		0	1	0	0	58	1,62	22,1	0
1619	0	0	0	0	0		0	2	0	1	65	1,7	22,49	0
1620	0	0	0	0	0		0	2	0	1	65	1,7	22,49	0
1621	0	1	0	0	0		1	2	1	0	80	1,65	29,38	0
1622	0	1	0	0	0		1	2	1	0	80	1,65	29,38	0
1623	0	0	0	0	0		1	2	1	0	72	1,68	25,51	0
1624	0	0	0	0	0		0	1	0	0	55	1,7	19,03	0
1625	0	0	0	0	0		1	2	1	0	72	1,68	25,51	0
1626	0	0	0	0	0		1	2	1	0	67	1,67	24,02	0
1627	0	0	0	0	0		0	1	0	0	60	1,56	24,65	0
1628	0	0	0	0	0		1	2	1	0	67	1,67	24,02	0
1629	0	0	0	0	0		1	2	1	0	59	1,6	23,05	0
1630	0	0	0	0	0		1	2	1	0	59	1,6	23,05	0
1631	0	0	0	0	0		1	2	1	0	66	1,71	22,57	0
1632	0	0	0	0	0		1	3	1	1	67	1,76	21,63	0
1633	0	0	0	0	0		1	3	1	1	67	1,76	21,63	0
1634	0	0	0	0	0		0	1	0	0	56	1,68	19,84	0
1635	0	0	0	0	0		1	2	1	0	66	1,71	22,57	0
1636	0	0	0	0	0		0	1	0	0	56	1,68	19,84	0
1637	0	0	0	0	0		0	1	0	0	52	1,68	18,42	0
1638	0	0	0	0	0		0	3	0	2	66	1,57	26,78	0
1639	0	0	0	0	0		0	3	0	2	66	1,57	26,78	0
1640	0	0	0	0	0		0	1	0	0	60	1,66	21,77	0
1641	0	0	0	0	0		1	4	3	0	62	1,65	22,77	0
1642	0	0	0	0	0		0	1	0	0	60	1,66	21,77	0
1643	0	0	0	0	0		1	6	5	0	75	1,56	30,82	0
1644	0	1	0	0	0		1	3	2	0	90	1,64	33,46	0
1645	0	1	0	0	0		1	3	2	0	90	1,64	33,46	0
1646	0	0	0	0	0		1	6	5	0	75	1,56	30,82	0
1647	0	0	0	0	0		1	3	2	1	65	1,58	26,04	0
1648	0	0	0	0	0		1	3	2	1	65	1,58	26,04	0
1649	0	0	0	0	0		1	2	1	0	60	1,55	24,97	0

1650	0	0	0	0	0		1	2	1	0	60	1,55	24,97	0
1651	0	0	0	0	0		0	1	0	0	68	1,73	22,72	0
1652	0	0	0	0	0		0	1	0	0	68	1,73	22,72	0
1653	0	0	0	0	0		0	1	0	0	94	1,8	29,01	0
1654	0	0	0	0	0		0	1	0	0	70	1,72	23,66	0
1655	0	0	0	0	0		1	2	1	0	76	1,62	28,96	0
1656	0	0	0	0	0		1	2	1	0	67	1,74	22,13	0
1657	0	0	0	0	0		0	1	0	0	70	1,72	23,66	0
1658	0	0	0	0	0		1	2	1	0	76	1,62	28,96	0
1659	0	0	0	0	0		1	2	1	0	67	1,74	22,13	0
1660	0	0	0	0	0		0	1	0	0	94	1,8	29,01	0
1661	0	0	0	0	0		1	2	1	0	75	1,67	26,89	0
1662	0	0	0	0	0		1	3	1	1	70	1,78	22,09	0
1663	0	0	0	0	0		1	3	1	1	70	1,78	22,09	0
1664	0	0	0	0	0		1	2	1	0	97	1,7	33,56	0
1665	0	0	0	0	0		1	2	1	0	97	1,7	33,56	0
1666	0	0	0	0	0		1	2	1	0	74	1,76	23,89	0
1667	0	0	0	0	0		1	2	1	0	74	1,76	23,89	0
1668	0	0	0	0	0		0	1	0	0	68	1,78	21,46	0
1669	0	0	0	0	0		0	1	0	0	68	1,78	21,46	0
1670	0	0	0	0	0		0	4	0	3	59	1,74	19,49	0
1671	0	0	0	0	0		0	4	0	3	59	1,74	19,49	0
1672	0	0	0	0	0		0	1	0	0	59	1,55	24,56	0
1673	0	0	0	0	0		0	1	0	0	59	1,55	24,56	0
1674	0	0	0	0	0		0	2	0	1	88	1,63	33,12	0
1675	0	0	0	0	0		0	2	0	1	88	1,63	33,12	0
1676	0	0	0	0	0		0	1	0	0	57	1,73	19,05	0
1677	0	0	0	0	0		1	3	1	1	61	1,68	21,61	0
1678	0	0	0	0	0		1	3	1	1	61	1,68	21,61	0
1679	0	0	0	0	0		0	1	0	0	90	1,66	32,66	0
1680	0	0	0	0	0		0	1	0	0	90	1,66	32,66	0
1681	0	0	0	0	0		1	3	1	1	48	1,58	19,23	1
1682	0	0	0	0	0		1	3	1	1	48	1,58	19,23	1

1683	0	0	0	0	0			1	3	2	0	61	1,6	23,83	0
1684	0	0	0	0	0			1	3	2	0	61	1,6	23,83	0
1685	0	0	0	0	0			1	4	3	0	80	1,58	32,05	0
1686	0	0	0	0	0			1	4	3	0	80	1,58	32,05	0
1687	0	0	0	0	0			1	2	1	0	52	1,64	19,33	0
1688	0	0	0	0	0			1	2	1	0	52	1,64	19,33	0
1689	0	0	0	0	0			0	1	0	0	98	1,68	34,72	0
1690	0	0	0	0	0			0	1	0	0	98	1,68	34,72	0
1691	1	0	0	1	0			1	5	1	3	72	1,6	28,12	0
1692	0	0	0	1	0			1	5	1	3	72	1,6	28,12	0
1693	0	0	1	0	0			0	1	0	0	69	1,64	25,65	0
1694	0	0	1	0	0			0	1	0	0	69	1,64	25,65	0
1695	0	0	0	0	0			1	2	1	0	65	1,67	23,31	0
1696	0	0	0	0	0			1	2	1	0	65	1,67	23,31	0
1697	0	0	0	1	0			1	5	1	3	90	1,6	35,16	0
1698	0	1	0	0	0			1	2	1	0	58	1,68	20,55	0
1699	0	1	0	0	0			1	2	1	0	58	1,68	20,55	0
1700	0	0	0	0	0			1	2	1	0	63	1,64	23,42	0
1701	0	0	0	0	0			0	1	0	0	99	1,72	33,46	0
1702	0	0	0	0	0			0	1	0	0	59	1,62	22,48	0
1703	0	0	0	0	0			0	1	0	0	52	1,59	20,57	0
1704	0	0	0	0	0			0	2	0	1	99	1,7	34,26	0
1705	0	0	0	0	0			0	1	0	0	80	1,7	27,68	0
1706	0	0	0	0	0			0	1	0	0	64	1,69	22,41	0
1707	0	0	0	0	0			0	1	0	0	71	1,73	23,72	0
1708	0	0	0	0	0			0	1	0	0	67	1,64	24,91	0
1709	0	0	0	0	0			0	1	0	0	85	1,59	33,62	0
1710	0	0	0	0	0			0	1	0	0	45	1,7	15,57	0
1711	0	1	0	0	0			1	2	1	0	102	1,52	44,15	0
1712	0	0	0	0	0			0	1	0	0	79	1,75	25,8	0
1713	0	1	0	0	0			1	3	2	0	68	1,64	25,28	0
1714	0	0	0	0	0			0	1	0	0	85	1,67	30,48	0
1715	0	0	0	0	0			0	1	0	0	79	1,75	25,8	0

1716	0	0	0	0	0			0	1	0	0	86	1,74	28,41	0
1717	0	0	0	0	0			0	1	0	0	57	1,65	20,94	0
1718	0	0	0	0	0			0	1	0	0	75	1,75	24,49	0
1719	0	1	0	0	0			0	2	0	1	65	1,66	23,59	0
1720	0	0	0	0	0			0	1	0	0	78	1,65	28,65	0
1721	0	1	0	0	0			1	3	2	0	69	1,68	24,45	0
1722	0	0	0	0	0			1	3	1	1	78	1,5	34,67	0
1723	0	0	0	0	0			0	4	0	3	66	1,51	28,95	0
1724	0	1	0	0	0			0	1	0	0	80	1,73	26,73	0
1725	0	1	0	0	0			1	2	1	0	68	1,52	29,43	0
1726	0	0	0	0	0			0	2	0	1	64	1,68	22,68	0
1727	0	0	0	0	0			1	2	1	0		1,53		0
1728	0	1	0	0	0			1	4	2	1	81	1,74	26,75	0
1729	0	0	0	0	0			0	2	0	1	68	1,68	24,09	0
1730	0	1	0	0	0			1	2	1	0	60	1,6	23,44	0
1731	0	0	0	0	0			1	3	2	0	62	1,64	23,05	0
1732	0	1	0	0	0			1	5	3	1	64	1,67	22,95	0
1733	0	1	0	0	0			1	5	3	1	64	1,67	22,95	0
1734	0	1	0	0	0			1	2	1	0	50	1,53	21,36	0
1735	0	0	0	0	0			0	1	0	0	65	1,62	24,77	0
1736	0	1	0	1	0			1	3	2	0	68	1,6	26,56	0
1737	0	1	0	1	0			1	2	1	0	79	1,7	27,34	0
1738	0	1	0	1	0			1	2	1	0	79	1,7	27,34	0
1739	0	1	0	1	0			1	2	1	0	79	1,7	27,34	0
1740	0	0	0	0	0			0	1	0	0	83	1,71	28,38	0
1741	0	0	0	0	0			0	1	0	0	83	1,71	28,38	0
1742	0	0	0	0	0			0	1	0	0	83	1,71	28,38	0
1743	0	0	1	0	0			0	1	0	0	64	1,65	23,51	0
1744	0	0	0	0	0			1	3	2	0	150	1,7	51,9	0
1745	0	0	0	0	0			0	1	0	0	78	1,62	29,72	0
1746	0	0	0	0	0			0	1	0	0	70	1,72	23,66	0
1747	0	0	0	0	0			0	1	0	0	70	1,72	23,66	0
1748	0	0	0	0	0			1	2	1	0	52	1,67	18,65	0

1749	0	0	0	0	0			0	1	0	0	48	1,65	17,63	0
1750	0	0	1	0	0			0	2	0	1		1,55		0
1751	0	0	0	0	0			1	3	2	0	63	1,66	22,86	0
1752	0	0	0	0	0			1	3	2	0	63	1,66	22,86	0
1753	0	0	1	0	0	1		1	2	1	0	76	1,65	27,92	0
1754	0	0	0	0	0	1		0	2	0	1	60	1,66	21,77	0
1755	0	0	0	0	0	1		0	2	0	1	60	1,66	21,77	0
1756	0	1	1	0	0			0	1	0	0	85	1,65	31,22	0
1757	0	1	0	0	0			0	1	0	0	85	1,65	31,22	0
1758	0	0	0	0	0			1	2	1	0	76	1,66	27,58	0
1759	0	0	0	0	0			1	3	1	1	76	1,56	31,23	0
1760	0	0	0	0	0			0	1	0	0		1,66		0
1761	0	0	0	0	0			1	2	1	0	89	1,65	32,69	0
1762	0	0	0	0	0			0	2	0	1	55	1,76	17,76	0
1763	0	0	0	0	0			0	2	0	1	70	1,6	27,34	0
1764	0	0	0	0	0			0	1	0	0	120	1,75	39,18	0
1765	0	0	0	0	0			0	1	0	0	95	1,75	31,02	0
1766	0	0	0	0	0			0	1	0	0	67,5	1,67	24,20	0
1767	0	0	0	0	0			0	1	0	0	51,5	1,7	17,82	0
1768	0	0	0	0	0			0	1	0	0	75	1,6	29,30	0
1769	0	0	0	0	0			0	1	0	0	64	1,63	24,09	0
1770	0	0	0	0	0			1	2	1	0	70	1,71	23,94	0
1771	0	0	0	0	0			0	1	0	0	70	1,7	24,22	0
1772	0	0	0	0	0			0	1	0	0	96	1,52	41,55	0
1773	0	0	0	0	0			0	1	0	0	114	1,75	37,22	0
1774	0	0	0	0	0			0	1	0	0	65	1,63	24,46	0
1775	0	0	0	0	0			0	1	0	0	66	1,77	21,07	0
1776	0	0	0	0	0			0	1	0	0	49,5	1,55	20,60	0
1777	0	0	0	0	0			0	1	0	0	60	1,63	22,58	0
1778	0	0	0	0	0			0	4	0	3	73	1,56	30,00	0
1779	0	0	0	0	0			0	1	0	0	78	1,72	26,37	0
1780	0	0	0	0	0			1	3	1	1	82	1,7	28,37	1
1781	0	0	0	0	0			0	1	0	0	98	1,79	30,59	0

1782	1	0	0	0	0		0	2	0	1	72	1,65	26,45	0
1783	0	1	0	0	0		0	1	0	0	65	1,69	22,76	0
1784	0	0	0	0	0		1	3	1	1	78	1,8	24,07	0
1785	0	1	0	0	0		1	2	1	0	89	1,59	35,2	0
1786	0	1	0	0	0		1	2	1	0	89	1,59	35,2	0
1787	0	0	0	0	0		1	2	1	0	60	1,62	22,86	0
1788	0	0	0	0	0		0	2	0	1	76	1,63	28,6	0
1789	0	0	0	0	0		1	3	2	0	60	1,63	22,58	0
1790	0	0	0	0	0		0	1	0	0	60	1,71	20,52	0
1791	0	0	0	0	0		0	1	0	0	74	1,7	25,61	0
1792	0	0	0	0	0		0	1	0	0	59	1,61	22,76	0
1793	0	1	0	0	0		1	2	1	0	69	1,69	24,16	0
1794	0	0	0	0	0		1	3	1	1	71	1,67	25,46	0
1795	0	0	0	0	0		0	1	0	0	65	1,66	23,59	0
1796	0	0	0	0	0		1	2	1	0	88	1,68	31,18	0
1797	0	0	0	0	0		0	1	0	0	68	1,63	25,59	0
1798	0	0	0	0	0		0	1	0	0	49	1,58	19,63	0
1799	0	0	0	0	0		0	1	0	0	45	1,54	18,97	0
1800	0	0	0	0	0		0	1	0	0	52	1,67	18,65	0
1801	0	0	0	0	0		0	1	0	0	61	1,64	22,68	0
1802	0	0	0	0	0		0	1	0	0	55	1,55	22,89	0
1803	0	0	0	0	0		0	1	0	0	75	1,65	27,55	0
1804	0	0	0	0	0		0	1	0	0	63	1,7	21,8	0
1805	0	0	0	0	0		0	1	0	0	58	1,6	22,66	0
1806	0	0	0	0	0		0	1	0	0	74	1,72	25,01	0
1807	0	1	0	0	0		0	1	0	0	73	1,7	25,26	1
1808	0	0	0	0	0		0	1	0	0	55	1,52	23,81	0
1809	0	0	0	0	0		1	2	1	0	64	1,63	24,09	0
1810	0	0	0	0	0		0	1	0	0	55	1,58	22,03	0
1811	0	0	0	0	0		1	4	2	1	61	1,63	22,96	0
1812	0	0	0	0	0		0	1	0	0	103	1,7	35,64	0
1813	0	0	0	0	0		0	1	0	0	61	1,66	22,14	0
1814	0	0	0	0	0		0	1	0	0	67	1,64	24,91	0

1815	0	0	0	0	0		1	2	1	0	59	1,69	20,66	0
1816	0	0	0	0	0		0	1	0	0	40	1,5	17,78	0
1817	0	0	0	0	0		0	1	0	0	61	1,61	23,53	0
1818	0	0	0	0	0		0	3	0	2	50	1,66	18,14	0
1819	0	0	0	0	0		0	1	0	0	76	1,63	28,6	0
1820	0	0	0	0	0		0	1	0	0	90	1,69	31,51	0
1821	0	0	0	0	0		0	1	0	0	74	1,72	25,01	0
1822	0	0	0	0	0		0	2	0	1		1,7		0
1823	0	0	0	0	0		0	1	0	0	62	1,67	22,23	0
1824	0	0	0	0	0		0	1	0	0	57	1,56	23,42	0
1825	0	0	0	0	0		0	1	0	0	74	1,61	28,55	0
1826	0	0	0	0	0		0	2	0	1	77	1,63	28,98	0
1827	0	0	0	0	0		0	1	0	0	55	1,6	21,48	0
1828	0	0	0	0	0		0	1	0	0	103	1,76	33,25	0
1829	0	0	0	0	0		0	1	0	0	76	1,73	25,39	0
1830	0	0	0	0	0		1	2	1	0	63	1,63	23,71	0
1831	0	0	0	0	0		1	3	1	1	75	1,68	26,57	0
1832	1	1	0	0	0	2	0	1	0	0	64	1,64	23,8	0
1833	2	0	0	0	0	1	0	1	0	0	89	1,65	32,69	0
1834	2	0	0	0	0	1	0	1	0	0	89	1,65	32,69	0
1835	2	1	0	0	0	3	0	1	0	0	74	1,6	28,91	0
1836	0	0	0	0	0		0	2	0	1	55	1,6	21,48	0
1837	0	0	0	0	0		0	3	0	2	62	1,73	20,72	0
1838	0	0	0	0	0		0	1	0	0	68	1,68	24,09	0
1839	0	0	0	0	0		0	3	0	2	90	1,74	29,73	0
1840	0	0	0	0	0		0	3	0	2	90	1,74	29,73	0
1841	0	0	0	0	0		0	1	0	0	74	1,6	28,91	0
1842	0	0	0	0	0		1	4	3	0	65	1,65	23,88	0
1843	0	0	0	0	0		1	4	3	0	65	1,65	23,88	0
1844	0	0	0	0	0		0	1	0	0	66	1,58	26,44	0
1845	0	1	0	0	0		0	1	0	0	97	1,75	31,67	0
1846	0	0	0	0	0		1	3	1	1	85	1,75	27,76	0
1847	0	0	0	0	0		0	2	0	1	58	1,67	20,8	0

1848	0	1	0	0	0		1	4	2	1	70	1,76	22,6	0
1849	0	1	0	0	0		1	4	2	1	70	1,76	22,6	0
1850	0	1	0	0	0		1	2	1	0	59	1,62	22,48	0
1851	0	1	0	0	0		1	2	1	0	59	1,62	22,48	0
1852	0	0	0	0	0		1	2	1	0	76	1,63	28,6	0
1853	0	0	0	0	0		0	1	0	0	68	1,77	21,71	0
1854	0	0	0	0	0		0	1	0	0	52	1,53	22,21	0
1855	0	1	0	0	0		0	1	0	0	58	1,69	20,31	0
1856	0	0	0	0	0		0	1	0	0	51	1,58	20,43	0
1857	0	0	0	0	0		0	1	0	0	79	1,63	29,73	0
1858	1	0	0	0	0		0	1	0	0	64	1,78	20,2	0
1859	0	0	0	0	0		0	2	0	1	58	1,72	19,61	0
1860	0	0	0	0	0		0	2	0	1	58	1,72	19,61	0
1861	0	0	0	0	0		0	2	0	1	58	1,72	19,61	0
1862	0	0	0	0	0		0	2	0	1	53	1,68	18,78	0
1863	0	0	0	0	0		0	1	0	0	47	1,55	19,56	0
1864	0	0	0	0	0		1	2	1	0	108	1,7	37,37	0
1865	0	0	0	0	0		0	1	0	0		1,64		0
1866	0	0	0	0	0		1	3	2	0		1,62		0
1867	0	1	0	1	0		1	2	1	0	61	1,57	24,75	0
1868	0	0	0	0	0		0	1	0	0	58	1,64	21,56	0
1869	0	0	0	0	0		0	1	0	0	58	1,67	20,8	0
1870	0	0	0	0	0		0	1	0	0		1,65		0
1871	0	0	0	0	0		0	3	0	2		1,65		0
1872	0	0	0	0	0		1	2	1	0	82	1,75	26,78	0
1873	0	0	0	0	0		0	1	0	0	118	1,63	44,41	0
1874	0	0	0	0	0		0	10	0	0	50	1,62	19,05	0
1875	0	0	0	0	0		0	1	0	0	56	1,64	20,82	0
1876	0	0	0	0	0		0	1	0	0	56	1,64	20,82	0
1877	0	0	0	0	0		0	1	0	0	56	1,64	20,82	0
1878	0	0	0	0	0		0	2	0	1	132	1,7	45,67	0
1879	0	0	0	0	0		1	3	1	1	70	1,65	25,71	0
1880	0	0	0	0	0		0	1	0	0	55	1,68	19,49	0

1881	1	1	0	0	0	1	0	3	0	2	72	1,7	24,91	0
1882	0	0	1	0	0		1	2	1	0	113	1,72	38,2	0
1883	0	0	1	0	0		0	1	0	0	92	1,7	31,83	0
1884	0	0	1	0	0		0	1	0	0	102	1,71	34,88	0
1885	0	0	1	0	0		1	7	3	3	48	1,52	20,78	0
1886	2	0	1	0	0	1	0	1	0	0	70	1,7	24,22	0
1887	0	0	0	0	0		1	2	1	0	97	1,7	33,56	0
1888	1	1	1	0	0		1	2	1	0	66	1,58	26,44	0
1889	2	0	0	0	0	1	0	1	0	0	62	1,61	23,92	0
1890	2	0	0	0	0	1	0	1	0	0	62	1,61	23,92	0
1891	2	0	0	0	0	1	0	1	0	0	62	1,61	23,92	0
1892	0	0	1	0	0	1	0	1	0	0	127	1,7	43,94	0
1893	0	0	0	0	0	1	1	2	1	0	68	1,65	24,98	1
1894	0	0	0	0	0	6	1	2	1	0	53	1,65	19,47	0
1895	0	0	0	0	0	6	1	2	1	0	53	1,65	19,47	0
1896	0	0	0	0	0	6	1	2	1	0	53	1,65	19,47	0
1897	0	0	0	0	0	1	0	2	0	1	83	1,65	30,49	0
1898	0	0	0	0	0	1	0	2	0	1	83	1,65	30,49	0
1899	1	0	0	0	1	1	0	2	0	1	66	1,72	22,31	0
1900	1	0	0	0	1	1	0	2	0	1	66	1,72	22,31	0
1901	1	0	0	0	1	1	0	2	0	1	66	1,72	22,31	0
1902	2	0	0	0	0	1	0	1	0	0	64	1,65	23,51	0
1903	2	0	0	0	0	1	0	1	0	0	64	1,65	23,51	0
1904	2	0	0	0	0	1	0	1	0	0	64	1,65	23,51	0
1905	1	0	0	0	0	1	0	2	0	1	56,8	1,58	22,75	0
1906	2	0	0	0	0	2	0	1	0	0	63	1,67	22,59	0
1907	2	0	0	0	0	2	0	1	0	0	63	1,67	22,59	0
1908	2	0	0	0	0	2	0	1	0	0	63	1,67	22,59	0
1909	2	1	0	0	0	1	1	2	1	0	57	1,61	21,99	0
1910	2	1	0	0	0	1	1	2	1	0	57	1,61	21,99	0
1911	2	1	0	0	0	1	1	2	1	0	57	1,61	21,99	0
1912	1	1	0	0	0	1	1	2	1	0	65	1,69	22,76	0
1913	1	1	0	0	0	1	1	2	1	0	65	1,69	22,76	0

1914	1	1	0	0	0	1	1	2	1	0	65	1,69	22,76	0
1915	0	0	0	0	0	2	0	1	0	0	53	1,53	22,64	0
1916	1	0	0	0	0	2	1	5	3	1	60	1,65	22,04	0
1917	1	0	0	0	0	2	1	5	3	1	60	1,65	22,04	0
1918	1	0	0	0	0	2	1	5	3	1	60	1,65	22,04	0
1919	1	0	1	0	1	1	0	1	0	0	112	1,76	36,16	0
1920	2	1	0	0	0	1	0	2	0	1	58	1,63	21,83	0
1921	2	1	0	0	0	1	0	2	0	1	58	1,63	21,83	0
1922	0	0	1	0	1	3	0	1	0	0	80	1,73	26,73	0
1923	1	1	0	0	0	2	1	2	1	0	87	1,68	30,82	0
1924	1	1	0	0	0	2	1	2	1	0	87	1,68	30,82	0
1925	1	1	0	0	0	2	1	2	1	0	87	1,68	30,82	0
1926	1	0	0	0	0	1	0	1	0	0	99	1,72	33,46	0
1927	1	0	0	0	0	1	0	1	0	0	99	1,72	33,46	0
1928	2	0	0	0	1	1	0	1	0	0	76	1,76	24,54	0
1929	2	0	0	0	1	1	0	1	0	0	76	1,76	24,54	0
1930	1	0	0	0	0	6	1	4	2	1	69	1,72	23,32	1
1931	1	0	0	0	0	6	1	4	2	1	69	1,72	23,32	1
1932	1	0	0	0	0	6	1	4	2	1	69	1,72	23,32	1
1933	0	0	1	0	0	1	0	2	0	1	66	1,66	23,95	0
1934	2	1	0	1	0	2	1	3	2	0	72	1,7	24,91	0
1935	1	0	0	1	0	1	0	1	0	0	75	1,8	23,15	0
1936	0	0	1	0	0		0	1	0	0	101	1,7	34,95	0
1937	0	0	0	0	0		0	1	0	0	60	1,7	20,76	0
1938	2	0	0	0	0		0	1	0	0	78	1,5	34,67	0
1939	0	0	0	0	0		1	2	1	0	70	1,65	25,71	0
1940	0	1	0	0	0		1	2	1	0	60	1,65	22,04	0
1941	0	0	0	0	0		0	1	0	0	95	1,65	34,89	0
1942	0	0	0	0	0		0	1	0	0	60	1,64	22,31	0
1943	0	1	0	0	0		0	1	0	0	64,2	1,56	26,38	1
1944	0	0	0	0	0		0	2	0	1	57	1,57	23,12	0
1945	0	0	0	0	0		0	1	0	0		1,55		0
1946	0	0	0	0	0		0	1	0	0	78	1,58	31,24	0

1947	0	0	0	0	0		1	2	1	0	114,8	1,77	36,64	0
1948	0	0	0	0	0		1	5	3	1	69	1,55	28,72	0
1949	0	0	0	0	0		0	1	0	0	65	1,59	25,71	0
1950	0	0	0	0	0		0	1	0	0	67	1,65	24,61	0
1951	0	0	0	0	0		0	1	0	0	78	1,61	30,09	0
1952	0	0	0	0	0		0	1	0	0	64	1,63	24,09	0
1953	0	0	0	0	0		0	1	0	0	57	1,58	22,83	0
1954	0	0	0	0	0		0	1	0	0	59	1,6	23,05	0
1955	0	0	0	0	0		0	1	0	0	54,5	1,66	19,78	0
1956	0	0	0	0	0		0	1	0	0	70	1,73	23,39	0
1957	0	0	0	0	0		0	1	0	0	76	1,8	23,46	0
1958	0	0	0	0	0		0	2	0	1	59	1,7	20,42	0
1959	0	0	0	0	0		0	1	0	0	77	1,65	28,28	0
1960	0	0	0	0	0		0	1	0	0	127	1,68	45,00	0
1961	0	0	0	0	0		0	2	0	1	70	1,7	24,22	0
1962	0	0	0	0	0		0	1	0	0	65	1,52	28,13	0
1963	0	0	0	0	0		0	1	0	0	100	1,63	37,64	0
1964	0	0	0	0	0		0	1	0	0	68	1,65	24,98	0
1965	0	0	0	0	0		0	1	0	0	49,5	1,53	21,15	0
1966	0	0	0	0	0		1	2	1	0	76	1,5	33,78	0
1967	0	0	0	0	0		1	6	1	4		1,72		0
1968	0	0	0	0	0		0	1	0	0	60	1,55	24,97	0
1969	0	0	0	0	0		0	2	0	1	88	1,65	32,32	0
1970	0	0	0	0	0		1	2	1	0	76	1,68	26,93	0
1971	0	0	0	0	0		0	1	0	0	65	1,67	23,31	0
1972	0	0	0	0	0		1	3	2	0	115	1,75	37,55	0
1973	0	0	0	0	0		0	1	0	0	112	1,76	36,16	0
1974	0	0	0	0	0		0	4	0	3	111	1,68	39,33	0
1975	0	0	0	0	0		0	1	0	0	76	1,72	25,69	0
1976	0	0	0	0	0		1	3	2	0	68	1,57	27,59	0
1977	0	0	0	0	0		0	1	0	0	69	1,69	24,16	0
1978	0	1	0	0	0		1	5	3	1	95	1,52	41,12	0
1979	0	0	0	0	0		1	3	2	0	64	1,64	23,80	0

1980	0	0	0	0	0		0	1	0	0	75	1,72	25,35	0
1981	0	0	0	0	0		0	1	0	0	95	1,75	31,02	0
1982	0	0	0	0	0		0	1	0	0	80,5	1,55	33,51	0
1983	0	1	0	0	0		1	4	1	2	71	1,68	25,16	0
1984	0	0	0	0	0		1	2	1	0	62	1,7	21,45	0
1985	0	0	0	0	0		0	1	0	0	69	1,7	23,88	0
1986	0	1	0	0	0		1	5	2	2	53	1,57	21,50	0
1987	0	0	0	0	0		0	1	0	0	75	1,68	26,57	0
1988	0	0	0	0	0		0	1	0	0	66	1,7	22,84	0
1989	0	0	0	0	0		0	1	0	0	73	1,65	26,81	0
1990	0	0	0	0	0		0	1	0	0	73	1,65	26,81	0
1991	0	0	0	0	0		0	2	0	1	78	1,63	29,36	0
1992	0	0	0	0	0		0	2	0	1	78	1,63	29,36	0
1993	0	0	0	0	0		0	1	0	0	62	1,66	22,5	0
1994	0	0	0	0	0		0	1	0	0	62	1,66	22,5	0
1995	0	0	0	0	0		0	1	0	0	83	1,65	30,49	0
1996	0	0	0	0	0		0	1	0	0	83	1,65	30,49	0
1997	0	0	0	0	0		0	1	0	0	64	1,68	22,68	0
1998	0	0	0	0	0		1	2	1	0	89	1,69	31,16	0
1999	0	0	0	0	0		0	1	0	0	58	1,56	23,83	0
2000	0	0	0	0	0		0	1	0	0	58	1,56	23,83	0
2001	0	0	0	0	0		0	1	0	0	66	1,77	21,07	0
2002	0	0	0	0	0		0	1	0	0	66	1,77	21,07	0
2003	0	0	0	0	0		0	1	0	0	106	1,68	37,56	0
2004	0	0	0	0	0		0	1	0	0	106	1,68	37,56	0
2005	0	0	0	0	0		0	1	0	0	55	1,71	18,81	0
2006	0	0	0	0	0		0	1	0	0	43	1,63	16,18	0
2007	0	1	0	0	0		1	2	1	0	85	1,68	30,12	0
2008	0	1	0	0	0		1	2	1	0	85	1,68	30,12	0
2009	0	0	0	0	0		0	1	0	0	47	1,58	18,83	0
2010	0	0	0	0	0		0	3	0	2	63	1,68	22,32	0
2011	0	0	0	0	0		0	1	0	0	49	1,62	18,67	0
2012	0	0	0	0	0		0	2	0	1	99	1,76	31,96	0

2013	0	0	0	0	0			1	2	1	0	69	1,56	28,35	0
2014	0	0	0	0	0			0	1	0	0	53	1,65	19,47	0
2015	0	0	0	0	0			0	1	0	0	95	1,74	31,38	0
2016	0	0	0	0	0			1	4	2	1	71	1,59	28,08	0
2017	0	0	0	0	0			0	1	0	0	72	1,59	28,48	0
2018	0	0	0	0	0			0	1	0	0	51	1,6	19,92	0
2019	0	0	0	0	0			0	1	0	0	70	1,68	24,8	0
2020	0	0	0	0	0			0	1	0	0	92	1,63	34,63	0
2021	0	1	0	0	0			1	2	1	0	61	1,64	22,68	0
2022	0	0	0	0	0			0	1	0	0	51	1,54	21,5	0
2023	0	0	0	0	0			0	1	0	0	45	1,57	18,26	0
2024	0	0	0	0	0			0	1	0	0		1,65		0
2025	0	1	0	0	0			1	3	1	1	70	1,64	26,03	0
2026	0	0	0	0	0			1	2	1	0	62	1,64	23,05	0
2027	0	0	0	0	0			0	1	0	0	90	1,65	33,06	0
2028	0	0	0	0	0			0	1	0	0	72	1,68	25,51	0
2029	0	0	0	0	0			1	2	1	0	66	1,65	24,24	0
2030	0	0	0	0	0			0	1	0	0	78	1,6	30,47	0
2031	0	0	0	0	0			1	2	1	0	82	1,7	28,37	0
2032	0	0	0	0	0			0	1	0	0	74	1,68	26,22	0
2033	0	0	0	0	0			0	2	0	1	65	1,55	27,06	0
2034	0	1	0	0	0			1	3	1	1	67	1,8	20,68	0
2035	0	0	0	0	0			1	5	1	3	90	1,58	36,05	0
2036	0	1	0	0	0			1	2	1	0	131	1,75	42,78	0
2037	0	1	0	0	0			1	2	1	0	131	1,75	42,78	0
2038	0	0	0	0	0			0	1	0	0	51	1,6	19,92	0
2039	0	0	0	0	0			0	1	0	0	66	1,68	23,38	0
2040	0	0	0	0	0			0	1	0	0	91	1,69	31,86	0
2041	0	1	0	0	0			0	1	0	0	101	1,73	33,75	0
2042	0	0	0	0	0			0	1	0	0	95	1,75	31,02	0
2043	0	0	0	0	0			1	2	1	0	83	1,76	26,79	0
2044	0	0	0	0	0			0	1	0	0	79	1,72	26,7	0
2045	0	0	0	0	0			0	1	0	0	52	1,64	19,33	0

2046	0	0	0	0	0		0	2	0	1	90	1,78	28,41	0
2047	0	0	0	0	0		0	1	0	0	87	1,72	29,41	0
2048	0	0	0	0	0		0	1	0	0	77	1,61	29,71	0
2049	0	0	0	0	0		0	2	0	1	90	1,78	28,41	0
2050	0	0	0	0	0		0	1	0	0	115	1,86	33,24	0
2051	0	0	0	0	0		0	1	0	0	112	1,75	36,57	0
2052	0	0	0	0	0		0	1	0	0	72	1,7	24,91	0
2053	0	0	0	0	0		0	2	0	1	92	1,68	32,6	0
2054	0	0	0	0	0		0	2	0	1	92	1,68	32,6	0
2055	0	0	0	0	0		0	3	0	2		1,57		0
2056	0	0	0	0	0		1	2	1	0	143	1,67	51,27	0
2057	0	0	0	0	0		1	2	1	0	88	1,56	36,16	0
2058	0	1	0	0	0	1	0	2	0	1	56	1,65	20,57	0
2059	0	1	0	0	0		1	3	2	0	66	1,67	23,67	0
2060	0	1	0	0	0		1	3	2	0	66	1,67	23,67	0
2061	0	1	0	0	0		1	3	2	0	66	1,67	23,67	0
2062	0	0	0	0	0		1	2	1	0	65	1,7	22,49	1
2063	0	0	0	0	0		1	2	1	0	70	1,84	20,68	0
2064	0	0	0	0	0		1	4	3	0	65	1,65	23,88	0
2065	0	0	0	0	0		0	1	0	0	65	1,68	23,03	0
2066	0	0	0	0	0		1	3	1	1	62	1,58	24,84	0
2067	0	0	0	0	0		0	1	0	0	68	1,56	27,94	0
2068	0	0	0	0	0		0	1	0	0	82	1,72	27,72	0
2069	0	0	0	0	0		0	1	0	0	58	1,6	22,66	0
2070	0	0	1	0	0		1	3	2	0	84	1,6	32,81	0
2071	0	1	0	1	0		1	2	1	0	62	1,51	27,19	0
2072	0	0	0	0	0		1	2	1	0	58	1,69	20,31	0
2073	0	0	0	0	0		1	2	1	0	58	1,69	20,31	0
2074	0	0	0	0	0		0	1	0	0	86	1,81	26,25	0
2075	0	1	1	0	0		1	2	1	0	92	1,7	31,83	0
2076	0	0	1	0	0		1	2	1	0	80	1,63	30,11	0
2077	0	0	1	1	0		1	6	2	3	55	1,43	26,90	0
2078	0	0	1	0	0		0	1	0	0	65	1,68	23,03	0

2079	0	0	1	0	0		1	4	2	1	84	1,61	32,41	0
2080	0	0	1	0	0		1	3	2	0	94	1,64	34,95	0
2081	0	0	1	0	0		1	3	1	1	100	1,7	34,6	0
2082	0	0	1	0	0		0	1	0	0	68	1,78	21,46	0
2083	0	1	0	0	1	1	1	2	1	0	62	1,63	23,34	0
2084	0	1	0	0	1	1	1	2	1	0	62	1,63	23,34	0
2085	0	1	0	0	1	1	1	2	1	0	62	1,63	23,34	0
2086	2	0	0	0	0	1	0	1	0	0	87	1,83	25,98	0
2087	2	0	0	1	1	1	1	2	1	0	57	1,68	20,2	1
2088	2	0	0	1	1	1	1	2	1	0	57	1,68	20,2	1
2089	2	0	0	1	1	1	1	2	1	0	57	1,68	20,2	1
2090	2	0	0	0	0	1	0	1	0	0	58	1,62	22,1	0
2091	2	0	0	0	0	1	0	1	0	0	58	1,62	22,1	0
2092	0	0	0	0	1	1	0	1	0	0	54	1,64	20,08	0
2093	0	0	0	0	0		1	3	2	0	55	1,71	18,81	0
2094	0	0	0	0	0		1	3	2	0	55	1,71	18,81	0
2095	0	0	0	0	0		1	3	2	0	55	1,71	18,81	0
2096	0	0	0	0	0	1	0	1	0	0	110	1,72	37,18	0
2097	0	0	0	0	0	1	0	1	0	0	110	1,72	37,18	0
2098	2	1	0	0	0	1	1	2	1	0	62	1,62	23,62	0
2099	2	1	0	0	0	1	1	2	1	0	62	1,62	23,62	0
2100	1	0	0	0	0	1	1	3	1	1	84	1,68	29,76	0
2101	1	0	0	0	0	1	1	3	1	1	84	1,68	29,76	0
2102	1	0	0	0	0	1	1	3	1	1	84	1,68	29,76	0
2103	2	0	0	0	1	1	0	1	0	0	62	1,5	27,56	0
2104	curretage , mka	1	0	0	0	1	0	2	0	1	66	1,68	23,38	0
2105	0	1	0	0	0	1	1	2	1	0	64	1,71	21,89	0
2106	1	1	0	0	1	1	1	3	1	1	64	1,66	23,23	0
2107	1	1	0	0	1	1	1	3	1	1	64	1,66	23,23	0
2108	1	1	0	0	1	1	1	3	1	1	64	1,66	23,23	0
2109	0	1	0	0	0	1	1	2	1	0	84	1,69	29,41	0
2110	2	0	1	0	1	1	0	1	0	0	70,5	1,76	22,76	0
2111	0	0	0	0	0	1	0	1	0	0	68	1,69	23,81	0

2112	0	0	0	0	0	1	0	1	0	0	68	1,69	23,81	0
2113	1	1	1	0	0	6	1	2	1	0	79	1,5	35,11	0
2114	1	1	1	0	0	6	1	2	1	0	79	1,5	35,11	0
2115	1	1	1	0	0	6	1	2	1	0	79	1,5	35,11	0
2116	1	0	0	0	1	6	0	1	0	0	55	1,55	22,89	0
2117	0	0	0	0	1	6	0	1	0	0	52	1,63	19,57	0
2118	0	0	0	0	0	6	0	1	0	0	52	1,63	19,57	0
2119	1	0	0	0	0	1	0	2	0	1	63	1,59	24,92	0
2120	1	0	0	0	0	1	0	2	0	1	63	1,59	24,92	0
2121	2	1	1	0	0	1	1	2	1	0	113	1,67	40,52	0
2122	2	1	1	0	0	1	1	2	1	0	113	1,67	40,52	0
2123	1	0	1	0	0	1	0	1	0	0	60	1,67	21,51	0
2124	1	0	1	0	0	1	0	1	0	0	60	1,67	21,51	0
2125	1	0	1	0	0	1	0	1	0	0	60	1,67	21,51	0
2126	1	0	1	0	0	3	0	1	0	0	60	1,54	25,3	0
2127	1	0	1	0	0	3	0	1	0	0	60	1,54	25,3	0
2128	0	0	0	0	1	1	0	1	0	0	54	1,6	21,09	0
2129	0	0	0	0	0	1	0	1	0	0	69	1,63	25,97	0
2130	0	0	0	0	0	1	0	1	0	0	69	1,63	25,97	0
2131	2	0	0	0	1	1	0	1	0	0	66	1,48	30,13	0
2132	2	0	0	0	1	1	0	1	0	0	66	1,48	30,13	0
2133	1	1	0	0	1	1	1	3	1	1	68	1,7	23,53	0
2134	1	1	0	0	1	1	1	3	1	1	68	1,7	23,53	0
2135	1	1	0	0	1	1	1	3	1	1	68	1,7	23,53	0
2136	1	0	0	0	1	1	0	1	0	0		1,6		0
2137	2	0	0	0	0	1	0	1	0	0	56	1,61	21,6	0
2138	2	0	0	0	0	1	0	1	0	0	56	1,61	21,6	0
2139	0	0	0	0	0	1	0	1	0	0	63	1,67	22,59	0
2140	0	0	0	0	0	1	0	1	0	0	63	1,67	22,59	0
2141	0	1	0	0	0	3	1	2	1	0	82	1,67	29,4	0
2142	0	1	0	0	0	3	1	2	1	0	82	1,67	29,4	0
2143	2	0	1	1	1	1	0	1	0	0	109,5	1,78	34,56	0
2144	1	1	0	0	0	1	0	1	0	0	67	1,68	23,74	0

2145	1	1	0	0	0	1	0	1	0	0	67	1,68	23,74	0
2146	0	0	0	0	0		0	2	0	1	56	1,58	22,43	0
2147	0	0	0	0	0		0	1	0	0	57	1,54	24,03	0
2148	0	0	0	0	0		1	6	2	3	55	1,43	26,90	1
2149	0	0	0	0	0		1	9	3	5	83	1,56	34,11	0
2150	0	0	0	0	0		1	5	1	3	60	1,6	23,44	0
2151	0	0	0	0	0		0	1	0	0	56,7	1,58	22,71	0
2152	0	0	0	0	0		0	1	0	0	68	1,66	24,68	0
2153	0	0	0	0	0		0	1	0	0	54	1,64	20,08	0
2154	0	0	0	0	0		1	2	1	0	50	1,65	18,37	0
2155	0	0	0	0	0		0	1	0	0	54	1,63	20,32	0
2156	0	0	0	0	0		0	1	0	0	78	1,7	26,99	0
2157	0	0	0	0	0		0	1	0	0	65	1,68	23,03	0
2158	0	1	0	0	0		0	1	0	0		1,65		0
2159	0	0	0	0	0		0	1	0	0	105	1,5	46,67	0
2160	0	0	0	0	0		0	1	0	0	64	1,54	26,99	0
2161	0	0	0	0	0		0	1	0	0	66	1,69	23,11	0
2162	0	0	0	0	0		0	1	0	0	77	1,6	30,08	0
2163	0	0	0	0	0		1	3	2	0	90	1,74	29,73	0
2164	0	0	0	0	0		0	1	0	0	63	1,7	21,80	0
2165	0	0	0	0	0		1	2	1	0	50	1,67	17,93	0
2166	0	0	0	0	0		1	2	1	0	75	1,6	29,30	0
2167	0	0	0	0	0		0	1	0	0	90	1,8	27,78	0
2168	0	0	0	0	0		0	2	0	1		1,7		0
2169	0	0	0	0	0		1	3	2	0	68	1,7	23,53	0
2170	0	0	0	0	0		0	1	0	0	58	1,55	24,14	0
2171	0	0	0	0	0		0	1	0	0	65	1,67	23,31	0
2172	0	0	0	0	0		0	1	0	0	78	1,67	27,97	0
2173	0	0	0	0	0		0	1	0	0	61	1,67	21,87	0
2174	0	0	0	0	0		0	1	0	0		1,72		0
2175	0	0	0	0	0		1	3	2	0	100	1,73	33,41	0
2176	0	0	0	0	0		0	1	0	0	70,5	1,76	22,76	0
2177	0	1	0	0	0		1	2	1	0	86	1,72	29,07	0

2178	0	0	0	0	0		0	2	0	1	92	1,74	30,39	0
2179	0	0	0	0	0		0	1	0	0	91	1,68	32,24	0
2180	0	0	0	0	0		0	1	0	0	73,3	1,7	25,36	0
2181	0	0	0	0	0		0	1	0	0	58	1,7	20,07	0
2182	0	0	0	0	0		0	1	0	0	58	1,7	20,07	0
2183	0	0	0	0	1		1	2	1	0	53	1,63	19,95	0
2184	0	0	0	0	1		1	2	1	0	53	1,63	19,95	0
2185	0	1	0	0	0		1	2	1	0	57	1,72	19,27	0
2186	0	1	0	0	0		1	2	1	0	57	1,72	19,27	0
2187	0	0	0	0	0		0	1	0	0	80	1,75	26,12	0
2188	0	0	0	0	0		0	1	0	0	80	1,75	26,12	0
2189	0	0	0	0	0		0	1	0	0	93	1,69	32,56	0
2190	0	0	0	0	0		0	1	0	0	93	1,69	32,56	0
2191	0	0	0	0	0		1	3	1	1	82	1,68	29,05	0
2192	0	0	0	0	0		1	3	1	1	82	1,68	29,05	0
2193	0	0	0	0	0		1	4	3	0	77	1,7	26,64	0
2194	0	0	0	0	0		1	4	3	0	77	1,7	26,64	0
2195	0	1	0	0	0		1	3	1	1	107	1,64	39,78	0
2196	0	0	0	0	0		0	1	0	0	83	1,7	28,72	0
2197	0	0	0	0	0		0	1	0	0	97	1,6	37,89	0
2198	0	0	0	0	0		0	1	0	0	97	1,6	37,89	0
2199	0	0	0	0	0		0	1	0	0	60	1,7	20,76	0
2200	0	0	0	0	0		0	1	0	0	60	1,7	20,76	0
2201	0	0	0	0	0		1	4	2	1		1,55		0
2202	0	1	0	0	0		1	3	1	1	66	1,56	27,12	1
2203	0	1	0	0	0		1	3	1	1	66	1,56	27,12	1
2204	0	0	0	0	0		1	2	1	0	70	1,73	23,39	0
2205	0	0	0	0	0		0	1	0	0	68	1,65	24,98	0
2206	0	0	0	0	0		1	2	1	0	62	1,58	24,84	0
2207	0	0	1	0	0		1	4	2	1	82	1,65	30,12	0
2208	0	1	0	0	0		1	2	1	0	78	1,78	24,62	0
2209	0	1	0	0	0		1	2	1	0	78	1,78	24,62	0
2210	0	0	1	0	0		0	2	0	1	92	1,7	31,83	0

2211	1	0	0	0	1	1	0	2	0	1	72	1,7	24,91	0
2212	1	0	0	0	0		0	1	0	0	58	1,55	24,14	0
2213	1	0	0	0	0		0	1	0	0	58	1,55	24,14	0
2214	1	0	0	0	0		0	1	0	0	58	1,55	24,14	0
2215	1	1	0	0	1	1	1	6	4	1	69	1,58	27,64	0
2216	1	1	0	0	1	1	1	6	4	1	69	1,58	27,64	0
2217	1	0	1	0	1	1	0	1	0	0	83	1,63	31,24	0
2218	1	0	1	0	1	1	0	1	0	0	83	1,63	31,24	0
2219	1	0	1	0	1	1	0	1	0	0	83	1,63	31,24	0
2220	1	0	0	0	0	3	0	1	0	0	65	1,69	22,76	0
2221	1	0	0	0	0	3	0	1	0	0	65	1,69	22,76	0
2222	1	0	0	0	1	1	1	2	1	0	90	1,81	27,47	0
2223	1	0	0	0	0	1	0	3	0	2	50	1,67	17,93	0
2224	1	0	0	0	0	1	0	3	0	2	50	1,67	17,93	0
2225	1	0	0	0	0	1	0	3	0	2	50	1,67	17,93	0
2226	0	1	0	0	0	1	0	1	0	0	60	1,6	23,44	0
2227	0	1	0	0	0	1	0	1	0	0	60	1,6	23,44	0
2228	0	1	0	0	0	1	0	1	0	0	60	1,6	23,44	0
2229	0	1	0	0	0		0	1	0	0	65	1,57	26,37	0
2230	0	1	0	0	0		0	1	0	0	65	1,57	26,37	0
2231	0	0	0	0	0	3	0	1	0	0	80	1,65	29,38	0
2232	0	0	0	0	0	3	0	1	0	0	80	1,65	29,38	0
2233	0	0	0	0	0	1	1	2	1	0	62	1,67	22,23	0
2234	1	0	0	0	0	2	1	2	1	0	63	1,68	22,32	0
2235	1	0	0	0	0	2	1	2	1	0	63	1,68	22,32	0
2236	0	0	0	0	0	1	0	1	0	0	96	1,73	32,08	0
2237	0	0	0	0	0	1	0	1	0	0	96	1,73	32,08	0
2238	0	0	0	1	0	1	0	1	0	0	82	1,7	28,37	0
2239	0	0	0	1	0	1	0	1	0	0	82	1,7	28,37	0
2240	0	0	0	1	0	1	0	1	0	0	82	1,7	28,37	0
2241	2	1	1	0	1	1	1	5	2	2	90	1,64	33,46	0
2242	2	1	1	0	1	1	1	5	2	2	90	1,64	33,46	0
2243	1	0	1	0	0	1	1	4	1	2	98	1,6	38,28	0

2244	1	0	1	0	0	1	0	1	0	0	79	1,73	26,4	0
2245	1	0	1	0	0	1	0	1	0	0	79	1,73	26,4	0
2246	1	0	1	0	1	2	1	2	1	0	90	1,6	35,16	0
2247	1	0	1	0	1	2	1	2	1	0	90	1,6	35,16	0
2248	1	0	1	0	1	2	1	2	1	0	90	1,6	35,16	0
2249	1	1	1	1	0	2	1	4	2	1	60	1,77	19,15	1
2250	1	0	0	1	1	3	0	1	0	0	104	1,6	40,63	0
2251	0	1	0	0	0	1	1	3	1	1	61	1,6	23,83	0
2252	1	1	0	0	0	1	1	3	2	0	60	1,7	20,76	0
2253	1	0	0	0	0		0	1	0	0	99	1,71	33,86	0
2254	0	1	0	0	0	1	1	2	1	0	62	1,6	24,22	0
2255	0	1	0	0	0	1	1	2	1	0	62	1,6	24,22	0
2256	0	1	0	0	0	1	1	2	1	0	62	1,6	24,22	0
2257	1	1	0	0	0	1	1	3	2	0	73,8	1,65	27,11	0
2258	0	1	0	0	0		1	3	2	0	60	1,7	20,76	0
2259	0	1	0	0	0		1	3	2	0	68	1,72	22,99	0
2260	1	0	0	0	0		0	1	0	0		1,58		0
2261	0	0	1	0	0		0	1	0	0	68	1,72	22,99	0
2262	0	0	1	0	0		0	1	0	0	68	1,72	22,99	0
2263	0	0	0	0	0		0	1	0	0	58	1,71	19,84	0
2264	0	0	0	0	0		0	1	0	0	58	1,71	19,84	0
2265	0	1	0	0	0		0	1	0	0	69	1,6	26,95	0
2266	0	0	0	0	0		1	2	1	0	66	1,65	24,24	0
2267	0	0	0	0	0		1	3	1	1	63	1,7	21,8	0
2268	0	0	0	0	0		1	3	1	1	71	1,75	23,18	0
2269	0	0	0	0	0		0	1	0	1	80	1,67	28,69	0
2270	0	0	0	0	0		1	3	2	0	47	1,56	19,31	0
2271	0	0	0	0	0		0	1	0	0	99	1,72	33,46	0
2272	0	0	0	0	0		0	4	0	3	50	1,64	18,59	0
2273	0	0	0	0	0		0	1	0	0	94	1,72	31,77	0
2274	0	0	0	0	0		1	3	1	1	50	1,53	21,36	0
2275	0	0	0	0	0		0	1	0	0	53	1,62	20,2	0
2276	0	0	0	0	0		0	1	0	0	53	1,62	20,2	0

2277	2	0	0	0	0	1	0	4	0	3	64	1,61	24,69	0
2278	2	0	0	0	0	1	0	4	0	3	64	1,61	24,69	0
2279	0	0	0	0	0	1	1	3	1	1	50	1,63	18,82	0
2280	0	0	0	0	0	1	1	3	1	1	50	1,63	18,82	0
2281	1	0	0	0	0	1	0	1	0	0	72,5	1,65	26,63	0
2282	0	1	1	0	1	1	1	3	2	0	59	1,62	22,48	0
2283	0	0	0	0	0	1	0	3	0	2	52	1,57	21,1	0
2284	0	0	0	0	0	1	0	3	0	2	52	1,57	21,1	0
2285	1	0	0	0	0	1	0	2	0	1	62	1,69	21,71	0
2286	1	0	0	0	0	1	0	2	0	1	62	1,69	21,71	0
2287	0	0	0	0	1	1	1	2	1	0	60	1,67	21,51	0
2288	0	0	0	0	1	1	1	2	1	0	60	1,67	21,51	0
2289	1	0	0	0	0	1	0	1	0	0	63	1,56	25,89	0
2290	1	0	0	0	0	4	0	4	0	3	55	1,52	23,81	0
2291	1	0	0	0	0	4	0	4	0	3	55	1,52	23,81	0
2292	2	0	0	0	0	3	1	3	1	2	52	1,6	20,31	0
2293	2	0	0	0	0	3	1	3	1	2	52	1,6	20,31	0
2294	2	0	0	0	0	3	0	1	0	0	83	1,48	37,89	0
2295	2	0	0	0	0	3	0	1	0	0	83	1,48	37,89	0
2296	2	0	0	0	0	1	1	2	1	0	63	1,6	24,61	0
2297	2	0	0	0	0	1	1	2	1	0	63	1,6	24,61	0
2298	1	0	0	0	1	1	1	3	1	1	59	1,64	21,94	0
2299	1	0	0	0	1	1	1	3	1	1	59	1,64	21,94	0
2300	1	1	1	0	0	1	1	2	1	0	64	1,62	24,39	0
2301	1	1	1	0	0	1	1	2	1	0	64	1,62	24,39	0
2302	1	0	0	0	0	1	0	2	0	1	72	1,7	24,91	0
2303	1	0	0	0	0	1	0	2	0	1	72	1,7	24,91	0
2304	0	0	0	0	0	2	1	3	2	0	77	1,61	29,71	0
2305	0	0	0	0	0	2	1	3	2	0	77	1,61	29,71	0
2306	2	0	0	0	1	1	0	3	0	2	49	1,63	18,44	0
2307	2	0	0	0	1	1	0	3	0	2	49	1,63	18,44	0
2308	1	0	0	0	1	1	0	1	0	0	61	1,64	22,68	0
2309	1	1	1	0	0	3	1	2	1	0	52	1,63	19,57	0

2310	1	1	1	0	0	3	1	2	1	0	52	1,63	19,57	0
2311	0	0	0	0	0	1	1	2	1	0	51	1,58	20,43	0
2312	0	0	0	0	0	1	1	2	1	0	51	1,58	20,43	0
2313	0	0	0	0	0	1	1	4	2	1	60	1,7	20,76	0
2314	0	0	0	0	0	1	1	4	2	1	60	1,7	20,76	0
2315	2	0	0	0	0	3	1	4	1	2	85	1,62	32,39	0
2316	2	0	0	0	0	3	1	4	1	2	85	1,62	32,39	0
2317	2	0	0	0	0	3	1	4	1	2	85	1,62	32,39	0
2318	1	0	1	0	1	1	0	1	0	0	123	1,66	44,64	0
2319	0	0	0	0	0		0	1	0	0	79	1,62	30,1	0
2320	0	0	0	0	0		0	1	0	0	79	1,62	30,1	0
2321	0	0	0	0	0		1	2	1	0	55	1,7	19,03	0
2322	0	0	0	0	0		1	3	2	0	63	1,63	23,71	0
2323	0	0	0	0	0		1	3	2	0	63	1,63	23,71	0
2324	0	0	0	0	0		1	2	1	0	55	1,7	19,03	0
2325	0	0	0	0	0		0	1	0	0	64	1,66	23,23	0
2326	0	0	0	0	0		0	1	0	0	75	1,58	30,04	0
2327	0	0	0	0	0		0	1	0	0	68	1,65	24,98	0
2328	0	0	0	0	0		0	1	0	0	66	1,68	23,38	0
2329	0	0	0	0	0		0	3	0	2	50	1,55	20,81	0
2330	0	0	0	0	0		0	1	0	0	58	1,64	21,56	0
2331	0	0	0	0	0		0	1	0	0	49	1,62	18,67	0
2332	2	0	0	0	1	1	1	3	1	1	69	1,69	24,16	0
2333	2	0	0	0	1	1	1	3	1	1	69	1,69	24,16	0
2334	0	0	1	0	0		0	3	0	2	48	1,64	17,85	0
2335	2	0	0	0	1	1	1	2	1	0	67	1,52	29	0
2336	2	0	0	0	1	1	1	2	1	0	67	1,52	29	0
2337	0	0	0	0	0	1	0	2	0	1	46	1,6	17,97	0
2338	2	0	0	0	0	6	1	2	1	0	50	1,5	22,22	0
2339	2	0	0	0	0	6	1	2	1	0	50	1,5	22,22	0
2340	2	0	0	0	1	3	1	2	1	0	54	1,63	20,32	0
2341	2	0	0	0	1	3	1	2	1	0	54	1,63	20,32	0
2342	2	0	0	0	1	1	0	1	0	0	56	1,66	20,32	0

2343	1	0	1	0	0	1	0	1	0	0	71	1,56	29,17	0
2344	0	0	0	0	0	3	1	2	1	0	71	1,63	26,72	0
2345	0	0	0	0	0	2	0	1	0	0	62	1,69	21,71	0
2346	0	0	0	0	0	3	1	2	1	0	71	1,63	26,72	0
2347	0	0	0	0	1	1	1	2	1	0	65	1,63	24,46	0
2348	0	0	0	0	0	2	0	1	0	0	62	1,69	21,71	0
2349	0	0	0	0	0	3	0	1	0	0	58	1,7	20,07	0
2350	0	0	0	0	1	1	1	2	1	0	65	1,63	24,46	0
2351	0	0	0	0	0	3	0	1	0	0	58	1,7	20,07	0
2352	1	0	0	0	1	1	1	3	1	1	64	1,68	22,68	0
2353	1	0	0	0	1	1	1	3	1	1	64	1,68	22,68	0
2354	1	0	0	0	1	1	1	3	1	1	64	1,68	22,68	0
2355	1	0	0	0	0	6	0	1	0	0	55	1,55	22,89	0
2356	1	1	0	0	1	1	1	4	2	1	74	1,65	27,18	0
2357	1	1	0	0	1	1	1	4	2	1	74	1,65	27,18	0
2358	1	1	0	0	1	1	1	4	2	1	74	1,65	27,18	0
2359	1	0	0	0	0	3	1	3	2	0	75	1,54	31,62	0
2360	1	0	1	0	1	3	1	3	1	1	42	1,56	17,26	0
2361	1	0	1	0	1	3	1	3	1	1	42	1,56	17,26	0
2362	1	0	0	0	0	1	0	1	0	0	52	1,59	20,57	0
2363	1	0	0	0	0	1	0	1	0	0	52	1,59	20,57	0
2364	2	0	1	0	1	5	1	2	1	0	40	1,42	19,84	0
2365	2	0	1	0	1	5	1	2	1	0	40	1,42	19,84	0
2366	0	0	0	0	1	1	0	1	0	0	60	1,75	19,59	0
2367	0	0	0	0	1	1	0	1	0	0	60	1,75	19,59	0
2368	0	0	0	0	1	1	0	1	0	0	60	1,75	19,59	0
2369	1	0	0	0	0	3	1	2	1	0	54	1,6	21,09	0
2370	0	0	0	0	0		1	2	1	0	52,5	1,59	20,77	0
2371	0	0	0	0	0		1	7	3	3	78	1,61	30,09	0
2372	0	0	0	0	0		1	2	1	0	79	1,67	28,33	0
2373	0	0	0	0	0		1	2	1	0	79	1,67	28,33	0
2374	0	0	0	0	0		1	7	3	3	78	1,61	30,09	0
2375	0	0	0	0	0		0	1	0	0	73	1,62	27,82	0

2376	0	0	0	0	0		0	1	0	0	73	1,62	27,82	0
2377	0	0	0	0	0		0	1	0	0	50	1,63	18,82	0
2378	0	0	0	0	0		0	1	0	0	74	1,76	23,89	0
2379	0	0	0	0	0		0	1	0	0	72	1,74	23,78	0
2380	0	0	0	0	0		1	9	1	7	68	1,55	28,3	0
2381	0	0	0	0	0		1	2	1	0	65	1,66	23,59	0
2382	0	0	0	0	0		0	1	0	0	57	1,6	22,27	0
2383	0	0	0	0	0		1	2	1	0	60	1,6	23,44	0
2384	0	0	0	0	0		1	2	1	0	61	1,68	21,61	0
2385	0	0	0	0	0		0	1	0	0	55	1,72	18,59	0
2386	0	0	0	0	0		1	4	2	1	44	1,56	18,08	0
2387	0	0	0	0	0		1	3	2	0	76	1,64	28,26	0
2388	0	0	0	0	0		0	2	0	1	58	1,65	21,3	0
2389	0	0	0	0	0		0	1	0	0	68	1,6	26,56	0
2390	0	0	0	0	0		1	4	2	2	62	1,61	23,92	0
2391	0	0	0	0	0		1	2	1	0	68	1,63	25,59	0
2392	0	1	0	0	0		0	1	0	0	48	1,64	17,85	0
2393	0	0	0	0	0		0	1	0	0	85	1,8	26,23	0
2394	0	0	0	0	0		0	1	0	0	77	1,67	27,61	0
2395	0	0	0	0	0		1	4	2	1	44	1,56	18,08	0
2396	0	0	0	0	0		0	2	0	1	46	1,68	16,3	0
2397	0	0	0	0	0		1	2	1	0	64	1,62	24,39	0
2398	0	0	0	0	0		0	4	0	3	124	1,64	46,1	0
2399	1	0	0	0	0		1	3	1	1	99	1,6	38,67	0
2400	0	0	1	0	0		1	2	1	0	84	1,59	33,23	0
2401	1	0	0	0	0	1	1	2	1	0	50	1,6	19,53	0
2402	1	0	0	0	0	1	1	2	1	0	50	1,6	19,53	0
2403	0	0	1	0	0		0	1	0	0	65	1,62	24,77	0
2404	1	0	0	0	0	2	1	3	1	1	56	1,49	25,22	0
2405	1	0	0	0	0	2	1	3	1	1	56	1,49	25,22	0
2406	1	0	0	0	0	1	0	1	0	0	80	1,64	29,74	0
2407	0	0	0	0	0	1	0	1	0	0	68	1,59	26,9	0
2408	0	0	0	0	0	1	0	1	0	0	68	1,59	26,9	0

2409	1	0	0	0	0	1	1	4	1	2	57	1,57	23,12	0
2410	1	0	0	0	0	1	1	4	1	2	57	1,57	23,12	0
2411	1	0	0	0	0	1	0	1	0	0	80	1,67	28,69	0
2412	1	0	0	0	0	1	0	1	0	0	80	1,67	28,69	0
2413	1	0	0	0	0	1	0	1	0	0	80	1,67	28,69	0
2414	1	0	0	0	0	1	1	6	3	2	45	1,64	16,73	0
2415	1	0	0	0	0	1	1	6	3	2	45	1,64	16,73	0
2416	0	0	0	0	0	6	0	1	0	0	49	1,64	18,22	0
2417	0	0	0	0	0	1	0	1	0	0	54	1,6	21,09	0
2418	2	0	0	0	1	3	0	2	0	1	49	1,6	19,14	0
2419	1	0	0	0	1	1	0	2	0	1	65	1,6	25,39	0
2420	2	0	0	0	1	1	0	1	0	0	58	1,67	20,8	0
2421	2	0	0	0	1	1	0	1	0	0	58	1,67	20,8	0
2422	2	0	1	0	1	1	0	1	0	0	60	1,72	20,28	0
2423	2	0	1	0	1	1	0	1	0	0	60	1,72	20,28	0
2424	3	1	0	0	0	1	1	2	1	1	65	1,7	22,49	0
2425	3	1	0	0	0	1	1	2	1	1	65	1,7	22,49	0
2426	0	0	0	0	0	1	0	2	0	1	68	1,63	25,59	0
2427	0	1	0	0	0	1	1	3	1	1	57	1,65	20,94	0
2428	0	1	0	0	0	1	1	3	1	1	57	1,65	20,94	0
2429	0	0	1	0	0	1	1	2	1	0	63	1,68	22,32	0
2430	0	0	1	0	0	1	1	2	1	0	63	1,68	22,32	0
2431	0	1	0	0	0	1	1	3	1	1	57	1,65	20,94	0
2432	2	0	0	0	0	2	1	3	1	1	53	1,62	20,2	0
2433	2	0	0	0	0	2	1	3	1	1	53	1,62	20,2	0
2434	1	0	0	0	0	1	1	2	1	0	53	1,58	21,23	0
2435	1	0	0	0	0	5	1	3	2	0	57	1,65	20,94	0
2436	1	0	0	0	0	5	1	3	2	0	57	1,65	20,94	0
2437	2	0	0	0	0	1	1	2	1	0	93	1,59	36,79	0
2438	2	0	0	0	0	1	1	2	1	0	93	1,59	36,79	0
2439	0	0	0	0	1	1	1	3	1	1	58	1,66	21,05	0
2440	0	0	0	0	1	1	1	3	1	1	58	1,66	21,05	0
2441	0	0	1	0	0	3	0	1	0	0	56	1,54	23,61	0

2442	0	0	1	0	0	3	0	1	0	0	56	1,54	23,61	0
2443	1	1	0	0	1	2	0	1	0	0	59	1,68	20,9	0
2444	1	1	0	0	1	2	0	1	0	0	59	1,68	20,9	0
2445	0	1	1	0	0	1	1	3	1	1	95	1,75	31,02	0
2446	1	0	0	0	0	3	1	5	4	0	56	1,52	24,24	0
2447	1	0	0	0	0	3	1	5	4	0	56	1,52	24,24	0
2448	1	0	0	0	0	1	0	1	0	0	55	1,65	20,2	0
2449	1	0	0	0	0	1	0	1	0	0	55	1,65	20,2	0
2450	1	0	1	0	1	6	1	2	1	0	63	1,64	23,42	0
2451	1	0	1	0	1	6	1	2	1	0	63	1,64	23,42	0
2452	1	0	0	0	0	1	1	2	1	0	56	1,66	20,32	0
2453	1	0	0	0	0	1	1	2	1	0	56	1,66	20,32	0
2454	0	0	0	0	0		1	3	1	1	53	1,55	22,06	0
2455	0	0	0	0	0		1	2	1	0	64	1,74	21,14	0
2456	0	0	0	0	0		0	8	0	7		1,63		0
2457	0	0	0	0	0		0	2	0	1	64	1,8	19,75	0
2458	0	0	0	0	0		1	3	1	1	63	1,69	22,06	0
2459	0	0	0	0	0		0	1	0	0	45	1,63	16,94	0
2460	0	0	0	0	0		1	5	3	1	84	1,6	32,81	0
2461	2	0	0	1	0	1	0	3	0	2	71	1,69	24,86	0
2462	2	0	0	1	0	1	0	3	0	2	71	1,69	24,86	0

ID Code	History IUGR (0= nee, 1=ja)	Gest age delivery	Mode delivery	Medication	Diagnosis (1=NL, 2=EPE, 3=LPE, 4=GH, 5=EH, 6=HELLP, 7=IUGR)
1	0	39,14	Vaginale partus	0	1
2	0	40	Secundaire sectio	0	1
3	0	35,57	Vaginale partus	0	1
4	0	40	Vaginale partus	0	1
5	0	33,86	Primaire sectio	0	1
6	0	37,57	Vaginale partus	0	1
7	0	33	Primaire sectio	0	1
8	0	40,28	Vaginale partus	0	1
9	0	37,14	Vaginale partus	0	1
10	0	40	Vaginale partus	0	1
11	0	30,72	Primaire sectio	0	1
12	0	39,57	Vaginale partus	2	1
13	0	38,72	Vaginale partus	0	1
14	0	34,43	Primaire sectio	0	1
15	0	37,14	Secundaire sectio	0	1
16	0	37,14	Secundaire sectio	0	1
17	0	34,43	Primaire sectio	0	1
18	0	38,86	Vaginale partus	0	1
19	0	38,86	Vaginale partus	0	1
20	0	37,57	Secundaire sectio	0	1
21	0	37,57	Secundaire sectio	0	1
22	0	40,14	vaginaal	1	1
23	0	38,28	Vaginale partus	0	1
24	0	39	Repeat sectio	0	1
25	0	39	Repeat sectio	0	1
26	0	40	Vaginale partus	0	1
27	0	40	Vaginale partus	0	1
28	0	39,28	Secundaire sectio	0	1
29	0	39,28	Secundaire sectio	0	1
30	0	40,43	Vaginale partus	0	1
31	0	40,43	Vaginale partus	0	1
32	0	23,57	Vaginale partus	1	1

33	0	23,57	Vaginale partus	1	1
34	0	34	prim.sectio	0	1
35	0	37,28	vaginaal	0	1
36	0	34,72	sec.sectio	0	1
37	0	37,43	vaginaal	0	1
38	0	39	Vaginale partus	0	1
39	0	39	Vaginale partus	0	1
40	0	40,14	Vaginale partus	0	1
41	0	39,43	Primaire sectio	0	1
42	0	38,86	Vaginale partus	0	1
43	0	31,43	Primaire sectio	0	1
44	0	36	Vaginale partus	0	1
45	0	37,14	Primaire sectio	0	1
46	0	41,28	Vaginale partus	0	1
47	0	40,43	Vaginale partus	0	1
48	0	40,28	Vaginale partus	0	1
49	0	37,14	Primaire sectio	0	1
50	0	39	prim.sectio	0	1
51	0	39,57	Vaginale partus	0	1
52	0	37,86	Vaginale partus	0	1
53	0	31,43	Primaire sectio	0	1
54	0	32,43	Secundaire sectio	0	1
55	0	37,28	Vaginale partus	0	1
56	0	38,57	Vaginale partus	0	1
57	0	38,57	Vaginale partus	0	1
58	0	39,14	Vaginale partus	0	1
59	0	40,43	Vaginale partus	0	1
60	0	34,14	Vaginale partus	0	1
61	0	40,43	Vaginale partus	0	1
62	0	38,57	Vaginale partus	0	1
63	0	40,72	vaginaal	0	1
64	0	40,43	Vaginale partus	0	1
65	0	36,57	Primaire sectio	0	1

66	0	37,86	Primaire sectio	0	1
67	0	40,43	Vaginale partus	0	1
68	0	38	Primaire sectio	0	1
69	0	38	Primaire sectio	0	1
70	0	38,43	Primaire sectio	0	1
71	0	38,14	Vaginale partus	0	1
72	0	37,43	Secundaire sectio	0	1
73	0	38,72	Secundaire sectio	0	1
74	0	40,28	Vaginale partus	0	1
75	0	35,86	Vaginale partus	0	1
76	0	31,86	Primaire sectio	0	1
77	0	40,43	Vaginale partus	0	1
78	0	41	Repeat sectio	0	1
79	0	35,43	Vaginale partus	0	1
80	0	41,43	Vaginale partus	0	1
81	0	33,86	Vaginale partus	0	1
82	0	41,43	Vaginale partus	0	1
83	0	40,72	Vaginale partus	0	1
84	0	37,86	Vaginale partus	0	1
85	0	37,86	Vaginale partus	0	1
86	0	41,14	Vaginale partus	0	1
87	0	31,43	Vaginale partus	0	1
88	0	35,14	Vaginale partus	0	1
89	0	38,57	Vaginale partus	0	1
90	0	38,57	Vaginale partus	0	1
91	0	39,43	Vaginale partus	0	1
92	0	39,43	Vaginale partus	0	1
93	0	41,57	Vaginale partus	0	1
94	0	38,86	Repeat sectio	0	1
95	0	38,43	Vaginale partus	0	1
96	0	40	Vaginale partus	0	1
97	0	37,86	Vaginale partus	0	1
98	0	39,57	Vaginale partus	0	1

99	0	40,86	Vaginale partus	0	1
100	0	40	Vaginale partus	0	1
101	0	40,14	Vaginale partus	0	1
102	0	40,86		0	1
103	0	39,43	Vaginale partus	0	1
104	0	39,14	Vaginale partus	0	1
105	0	40,57	Vaginale partus	0	1
106	0	41,14	Vaginale partus	0	1
107	0	40,57	Vaginale partus	0	1
108	0	39,28	Kunstverlossing	0	1
109	0	38,28	Primaire sectio	0	1
110	0	38,28	Primaire sectio	0	1
111	0	40,43	Vaginale partus	0	1
112	0	40,43	Vaginale partus	0	1
113	0	39,86	Vaginale partus	0	1
114	0	41	Vaginale partus	0	1
115	0	40	Vaginale partus	0	1
116	0	39,57	Vaginale partus	0	1
117	0	38,72	Primaire sectio	0	1
118	0	37,14	Vaginale partus	0	1
119	0	39,86	Vaginale partus	0	1
120	0	41,28	Vaginale partus	0	1
121	0	38,14	Vaginale partus	0	1
122	0	37,72	Primaire sectio	0	1
123	0	39,72	Vaginale partus	0	1
124	0	41,28	Vaginale partus	0	1
125	0	41,57	vaginaal	0	1
126	0	37,72	Primaire sectio	0	1
127	0	39,72	Vaginale partus	0	1
128	0	39,72	Vaginale partus	0	1
129	0	39,72	Vaginale partus	0	1
130	0	34	Vaginale partus	0	1
131	0	40,86	vaginaal	0	1

132	0	34	Vaginale partus	0	1
133	0	38,57	Primaire sectio	0	1
134	0	36,72	Vaginale partus	0	1
135	0	40,28	Kunstverlossing	0	1
136	0	38,43	Vaginale partus	0	1
137	0	37,43	Vaginale partus	0	1
138	0	39,72	Vaginale partus	0	1
139	0	39,28	Vaginale partus	0	1
140	0	38,43	Vaginale partus	0	1
141	0	40,43	Primaire sectio	0	1
142	0	40,72	Vaginale partus	0	1
143	0	41,57	Vaginale partus	0	1
144	0	40,28	Secundaire sectio	0	1
145	0	40,28	Secundaire sectio	0	1
146	0	38,72	sec.sectio	0	1
147	0	39	Vaginale partus	0	1
148	0	38,43	Vaginale partus	0	1
149	0	39,43	Vaginale partus	0	1
150	0	38,72	Vaginale partus	0	1
151	0	40,14	Vaginale partus	0	1
152	0	39,72	Vaginale partus	0	1
153	0	40,86	Vaginale partus	0	1
154	0	39,72	Vaginale partus	0	1
155	0	40,43	Vaginale partus	0	1
156	0	40,72	Vaginale partus	0	1
157	0	40,57	vaginaal	0	1
158	0	39,86	Vaginale partus	0	1
159	0	39,86	Vaginale partus	0	1
160	0	41,28	Vaginale partus	0	1
161	0	41,28	Vaginale partus	0	1
162	0	38,28	Primaire sectio	0	1
163	0	38,28	Primaire sectio	0	1
164	0	39	Vaginale partus	0	1

165	0	39	Vaginale partus	0	1
166	0	38,57	Repeat sectio	0	1
167	0	38,57	Repeat sectio	0	1
168	0	37,57	vaginaal	0	1
169	0	34,86	Vaginale partus	0	1
170	0	40,28	Vaginale partus	0	1
171	0	40,28	Vaginale partus	0	1
172	0	40,57	Kunstverlossing	0	1
173	0	38,72	Primaire sectio	1	1
174	0	39,14	Primaire sectio	2	1
175	0	41,86	vaginaal	2	1
176	0	36,43	Vaginale partus	2	1
177	0	35,72	Primaire sectio	2	1
178	0	39,43	Repeat sectio	2	1
179	0	39,43	Repeat sectio	2	1
180	0	39,43	Repeat sectio	2	1
181	0	40	Vaginale partus	1	1
182	0	39,43	Vaginale partus	0	1
183	0	35,43	Vaginale partus	1	1
184	0	40,14	Vaginale partus	0	1
185	0	39,28	Vaginale partus	1	1
186	0	40,72	Vaginale partus	0	1
187	0	38,57	Repeat sectio	0	1
188	0	39,28	Vaginale partus	0	1
189	0	39,57	Vaginale partus	0	1
190	0	35,86	Primaire sectio	0	1
191	0	35,86	Primaire sectio	0	1
192	0	40,86	Vaginale partus	1	1
193	0	39	vaginaal	0	1
194	0	40,14	vaginaal	1	1
195	0	40,14	Vaginale partus	0	1
196	0	39,57	Vaginale partus	0	1
197	0	39,57	Vaginale partus	0	1

198	0	40,72	Vaginale partus	0	1
199	0	37,14	Vaginale partus	0	1
200	0	28,57	Secundaire sectio	0	1
201	0	39,43		1	1
202	0	39,43		1	1
203	0	39,43		1	1
204	0	37	Secundaire sectio	1	1
205	0	37	Secundaire sectio	1	1
206	0	40,72	Vaginale partus	1	1
207	0	40,72	Vaginale partus	1	1
208	0	41,14	Vaginale partus	0	1
209	0	39,14	Secundaire sectio	0	1
210	0	39,86	Vaginale partus	0	1
211	0	41,28	Vaginale partus	0	1
212	0	40,86	Vaginale partus	2	1
213	0	38,28	Vaginale partus	0	1
214	0	38,28	Vaginale partus	0	1
215	0	38,28	Vaginale partus	2	1
216	0	38,28	Vaginale partus	2	1
217	0	40,43	Vaginale partus	0	1
218	0	39,72	Vaginale partus	2	1
219	0	39,72	Vaginale partus	2	1
220	0	39,72	Vaginale partus	2	1
221	0	38,28	Vaginale partus	0	1
222	0	37,86	Vaginale partus	1	1
223	0	37,28	Secundaire sectio	0	1
224	0	35,57	Secundaire sectio	0	1
225	0	39,14	Vaginale partus	0	1
226	0	36,86	Vaginale partus	0	1
227	0	35,72	Vaginale partus	0	1
228	0	39,14	Vaginale partus	0	1
229	0	38,43	Vaginale partus	2	1
230	0	38,43	Vaginale partus	2	1

231	1	36,43	Vaginale partus	0	1
232	1	36,43	Vaginale partus	0	1
233	1	36,43	Vaginale partus	0	1
234	0	37,28	vaginaal	1	1
235	0	32,14	Primaire sectio	0	1
236	0	37,14	Vaginale partus	0	1
237	0	38,14	Primaire sectio	0	1
238	0	38,14	Primaire sectio	0	1
239	0	38,57	vaginaal	0	1
240	0	40,14	Secundaire sectio	0	1
241	0	36,28	Secundaire sectio	0	1
242	0	38	Repeat sectio	0	1
243	0	38	Repeat sectio	0	1
244	0	41,14	Primaire sectio	0	1
245	0	37,14	Repeat sectio	0	1
246	0	37,14	Repeat sectio	0	1
247	0	37,86	Vaginale partus	1	1
248	0	37,86	Vaginale partus	1	1
249	0	38	Primaire sectio	1	1
250	0	36,86	Secundaire sectio	1	1
251	0	39,57	Vaginale partus	0	1
252	0	39,57	Vaginale partus	0	1
253	0	38,86	Vaginale partus	0	1
254	0	38,86	Vaginale partus	0	1
255	0	39,43	Vaginale partus	0	1
256	0	39,43	Vaginale partus	0	1
257	0	39	Primaire sectio	0	1
258	0	37,43	Vaginale partus	0	1
259	0	37,43	Vaginale partus	0	1
260	0	36,72	Vaginale partus	0	1
261	0	36,72	Vaginale partus	0	1
262	0	36,86	Vaginale partus	0	1
263	0	32,72	Vaginale partus	0	1

264	0	32,72	Vaginale partus	0	1
265	0	40,57	Vaginale partus	0	1
266	0	39,72	Vaginale partus	0	1
267	0	39,72	Vaginale partus	0	1
268	0	40,28	Vaginale partus	1	1
269	0	40,28	Vaginale partus	1	1
270	0	36,43	Secundaire sectio	0	1
271	0	36,43	Secundaire sectio	0	1
272	0	39,72	Secundaire sectio	0	1
273	0	39,72	Secundaire sectio	0	1
274	0	37,43	Vaginale partus	0	1
275	0	37,43	Vaginale partus	0	1
276	0	41,57	Secundaire sectio	0	1
277	0	41,57	Secundaire sectio	0	1
278	0	39,14	Vaginale partus	0	1
279	0	39,14	Vaginale partus	0	1
280	0	39,43	Vaginale partus	0	1
281	0	39,43	Vaginale partus	0	1
282	0	41,14	Vaginale partus	0	1
283	0	41,14	Vaginale partus	0	1
284	0	38,28	Repeat sectio	0	1
285	0	38,28	Repeat sectio	0	1
286	0	40,14	Vaginale partus	0	1
287	0	40,14	Vaginale partus	0	1
288	0	39,14	Vaginale partus	0	1
289	0	39,14	Vaginale partus	0	1
290	0	40,72	Vaginale partus	0	1
291	0	40,72	Vaginale partus	0	1
292	0	40,57	Vaginale partus	0	1
293	0	40,57	Vaginale partus	0	1
294	0	41,14	Vaginale partus	0	1
295	0	41,14	Vaginale partus	0	1
296	0	39	Vaginale partus	0	1

297	0	39	Vaginale partus	0	1
298	0	38,86	Vaginale partus	1	1
299	0	38,86	Vaginale partus	1	1
300	0	40,86	Vaginale partus	1	1
301	0	40,86	Vaginale partus	1	1
302	0	37,43	Vaginale partus	0	1
303	0	37,43	Vaginale partus	0	1
304	0	39,28	Vaginale partus	0	1
305	0	39,28	Vaginale partus	0	1
306	0	40,72	Vaginale partus	0	1
307	0	40,72	Vaginale partus	0	1
308	0	41,28	Vaginale partus	1	1
309	0	39,14	Vaginale partus	0	1
310	0	39,14	Vaginale partus	0	1
311	0	38,14	Vaginale partus	0	1
312	0	38,14	Vaginale partus	0	1
313	0	40,14	Vaginale partus	0	1
314	0	40,14	Vaginale partus	0	1
315	0	36,14	Repeat sectio	0	1
316	0	36,14	Repeat sectio	0	1
317	0	37,72	Vaginale partus	1	1
318	0	37,72	Vaginale partus	1	1
319	0	39,86	Vaginale partus	1	1
320	0	39,86	Vaginale partus	1	1
321	0	37,43	Vaginale partus	0	1
322	0	41,28	Vaginale partus	0	1
323	0	41,28	Vaginale partus	0	1
324	0	37,72	Vaginale partus	0	1
325	0	37,72	Vaginale partus	0	1
326	0	40,28	Vaginale partus	0	1
327	0	39,28	Vaginale partus	0	1
328	0	39,28	Vaginale partus	0	1
329	0	39	Vaginale partus	0	1

330	0	37,72	Vaginale partus	0	1
331	0	37,72	Vaginale partus	0	1
332	0	41,14	Vaginale partus	0	1
333	0	41,14	Vaginale partus	0	1
334	0	40	Vaginale partus	0	1
335	0	40	Vaginale partus	0	1
336	0	40,43	Vaginale partus	0	1
337	0	40,43	Vaginale partus	0	1
338	0	41,43	Vaginale partus	0	1
339	0	38,28	Repeat sectio	0	1
340	0	38,28	Repeat sectio	0	1
341	0	40,57	Vaginale partus	1	1
342	0	40,57	Vaginale partus	1	1
343	0	40	Vaginale partus	0	1
344	0	40	Vaginale partus	0	1
345	0	38,57	Vaginale partus	0	1
346	0	38,57	Vaginale partus	0	1
347	0	41	Secundaire sectio	1	1
348	0	41	Secundaire sectio	1	1
349	0	41,14	Vaginale partus	0	1
350	0	41,14	Vaginale partus	0	1
351	0	40,57	Vaginale partus	0	1
352	0	40,57	Vaginale partus	0	1
353	0	40,57	Vaginale partus	0	1
354	0	40,57	Vaginale partus	0	1
355	0	34,57	Vaginale partus	1	1
356	0	34,57	Vaginale partus	1	1
357	0	40,14	vaginaal	0	1
358	0	41,28	vaginaal	0	1
359	0	37	vaginaal	0	1
360	0	37	vaginaal	0	1
361	0	40,43	vaginaal	0	1
362	0	38,86	prim.sectio	0	1

363	0	37,57	vaginaal	0	1
364	0	39,43	vaginaal	0	1
365	0	40,28	vaginaal	0	1
366	0	41,57	sec.sectio	0	1
367	0	40,28	vaginaal	2	1
368	0	37,43	vaginaal	1	1
369	0	39	vaginaal	1	1
370	0	39,57	prim.sectio	1	1
371	0	40	prim.sectio	1	1
372	0	39,86	vaginaal	0	1
373	0	39,72	vaginaal	0	1
374	0	39,14	vaginaal	1	1
375	0	38,86	prim.sectio	0	1
376	0	40,28	vaginaal	0	1
377	0	36	vaginaal	0	1
378	0	40,14	vaginaal	0	1
379	0	38,72	vaginaal	1	1
380	0	38,57	vaginaal	0	1
381	0	40,86	vaginaal	0	1
382	0	38,28	prim.sectio	0	1
383	0	41	vaginaal	1	1
384	0	38,14	vaginaal	1	1
385	0	39	Vaginale partus	0	1
386	0	39	Vaginale partus	0	1
387	0	41	Vaginale partus	0	1
388	0	41	Vaginale partus	0	1
389	0	40,43	Vaginale partus	0	1
390	0	40,43	Vaginale partus	0	1
391	0	40,14	Vaginale partus	0	1
392	0	40,14	Vaginale partus	0	1
393	0	39,57	Vaginale partus	2	1
394	0	39,57	Vaginale partus	2	1
395	0	37,14	Vaginale partus	0	1

396	0	37,14	Vaginale partus	0	1
397	0	40,14	Vaginale partus	0	1
398	0	40,14	Vaginale partus	0	1
399	0	39,57	Vaginale partus	0	1
400	0	39,57	Vaginale partus	0	1
401	0	40,43	Vaginale partus	0	1
402	0	39,43	Vaginale partus	1	1
403	0	39,43	Vaginale partus	1	1
404	0	37,43	Vaginale partus	0	1
405	0	40,14	vaginaal	0	1
406	0	41,14	vaginaal	0	1
407	0	39,86	vaginaal	0	1
408	0	38,28	vaginaal	0	1
409	0	40	vaginaal	0	1
410	0	38,57	sec.sectio	0	1
411	0	39,43	vaginaal	1	1
412	0	40,57	vaginaal	0	1
413	0	40	vaginaal	0	1
414	0	41,14	sec.sectio	0	1
415	0	35,57	vaginaal	1	1
416	0	30,57	vaginaal	0	1
417	0	41	Vaginale partus	0	1
418	0	40,28	Vaginale partus	0	1
419	0	40,28	Vaginale partus	0	1
420	0	39,86	vaginaal	0	1
421	0	41,57	Vaginale partus	0	1
422	0	41,57	Vaginale partus	0	1
423	0	38	vaginaal	0	1
424	0	38,86	Primaire sectio	0	1
425	0	38,86	Primaire sectio	0	1
426	0	37,14	vaginaal	0	1
427	0	37,28	vaginaal	0	1
428	0	39,28	vaginaal	0	1

429	0	38	sec.sectio	0	1
430	0	40,57	Vaginale partus	0	1
431	0	40,57	Vaginale partus	0	1
432	0	39,86	vaginaal	0	1
433	0	39,57	Vaginale partus	0	1
434	0	39,57	Vaginale partus	0	1
435	0	41,14	vaginaal	2	1
436	0	38,72	vaginaal	0	1
437	0	41	sec.sectio	1	1
438	0	37,43	Repeat sectio	1	1
439	0	37,43	Repeat sectio	1	1
440	0	41,14	Vaginale partus	0	1
441	0	41,14	Vaginale partus	0	1
442	0	41,43	Vaginale partus	0	1
443	0	41,43	Vaginale partus	0	1
444	0	41	Vaginale partus	0	1
445	0	41	Vaginale partus	0	1
446	0	38,57	Primaire sectio	0	1
447	0	38,57	Primaire sectio	0	1
448	0	39,72	vaginaal	1	1
449	0	40,57		0	1
450	0	39,43	Vaginale partus	0	1
451	0	39,43	Vaginale partus	0	1
452	0	40,14	vaginaal	0	1
453	0	40,14	Vaginale partus	0	1
454	0	40,14	Vaginale partus	0	1
455	0	40,14	Vaginale partus	0	1
456	0	40,14	Vaginale partus	0	1
457	0	40,28	Vaginale partus	0	1
458	0	41	vaginaal	0	1
459	0	40	Vaginale partus	0	1
460	0	40	Vaginale partus	0	1
461	0	38,43	Vaginale partus	1	1

462	0	38,43	Vaginale partus	1	1
463	0	38,57	Vaginale partus	0	1
464	0	38,57	Vaginale partus	0	1
465	0	40,28	Vaginale partus	0	1
466	0	40,28	Vaginale partus	0	1
467	0	41,43	Secundaire sectio	0	1
468	0	40,57	Vaginale partus	0	1
469	0	40,57	Vaginale partus	0	1
470	0	39,43	Vaginale partus	0	1
471	0	39,43	Vaginale partus	0	1
472	0	37,14	Primaire sectio	0	1
473	0	37,14	Primaire sectio	0	1
474	0	39	Vaginale partus	0	1
475	0	39	Vaginale partus	0	1
476	0	39,14	Primaire sectio	0	1
477	0	38,43	Vaginale partus	0	1
478	0	38,43	Vaginale partus	0	1
479	0	39,14	Primaire sectio	0	1
480	0	40,28	vaginaal	0	1
481	0	39,14	Vaginale partus	0	1
482	0	41,14	Vaginale partus	0	1
483	0	39,14	Vaginale partus	0	1
484	0	39,28	vaginaal	0	1
485	0	40	Vaginale partus	0	1
486	0	39,43	vaginaal	0	1
487	0	40	Vaginale partus	0	1
488	0	40,86	Vaginale partus	0	1
489	0	40,86	Vaginale partus	0	1
490	0	39,14	Vaginale partus	0	1
491	0	39,14	Vaginale partus	0	1
492	0	39,43	Vaginale partus	0	1
493	0	39,43	Vaginale partus	0	1
494	0	41,14	Vaginale partus	0	1

495	0	39	Vaginale partus	0	1
496	0	39,86	Vaginale partus	0	1
497	0	40,28	Vaginale partus	0	1
498	0	41,14	Vaginale partus	0	1
499	0	39,86	Vaginale partus	0	1
500	0	39	Vaginale partus	0	1
501	0	40,28	Vaginale partus	0	1
502	0	38,57	Vaginale partus	0	1
503	0	40,57	Vaginale partus	0	1
504	0	40,57	Vaginale partus	0	1
505	0	38,57	Vaginale partus	0	1
506	0	40,57	Vaginale partus	0	1
507	0	40,57	Vaginale partus	0	1
508	0	39,14	Vaginale partus	0	1
509	0	39,14	Vaginale partus	0	1
510	0	38,28	sec.sectio	0	1
511	0	39,86	Vaginale partus	0	1
512	0	39,86	Vaginale partus	0	1
513	0	40,72	Vaginale partus	0	1
514	0	40,72	Vaginale partus	0	1
515	0	39,14	Vaginale partus	0	1
516	0	39,14	Vaginale partus	0	1
517	0	39,72	vaginaal	0	1
518	0	38,72	Vaginale partus	0	1
519	0	37,72	Vaginale partus	0	1
520	0	37,72	Vaginale partus	0	1
521	0	40	Vaginale partus	0	1
522	0	40	Vaginale partus	0	1
523	0	40	Vaginale partus	0	1
524	0	40	Vaginale partus	0	1
525	0	40,28	Vaginale partus	0	1
526	0	38,43	Vaginale partus	0	1
527	0	41,28	vaginaal	0	1

528	0	40,28	Vaginale partus	0	1
529	0	38,43	Vaginale partus	0	1
530	0	38,43	Vaginale partus	0	1
531	0	41,57	Vaginale partus	0	1
532	0	38,43	Vaginale partus	0	1
533	0	41,57	Vaginale partus	0	1
534	0	40,57	Vaginale partus	0	1
535	0	39,72	Vaginale partus	0	1
536	0	39,72	Vaginale partus	0	1
537	0	39,57	vaginaal	0	1
538	0	40,57	Vaginale partus	0	1
539	0	39,14	vaginaal	0	1
540	0	40,57	Vaginale partus	0	1
541	0	40,28	vaginaal	0	1
542	0	39,86	vaginaal	0	1
543	0	39,43	Vaginale partus	0	1
544	0	39,43	Vaginale partus	0	1
545	0	37,72	Vaginale partus	0	1
546	0	37,72	Vaginale partus	0	1
547	0	37,14	vaginaal	0	1
548	0	41,43	vaginaal	0	1
549	0	41,14	vaginaal	0	1
550	0	40,28	Vaginale partus	0	1
551	0	39,43	vaginaal	0	1
552	0	40,28	Vaginale partus	0	1
553	0	40,57	Secundaire sectio	0	1
554	0	40,57	Secundaire sectio	0	1
555	0	40	Vaginale partus	0	1
556	0	40	Vaginale partus	0	1
557	0	39,57	vaginaal	0	1
558	0	39,14	Vaginale partus	0	1
559	0	39,28	Secundaire sectio	0	1
560	0	39,28	Secundaire sectio	0	1

561	0	40,28	Vaginale partus	0	1
562	0	40,28	Vaginale partus	0	1
563	0	41,14	Vaginale partus	0	1
564	0	41,14	Vaginale partus	0	1
565	0	38,86	Vaginale partus	0	1
566	0	39	Vaginale partus	0	1
567	0	38,86	Vaginale partus	0	1
568	0	39	Vaginale partus	0	1
569	0	38,72	vaginaal	0	1
570	0	41,28	Vaginale partus	0	1
571	0	39,28	vaginaal	0	1
572	0	41,28	Vaginale partus	0	1
573	0	39,14	vaginaal	0	1
574	0	41,14	Secundaire sectio	0	1
575	0	41	Vaginale partus	0	1
576	0	41,14	Secundaire sectio	0	1
577	0	39,57	Vaginale partus	0	1
578	0	41	Vaginale partus	0	1
579	0	39,86	Vaginale partus	0	1
580	0	39,86	Vaginale partus	0	1
581	0	39,57	Vaginale partus	0	1
582	0	39,86	Vaginale partus	0	1
583	0	39,86	Vaginale partus	0	1
584	0	40,57	Secundaire sectio	0	1
585	0	40,57	Secundaire sectio	0	1
586	0	40,57	Secundaire sectio	0	1
587	0	39,86	Vaginale partus	0	1
588	0	39,86	Vaginale partus	0	1
589	0	41	Vaginale partus	0	1
590	0	40,57	Vaginale partus	0	1
591	0	40,57	Vaginale partus	0	1
592	0	39,57	Vaginale partus	0	1
593	0	38,72	Primaire sectio	0	1

594	0	37,43	Vaginale partus	0	1
595	0	37,43	Vaginale partus	0	1
596	0	40	Vaginale partus	0	1
597	0	41	Vaginale partus	0	1
598	0	39,57	Vaginale partus	0	1
599	0	40,86	Vaginale partus	0	1
600	0	38,72	Primaire sectio	0	1
601	0	39,57	Vaginale partus	0	1
602	0	40,86	Vaginale partus	0	1
603	0	39,57	Vaginale partus	0	1
604	0	38,57	Vaginale partus	0	1
605	0	40,14	Vaginale partus	0	1
606	0	40,86	Vaginale partus	0	1
607	0	38,57	Vaginale partus	0	1
608	0	40,14	Vaginale partus	0	1
609	0	39	vaginaal	0	1
610	0	40,86	Vaginale partus	0	1
611	0	40,43	Vaginale partus	0	1
612	0	40,43	Vaginale partus	0	1
613	0	41,14	Vaginale partus	0	1
614	0	41,14	Vaginale partus	0	1
615	0	37,57	Vaginale partus	0	1
616	0	41,14	Vaginale partus	0	1
617	0	39,14	Repeat sectio	0	1
618	0	39,14	Repeat sectio	0	1
619	0	37,57	Vaginale partus	0	1
620	0	40,14	Vaginale partus	0	1
621	0	39,57	Vaginale partus	0	1
622	0	36,86	Vaginale partus	0	1
623	0	39,57	Vaginale partus	0	1
624	0	38,14	Vaginale partus	0	1
625	0	38,14	Vaginale partus	0	1
626	0	40,43	vaginaal	0	1

627	0	36,86	Vaginale partus	0	1
628	0	37,72	Repeat sectio	0	1
629	0	40,14	Secundaire sectio	0	1
630	0	41,28	vaginaal	0	1
631	0	40,14	Secundaire sectio	0	1
632	0	37,72	Repeat sectio	0	1
633	0	37,72	Repeat sectio	0	1
634	0	39,43	Vaginale partus	0	1
635	0	39,72	Vaginale partus	0	1
636	0	39,43	Vaginale partus	0	1
637	0	38,72	Vaginale partus	0	1
638	0	38,72	Vaginale partus	0	1
639	0	39,86	Primaire sectio	0	1
640	0	40,43	Vaginale partus	0	1
641	0	39,86	Primaire sectio	0	1
642	0	40,43	Vaginale partus	0	1
643	0	39		0	1
644	0	39,14	Vaginale partus	0	1
645	0	39,14	Vaginale partus	0	1
646	0	39,72	Vaginale partus	0	1
647	0	39,72	Vaginale partus	0	1
648	0	40,86	Vaginale partus	0	1
649	0	40,86	Vaginale partus	0	1
650	0	40	vaginaal	0	1
651	0	39,57	Vaginale partus	0	1
652	0	39,43	Vaginale partus	0	1
653	0	39,43	Vaginale partus	0	1
654	0	39,57	Secundaire sectio	0	1
655	0	39,57	Secundaire sectio	0	1
656	0	39,14	vaginaal	0	1
657	0	40,86	Repeat sectio	0	1
658	0	39,43	Repeat sectio	0	1
659	0	39,43	Repeat sectio	0	1

660	0	40,72	vaginaal	0	1
661	0	38,28	Vaginale partus	0	1
662	0	38,28	Vaginale partus	0	1
663	0	41,28	Vaginale partus	0	1
664	0	41,28	Vaginale partus	0	1
665	0	39,28	vaginaal	0	1
666	0	40,43	Vaginale partus	0	1
667	0	40,43	Vaginale partus	0	1
668	0	40,14	Vaginale partus	0	1
669	0	40,14	Vaginale partus	0	1
670	0	40,57	vaginaal	0	1
671	0	41,28	Secundaire sectio	0	1
672	0	41,28	Secundaire sectio	0	1
673	0	40,14	vaginaal	0	1
674	0	40,57	Vaginale partus	0	1
675	0	40,57	Vaginale partus	0	1
676	0	40,86	Vaginale partus	0	1
677	0	39,57	Vaginale partus	0	1
678	0	41,28	Vaginale partus	0	1
679	0	41,28	Vaginale partus	0	1
680	0	38,72	vaginaal	0	1
681	0	38,57	Primaire sectio	0	1
682	0	40,28	vaginaal	0	1
683	0	40,14	Vaginale partus	0	1
684	0	40,14	Vaginale partus	0	1
685	0	38,57	Repeat sectio	0	1
686	0	38,57	Repeat sectio	0	1
687	0	40,43	Vaginale partus	0	1
688	0	40,43	Vaginale partus	0	1
689	0	39,57	vaginaal	0	1
690	0	38,57	Vaginale partus	0	1
691	0	38,57	Vaginale partus	0	1
692	0	39,86	Secundaire sectio	0	1

693	0	39,86	Secundaire sectio	0	1
694	0	39,86	Secundaire sectio	0	1
695	0	40	vaginaal	0	1
696	0	39,28	vaginaal	0	1
697	0	38,57	Vaginale partus	0	1
698	0	38,72	Vaginale partus	0	1
699	0	38,72	Vaginale partus	0	1
700	0	39,43	Vaginale partus	0	1
701	0	39,43	Vaginale partus	0	1
702	0	39,14	sec.sectio	0	1
703	0	41,14	vaginaal	0	1
704	0	38	vaginaal	0	1
705	1	39	vaginaal	0	1
706	0	39,72	vaginaal	0	1
707	0	40,43	vaginaal	0	1
708	0	41	vaginaal	0	1
709	0	38,72	vaginaal	0	1
710	0	39,43	vaginaal	0	1
711	0	40,72	vaginaal	0	1
712	0	39,28	vaginaal	0	1
713	0	39,86	vaginaal	0	1
714	0	40,43	prim.sectio	0	1
715	0	37,57	vaginaal	0	1
716	0	40,14	vaginaal	0	1
717	0	40	Vaginale partus	0	1
718	0	40	Vaginale partus	0	1
719	0	39,57	vaginaal	0	1
720	0	39,86	vaginaal	0	1
721	0	38,86	vaginaal	0	1
722	0	39,86	vaginaal	0	1
723	0	40,28	vaginaal	0	1
724	0	37,72	vaginaal	0	1
725	0	39,28	vaginaal	0	1

726	0	41,28	vaginaal	0	1
727	0	38,72	vaginaal	0	1
728	0	38,86	vaginaal	0	1
729	0	41,72	vaginaal	0	1
730	0	41	vaginaal	0	1
731	0	38,43	prim.sectio	0	1
732	0	38,14	vaginaal	0	1
733	0	38	Vaginale partus	0	1
734	0	38	Vaginale partus	0	1
735	0	41,14	Vaginale partus	0	1
736	0	41,14	Vaginale partus	0	1
737	0	37,57	vaginaal	0	1
738	0	39,86	Vaginale partus	0	1
739	0	40,86	Vaginale partus	0	1
740	0	40,86	Vaginale partus	0	1
741	0	37,43	vaginaal	0	1
742	0	41	Vaginale partus	0	1
743	0	41	Vaginale partus	0	1
744	0	41,43	vaginaal	0	1
745	0	41,28	sectio	0	1
746	0	41,43	vaginaal	0	1
747	0	38,57	Vaginale partus	0	1
748	0	38,57	Vaginale partus	0	1
749	0	39	Vaginale partus	0	1
750	0	39	Vaginale partus	0	1
751	1	38,14	prim.sectio	0	1
752	0	39,72	vaginaal	0	1
753	0	40,14	prim.sectio	0	1
754	0	38,72	vaginaal	0	1
755	0	41,57	prim.sectio	0	1
756	0	39	Repeat sectio	0	1
757	0	39	Repeat sectio	0	1
758	0	39	Repeat sectio	0	1

759	0	39	Repeat sectio	0	1
760	0	38,43	Vaginale partus	0	1
761	0	38,43	Vaginale partus	0	1
762	0	37,57	Secundaire sectio	0	1
763	0	37,57	Secundaire sectio	0	1
764	0	39,28	Vaginale partus	0	1
765	0	39,28	Vaginale partus	0	1
766	0	40,86	Vaginale partus	0	1
767	0	40,86	Vaginale partus	0	1
768	0	38,43	Vaginale partus	0	1
769	0	38,43	Vaginale partus	0	1
770	0	39,86	Vaginale partus	0	1
771	0	39,86	Vaginale partus	0	1
772	0	40	Vaginale partus	0	1
773	0	40	Vaginale partus	0	1
774	0	40,28	Vaginale partus	0	1
775	0	40,28	Vaginale partus	0	1
776	0	40,14	vaginaal	0	1
777	0	41,72	vaginaal	0	1
778	0	38,86	vaginaal	0	1
779	0	40,43	Vaginale partus	0	1
780	0	40,43	Vaginale partus	0	1
781	0	41,28	Vaginale partus	0	1
782	0	41,28	Vaginale partus	0	1
783	0	40	Vaginale partus	0	1
784	0	39,28	Vaginale partus	0	1
785	0	40	Vaginale partus	0	1
786	0	37,86	Vaginale partus	0	1
787	0	37,86	Vaginale partus	0	1
788	0	39,43	vaginaal	0	1
789	0	40,43	Vaginale partus	0	1
790	0	40,43	Vaginale partus	0	1
791	0	38,28	Vaginale partus	0	1

792	0	38,28	Vaginale partus	0	1
793	0	41,28	vaginaal	0	1
794	0	38,86	Primaire sectio	0	1
795	0	38,86	Primaire sectio	0	1
796	0	39,43	Vaginale partus	0	1
797	0	39,43	Vaginale partus	0	1
798	0	41,28	Secundaire sectio	0	1
799	0	41,28	Secundaire sectio	0	1
800	0	41,43	Repeat sectio	0	1
801	0	41,43	Repeat sectio	0	1
802	0	38,43	Vaginale partus	0	1
803	0	38,43	Vaginale partus	0	1
804	0	38,86	Vaginale partus	0	1
805	0	38,86	Vaginale partus	0	1
806	0	40,57	Vaginale partus	0	1
807	0	40,57	Vaginale partus	0	1
808	0	39,72	vaginaal	0	1
809	0	40,28	Secundaire sectio	0	1
810	0	40,28	Secundaire sectio	0	1
811	0	41	Vaginale partus	0	1
812	0	41	Vaginale partus	0	1
813	0	40,43	vaginaal	0	1
814	0	38,57	Vaginale partus	0	1
815	0	38,57	Vaginale partus	0	1
816	0	39,72	Vaginale partus	0	1
817	0	40	Vaginale partus	0	1
818	0	40	Vaginale partus	0	1
819	0	40	Vaginale partus	0	1
820	0	39,57	Vaginale partus	0	1
821	0	39,57	Vaginale partus	0	1
822	0	39,86	vaginaal	0	1
823	0	39,43	vaginaal	0	1
824	0	41,14	Vaginale partus	0	1

825	0	41,43	Secundaire sectio	2	1
826	0	41,43	Secundaire sectio	2	1
827	0	41,43	Secundaire sectio	2	1
828	1	40,14	prim.sectio	2	1
829	0	38,57	Primaire sectio	1	1
830	0	37,86	vaginaal	1	1
831	0	41,43	Secundaire sectio	1	1
832	0	41,43	Secundaire sectio	1	1
833	0	40,14	Secundaire sectio	1	1
834	0	40,14	Secundaire sectio	1	1
835	0	39,86	vaginaal	1	1
836	0	40,57	vaginaal	1	1
837	0	40	Secundaire sectio	1	1
838	0	40	Secundaire sectio	1	1
839	0	41	Vaginale partus	1	1
840	0	41	Vaginale partus	1	1
841	0	37,28	Vaginale partus	1	1
842	0	37,28	Vaginale partus	1	1
843	0	41	Vaginale partus	1	1
844	0	41	Vaginale partus	1	1
845	0	41	Repeat sectio	1	1
846	0	41	Repeat sectio	1	1
847	0	38	Primaire sectio	0	1
848	0	38	Primaire sectio	0	1
849	0	39,28	Primaire sectio	0	1
850	0	39,28	Primaire sectio	0	1
851	0	38,86	Primaire sectio	0	1
852	0	38,86	Primaire sectio	0	1
853	0	40,43	vaginaal	0	1
854	0	38,72	Repeat sectio	2	1
855	0	38,72	Repeat sectio	2	1
856	0	40,86	vaginaal	0	1
857	0	39,28	Vaginale partus	1	1

858	0	39,28	Vaginale partus	1	1
859	0	41,14	Vaginale partus	1	1
860	0	41,14	Vaginale partus	1	1
861	0	40,57	Vaginale partus	1	1
862	0	40,57	Vaginale partus	1	1
863	0	40,57	Vaginale partus	1	1
864	0	38,86	Vaginale partus	1	1
865	0	38,86	Vaginale partus	1	1
866	0	40,57	Vaginale partus	1	1
867	0	40,57	Vaginale partus	1	1
868	0	39,72	Vaginale partus	1	1
869	0	39,72	Vaginale partus	1	1
870	0	39,86	Vaginale partus	1	1
871	0	39,86	Vaginale partus	1	1
872	0	38,57	Primaire sectio	0	1
873	0	38,57	Primaire sectio	0	1
874	0	38,72	Secundaire sectio	0	1
875	0	38,72	Secundaire sectio	0	1
876	0	40	prim.sectio	0	1
877	0	39,72	Vaginale partus	0	1
878	0	39,72	Vaginale partus	0	1
879	0	39,28	Vaginale partus	0	1
880	0	39,28	Vaginale partus	0	1
881	0	41,72	Secundaire sectio	0	1
882	0	41,72	Secundaire sectio	0	1
883	0	41	Vaginale partus	0	1
884	0	41	Vaginale partus	0	1
885	0	39,86	Vaginale partus	0	1
886	0	39,86	Vaginale partus	0	1
887	0	39,72	sec.sectio	0	1
888	0	41	Vaginale partus	0	1
889	0	41	Vaginale partus	0	1
890	0	39,57	Vaginale partus	0	1

891	0	40,14	Vaginale partus	0	1
892	0	40,14	Vaginale partus	0	1
893	0	39,86	vaginaal	0	1
894	0	39	Vaginale partus	0	1
895	0	39	Vaginale partus	0	1
896	0	39,57	sec.sectio	0	1
897	0	39,14	Repeat sectio	0	1
898	0	39,14	Repeat sectio	0	1
899	0	37,43	Vaginale partus	0	1
900	0	37,43	Vaginale partus	0	1
901	0	40	vaginaal	0	1
902	0	39,72	Vaginale partus	0	1
903	0	39,72	Vaginale partus	0	1
904	0	41,14	vaginaal	0	1
905	0	39,86	Vaginale partus	1	1
906	0	39,86	Vaginale partus	1	1
907	0	39	Repeat sectio	1	1
908	0	39	Repeat sectio	1	1
909	0	40,14	vaginaal	1	1
910	0	35,86	vaginaal	0	1
911	0	41,57	vaginaal	1	1
912	0	40,43	vaginaal	0	1
913	0	39	Primaire sectio	0	1
914	0	39	Primaire sectio	0	1
915	0	40,72	Vaginale partus	0	1
916	0	40,72	Vaginale partus	0	1
917	0	41,14	vaginaal	0	1
918	0	37,86	Primaire sectio	0	1
919	0	37,86	Primaire sectio	0	1
920	0	40,14	vaginaal	0	1
921	0	38	vaginaal	0	1
922	0	39,72	Repeat sectio	0	1
923	0	39,72	Repeat sectio	0	1

924	0	40,28	sec.sectio	0	1
925	0	38,43	Vaginale partus	0	1
926	0	38,43	Vaginale partus	0	1
927	0	39,57	Vaginale partus	1	1
928	0	39,57	Vaginale partus	1	1
929	0	40	Vaginale partus	1	1
930	0	40	Vaginale partus	1	1
931	0	38,86	Secundaire sectio	1	1
932	0	38,14	Secundaire sectio	0	1
933	0	38,14	Secundaire sectio	0	1
934	0	39,43	Secundaire sectio	1	1
935	0	39,43	Secundaire sectio	1	1
936	0	40,14	Vaginale partus	1	1
937	0	40,14	Vaginale partus	1	1
938	0	41,14	Secundaire sectio	1	1
939	0	41,14	Secundaire sectio	1	1
940	0	39,57	Vaginale partus	0	1
941	0	39,57	Vaginale partus	0	1
942	0	40,43	Vaginale partus	0	1
943	0	40,43	Vaginale partus	0	1
944	0	40,14	vaginaal	0	1
945	0	38,14	Vaginale partus	1	1
946	0	38,14	Vaginale partus	1	1
947	0	38,72	Vaginale partus	0	1
948	0	38,43	Primaire sectio	0	1
949	0	38,43	Primaire sectio	0	1
950	0	40,72	Vaginale partus	0	1
951	0	40,72	Vaginale partus	0	1
952	0	41,43	vaginaal	0	1
953	0	41,28	Secundaire sectio	0	1
954	0	41,28	Vaginale partus	0	1
955	0	41,28	Vaginale partus	0	1
956	0	40,72	Vaginale partus	0	1

957	0	40,72	Vaginale partus	0	1
958	0	41,43	Vaginale partus	0	1
959	0	39,72	Vaginale partus	0	1
960	0	39,72	Vaginale partus	0	1
961	0	37,57	Secundaire sectio	0	1
962	0	37,57	Secundaire sectio	0	1
963	0	39,14	Vaginale partus	0	1
964	0	39,14	Vaginale partus	0	1
965	0	39,86	Vaginale partus	2	1
966	0	39,86	Vaginale partus	2	1
967	0	39,86	Vaginale partus	2	1
968	0	40,28	Vaginale partus	0	1
969	0	40,28	Vaginale partus	0	1
970	0	39,14	Vaginale partus	0	1
971	0	39,14	Vaginale partus	0	1
972	0	40,28	vaginaal	0	1
973	0	40,14	vaginaal	0	1
974	0	39,86	Vaginale partus	0	1
975	0	39,86	Vaginale partus	0	1
976	0	40,43	Vaginale partus	0	1
977	0	40,43	Vaginale partus	0	1
978	0	40	vaginaal	0	1
979	0	40,43	Vaginale partus	0	1
980	0	40,28	vaginaal	0	1
981	0	37	Secundaire sectio	2	1
982	0	37	Secundaire sectio	2	1
983	0	39	Vaginale partus	0	1
984	0	39	Vaginale partus	0	1
985	0	41	Vaginale partus	0	1
986	0	41	Vaginale partus	0	1
987	0	41,28	Secundaire sectio	1	1
988	0	41,28	Secundaire sectio	1	1
989	0	41	Vaginale partus	0	1

990	0	41	Vaginale partus	0	1
991	0	35,43	Secundaire sectio	2	1
992	0	35,43	Secundaire sectio	2	1
993	0	36	Vaginale partus	0	1
994	0	37	Secundaire sectio	2	1
995	0	37	Secundaire sectio	2	1
996	0	37	Secundaire sectio	2	1
997	0	40	Vaginale partus	0	1
998	0	40	Vaginale partus	0	1
999	0	38,57	vaginaal	1	1
1000	0	39,28	vaginaal	0	1
1001	0	39	prim.sectio	0	1
1002	0	41,14	vaginaal	0	1
1003	0	40,72	Vaginale partus	0	1
1004	0	40,72	Vaginale partus	0	1
1005	0	40,72	Vaginale partus	0	1
1006	0	38,43	Vaginale partus	0	1
1007	0	38,43	Vaginale partus	0	1
1008	0	38,43	Vaginale partus	0	1
1009	0	41,14	Vaginale partus	0	1
1010	0	41,14	Vaginale partus	0	1
1011	0	40,28	Vaginale partus	0	1
1012	0	40,28	Vaginale partus	0	1
1013	0	39,86	Vaginale partus	1	1
1014	0	39,86	Vaginale partus	1	1
1015	0	35	vaginaal	0	1
1016	0	31,86	Vaginale partus	1	1
1017	0	31,86	Vaginale partus	1	1
1018	0	37,57	vaginaal	0	1
1019	0	40,72	Vaginale partus	0	1
1020	0	40,72	Vaginale partus	0	1
1021	0	39,14	prim.sectio	1	1
1022	0	40,43	Vaginale partus	0	1

1023	0	40,43	Vaginale partus	0	1
1024	0	38,57	Vaginale partus	0	1
1025	0	40	vaginaal	0	1
1026	0	40,86	sec.sectio	0	1
1027	0	40	Vaginale partus	0	1
1028	0	40	Vaginale partus	0	1
1029	0	40,14	Vaginale partus	0	1
1030	0	40,14	Vaginale partus	0	1
1031	0	39,28	Vaginale partus	0	1
1032	0	39,28	Vaginale partus	0	1
1033	0	39,28	Vaginale partus	0	1
1034	0	37,14	Vaginale partus	0	1
1035	0	37,14	Vaginale partus	0	1
1036	0	41,14	Vaginale partus	0	1
1037	0	40,57	Vaginale partus	0	1
1038	0	39,14	vaginaal	0	1
1039	0	40,86	Secundaire sectio	0	1
1040	0	40,86	Secundaire sectio	0	1
1041	0	38	Vaginale partus	0	1
1042	0	38	Vaginale partus	0	1
1043	0	38,57	Repeat sectio	0	1
1044	0	38,57	Repeat sectio	0	1
1045	0	40,86	Vaginale partus	0	1
1046	0	40,86	Vaginale partus	0	1
1047	0	40,86	Vaginale partus	0	1
1048	0	37,14	sec.sectio	0	1
1049	0	39	Primaire sectio	0	1
1050	0	39	Primaire sectio	0	1
1051	0	40,14	vaginaal	0	1
1052	0	40,57	Vaginale partus	0	1
1053	0	40,57	Vaginale partus	0	1
1054	0	39,57	Vaginale partus	2	1
1055	0	39,57	Vaginale partus	2	1

1056	0	40,86	vaginaal	1	1
1057	0	37,28	Secundaire sectio	1	1
1058	0	37,28	Secundaire sectio	1	1
1059	0	40,28	Vaginale partus	0	1
1060	0	40,28	Vaginale partus	0	1
1061	0	40,28	Vaginale partus	0	1
1062	0	38	Vaginale partus	1	1
1063	0	38	Vaginale partus	1	1
1064	0	38,57	Vaginale partus	0	1
1065	0	38,43	Vaginale partus	2	1
1066	0	38,43	Vaginale partus	2	1
1067	0	38,43	Vaginale partus	2	1
1068	0	41,28	Vaginale partus	1	1
1069	0	41,28	Vaginale partus	1	1
1070	0	41,28	Vaginale partus	1	1
1071	0	39,72	Vaginale partus	1	1
1072	0	39,72	Vaginale partus	1	1
1073	0	40,43	vaginaal	0	1
1074	0	39,86	vaginaal	0	1
1075	0	39	Vaginale partus	1	1
1076	0	39	Vaginale partus	1	1
1077	0	40,86	vaginaal	1	1
1078	0	38,72	Vaginale partus	0	1
1079	0	37,28	Vaginale partus	1	1
1080	0	37,28	Vaginale partus	1	1
1081	0	41,43	vaginaal	1	1
1082	0	39,72	Vaginale partus	1	1
1083	0	39,72	Vaginale partus	1	1
1084	0	41,57	Vaginale partus	0	1
1085	0	41,57	Vaginale partus	0	1
1086	0	41,43	Vaginale partus	0	1
1087	0	41,43	Vaginale partus	0	1
1088	0	40,57	Vaginale partus	0	1

1089	0	38	prim.sectio	1	1
1090	0	41	Vaginale partus	0	1
1091	0	41	Vaginale partus	0	1
1092	0	40	vaginaal	0	1
1093	0	40,43	Vaginale partus	1	1
1094	0	37,28	sectio	1	1
1095	0	38,86	Repeat sectio	1	1
1096	0	38,86	Repeat sectio	1	1
1097	0	40	Vaginale partus	0	1
1098	0	40	Vaginale partus	0	1
1099	0	39,57	Vaginale partus	1	1
1100	0	39,57	Vaginale partus	1	1
1101	0	39,57	Vaginale partus	1	1
1102	0	37,43	prim.sectio	2	1
1103	0	38,57	Primaire sectio	0	1
1104	0	38,57	Primaire sectio	0	1
1105	0	38,57	Primaire sectio	0	1
1106	0	41,14	Secundaire sectio	0	1
1107	0	41,14	Secundaire sectio	0	1
1108	0	39	Primaire sectio	1	1
1109	0	39	Primaire sectio	1	1
1110	0	37,86	Vaginale partus	1	1
1111	0	37,86	Vaginale partus	1	1
1112	0	37,86	Vaginale partus	1	1
1113	0	40,14	sec.sectio	2	1
1114	0	39	Vaginale partus	1	1
1115	0	39	Vaginale partus	1	1
1116	0	40,57	vaginaal	0	1
1117	0	38,43	Primaire sectio	0	1
1118	0	38,43	Primaire sectio	0	1
1119	0	38,43	Primaire sectio	0	1
1120	0	39,57	vaginaal	2	1
1121	0	39,72	Vaginale partus	0	1

1122	0	39,72	Vaginale partus	0	1
1123	0	38,14	Vaginale partus	0	1
1124	0	38,14	Vaginale partus	0	1
1125	0	37	Vaginale partus	0	1
1126	0	37	Vaginale partus	0	1
1127	0	40,43	vaginaal	0	1
1128	0	39	Vaginale partus	0	1
1129	0	40	vaginaal	0	1
1130	0	39	Vaginale partus	0	1
1131	0	38,43	Vaginale partus	0	1
1132	0	39,28	Vaginale partus	0	1
1133	0	39,28	Vaginale partus	0	1
1134	0	38,43	Vaginale partus	0	1
1135	0	40,57	Vaginale partus	0	1
1136	0	40,57	Vaginale partus	0	1
1137	0	40,57	Vaginale partus	0	1
1138	0	40,28	Vaginale partus	0	1
1139	0	40,28	Vaginale partus	0	1
1140	0	39,72	vaginaal	0	1
1141	0	41,14	Vaginale partus	0	1
1142	0	35,57	Vaginale partus	0	1
1143	0	39,72	Vaginale partus	0	1
1144	0	39,57	Vaginale partus	0	1
1145	0	35,57	Vaginale partus	0	1
1146	0	39,72	Vaginale partus	0	1
1147	0	39,57	Vaginale partus	0	1
1148	0	39,72	Vaginale partus	0	1
1149	0	41	Vaginale partus	0	1
1150	0	41	Vaginale partus	0	1
1151	0	39,43	Vaginale partus	0	1
1152	0	39,43	Vaginale partus	0	1
1153	0	37,43	Vaginale partus	0	1
1154	0	37,57	sec.sectio	0	1

1155	0	40,57	Vaginale partus	0	1
1156	0	40,43	Vaginale partus	0	1
1157	0	40,57	Vaginale partus	0	1
1158	0	40,43	Vaginale partus	0	1
1159	0	39,43	Vaginale partus	0	1
1160	0	39,43	Vaginale partus	0	1
1161	0	39,28	vaginaal	0	1
1162	0	40,14	Vaginale partus	0	1
1163	0	40,14	Vaginale partus	0	1
1164	0	39,57	vaginaal	0	1
1165	0	39,72	vaginaal	0	1
1166	0	40,57	vaginaal	0	1
1167	0	37,43	Vaginale partus	0	1
1168	0	39,14	vaginaal	0	1
1169	0	40,28	Vaginale partus	0	1
1170	0	40,28	Vaginale partus	0	1
1171	0	40	Vaginale partus	0	1
1172	0	38,43	Vaginale partus	0	1
1173	0	40	Vaginale partus	0	1
1174	0	38,43	Vaginale partus	0	1
1175	0	40,86	Vaginale partus	0	1
1176	0	41	Vaginale partus	0	1
1177	0	39,72	Vaginale partus	0	1
1178	0	39,72	Vaginale partus	0	1
1179	0	40,86	Vaginale partus	0	1
1180	0	39,14	vaginaal	0	1
1181	0	40,28	Vaginale partus	0	1
1182	0	40,28	Vaginale partus	0	1
1183	0	38,86	Vaginale partus	0	1
1184	0	37,43	Vaginale partus	0	1
1185	0	39,86	Vaginale partus	0	1
1186	0	38,86	Vaginale partus	0	1
1187	0	35,86	Vaginale partus	0	1

1188	0	37,43	Vaginale partus	0	1
1189	0	40,28	Vaginale partus	0	1
1190	0	35,86	Vaginale partus	0	1
1191	0	39,86	Vaginale partus	0	1
1192	0	38,86	Vaginale partus	0	1
1193	0	40,28	Vaginale partus	0	1
1194	0	38,86	Vaginale partus	0	1
1195	0	40,28	Vaginale partus	0	1
1196	0	38,86	Vaginale partus	0	1
1197	0	39,14	vaginaal	0	1
1198	0	39,28	Vaginale partus	0	1
1199	0	39,28	Vaginale partus	0	1
1200	0	41,43	sec.sectio	0	1
1201	0	38,28	vaginaal	0	1
1202	0	41,28	vaginaal	0	1
1203	0	41,14	Secundaire sectio	0	1
1204	0	41,14	Secundaire sectio	0	1
1205	0	41,14	Secundaire sectio	0	1
1206	0	40,86	Vaginale partus	0	1
1207	0	40,86	Vaginale partus	0	1
1208	0	35,86	Vaginale partus	0	1
1209	0	41	Vaginale partus	0	1
1210	0	40,86	Vaginale partus	0	1
1211	0	41	Vaginale partus	0	1
1212	0	36	vaginaal	0	1
1213	0	39,86	Vaginale partus	0	1
1214	0	40,14	Vaginale partus	0	1
1215	0	40	Vaginale partus	0	1
1216	0	39,86	Vaginale partus	0	1
1217	0	40	vaginaal	0	1
1218	0	40	Vaginale partus	0	1
1219	0	39	Vaginale partus	0	1
1220	0	40,14	Vaginale partus	0	1

1221	0	40,28	Vaginale partus	0	1
1222	0	41,28	vaginaal	0	1
1223	0	40,28	Vaginale partus	0	1
1224	0	40,43	Vaginale partus	0	1
1225	0	39,86	vaginaal	0	1
1226	0	40,43	Vaginale partus	0	1
1227	0	39,43	Vaginale partus	0	1
1228	0	39,72	Vaginale partus	0	1
1229	0	39,72	Vaginale partus	0	1
1230	0	39	prim.sectio	0	1
1231	0	39,57	Primaire sectio	0	1
1232	0	39,57	Primaire sectio	0	1
1233	0	40,86	vaginaal	0	1
1234	0	39,28	vaginaal	0	1
1235	0	38	Vaginale partus	0	1
1236	0	38	Vaginale partus	0	1
1237	0	39,57	Vaginale partus	0	1
1238	0	39,57	Vaginale partus	0	1
1239	0	38,43	Primaire sectio	0	1
1240	0	38,43	Primaire sectio	0	1
1241	0	39,28	vaginaal	0	1
1242	0	40,86	Vaginale partus	0	1
1243	0	40,86	Vaginale partus	0	1
1244	0	38,57	sec.sectio	0	1
1245	0	39	vaginaal	0	1
1246	0	39,72	Vaginale partus	0	1
1247	0	39,72	Vaginale partus	0	1
1248	0	39,43	vaginaal	0	1
1249	0	37,14	Vaginale partus	0	1
1250	0	37,14	Secundaire sectio	0	1
1251	0	37,14	Secundaire sectio	0	1
1252	0	39,72	Vaginale partus	0	1
1253	0	39,72	Vaginale partus	0	1

1254	0	40,72	Vaginale partus	0	1
1255	0	40,72	Vaginale partus	0	1
1256	0	39,28		0	1
1257	0	40	Vaginale partus	0	1
1258	0	40	Vaginale partus	0	1
1259	0	40,43	vaginaal	0	1
1260	0	39,14	Vaginale partus	0	1
1261	0	39,14	Vaginale partus	0	1
1262	0	39,57	vaginaal	0	1
1263	0	40,14	vaginaal	0	1
1264	0	38,28	Vaginale partus	0	1
1265	0	38,28	Vaginale partus	0	1
1266	0	40,56	Vaginale partus	0	1
1267	0	38,86	Vaginale partus	0	1
1268	0	38,86	Vaginale partus	0	1
1269	0	40,43	vaginaal	0	1
1270	0	41,28	vaginaal	0	1
1271	0	39,57	vaginaal	0	1
1272	0	39,14	Vaginale partus	0	1
1273	0	39,14	Vaginale partus	0	1
1274	0	39,43	Vaginale partus	0	1
1275	0	39,43	Vaginale partus	0	1
1276	0	41,14	vaginaal	0	1
1277	0	39,43	Secundaire sectio	0	1
1278	0	39,43	Secundaire sectio	0	1
1279	0	40,14	Vaginale partus	0	1
1280	0	40,14	Vaginale partus	0	1
1281	0	40,14	Vaginale partus	0	1
1282	0	41,14	Vaginale partus	0	1
1283	0	41,14	Vaginale partus	0	1
1284	0	39,57	vaginaal	0	1
1285	0	40,72	Vaginale partus	0	1
1286	0	40,72	Vaginale partus	0	1

1287	0	39,86	Vaginale partus	0	1
1288	0	39,86	Vaginale partus	0	1
1289	0	40,72	Vaginale partus	0	1
1290	0	40,72	Vaginale partus	0	1
1291	0	39,28	Primaire sectio	0	1
1292	0	39,28	Primaire sectio	0	1
1293	0	39,28	Primaire sectio	0	1
1294	0	39	Primaire sectio	0	1
1295	0	39	Primaire sectio	0	1
1296	0	37,28	prim.sectio	0	1
1297	0	39,72	sec.sectio	0	1
1298	0	38,57	Primaire sectio	0	1
1299	0	38,57	Primaire sectio	0	1
1300	0	38,86	vaginaal	0	1
1301	0	39	vaginaal	0	1
1302	0	41,43	Vaginale partus	0	1
1303	0	41,43	Vaginale partus	0	1
1304	0	38,86	Vaginale partus	0	1
1305	0	38,86	Vaginale partus	0	1
1306	0	38,72	Vaginale partus	0	1
1307	0	38,72	Vaginale partus	0	1
1308	0	38,72	Vaginale partus	0	1
1309	0	38,28	Vaginale partus	0	1
1310	0	38,28	Vaginale partus	0	1
1311	0	40	vaginaal	0	1
1312	0	39,72	Vaginale partus	0	1
1313	0	39,72	Vaginale partus	0	1
1314	0	41,14	Vaginale partus	0	1
1315	0	41,14	Vaginale partus	0	1
1316	0	41,14	Vaginale partus	0	1
1317	0	41,14	Vaginale partus	0	1
1318	0	40,14	Vaginale partus	2	1
1319	0	40,14	Vaginale partus	2	1

1320	0	40,86		1	1
1321	0	40,86		1	1
1322	0	40,28	Vaginale partus	1	1
1323	0	40,57	Vaginale partus	1	1
1324	0	41	Vaginale partus	0	1
1325	0	41	Vaginale partus	0	1
1326	0	39,43	Vaginale partus	0	1
1327	0	39,43	Vaginale partus	0	1
1328	0	40,86	vaginaal	0	1
1329	0	41,14	vaginaal	0	1
1330	0	40	vaginaal	0	1
1331	0	39,72	vaginaal	0	1
1332	0	40,43	Vaginale partus	0	1
1333	0	40,43	Vaginale partus	0	1
1334	0	38,43	sec.sectio	0	1
1335	0	39,57	Vaginale partus	1	1
1336	0	39,57	Vaginale partus	1	1
1337	0	39,72	Vaginale partus	1	1
1338	0	39,72	Vaginale partus	1	1
1339	0	40,72	Vaginale partus	0	1
1340	0	40,72	Vaginale partus	0	1
1341	0	39,43	Primaire sectio	0	1
1342	0	39,43	Primaire sectio	0	1
1343	0	38,43		0	1
1344	0	38,43		0	1
1345	0	40,72	Vaginale partus	0	1
1346	0	40,72	Vaginale partus	0	1
1347	0	40	Vaginale partus	0	1
1348	0	40	Vaginale partus	0	1
1349	0	39	Vaginale partus	0	1
1350	0	40,14	Vaginale partus	0	1
1351	0	40,14	Vaginale partus	0	1
1352	0	40,43	Vaginale partus	2	1

1353	0	40,43	Vaginale partus	2	1
1354	0	40,43	Vaginale partus	2	1
1355	0	39,28	Secundaire sectio	2	1
1356	0	39,28	Secundaire sectio	2	1
1357	0	39,28	Secundaire sectio	2	1
1358	0	40,14	Vaginale partus	0	1
1359	0	40,14	Vaginale partus	0	1
1360	0	41,14	Vaginale partus	0	1
1361	0	41,14	Vaginale partus	0	1
1362	0	39,57	vaginaal	0	1
1363	0	41,43	Vaginale partus	0	1
1364	0	41,43	Vaginale partus	0	1
1365	0	37,57	Vaginale partus	0	1
1366	0	37,57	Vaginale partus	0	1
1367	0	39,28	Vaginale partus	1	1
1368	0	39,28	Vaginale partus	1	1
1369	0	40,14	Vaginale partus	1	1
1370	0	40,14	Vaginale partus	1	1
1371	0	41,28	vaginaal	1	1
1372	0	40,57	Vaginale partus	1	1
1373	0	40,57	Vaginale partus	1	1
1374	0	40,14	Vaginale partus	1	1
1375	0	40,14	Vaginale partus	1	1
1376	0	40,14	Vaginale partus	0	1
1377	0	40,14	Vaginale partus	0	1
1378	0	38,28	Vaginale partus	1	1
1379	0	38,28	Vaginale partus	1	1
1380	0	40,57	Vaginale partus	0	1
1381	0	39,57	vaginaal	0	1
1382	0	37,57	Vaginale partus	0	1
1383	0	37,57	Vaginale partus	0	1
1384	0	39,86	Vaginale partus	0	1
1385	0	39,86	Vaginale partus	0	1

1386	0	40	Vaginale partus	2	1
1387	0	40	Vaginale partus	2	1
1388	0	40	Vaginale partus	2	1
1389	0	38,57	Primaire sectio	0	1
1390	0	38,57	Primaire sectio	0	1
1391	0	39,43	Vaginale partus	1	1
1392	0	39,43	Vaginale partus	1	1
1393	0	41	Vaginale partus	0	1
1394	0	41	Vaginale partus	0	1
1395	0	39	prim.sectio	0	1
1396	0	40,28	Vaginale partus	0	1
1397	0	40,28	Vaginale partus	0	1
1398	0	40,28	Vaginale partus	0	1
1399	0	38,72	Vaginale partus	0	1
1400	0	38,72	Vaginale partus	0	1
1401	0	40	vaginaal	1	1
1402	0	37,57	Primaire sectio	0	1
1403	0	37,57	Primaire sectio	0	1
1404	0	39,43	vaginaal	0	1
1405	0	37,86	prim.sectio	0	1
1406	0	40	vaginaal	0	1
1407	0	39,28	Vaginale partus	0	1
1408	0	39,28	Vaginale partus	0	1
1409	0	39,14	Vaginale partus	0	1
1410	0	39,14	Vaginale partus	0	1
1411	0	36,72	sec.sectio	0	1
1412	0	38,14	Vaginale partus	1	1
1413	0	38,14	Vaginale partus	1	1
1414	0	37,86	Vaginale partus	0	1
1415	0	37,86	Vaginale partus	0	1
1416	0	39,28	Vaginale partus	0	1
1417	0	37,43	Vaginale partus	1	1
1418	0	37,43	Vaginale partus	1	1

1419	0	37,72	vaginaal	1	1
1420	0	34,86	Secundaire sectio	0	1
1421	0	35,86	vaginaal	0	1
1422	0	36,86	Vaginale partus	0	1
1423	0	36,86	Vaginale partus	0	1
1424	0	34,14	Vaginale partus	0	1
1425	0	34,14	Vaginale partus	0	1
1426	0	36,43	Secundaire sectio	1	1
1427	0	36,43	Secundaire sectio	1	1
1428	0	36,86	Vaginale partus	1	1
1429	0	36,86	Vaginale partus	1	1
1430	0	34,86	Secundaire sectio	1	1
1431	0	34,86	Secundaire sectio	1	1
1432	0	35,57	Vaginale partus	0	1
1433	0	35,57	Vaginale partus	0	1
1434	0	36	Vaginale partus	0	1
1435	0	36	Vaginale partus	0	1
1436	0	36,14	Vaginale partus	0	1
1437	0	36,14	Vaginale partus	0	1
1438	0	36,14	Vaginale partus	0	1
1439	0	36,57	Vaginale partus	0	1
1440	0	36,57	Vaginale partus	0	1
1441	0	36,57	Vaginale partus	0	1
1442	0	36,57	Vaginale partus	0	1
1443	0	36,57	Vaginale partus	0	1
1444	0	36,57	Vaginale partus	0	1
1445	0	36	Vaginale partus	0	1
1446	0	36	Vaginale partus	0	1
1447	0	36	Vaginale partus	0	1
1448	0	36	Vaginale partus	0	1
1449	0	36,57	Vaginale partus	0	1
1450	0	36,57	Vaginale partus	0	1
1451	0	36,28	Vaginale partus	0	1

1452	0	36,28	Vaginale partus	0	1
1453	0	33,28	vaginaal	0	1
1454	0	30,43	Vaginale partus	0	1
1455	1	33,72	Vaginale partus	2	1
1456	1	33,72	Vaginale partus	2	1
1457	1	33,72	Vaginale partus	2	1
1458	0	38,14	Vaginale partus	0	1
1459	0	38,14	Vaginale partus	0	1
1460	0	31,14	Secundaire sectio	0	1
1461	0	31,14	Secundaire sectio	0	1
1462	0	32,86	Secundaire sectio	0	1
1463	0	33,86	Secundaire sectio	0	1
1464	0	33,86	Secundaire sectio	0	1
1465	0	35,86	Vaginale partus	0	1
1466	0	35,86	Vaginale partus	0	1
1467	0	32,72	Vaginale partus	2	1
1468	0	32,72	Vaginale partus	2	1
1469	0	32,72	Vaginale partus	2	1
1470	0	29,72	vaginaal	0	1
1471	0	38,72	vaginaal	0	1
1472	0	40,86	vaginaal	0	1
1473	0	40,14	prim.sectio	2	1
1474	0	38,57	Vaginale partus	0	1
1475	0	40,14	Vaginale partus	0	1
1476	0	40,14	Vaginale partus	0	1
1477	0	38,28	Primaire sectio	1	1
1478	0	39,57	Vaginale partus	0	1
1479	0	38,57	Vaginale partus	0	1
1480	0	39,57	Vaginale partus	0	1
1481	0	38,43	Vaginale partus	0	1
1482	0	38,43	Vaginale partus	0	1
1483	0	38,43	Vaginale partus	0	1
1484	0	38,43	Vaginale partus	0	1

1485	0	38,14	Secundaire sectio	0	1
1486	0	39,28	Vaginale partus	0	1
1487	0	39,28	Vaginale partus	0	1
1488	0	39,43	Vaginale partus	0	1
1489	0	41	Vaginale partus	0	1
1490	0	40,14	Vaginale partus	0	1
1491	0	40,14	Vaginale partus	0	1
1492	0	39,86	Vaginale partus	0	1
1493	0	40,28	Vaginale partus	0	1
1494	0	40,28	Vaginale partus	0	1
1495	0	39,86	Vaginale partus	0	1
1496	0	38,43	Repeat sectio	0	1
1497	0	38,43	Repeat sectio	0	1
1498	0	39	Vaginale partus	0	1
1499	0	39	Vaginale partus	0	1
1500	0	38,57	Vaginale partus	0	1
1501	0	38,57	Vaginale partus	0	1
1502	0	38,57	Vaginale partus	0	1
1503	0	38,43	Vaginale partus	0	1
1504	0	39,57	Repeat sectio	0	1
1505	0	38,43	Vaginale partus	0	1
1506	0	38,72	Vaginale partus	0	1
1507	0	38,72	Vaginale partus	0	1
1508	0	41	Vaginale partus	0	1
1509	0	40	Vaginale partus	0	1
1510	0	40,72	Vaginale partus	0	1
1511	0	40	Vaginale partus	0	1
1512	0	41	Vaginale partus	0	1
1513	0	38	Vaginale partus	0	1
1514	0	40,57	Vaginale partus	0	1
1515	0	40,57	Vaginale partus	0	1
1516	0	38,14	Vaginale partus	0	1
1517	0	38	Vaginale partus	0	1

1518	0	38,14	Vaginale partus	0	1
1519	0	41	Vaginale partus	0	1
1520	0	41,14		0	1
1521	0	41,14		0	1
1522	0	39,72	Vaginale partus	0	1
1523	0	36,57	Vaginale partus	0	1
1524	0	36,57	Vaginale partus	0	1
1525	0	40,43	Vaginale partus	0	1
1526	0	38,72	Vaginale partus	0	1
1527	0	40,43	Vaginale partus	0	1
1528	0	38,72	Vaginale partus	0	1
1529	0	40,14	Secundaire sectio	0	1
1530	0	40,14	Secundaire sectio	0	1
1531	0	39,43	Vaginale partus	0	1
1532	0	39,14	Primaire sectio	0	1
1533	0	39,14	Primaire sectio	0	1
1534	0	39,86	Vaginale partus	0	1
1535	0	39,86	Vaginale partus	0	1
1536	0	40,43	Vaginale partus	0	1
1537	0	38,14	Vaginale partus	0	1
1538	0	38,14	Vaginale partus	0	1
1539	0	38,57	Vaginale partus	0	1
1540	0	40	Vaginale partus	0	1
1541	0	40	Vaginale partus	0	1
1542	0	38,57	Vaginale partus	0	1
1543	0	40,43	Vaginale partus	0	1
1544	0	39,28	Vaginale partus	0	1
1545	0	39,57	Vaginale partus	0	1
1546	0	39,28	Vaginale partus	0	1
1547	0	41,43	Vaginale partus	0	1
1548	0	41,43	Vaginale partus	0	1
1549	0	39,86	Primaire sectio	0	1
1550	0	38,14	Vaginale partus	0	1

1551	0	38,14	Vaginale partus	0	1
1552	0	39,14	Vaginale partus	0	1
1553	0	39,14	Vaginale partus	0	1
1554	0	41,57	Secundaire sectio	0	1
1555	0	41,57	Secundaire sectio	0	1
1556	0	40,57	Vaginale partus	0	1
1557	0	36	Vaginale partus	0	1
1558	0	36	Vaginale partus	0	1
1559	0	40,57	Vaginale partus	0	1
1560	0	38,14	Secundaire sectio	0	1
1561	0	39,43	Vaginale partus	0	1
1562	0	38,14	Vaginale partus	0	1
1563	0	38,14	Vaginale partus	0	1
1564	0	39,43	Vaginale partus	0	1
1565	0	40	Vaginale partus	0	1
1566	0	40	Vaginale partus	0	1
1567	0	40,86	Vaginale partus	0	1
1568	0	40,86	Vaginale partus	0	1
1569	0	38,28	Primaire sectio	0	1
1570	0	39,43	Vaginale partus	0	1
1571	0	41,57	Vaginale partus	0	1
1572	0	37,43	Vaginale partus	0	1
1573	0	40,14	Secundaire sectio	0	1
1574	0	39,43	Vaginale partus	0	1
1575	0	37,43	Vaginale partus	0	1
1576	0	41,57	Vaginale partus	0	1
1577	0	40,14	Secundaire sectio	0	1
1578	0	40	Vaginale partus	0	1
1579	0	40	Vaginale partus	0	1
1580	0	35,43	Vaginale partus	0	1
1581	0	35,43	Vaginale partus	0	1
1582	0	40	Vaginale partus	0	1
1583	0	39,86	Vaginale partus	0	1

1584	0	39,86	Vaginale partus	0	1
1585	0	37,28	Vaginale partus	0	1
1586	0	40	Vaginale partus	0	1
1587	0	37,28	Vaginale partus	0	1
1588	0	40,72	Vaginale partus	0	1
1589	0	40,72	Vaginale partus	0	1
1590	0	39,86	Vaginale partus	0	1
1591	0	39,86	Vaginale partus	0	1
1592	0	39,86	Vaginale partus	0	1
1593	0	39,86	Vaginale partus	0	1
1594	0	40	Vaginale partus	0	1
1595	0	40	Vaginale partus	0	1
1596	0	38,72	Primaire sectio	0	1
1597	0	41	Vaginale partus	0	1
1598	0	38,28	Vaginale partus	0	1
1599	0	40,72	Vaginale partus	0	1
1600	0	40,72	Vaginale partus	0	1
1601	0	38,86	Repeat sectio	0	1
1602	0	38,86	Repeat sectio	0	1
1603	0	38,28	Vaginale partus	0	1
1604	0	39,57	Vaginale partus	0	1
1605	0	40,28	Vaginale partus	0	1
1606	0	35,57	Vaginale partus	0	1
1607	0	38,14	Primaire sectio	0	1
1608	0	40,28	Vaginale partus	0	1
1609	0	40,57	Vaginale partus	0	1
1610	0	39,72	Vaginale partus	0	1
1611	0	39,72	Vaginale partus	0	1
1612	0	38,86	Vaginale partus	0	1
1613	0	35,57	Vaginale partus	0	1
1614	0	40,57	Vaginale partus	0	1
1615	0	38,14	Primaire sectio	0	1
1616	0	38,86	Vaginale partus	0	1

1617	0	39,86	Vaginale partus	0	1
1618	0	39,86	Vaginale partus	0	1
1619	0	41,14	Secundaire sectio	0	1
1620	0	41,14	Secundaire sectio	0	1
1621	0	38,86	Primaire sectio	0	1
1622	0	38,86	Primaire sectio	0	1
1623	0	41,28	Vaginale partus	0	1
1624	0	38	Vaginale partus	0	1
1625	0	41,28	Vaginale partus	0	1
1626	0	39,86	Vaginale partus	0	1
1627	0	40,86	Vaginale partus	0	1
1628	0	39,86	Vaginale partus	0	1
1629	0	38,86	Vaginale partus	0	1
1630	0	38,86	Vaginale partus	0	1
1631	0	39,14	Vaginale partus	0	1
1632	0	40,14	Vaginale partus	0	1
1633	0	40,14	Vaginale partus	0	1
1634	0	37,57	Vaginale partus	0	1
1635	0	39,14	Vaginale partus	0	1
1636	0	37,57	Vaginale partus	0	1
1637	0	40	Vaginale partus	0	1
1638	0	41,43	Vaginale partus	0	1
1639	0	41,43	Vaginale partus	0	1
1640	0	40,43	Vaginale partus	0	1
1641	0	40,72	Vaginale partus	0	1
1642	0	40,43	Vaginale partus	0	1
1643	0	39,14	Vaginale partus	0	1
1644	0	39,72	Vaginale partus	0	1
1645	0	39,72	Vaginale partus	0	1
1646	0	39,14	Vaginale partus	0	1
1647	0	40,86	Vaginale partus	0	1
1648	0	40,86	Vaginale partus	0	1
1649	0	39,28	Secundaire sectio	0	1

1650	0	39,28	Secundaire sectio	0	1
1651	0	39,86	Primaire sectio	0	1
1652	0	39,86	Primaire sectio	0	1
1653	0	39	Vaginale partus	0	1
1654	0	40	Vaginale partus	0	1
1655	0	39,57	Vaginale partus	0	1
1656	0	41,43	Vaginale partus	0	1
1657	0	40	Vaginale partus	0	1
1658	0	39,57	Vaginale partus	0	1
1659	0	41,43	Vaginale partus	0	1
1660	0	39	Vaginale partus	0	1
1661	0	39,57	Repeat sectio	0	1
1662	0	40,86	Vaginale partus	0	1
1663	0	40,86	Vaginale partus	0	1
1664	0	39	Vaginale partus	1	1
1665	0	39	Vaginale partus	1	1
1666	0	40,28	Vaginale partus	1	1
1667	0	40,28	Vaginale partus	1	1
1668	0	41,14	Vaginale partus	2	1
1669	0	41,14	Vaginale partus	2	1
1670	0	39	Vaginale partus	2	1
1671	0	39	Vaginale partus	2	1
1672	0	39,86	Vaginale partus	1	1
1673	0	39,86	Vaginale partus	1	1
1674	0	39,57	Vaginale partus	1	1
1675	0	39,57	Vaginale partus	1	1
1676	0	40,57	Secundaire sectio	1	1
1677	0	39,72	Secundaire sectio	0	1
1678	0	39,72	Secundaire sectio	0	1
1679	0	40,86	Secundaire sectio	0	1
1680	0	40,86	Secundaire sectio	0	1
1681	0	38,28	Vaginale partus	0	1
1682	0	38,28	Vaginale partus	0	1

1683	0	40,43	Vaginale partus	0	1
1684	0	40,43	Vaginale partus	0	1
1685	1	38,43	Vaginale partus	0	1
1686	1	38,43	Vaginale partus	0	1
1687	1	41,28	Vaginale partus	2	1
1688	1	41,28	Vaginale partus	2	1
1689	0	40,72	Vaginale partus	1	1
1690	0	40,72	Vaginale partus	1	1
1691	0	40,28	Vaginale partus	1	1
1692	0	40,28	Vaginale partus	1	1
1693	0	40,28	Vaginale partus	0	1
1694	0	40,28	Vaginale partus	0	1
1695	0	40	Vaginale partus	1	1
1696	0	40	Vaginale partus	1	1
1697	0	37,86	Vaginale partus	2	1
1698	0	39,43	Primaire sectio	1	1
1699	0	39,43	Primaire sectio	1	1
1700	0	33,14	Primaire sectio	0	2
1701	0	23,14	Vaginale partus	0	2
1702	0	33,72	Primaire sectio	0	2
1703	0	26,57	Secundaire sectio	0	2
1704	0	31,28	Primaire sectio	0	2
1705	0	26,14	Primaire sectio	0	2
1706	0	27,28	Primaire sectio	0	2
1707	0	28,14	Secundaire sectio	0	2
1708	0	30	Primaire sectio	0	2
1709	0	31,57	Secundaire sectio	0	2
1710	0	32,43	Secundaire sectio	0	2
1711	0	32,86	Primaire sectio	0	2
1712	0	29	Primaire sectio	0	2
1713	0	27	Primaire sectio	0	2
1714	0	30,57	Vaginale partus	0	2
1715	0	27	Primaire sectio	0	2

1716	0	28,28	Primaire sectio	0	2
1717	0	31,57	Secundaire sectio	0	2
1718	0	31,28	Primaire sectio	0	2
1719	0	31,57	Secundaire sectio	0	2
1720	0	31,72	Secundaire sectio	0	2
1721	0	33,28	Repeat sectio	0	2
1722	0	31	Vaginale partus	0	2
1723	0	30,72	Secundaire sectio	0	2
1724	0	26,86		2	2
1725	0	33,72	Repeat sectio	2	2
1726	0	31,57	Primaire sectio	2	2
1727	0	31,86	Vaginale partus	2	2
1728	0	33,72	Vaginale partus	1	2
1729	0	28,72	Primaire sectio	2	2
1730	0	31,72	Secundaire sectio	1	2
1731	0	26,28	Primaire sectio	1	2
1732	0	33,14	Primaire sectio	2	2
1733	0	33,14	Primaire sectio	2	2
1734	0	33,43		0	2
1735	0	27,43	prim.sectio	0	2
1736	0	29,43	Vaginale partus	1	2
1737	0	33,43	Repeat sectio	2	2
1738	0	33,43	Repeat sectio	2	2
1739	0	33,43	Repeat sectio	2	2
1740	0	32,43	Primaire sectio	2	2
1741	0	32,43	Primaire sectio	2	2
1742	0	32,43	Primaire sectio	2	2
1743	0	33,57	Vaginale partus	0	2
1744	0	33,57	Vaginale partus	1	2
1745	0	27,14	Primaire sectio	0	2
1746	0	27	Primaire sectio	0	2
1747	0	27	Primaire sectio	0	2
1748	0	33,14	Secundaire sectio	0	2

1749	0	29,14	Primaire sectio	1	2
1750	0	32,72	Secundaire sectio	0	2
1751	0	32	Primaire sectio	2	2
1752	0	32	Primaire sectio	2	2
1753	0	33	vaginaal	2	2
1754	0	27,14	Vaginale partus	2	2
1755	0	27,14	Vaginale partus	2	2
1756	0	31,72		2	2
1757	0	31,72		2	2
1758	0	33,14	vaginaal	0	2
1759	0	27,14	prim.sectio	0	2
1760	0	25	vaginaal	0	2
1761	0	32,14	prim.sectio	0	2
1762	0	33,28	prim.sectio	0	2
1763	0	32,57	prim.sectio	0	2
1764	0	26,28	sec.sectio	0	2
1765	0	32,14	vaginaal	0	2
1766	0	33,28	vaginaal	0	2
1767	0	31,14	sec.sectio	0	2
1768	0	31,57	prim.sectio	0	2
1769	0	33,43	vaginaal	0	2
1770	0	33	sec.sectio	0	2
1771	0	33,72	vaginaal	0	2
1772	0	27,43	prim.sectio	0	2
1773	0	32,14	sec.sectio	0	2
1774	0	27,43	prim.sectio	0	2
1775	0	27,86	prim.sectio	0	2
1776	0	29,86	prim.sectio	0	2
1777	0	33,14	prim.sectio	0	2
1778	0	27,86	prim.sectio	0	2
1779	0	22,86		0	2
1780	0	33,86	prim.sectio	1	2
1781	0	32,57	sec.sectio	1	2

1782	0	31,14	prim.sectio	0	2
1783	0	27,43	prim.sectio	0	2
1784	0	30,28	prim.sectio	0	2
1785	0	26,28	Primaire sectio	2	2
1786	0	26,28	Primaire sectio	2	2
1787	0	34,43	Primaire sectio	0	3
1788	0	38	Secundaire sectio	0	3
1789	0	34	Primaire sectio	0	3
1790	0	35,57	Primaire sectio	0	3
1791	0	37,57	Vaginale partus	0	3
1792	0	38,86	Secundaire sectio	0	3
1793	0	39,28	Vaginale partus	0	3
1794	0	36,43	Vaginale partus	0	3
1795	0	34,86	Vaginale partus	0	3
1796	0	39,72	Repeat sectio	0	3
1797	0	39,57	Kunstverlossing	0	3
1798	0	37,28	Vaginale partus	0	3
1799	0	36	Vaginale partus	0	3
1800	0	39,57	Vaginale partus	0	3
1801	0	38,14	Vaginale partus	0	3
1802	0	40,28	Vaginale partus	0	3
1803	0	38,28	Vaginale partus	0	3
1804	0	39,72	Secundaire sectio	0	3
1805	0	36,28	Secundaire sectio	0	3
1806	0	38	Secundaire sectio	0	3
1807	0	37,14	Vaginale partus	0	3
1808	0	38,14	Vaginale partus	0	3
1809	0	35,57	Vaginale partus	0	3
1810	0	40,14	Secundaire sectio	0	3
1811	0	38,72	Vaginale partus	0	3
1812	0	39	Secundaire sectio	0	3
1813	0	39,14	Primaire sectio	0	3
1814	0	38,28	Vaginale partus	0	3

1815	0	37,86	Repeat sectio	0	3
1816	0	37,72	Primaire sectio	0	3
1817	0	38,86	Vaginale partus	0	3
1818	0	39	Vaginale partus	0	3
1819	0	38,57	Vaginale partus	0	3
1820	0	38,86	Kunstverlossing	0	3
1821	0	37	Vaginale partus	0	3
1822	0	38,43	Vaginale partus	0	3
1823	0	37,72		0	3
1824	0	35,72	Vaginale partus	0	3
1825	0	38	Vaginale partus	0	3
1826	0	37,72	Vaginale partus	0	3
1827	0	36,28	Vaginale partus	0	3
1828	0	39,86	Vaginale partus	0	3
1829	0	39	Secundaire sectio	0	3
1830	0	38,43	Repeat sectio	0	3
1831	0	39,86	Repeat sectio	0	3
1832	0	36	Vaginale partus	0	3
1833	0	37,28	Secundaire sectio	0	3
1834	0	37,28	Secundaire sectio	0	3
1835	0	38,72	Vaginale partus	0	3
1836	0	39,43	Vaginale partus	0	3
1837	0	34,14	Secundaire sectio	2	3
1838	0	37	Vaginale partus	2	3
1839	0	38,14		2	3
1840	0	38,14		2	3
1841	0	38,14	Secundaire sectio	2	3
1842	0	37,43	Vaginale partus	2	3
1843	0	37,43	Vaginale partus	2	3
1844	0	34,28	Secundaire sectio	2	3
1845	0	34,28		2	3
1846	0	37,72	Kunstverlossing	2	3
1847	0	37,28	Vaginale partus	2	3

1848	0	37	Primaire sectio	2	3
1849	0	37	Primaire sectio	2	3
1850	0	37,14		2	3
1851	0	37,14		2	3
1852	0	37,14		2	3
1853	0	41	Secundaire sectio	1	3
1854	0	39,43	Kunstverlossing	2	3
1855	0	37	Vaginale partus	2	3
1856	0	34,14	Vaginale partus	2	3
1857	0	34,72	Vaginale partus	2	3
1858	0	38,72	Vaginale partus	0	3
1859	0	37,57	Secundaire sectio	1	3
1860	0	37,57	Secundaire sectio	1	3
1861	0	37,57	Secundaire sectio	1	3
1862	0	35,57	Vaginale partus	0	3
1863	0	38,86	Vaginale partus	0	3
1864	0	40,14	Vaginale partus	1	3
1865	0	36,86	Primaire sectio	2	3
1866	0	39,14	Primaire sectio	0	3
1867	0	36,28	Repeat sectio	0	3
1868	0	37,57	Vaginale partus	0	3
1869	0	36,43	Primaire sectio	2	3
1870	0	37,72	Primaire sectio	1	3
1871	0	37,57		0	3
1872	0	39	Vaginale partus	0	3
1873	0	37,28	Vaginale partus	2	3
1874	0	34,28	Secundaire sectio	0	3
1875	0	35,72		2	3
1876	0	35,72		2	3
1877	0	35,72		2	3
1878	0	39,14	Vaginale partus	2	3
1879	0	36,86	Vaginale partus	1	3
1880	0	37		0	3

1881	0	37,28	Vaginale partus	0	3
1882	0	37,14	Vaginale partus	0	3
1883	0	39	Vaginale partus	0	3
1884	0	37,43	Secundaire sectio	0	3
1885	0	36,14	Primaire sectio	2	3
1886	0	39,72	vaginaal	2	3
1887	0	38,72	Repeat sectio	1	3
1888	0	35,86	prim.sectio	1	3
1889	0	34,86	Vaginale partus	2	3
1890	0	34,86	Vaginale partus	2	3
1891	0	34,86	Vaginale partus	2	3
1892	0	38	vaginaal	2	3
1893	0	36,43	vaginaal	2	3
1894	0	39,86	Vaginale partus	0	3
1895	0	39,86	Vaginale partus	0	3
1896	0	39,86	Vaginale partus	0	3
1897	0	39	Vaginale partus	0	3
1898	0	39	Vaginale partus	0	3
1899	0	40,57	Vaginale partus	0	3
1900	0	40,57	Vaginale partus	0	3
1901	0	40,57	Vaginale partus	0	3
1902	0	37,28	Secundaire sectio	0	3
1903	0	37,28	Secundaire sectio	0	3
1904	0	37,28	Secundaire sectio	0	3
1905	0	37,28	sec.sectio	2	3
1906	0	36,72	Secundaire sectio	2	3
1907	0	36,72	Secundaire sectio	2	3
1908	0	36,72	Secundaire sectio	2	3
1909	0	37,14		2	3
1910	0	37,14		2	3
1911	0	37,14		2	3
1912	0	36	Primaire sectio	2	3
1913	0	36	Primaire sectio	2	3

1914	0	36	Primaire sectio	2	3
1915	0	39,43	Vaginale partus	1	3
1916	0	37,57	Primaire sectio	2	3
1917	0	37,57	Primaire sectio	2	3
1918	0	37,57	Primaire sectio	2	3
1919	0	36,72	vaginaal	1	3
1920	0	36,43	Vaginale partus	2	3
1921	0	36,43	Vaginale partus	2	3
1922	0	39,86	Vaginale partus	1	3
1923	0	37,43	Primaire sectio	2	3
1924	0	37,43	Primaire sectio	2	3
1925	0	37,43	Primaire sectio	2	3
1926	0	38,14	Vaginale partus	0	3
1927	0	38,14	Vaginale partus	0	3
1928	0	40	Vaginale partus	0	3
1929	0	40	Vaginale partus	0	3
1930	0	35,14	Repeat sectio	2	3
1931	0	35,14	Repeat sectio	2	3
1932	0	35,14	Repeat sectio	2	3
1933	0	40,43	vaginaal	0	3
1934	0	35,72	prim.sectio	2	3
1935	0	37,28	vaginaal	0	3
1936	0	36,28	vaginaal	1	3
1937	0	36,57	vaginaal	1	3
1938	0	38,86	vaginaal	0	3
1939	0	36,86	vaginaal	0	3
1940	0	34	sectio	0	3
1941	0	39,28	vaginaal	0	3
1942	0	35,86	vaginaal	0	3
1943	0	37,14	vaginaal	0	3
1944	0	36,72	prim.sectio	0	3
1945	0	37,57	vaginaal	0	3
1946	0	38,72	vaginaal	0	3

1947	0	34	sec.sectio	0	3
1948	0	37,43	sec.sectio	0	3
1949	0	38	prim.sectio	0	3
1950	0	40	sec.sectio	0	3
1951	0	34,28	vaginaal	0	3
1952	0	34,86	prim.sectio	0	3
1953	0	38,72	vaginaal	0	3
1954	0	39	vaginaal	0	3
1955	0	38,43	vaginaal	0	3
1956	0	40,72	vaginaal	0	3
1957	0	36	vaginaal	0	3
1958	0	38,28	prim.sectio	0	3
1959	0	36,86	vaginaal	0	3
1960	0	38,43	vaginaal	0	3
1961	0	35	prim.sectio	0	3
1962	0	40,86	vaginaal	0	3
1963	0	40,28	vaginaal	0	3
1964	0	40,28	sec.sectio	0	3
1965	0	36,14	prim.sectio	0	3
1966	0	34,43	vaginaal	0	3
1967	0	36,43	vaginaal	0	3
1968	0	34	sec.sectio	0	3
1969	0	38,72	vaginaal	0	3
1970	0	39,14	vaginaal	0	3
1971	0	38,14	prim.sectio	0	3
1972	0	38,43	vaginaal	0	3
1973	0	36,72	vaginaal	0	3
1974	0	40,72	vaginaal	0	3
1975	0	39,28	vaginaal	0	3
1976	0	40,43	vaginaal	0	3
1977	0	40	vaginaal	0	3
1978	0	37,72	prim.sectio	0	3
1979	0	36,86	prim.sectio	0	3

1980	0	38	vaginaal	0	3
1981	0	37	vaginaal	0	3
1982	0	37,72	vaginaal	1	3
1983	1	34,57	prim.sectio	2	3
1984	0	35,43	vaginaal	0	3
1985	0	36,43	vaginaal	2	3
1986	1	34,86	vaginaal	0	3
1987	0	39,14	vaginaal	0	3
1988	0	36,57	vaginaal	0	3
1989	0	36,72	Vaginale partus	0	3
1990	0	36,72	Vaginale partus	0	3
1991	0	40	Vaginale partus	0	3
1992	0	40	Vaginale partus	0	3
1993	0	41	Secundaire sectio	0	3
1994	0	41	Secundaire sectio	0	3
1995	0	39,72	Vaginale partus	0	3
1996	0	39,72	Vaginale partus	0	3
1997	0	40,57	Vaginale partus	0	3
1998	0	39,28	Vaginale partus	0	3
1999	0	37,14	Secundaire sectio	0	3
2000	0	37,14	Secundaire sectio	0	3
2001	0	40,72	Secundaire sectio	0	3
2002	0	40,72	Secundaire sectio	0	3
2003	0	37,86	Vaginale partus	2	3
2004	0	37,86	Vaginale partus	2	3
2005	0	26,14	Vaginale partus	0	4
2006	0	37	Vaginale partus	0	4
2007	0	39	Vaginale partus	0	4
2008	0	39	Vaginale partus	0	4
2009	0	38,43	Primaire sectio	0	4
2010	0	39,14	Kunstverlossing	0	4
2011	0	37,14	Vaginale partus	0	4
2012	0	30,57	Primaire sectio	0	4

2013	0	40,72	Vaginale partus	0	4
2014	0	38,43	Vaginale partus	0	4
2015	0	40,86	Vaginale partus	0	4
2016	0	38,57	Vaginale partus	0	4
2017	0	40,86	Vaginale partus	0	4
2018	0	38,86	Vaginale partus	0	4
2019	0	39,28	Vaginale partus	0	4
2020	0	35,72	Secundaire sectio	0	4
2021	0	39,43	Vaginale partus	0	4
2022	0	39,72	Vaginale partus	0	4
2023	0	37,86	Vaginale partus	0	4
2024	0	40,43	Vaginale partus	0	4
2025	0	37,28	Repeat sectio	0	4
2026	0	38,28	Vaginale partus	0	4
2027	0	40	Vaginale partus	0	4
2028	0	37,14	Vaginale partus	0	4
2029	0	39,43	Vaginale partus	0	4
2030	0	40,43	Vaginale partus	0	4
2031	0	40,72	Vaginale partus	0	4
2032	0	40,72	Vaginale partus	0	4
2033	0	37,57	Secundaire sectio	0	4
2034	0	37,14	Vaginale partus	0	4
2035	0	39,72	Vaginale partus	0	4
2036	0	37,86	Repeat sectio	0	4
2037	0	37,86	Repeat sectio	0	4
2038	0	36,43	Vaginale partus	0	4
2039	0	38,28	Vaginale partus	0	4
2040	0	40,14	Secundaire sectio	0	4
2041	0	39,14	Secundaire sectio	0	4
2042	0	40,43	Vaginale partus	0	4
2043	0	39,14	Vaginale partus	0	4
2044	0	37,28	Vaginale partus	0	4
2045	0	38,57	Vaginale partus	0	4

2046	0	38,72	Vaginale partus	0	4
2047	0	39,28	Secundaire sectio	0	4
2048	0	39,28	Secundaire sectio	0	4
2049	0	38,72	Vaginale partus	0	4
2050	0	38,57	Vaginale partus	0	4
2051	0	39,28	Secundaire sectio	0	4
2052	0	40,57	Secundaire sectio	0	4
2053	0	38,28	Vaginale partus	0	4
2054	0	38,28	Vaginale partus	0	4
2055	0	36,72	Vaginale partus	2	4
2056	0	38,72	Primaire sectio	2	4
2057	0	38,72	Repeat sectio	2	4
2058	1	39,86	Vaginale partus	2	4
2059	0	38,72	Vaginale partus	2	4
2060	0	38,72	Vaginale partus	2	4
2061	0	38,72	Vaginale partus	2	4
2062	0	33	Primaire sectio	2	4
2063	0	38,28	Vaginale partus	2	4
2064	0	39,43	Vaginale partus	2	4
2065	0	36,86	Primaire sectio	1	4
2066	0	38,14	Repeat sectio	0	4
2067	0	40,57	Vaginale partus	0	4
2068	0	38,57	Vaginale partus	0	4
2069	0	37,86	Secundaire sectio	1	4
2070	1	34	Primaire sectio	1	4
2071	0	37,28	Vaginale partus	0	4
2072	0	37	Secundaire sectio	0	4
2073	0	37	Secundaire sectio	0	4
2074	0	40,14	Vaginale partus	2	4
2075	0	39,57	Vaginale partus	2	4
2076	0	39,14	Vaginale partus	0	4
2077	1	29,28	sectio	1	4
2078	0	41,43	Vaginale partus	0	4

2079	0	38,28	Vaginale partus	2	4
2080	0	37,28	Repeat sectio	0	4
2081	0	35,72	Vaginale partus	0	4
2082	0	36,14	Vaginale partus	2	4
2083	0	41,14	Vaginale partus	2	4
2084	0	41,14	Vaginale partus	2	4
2085	0	41,14	Vaginale partus	2	4
2086	0	39,14	Vaginale partus	2	4
2087	1	38,14	Vaginale partus	1	4
2088	1	38,14	Vaginale partus	1	4
2089	1	38,14	Vaginale partus	1	4
2090	0	39,86	Vaginale partus	2	4
2091	0	39,86	Vaginale partus	2	4
2092	0	40	vaginaal	0	4
2093	0	38,57	Vaginale partus	0	4
2094	0	38,57	Vaginale partus	0	4
2095	0	38,57	Vaginale partus	0	4
2096	0	41,28	Secundaire sectio	0	4
2097	0	41,28	Secundaire sectio	0	4
2098	0	40,72	Vaginale partus	0	4
2099	0	40,72	Vaginale partus	0	4
2100	0	39,57	Repeat sectio	0	4
2101	0	39,57	Repeat sectio	0	4
2102	0	39,57	Repeat sectio	0	4
2103	0	40,72	vaginaal	0	4
2104	0	38,72	vaginaal	2	4
2105	0	38,57	vaginaal	2	4
2106	0	37,72	Secundaire sectio	2	4
2107	0	37,72	Secundaire sectio	2	4
2108	0	37,72	Secundaire sectio	2	4
2109	0	38,72	vaginaal	2	4
2110	0	39,57	vaginaal	2	4
2111	0	39,14	Vaginale partus	0	4

2112	0	39,14	Vaginale partus	0	4
2113	0	39	Secundaire sectio	2	4
2114	0	39	Secundaire sectio	2	4
2115	0	39	Secundaire sectio	2	4
2116	0	40	sec.sectio	0	4
2117	0	36,86	Vaginale partus	0	4
2118	0	36,86	Vaginale partus	0	4
2119	0	38,43	Vaginale partus	1	4
2120	0	38,43	Vaginale partus	1	4
2121	0	38,72	Vaginale partus	2	4
2122	0	38,72	Vaginale partus	2	4
2123	0	38,86	Vaginale partus	1	4
2124	0	38,86	Vaginale partus	1	4
2125	0	38,86	Vaginale partus	1	4
2126	0	39,14	Secundaire sectio	0	4
2127	0	39,14	Secundaire sectio	0	4
2128	0	40,28	vaginaal	2	4
2129	0	40,86	Secundaire sectio	0	4
2130	0	40,86	Secundaire sectio	0	4
2131	0	40,72	Vaginale partus	1	4
2132	0	40,72	Vaginale partus	1	4
2133	0	40,72	Vaginale partus	2	4
2134	0	40,72	Vaginale partus	2	4
2135	0	40,72	Vaginale partus	2	4
2136	0	41,72	sec.sectio	0	4
2137	0	41,14	Secundaire sectio	0	4
2138	0	41,14	Secundaire sectio	0	4
2139	0	39,43	Primaire sectio	0	4
2140	0	39,43	Primaire sectio	0	4
2141	0	36,28	Vaginale partus	2	4
2142	0	36,28	Vaginale partus	2	4
2143	0	36,43	vaginaal	1	4
2144	0	33,86	Secundaire sectio	2	4

2145	0	33,86	Secundaire sectio	2	4
2146	0	40,72	vaginaal	0	4
2147	0	40	sec.sectio	0	4
2148	1	29,28	prim.sectio	0	4
2149	0	26,28	prim.sectio	0	4
2150	0	31,14	prim.sectio	0	4
2151	0	37,72	sec.sectio	0	4
2152	0	40,86	vaginaal	0	4
2153	0	40	vaginaal	0	4
2154	0	40,14	vaginaal	0	4
2155	0	39,43	vaginaal	0	4
2156	0	40,14	vaginaal	0	4
2157	0	39,86	vaginaal	0	4
2158	0	30,86	prim.sectio	0	4
2159	0	33,57	vaginaal	0	4
2160	0	36,43	vaginaal	0	4
2161	0	33,43	vaginaal	0	4
2162	0	41,72	sec.sectio	0	4
2163	0	39	vaginaal	0	4
2164	0	37,72	vaginaal	0	4
2165	0	39,28	vaginaal	0	4
2166	0	39,57	vaginaal	0	4
2167	0	39,14	vaginaal	0	4
2168	0	38,72	vaginaal	0	4
2169	0	40,43	vaginaal	0	4
2170	0	40	vaginaal	0	4
2171	0	39,86	sec.sectio	0	4
2172	0	39,72	vaginaal	0	4
2173	0	38	sec.sectio	0	4
2174	0	39,86	vaginaal	0	4
2175	0	39,28	vaginaal	0	4
2176	0	39,57	vaginaal	0	4
2177	0	38,72	vaginaal	0	4

2178	0	39,14	vaginaal	0	4
2179	0	39,43	vaginaal	1	4
2180	0	35	prim.sectio	2	4
2181	0	40,57	Vaginale partus	0	4
2182	0	40,57	Vaginale partus	0	4
2183	0	40,43	Vaginale partus	0	4
2184	0	40,43	Vaginale partus	0	4
2185	0	40,14	Vaginale partus	0	4
2186	0	40,14	Vaginale partus	0	4
2187	0	40	Vaginale partus	2	4
2188	0	40	Vaginale partus	2	4
2189	0	41	Vaginale partus	2	4
2190	0	41	Vaginale partus	2	4
2191	0	39,57	Vaginale partus	2	4
2192	0	39,57	Vaginale partus	2	4
2193	0	38,28	Secundaire sectio	0	5
2194	0	38,28	Secundaire sectio	0	5
2195	0	33,86	Vaginale partus	0	5
2196	0	40,72	Kunstverlossing	0	5
2197	0	37	Vaginale partus	2	5
2198	0	37	Vaginale partus	2	5
2199	0	36	Secundaire sectio	2	5
2200	0	36	Secundaire sectio	2	5
2201	0	38,57	Primaire sectio	2	5
2202	0	35,86	Primaire sectio	2	5
2203	0	35,86	Primaire sectio	2	5
2204	0	35,86	Primaire sectio	2	5
2205	0	32	Primaire sectio	0	5
2206	0	38,28	Secundaire sectio	0	5
2207	0	37,43	Primaire sectio	2	5
2208	0	41,28	Vaginale partus	2	5
2209	0	41,28	Vaginale partus	2	5
2210	0	40	sec.sectio	0	5

2211	0	19,72	Vaginale partus	2	5
2212	0	34,72	Vaginale partus	2	5
2213	0	34,72	Vaginale partus	2	5
2214	0	34,72	Vaginale partus	2	5
2215	0	39,28	Vaginale partus	2	5
2216	0	39,28	Vaginale partus	2	5
2217	0	38,28	Vaginale partus	2	5
2218	0	38,28	Vaginale partus	2	5
2219	0	38,28	Vaginale partus	2	5
2220	0	38,14	Vaginale partus	2	5
2221	0	38,14	Vaginale partus	2	5
2222	0	40	prim.sectio	0	5
2223	0	40,28	Vaginale partus	2	5
2224	0	40,28	Vaginale partus	2	5
2225	0	40,28	Vaginale partus	2	5
2226	0	37,28	Vaginale partus	2	5
2227	0	37,28	Vaginale partus	2	5
2228	0	37,28	Vaginale partus	2	5
2229	0	27,57	Secundaire sectio	2	5
2230	0	27,57	Secundaire sectio	2	5
2231	0	39,86	Vaginale partus	2	5
2232	0	39,86	Vaginale partus	2	5
2233	0	39,72	vaginaal	2	5
2234	0	39,57	Primaire sectio	2	5
2235	0	39,57	Primaire sectio	2	5
2236	0	40,72	Vaginale partus	0	5
2237	0	40,72	Vaginale partus	0	5
2238	0	40,43	Vaginale partus	2	5
2239	0	40,43	Vaginale partus	2	5
2240	0	40,43	Vaginale partus	2	5
2241	0	39	Vaginale partus	2	5
2242	0	39	Vaginale partus	2	5
2243	0	39,86	vaginaal	2	5

2244	0	38,86	Vaginale partus	1	5
2245	0	38,86	Vaginale partus	1	5
2246	0	38,86	Repeat sectio	2	5
2247	0	38,86	Repeat sectio	2	5
2248	0	38,86	Repeat sectio	2	5
2249	0	38,14	vaginaal	2	5
2250	0	40,86	vaginaal	2	5
2251	0	37,28	Vaginale partus	2	5
2252	0	37,72	vaginaal	2	5
2253	0	40,72	vaginaal	2	5
2254	0	36,86	Vaginale partus	0	5
2255	0	36,86	Vaginale partus	0	5
2256	0	36,86	Vaginale partus	0	5
2257	0	38,14	sec.sectio	2	5
2258	0	37,72	vaginaal	0	5
2259	0	36,86	prim.sectio	0	5
2260	0	38	vaginaal	0	5
2261	0	38,14	Secundaire sectio	2	5
2262	0	38,14	Secundaire sectio	2	5
2263	0	39,43	Vaginale partus	2	5
2264	0	39,43	Vaginale partus	2	5
2265	0	36,72	Vaginale partus	0	7
2266	0	37,28		0	7
2267	0	39	Vaginale partus	0	7
2268	0	41,43	vaginaal	0	7
2269	0	37,72	Vaginale partus	0	7
2270	0	37,14	Primaire sectio	0	7
2271	0	36,72	Primaire sectio	1	7
2272	0	34,28	Primaire sectio	0	7
2273	0	37,57	Vaginale partus	0	7
2274	0	35	Repeat sectio	1	7
2275	0	38,28	Vaginale partus	0	7
2276	0	38,28	Vaginale partus	0	7

2277	0	39,57	Vaginale partus	0	7
2278	0	39,57	Vaginale partus	0	7
2279	0	39,72	Vaginale partus	0	7
2280	0	39,72	Vaginale partus	0	7
2281	0	39,14	vaginaal	0	7
2282	0	41	vaginaal	0	7
2283	0	41,14	Secundaire sectio	0	7
2284	0	41,14	Secundaire sectio	0	7
2285	0	40,14	Vaginale partus	0	7
2286	0	40,14	Vaginale partus	0	7
2287	1	38,72	Vaginale partus	0	7
2288	1	38,72	Vaginale partus	0	7
2289	0	36,57	sec.sectio	0	7
2290	0	40	Vaginale partus	0	7
2291	0	40	Vaginale partus	0	7
2292	0	40,72	Vaginale partus	0	7
2293	0	40,72	Vaginale partus	0	7
2294	0	38,28	Vaginale partus	0	7
2295	0	38,28	Vaginale partus	0	7
2296	0	41,14	Vaginale partus	0	7
2297	0	41,14	Vaginale partus	0	7
2298	0	40,72	Secundaire sectio	1	7
2299	0	40,72	Secundaire sectio	1	7
2300	1	40,28	Vaginale partus	0	7
2301	1	40,28	Vaginale partus	0	7
2302	0	39,14	Vaginale partus	0	7
2303	0	39,14	Vaginale partus	0	7
2304	0	40,14	Vaginale partus	0	7
2305	0	40,14	Vaginale partus	0	7
2306	0	38,43	Primaire sectio	1	7
2307	0	38,43	Primaire sectio	1	7
2308	0	41,43	vaginaal	0	7
2309	0	35	Vaginale partus	2	7

2310	0	35	Vaginale partus	2	7
2311	0	39	Vaginale partus	0	7
2312	0	39	Vaginale partus	0	7
2313	0	39,43	Vaginale partus	0	7
2314	0	39,43	Vaginale partus	0	7
2315	0	40,14	Vaginale partus	0	7
2316	0	40,14	Vaginale partus	0	7
2317	0	40,14	Vaginale partus	0	7
2318	0	40	vaginaal	0	7
2319	0	39	Vaginale partus	0	7
2320	0	39	Vaginale partus	0	7
2321	0	35,86	Vaginale partus	0	7
2322	0	41,14	Vaginale partus	0	7
2323	0	41,14	Vaginale partus	0	7
2324	0	35,86	Vaginale partus	0	7
2325	0	39	Vaginale partus	0	7
2326	0	40,14	Secundaire sectio	0	7
2327	0	40,43	Vaginale partus	0	7
2328	0	39	Primaire sectio	0	7
2329	0	38,14	Primaire sectio	0	7
2330	0	37,43	Vaginale partus	0	7
2331	0	40,43	Vaginale partus	1	7
2332	0	38,72	Secundaire sectio	0	7
2333	0	38,72	Secundaire sectio	0	7
2334	0	37,14	Secundaire sectio	0	7
2335	0	37,86	Vaginale partus	0	7
2336	0	37,86	Vaginale partus	0	7
2337	0	38,43	Vaginale partus	0	7
2338	0	38,57	Vaginale partus	0	7
2339	0	38,57	Vaginale partus	0	7
2340	0	37,14	Vaginale partus	0	7
2341	0	37,14	Vaginale partus	0	7
2342	0	41,43	vaginaal	0	7

2343	0	37,43	vaginaal	0	7
2344	0	38,57	Primaire sectio	0	7
2345	0	39,72	Vaginale partus	0	7
2346	0	38,57	Primaire sectio	0	7
2347	0	39,57	Vaginale partus	0	7
2348	0	39,72	Vaginale partus	0	7
2349	0	39,57	Vaginale partus	0	7
2350	0	39,57	Vaginale partus	0	7
2351	0	39,57	Vaginale partus	0	7
2352	0	37,57	Primaire sectio	0	7
2353	0	37,57	Primaire sectio	0	7
2354	0	37,57	Primaire sectio	0	7
2355	0	38,86	vaginaal	0	7
2356	0	37,28	Vaginale partus	0	7
2357	0	37,28	Vaginale partus	0	7
2358	0	37,28	Vaginale partus	0	7
2359	0	39,14	Repeat sectio	0	7
2360	1	38,72	Vaginale partus	1	7
2361	1	38,72	Vaginale partus	1	7
2362	0	40	Vaginale partus	1	7
2363	0	40	Vaginale partus	1	7
2364	0	39,28	Repeat sectio	1	7
2365	0	39,28	Repeat sectio	1	7
2366	0	40	Vaginale partus	0	7
2367	0	40	Vaginale partus	0	7
2368	0	40	Vaginale partus	0	7
2369	1	39,57	Vaginale partus	0	7
2370	0	32,28	prim.sectio	0	7
2371	0	40,57	Vaginale partus	0	7
2372	0	38,72	Secundaire sectio	0	7
2373	0	38,72	Secundaire sectio	0	7
2374	0	40,57	Vaginale partus	0	7
2375	0	38,14	Vaginale partus	1	7

2376	0	38,14	Vaginale partus	1	7
2377	0	36,86	Vaginale partus	0	7
2378	0	35,28	Kunstverlossing	0	7
2379	0	27,72	Primaire sectio	0	7
2380	0	38,43	Vaginale partus	0	7
2381	0	37,14	Vaginale partus	0	7
2382	0	35,72	Vaginale partus	0	7
2383	0	38	Vaginale partus	0	7
2384	0	38	Repeat sectio	0	7
2385	0	33	Primaire sectio	0	7
2386	0	41,14	Repeat sectio	0	7
2387	0	38,28	Primaire sectio	0	7
2388	0	39	Vaginale partus	0	7
2389	0	32,72	Primaire sectio	0	7
2390	0	39,57	Vaginale partus	0	7
2391	0	33	Vaginale partus	0	7
2392	0	36,72	Primaire sectio	0	7
2393	0	35,28	Vaginale partus	0	7
2394	0	39,86	Vaginale partus	0	7
2395	0	41,14	Repeat sectio	0	7
2396	0	37,57	Primaire sectio	0	7
2397	1	40,28	Repeat sectio	2	7
2398	0	24,57	Secundaire sectio	0	7
2399	0	39,28	Vaginale partus	0	7
2400	0	26,43	Primaire sectio	0	7
2401	0	40,28	Vaginale partus	0	7
2402	0	40,28	Vaginale partus	0	7
2403	0	37	Vaginale partus	0	7
2404	0	38,28	Vaginale partus	0	7
2405	0	38,28	Vaginale partus	0	7
2406	0	39,43	vaginaal	0	7
2407	0	41	Vaginale partus	0	7
2408	0	41	Vaginale partus	0	7

2409	0	40,57	Vaginale partus	1	7
2410	0	40,57	Vaginale partus	1	7
2411	0	37,43	Secundaire sectio	0	7
2412	0	37,43	Secundaire sectio	0	7
2413	0	37,43	Secundaire sectio	0	7
2414	0	37,28	Vaginale partus	1	7
2415	0	37,28	Vaginale partus	1	7
2416	0	37,43	vaginaal	0	7
2417	0	40	vaginaal	0	7
2418	0	40	Vaginale partus	0	7
2419	0	40,43	vaginaal	0	7
2420	0	39,86	Vaginale partus	0	7
2421	0	39,86	Vaginale partus	0	7
2422	0	39,43	Vaginale partus	0	7
2423	0	39,43	Vaginale partus	0	7
2424	0	38,86	Vaginale partus	0	7
2425	0	38,86	Vaginale partus	0	7
2426	0	39,43	vaginaal	1	7
2427	1	39,28	Vaginale partus	0	7
2428	1	39,28	Vaginale partus	0	7
2429	0	39,14	Vaginale partus	0	7
2430	0	39,14	Vaginale partus	0	7
2431	1	39,28	Vaginale partus	0	7
2432	0	39,43	Vaginale partus	0	7
2433	0	39,43	Vaginale partus	0	7
2434	0	39,72	vaginaal	0	7
2435	0	41,14	Repeat sectio	0	7
2436	0	41,14	Repeat sectio	0	7
2437	1	37,43	Vaginale partus	0	7
2438	1	37,43	Vaginale partus	0	7
2439	0	38,43	Vaginale partus	0	7
2440	0	38,43	Vaginale partus	0	7
2441	0	41	Vaginale partus	0	7

2442	0	41	Vaginale partus	0	7
2443	0	39,43	Vaginale partus	1	7
2444	0	39,43	Vaginale partus	1	7
2445	0	32,14	vaginaal	2	7
2446	0	40,28	Vaginale partus	0	7
2447	0	40,28	Vaginale partus	0	7
2448	0	41	Secundaire sectio	0	7
2449	0	41	Secundaire sectio	0	7
2450	0	40,86	Primaire sectio	0	7
2451	0	40,86	Primaire sectio	0	7
2452	0	39,28	Vaginale partus	0	7
2453	0	39,28	Vaginale partus	0	7
2454	1	37	vaginaal	0	7
2455	0	29,57	prim.sectio	0	7
2456	0	34,72	vaginaal	0	7
2457	0	37,14	Primaire sectio	0	7
2458	0	18,43		0	7
2459	0	37,86	Secundaire sectio	0	7
2460	0	37,86	Vaginale partus	0	7
2461	0	28,28	Primaire sectio	0	7
2462	0	28,28	Primaire sectio	0	7

ID Code	PPROM <37w	PPROM <34w	Conception	Smoking	Gender	birth weight	BW percentile	SBP-st	DBP-st	MAP-st	HR-st	SV-st	SI-sts	CO-st
1	0	0			0	2965	10	111	79	87	100	66	37	6,6
2	0	0			1	2980	10	147	101	113	105	86	42	9
3	0	0			0	2060	10	127	89	101	113	48	31	5,5
4	0	0			1	2960	10	133	78	91	103	52	34	5,3
5	0	0			0	1780	10	99	71	79	90	84	47	7,5
6	0	0			0	2600	10	117	82	91	113	77	43	8,7
7	0	0			1	1437	10	119	82	91	82	96	48	7,9
8	0	0			0	3020	10	105	73	82	81	66	44	5,3
9	0	0			0	2380	10	131	71	86	92	107	56	9,9
10	0	0			0	3110	10	140	75	86	74	99	58	7,3
11	0	0			1	1160	10	124	94	103	114	52	32	5,9
12	0	0			1	2855	10	110	78	87	105	53	34	5,5
13	0	0			0	2705	10							
14	0	0			0	1845	10	136	90	101	92	77	47	7,1
15	0	0	1		0	2460	10	113	69	80	79	101	58	8
16	0	0	1		0	2460	10	116	64	76	85	97	58	8,2
17	0	0	0	1	0	1775	10	113	76	84	75	79	45	5,9
18	0	0	0	1	0	2805	10	114	63	75	97	101	49	9,8
19	0	0	0	1	0	2805	10	125	70	80	94	98	46	9,2
20	0	0	0	1	1	2395	10	101	70	77	121	56	39	6,7
21	0	0	0	1	1	2395	10	108	72	82	114	44	32	5
22	0	0	0	2	0	3120	10	117	73	85	69	92		6,3
23	0	0	0	0	1	2685	10	116	74	85	93	74	42	6,8
24	0	0	0	0	1	2845	10	111	81	89	96	82	47	7,9
25	0	0	0	0	1	2845	10	119	69	80	84	86	49	7,2
26	0	0	0	0	1	2940	10	94	72	76	98	81	46	7,9
27	0	0	0	0	1	2940	10	97	63	72	106	67	38	7,2
28	0	0	0	0	0	2845	10	108	70	80	100	67	40	6,7
29	0	0	0	0	0	2845	10	97	80	84	98	69	40	6,8
30	0	0	1	0	1	2970	10	134	80	92	89	91	44	8,1
31	0	0	1	0	1	2970	10	121	78	89	84	103	51	8,6
32	0	1	1	0	0	620	10							

33	0	1	1	0	0	620	10	144	94	106	151	55	32	8,4
34	0	0		0	0	1760	10	122	89	98	94	59		5,6
35	0	0		0	1	2460	10	139	99	109	89	78		6,8
36	0	0		0	0	1816	10	134	83	97	80	102		8,2
37	0	0		1	0	2450	10	102	68	77	89	82		7,3
38	0	0			0	2875	10	122	87	96	108	77	41	8,4
39	0	0			0	2875	10	140	87	98	101	85	47	8,6
40	0	0			0	3080	12,5	126	86	95	109	77	39	8,4
41	0	0			1	2785	12,5	125	76	85	122	48	29	5,7
42	0	0			0	2760	12,5	106	75	80	107	54	32	5,7
43	0	0			0	1150	12,5	136	93	104	69	112	57	7,8
44	0	0			0	2290	12,5	104	67	75	89	71	45	6,3
45	0	0			1	2570	15	106	67	77	103	79	45	8,1
46	0	0			0	3250	15	115	74	83	77	100	53	7,7
47	0	0			0	3095	15	143	82	95	118	78	43	9,2
48	0	0			1	2940	15	108	67	75	80	115	63	9,3
49	0	0			1	2570	15	113	70	82	92	77	45	7,1
50	0	0	0		1	2925	15	85	68	75	106	48		5,1
51	0	0			0	2940	15	125	87	96	67	77	46	5,1
52	0	0			1	2620	17,5	119	66	76	104	63	34	6,5
53	0	0			0	1250	17,5	124	85	96	82	85	45	7
54	0	0			1	1420	17,5	126	82	92	93	50	36	4,7
55	0	0			0	2610	17,5	116	84	93	85	67	41	5,7
56	0	0			1	2770	20	125	81	92	69	93	45	7,1
57	0	0			1	2770	20	113	71	82	119	98	49	11,7
58	0	0			1	3000	20	108	74	82	111	46	29	5,1
59	0	0			1	3015	20	118	81	90	89	85	46	7,6
60	0	0			1	1860	20	139	92	103	104	61	32	6,3
61	0	0			1	3015	20	125	80	90	82	98	54	8
62	0	0			1	2770	20	123	82	90	122	88	43	10,8
63	0	0			0	3200	20	111	75	85	95	90		8,5
64	0	0			1	3030	20	116	76	87	91	72	41	6,6
65	0	0			0	2565	22,5	146	99	110	123	100	48	12,4

66	0	0			1	2670	22,5	123	76	86	96	66	39	6,4
67	0	0			1	3170	22,5	122	83	94	106	67	41	7,1
68	0	0			0	3010	22,5	110	73	81	86	84	48	7,2
69	0	0			0	3010	22,5	117	81	89	82	91	50	7,4
70	0	0			1	2895	25	118	81	90	110	85	43	9,3
71	0	0			0	2930	25	108	71	80	86	76	42	6,6
72	0	0			1	2610	25	110	65	73	90	60	36	5,4
73	0	0			0	3075	27,5	118	71	81	96	81	48	7,8
74	0	0			1	3235	27,5	147	81	98	99	92	42	9,1
75	0	0			0	2415	30	112	71	80	97	46	29	4,5
76	0	0			1	1367	30	184	125	140	88	84	40	7,4
77	0	0			1	3290	35	120	69	79	99	73	42	7,2
78	0	0			0	3595	37,5	108	61	72	103	125	61	12,8
79	0	0			0	2500	40	105	70	79	91	88	50	8,1
80	0	0			1	3360	40	122	76	86	97	75	42	7,4
81	0	0			1	1820	40	106	66	77	73	85	54	6,3
82	0	0			1	3360	40	105	74	82	103	72	40	7,5
83	0	0			0	3545	42,5	137	94	104	99	81	39	8
84	0	0	0		0	2940	45	121	73	84	108	70	34	7,6
85	0	0	0		0	2940	45	110	74	84	96	85	41	8,1
86	0	0			1	3450	45,5	142	97	109	113	75	40	8,5
87	0	0			1	1475	47,5	111	76	85	113	75	43	8,5
88	0	0			0	2590	50	108	76	86	99	82	46	8,2
89	0	0			0	3305	50	114	68	76	85	97	53	8,3
90	0	0			0	3305	50	115	66	78	86	90	51	7,8
91	0	0			1	3350	52,5	128	81	93	99	89	52	8,8
92	0	0			1	3350	52,5	123	90	100	109	67	40	7,3
93	0	0			0	3670	55	126	86	96	91	82	44	7,5
94	0	0			0	3230	55	118	76	86	105	80	41	8,4
95	0	0			0	3255	57,5	130	78	92	91	80	47	7,3
96	0	0			0	3695	57,5	112	70	80	120	51	29	6,2
97	0	0			1	2945	57,5							
98	0	0			1	3430	60	119	65	80	93	80	44	7,4

99	0	0			1	3490	60	99	65	75	75	102	59	7,6
100	0	0			1	3545	60	115	71	80	87	102	50	8,9
101	0	0			1	3460	62,5	134	83	90	125	68	38	8,6
102	0	0			0	3620	62,5	99	75	84	90	120	63	10,8
103	0	0			0	3465	62,5	141	95	108	103	78	41	8,1
104	0	0			0	3465	62,5	138	94	104	108	56	34	6,1
105	0	0			0	3620	62,5	110	73	82	84	53	36	4,5
106	0	0			0	3885	62,5	82	61	66	90	51	35	4,6
107	0	0			0	3620	62,5	103	67	75	95	51	34	4,9
108	0	0			0	3490	65	143	98	109	104	58	34	6,1
109	0	0			0	3445	65	124	83	94	103	98	49	10,1
110	0	0			0	3445	65	124	82	93	98	92	44	9
111	0	0			1	3650	67,5	101	68	78	101	81	49	8,2
112	0	0			1	3650	67,5	110	70	79	97	67	42	6,5
113	0	0			0	3520	67,5	120	74	84	100	89	49	8,9
114	0	0			1	3630	67,5	115	73	84	88	83	47	7,3
115	0	0			0	3710	70	121	74	86	88	81	47	7,1
116	0	0			0	3705	70	131	77	89	105	64	35	6,8
117	0	0			1	3275	70	126	72	85	83	76	43	6,4
118	0	0			1	3200	72,5	123	88	98	122	76	39	9,3
119	0	0			1	3420	72,5	126	74	85	120	62	31	7,5
120	0	0			1	3725	75	116	75	85	84	128	65	10,7
121	0	0			0	3586	75	138	91	102	135	60	33	8,2
122	0	0			0	3260	75	124	75	86	117	75	35	8,8
123	0	0			1	3615	75	121	82	91	93	93	48	8,7
124	0	0			1	3725	75	128	75	86	85	113	59	9,6
125	0	0	0		1	3830	75	115	68	79	96	104		10
126	0	0			0	3260	75	123	75	87	113	82	37	9,2
127	0	0			1	3615	75	132	84	93	105	79	43	8,4
128	0	0			1	3615	75	130	76	87	88	93	49	8,2
129	0	0			1	3640	77,5	122	88	98	72	108	51	7,8
130	0	0			1	2610	77,5	119	72	83	87	77	40	6,7
131	0	0			1	3670	77,5	111	84	92	101	70		7,1

132	0	0			1	2610	77,5	121	75	87	87	93	49	8,1
133	0	0			0	3665	80	134	88	97	92	86	45	7,9
134	0	0			0	3150	80	152	117	126	87	83	44	7,2
135	0	0			1	3710	80	128	69	83	105	74	45	7,7
136	0	0			1	3510	80	118	84	93	99	77	38	7,6
137	0	0			1	3215	80	108	74	81	92	101	48	9,3
138	0	0			0	3995	80	99	68	77	90	104	57	9,4
139	0	0			1	3545	82,5	151	94	110	107	97	48	10,4
140	0	0			0	3750	85	122	86	95	104	72	38	7,6
141	0	0			0	3910	85	162	102	116	94	77	42	7,3
142	0	0			0	4085	85	109	67	77	93	85	47	7,9
143	0	0			1	4040	87,5	131	81	92	110	116	57	12,8
144	0	0			1	3825	87,5	105	72	81	98	77	43	7,6
145	0	0			1	3825	87,5	122	68	84	97	96	53	9,3
146	0	0	0		1	3490	87,5	110	72	81	91	76		6,9
147	0	0			0	3850	90	122	81	90	109	91	48	9,9
148	0	0			1	3690	90	160	104	119	108	109	52	11,8
149	0	0			1	3975	92,5	124	88	98	88	129	56	11,3
150	0	0			1	3620	92,5	135	84	98	92	80	45	7,4
151	0	0			1	4060	92,5	167	118	127	78	131	62	10,3
152	0	0			1	4135	97,5				102	82	41	8,4
153	0	0			0	4375	97,5	111	70	81	112	70	41	7,8
154	0	0			1	4135	97,5	128	81	94	100	84	42	8,3
155	0	0			1	4305	97,5	119	73	83	113	55	34	6,2
156	0	0			0	4775	97,5	120	66	77	97	107	54	10,4
157	0	0	0		0	3485	37,5	126	86	96	102	70		7,1
158	0	0	1		0	3045	20	109	75	83	84	85	54	7,1
159	0	0	1		0	3045	20	99	73	82	92	70	45	6,4
160	0	0	0		0	3890	62,5	115	70	82	91	88	52	7,9
161	0	0	0		0	3890	62,5	118	69	81	97	84	49	8,1
162	0	0	1		0	3385	70	111	69	77	115	63	32	7,2
163	0	0	1		0	3385	70	118	70	79	95	79	42	7,5
164	0	0	0		0	3025	20	105	67	77	96	114	56	10,9

165	0	0	0		0	3025	20	131	74	89	111	83	42	9,2
166	0	0			0	3285	47,5	118	82	91	93	71	36	6,6
167	0	0			0	3285	47,5	98	68	77	69	96	50	6,7
168	0	0	0		0	2835	25	100	73	81	84	74		6,3
169	0	0			1	1765	15	124	81	90	126	68	35	8,6
170	0	0			0	3910	75	119	73	85	96	78	50	7,5
171	0	0			0	3910	75	114	73	84	104	52	34	5,4
172	0	0			1	3525	55	133	83	95	99	83	43	8,2
173	0	0			0	4270	97,5	98	70	78	96	92	48	8,9
174	0	0			0	3090	15	128	77	87	100	82	49	8,2
175	0	0			1	3685	72,5	103	66	76	75	97		7,3
176	0	0			1	3285	90							
177	0	0			1	2820	80	99	69	78	103	65	41	6,8
178	0	0			1	3280	45	101	74	82	114	71	40	8
179	0	0			1	3280	45	130	79	92	93	89	48	8,2
180	0	0			1	3280	45	128	81	91	91	88	49	8
181	0	0			0	4255	92,5	133	83	91	115	67	36	7,8
182	0	0			0	3305	32,5	123	82	94	96	73	43	6,9
183	0	0			0	2355	20	107	68	77	105	65	40	6,9
184	0	0			1	3375	42,5	124	78	88	136	49	29	6,6
185	0	0			0	3170	20	138	89	98	110	68	40	7,5
186	0	0			1	4280	97,5	108	63	72	90	96	48	8,8
187	0	0			1	3010	32,5	141	81	96	91	93	45	8,5
188	0	0			1	3875	90	158	95	102	112	75	37	8,4
189	0	0			0	3430	32,5	118	75	85	101	103	55	10,4
190	0	0			0	2320	20	131	83	93	102	82	41	8,5
191	0	0			0	2320	20	151	98	110	89	74	36	6,6
192	0	0			1	3250	30	130	94	103	79	72	38	5,7
193	0	0	1					118	75	86	93	61		5,7
194	0	0	0		1	3205	25	109	75	85	101	69		7
195	0	0			1	3525	57,5	131	84	93	109	73	39	8
196	0	0	0		1	3050	22,5	109	74	84	92	59	39	5,4
197	0	0	0		1	3050	22,5	109	71	80	101	71	46	7,1

198	0	0			1	3075	22,5							
199	0	0			0	2780	20	120	74	85	105	107	56	11,3
200	0	0			0	1170	55	122	69	81	103	83	46	8,7
201	0	0	0		0	3675	67,5	121	85	94	79	104	55	8,1
202	0	0	0		0	3675	67,5	117	68	80	73	93	51	6,8
203	0	0	0		0	3675	67,5	117	81	88	66	102	57	6,7
204	0	0			0	3605	100	153	88	104	99	74	35	7,4
205	0	0			0	3605	100	156	84	103	101	91	42	9,2
206	0	0			1	3495	55							
207	0	0			1	3495	55	103	68	79	102	99	48	10,1
208	0	0			0	3975	77,5	109	75	85	91	99	48	8,9
209	0	0			0	3940	92,5	118	81	91	102	94	48	9,4
210	0	0			1	3980	92,5	132	90	102	91	75	40	6,8
211	0	0	2		0	3325	22,5	124	79	90	81	130	58	10,6
212	0	0			1	3295	32,5	107	73	83	84	92	47	7,8
213	0	0			0	3440	62,5	105	70	79	104	54	31	5,6
214	0	0			0	3440	62,5	122	82	93	96	76	44	7,3
215	0	0			0	3420	72,5	115	73	83	80	125	61	10
216	0	0			0	3420	72,5	122	76	88	81	153	74	12,3
217	0	0			0	3820	67,5	109	78	86	87	80	42	6,9
218	0	0			1	3815	87,5	126	85	95	102	87	47	8,8
219	0	0			1	3815	87,5	110	75	84	88	87	52	7,6
220	0	0			1	3815	87,5	117	76	86	93	75	44	7
221	0	0			1	2895	25	107	69	78	82	82	48	6,8
222	0	0			1	3635	97,5	138	99	110	97	81	37	7,8
223	0	0			0	3100	50	124	83	92	103	97	55	9,9
224	0	0			0	2450	30	125	84	95	89	85	44	7,6
225	0	0			0	3485	65	148	99	111	79	106	56	8,4
226	0	0			0	2520	20	107	74	83	104	62	35	6,5
227	0	0			1	2620	67,5	124	88	98	86	90	46	7,7
228	0	0			0	3785	87,5	152	100	112	86	70	33	6
229	0	0			1	2970	30	157	98	111	118	81	37	9,6
230	0	0			1	2970	30	141	86	98	104	96	46	10

231	0	0	0		0	2700	37,5	143	83	95	105	145	60	15,2
232	0	0	0		0	2700	37,5	132	69	82	83	152	69	12,6
233	0	0	0		0	2700	37,5	123	84	93	128	92	41	11,9
234	0	0	0		1	2780	32,5	125	90	101	103	61		6,2
235	0	0			1	1658	42,5	148	103	115	104	82	38	8,5
236	0	0			1	4270	97,5	135	78	93	94	134	63	12,6
237	0	0	0		0	3718	82,5	141	88	100	101	107	43	10,8
238	0	0	0		0	3718	82,5	138	89	97	99	95	38	9,4
239	0	0	0		0	3885	95	81	67	71	92	85		7,8
240	0	0			0	3440	45	116	70	79	83	107	55	9
241	0	0			0	3275	87,5	115	52	69	108	68	41	7,4
242	0	0			0	3775	87,5	144	90	102	95	88	40	8,4
243	0	0			0	3775	87,5	135	82	97	101	72	33	7,2
244	0	0			0	4255	87,5	135	77	90	88	109	51	9,6
245	0	0			0	4550	100	140	91	103	122	62	31	7,6
246	0	0			0	4550	100	155	97	110	108	72	34	7,8
247	0	0			0	3545	92,5	160	97	112	121	92	46	11,1
248	0	0			0	3545	92,5	146	82	97	119	85	44	10,2
249	0	0			1	3135	47,5	127	86	96	126	94	48	11,9
250	0	0			0	4285	100	179	114	131	119	53	26	6,3
251	0	0	0		1	3940	97,5	123	64	75	98	77	46	7,5
252	0	0	0		1	3940	97,5	123	68	79	107	61	36	6,5
253	0	0	0		1	3910	95	105	71	83	96	84	47	8,1
254	0	0	0		1	3910	95	112	79	87	97	74	43	7,2
255	0	0	0		1	3140	30	106	68	78	104	78	46	8,1
256	0	0	0		1	3140	30	111	67	76	98	68	41	6,7
257	0	0	0		1	3315	47,5	110	71	81	86	85	49	7,3
258	0	0	0		1	2765	37,5	121	65	77	83	97	49	8,1
259	0	0	0		1	2765	37,5	99	62	70	81	120	59	9,8
260	1	0			0	2800	42,5	130	76	86	83	76	46	6,4
261	1	0			0	2800	42,5	118	79	88	76	81	49	6,2
262	1	0			0	3175	82,5	103	67	76	85	93	55	7,9
263	0	1	0		1	2035	77,5	118	81	91	85	95	50	8,1

264	0	1	0		1	2035	77,5	121	83	94	79	86	45	6,7
265	0	0	0	3	1	3630	65	119	80	90	110	72	36	7,9
266	0	0	0	3	0	3025	20	105	74	83	108	60	36	6,5
267	0	0	0	3	0	3025	20	119	79	89	98	61	36	5,9
268	0	0	0	3	1	3770	77,5	117	71	82	94	80	44	7,5
269	0	0	0	3	1	3770	77,5	107	77	86	96	82	45	7,9
270	0	0	0	3	0	2785	52,5	102	65	74	92	65	39	6
271	0	0	0	3	0	2785	52,5	132	72	85	89	66	41	5,9
272	0	0	0	3	0	3430	57,5	119	76	86	91	77	41	7,1
273	0	0	0	3	0	3430	57,5	124	79	88	80	88	49	7,1
274	0	0	0	1	1	2490	15	114	70	81	83	88	56	7,3
275	0	0	0	1	1	2490	15	124	81	93	90	71	46	6,4
276	0	0	0	1	1	3165	20	129	74	87	90	67	38	6,1
277	0	0	0	1	1	3165	20	133	74	88	99	72	41	7,2
278	0	0	0	1	0	3270	27,5	128	73	83	95	106	55	10,1
279	0	0	0	1	0	3270	27,5	124	79	93	106	83	45	8,8
280	0	0	0	1	1	3105	27,5	105	65	76	85	104	56	8,9
281	0	0	0	1	1	3105	27,5	107	62	72	83	133	69	11,4
282	0	0	0	1	1	3460	37,5	126	83	93	101	62	29	6,3
283	0	0	0	1	1	3460	37,5	133	84	99	90	80	39	7,2
284	0	0	0	1	0	3320	50	113	69	80	100	82	43	8,2
285	0	0	0	1	0	3320	50	116	73	83	98	90	49	8,9
286	0	0	0	1	0	3875	72,5	114	63	73	97	75	40	7,2
287	0	0	0	1	0	3875	72,5	110	68	79	92	87	46	8
288	0	0	0	1	1	3730	82,5	76	45	54	95	68	39	6,5
289	0	0	0	1	1	3730	82,5	103	61	70	90	71	41	6,3
290	0	0	0	1	0	3495	37,5	123	69	82	72	107	55	7,7
291	0	0	0	1	0	3495	37,5	125	72	83	62	115	61	7,1
292	0	0	0	1	0	3565	57,5	132	86	96	100	61	37	6,1
293	0	0	0	1	0	3565	57,5	125	76	87	93	87	50	8
294	0	0	0	1	1	3405	32,5	114	76	87	107	85	46	9,1
295	0	0	0	1	1	3405	32,5	111	79	88	99	85	47	8,4
296	0	0	0	1	1	3020	20	101	72	81	98	76	43	7,4

297	0	0	0	1	1	3020	20	111	74	84	102	90	50	9,2
298	0	0	2	1	0	2810	15	114	74	82	97	57	37	5,5
299	0	0	2	1	0	2810	15	122	74	84	105	56	37	5,8
300	0	0	0	1	1	3980	92,5	153	84	95	112	122	59	13,6
301	0	0	0	1	1	3980	92,5	127	90	99	101	98	48	9,9
302	0	0	0	1	0	2835	47,5	116	69	78	103	82	49	8,5
303	0	0	0	1	0	2835	47,5	118	68	83	107	72	43	7,7
304	0	0	0	1	0	3470	62,5	108	64	74	88	83	48	7,3
305	0	0	0	1	0	3470	62,5	101	63	73	93	81	49	7,6
306	0	0	0	1	0	3800	65	90	72	77	91	81	44	7,3
307	0	0	0	1	0	3800	65	113	78	86	108	85	46	9,1
308	0	0	0	1	0	4180	85	103	69	78	98	86	49	8,4
309	0	0	0	1	0	2950	15	121	82	92	108	63	35	6,7
310	0	0	0	1	0	2950	15	121	87	96	106	63	37	6,7
311	0	0	0	1	0	3470	65	100	69	76	103	64	38	6,5
312	0	0	0	1	0	3470	65	100	64	72	96	79	46	7,6
313	0	0	0	1	0	3310	22,5	108	75	85	94	78	45	7,4
314	0	0	0	1	0	3310	22,5	100	68	77	92	93	53	8,6
315	0	0	0	1	1	3040	77,5	106	66	76	63	100	57	6,2
316	0	0	0	1	1	3040	77,5	105	74	82	79	89	50	7,1
317	0	0		1	1	2675	30	109	78	86	93	74	40	6,9
318	0	0		1	1	2675	30	109	73	82	92	75	41	6,9
319	0	0	0	1	1	3660	77,5	141	86	97	112	82	37	9,2
320	0	0	0	1	1	3660	77,5	107	73	82	100	83	38	8,2
321	0	0	1	1	0	3425	80	109	69	79	90	70	42	6,3
322	0	0	0	1	1	3455	47,5	106	67	74	89	78	50	7
323	0	0	0	1	1	3455	47,5	111	66	78	79	79	52	6,3
324	0	0	0	1	0	3070	60	111	71	82	114	82	41	9,3
325	0	0	0	1	0	3070	60	111	73	81	97	75	39	7,3
326	0	0	0	1	0	3330	25	111	68	79	84	103	58	8,6
327	0	0	2	1	0	3155	30	117	72	82	100	77	45	7,7
328	0	0	2	1	0	3155	30	119	77	87	72	93	54	6,7
329	0	0	0	1	1	3055	35	114	70	80	116	50	29	5,7

330	0	0	0	1	1	2825	37,5	100	63	72	91	89	55	8
331	0	0	0	1	1	2825	37,5	108	70	80	88	67	43	5,9
332	0	0	0	1	1	3655	67,5	116	79	89	97	92	52	9
333	0	0	0	1	1	3655	67,5	120	83	92	79	72	44	5,7
334	0	0	0	1	0	3480	47,5	100	66	75	113	108	56	12,2
335	0	0	0	1	0	3480	47,5	105	62	75	87	109	56	9,5
336	0	0	0	1	1	3150	20	106	73	82	86	84	47	7,2
337	0	0	0	1	1	3150	20	122	85	94	86	86	49	7,4
338	0	0	0	1	0	4530	97,5	143	82	92	98	95	42	9,4
339	0	0	0	1	1	3415	72,5	106	64	75	82	76	43	6,3
340	0	0	0	1	1	3415	72,5	93	62	70	80	89	49	7,1
341	0	0	0	1	0	3840	70	110	67	78	107	85	48	9,1
342	0	0	0	1	0	3840	70	122	71	83	117	68	39	8
343	0	0	0	1	0	3640	52,5	112	66	77	88	70	41	6,2
344	0	0	0	1	0	3640	52,5	107	62	70	84	84	49	7,1
345	0	0	0	1	1	3035	37,5	112	76	84	123	53	31	6,6
346	0	0	0	1	1	3035	37,5	113	71	81	94	65	39	6,1
347	0	0	0	1	0	3815	67,5	105	71	79	92	72	39	6,7
348	0	0	0	1	0	3815	67,5	119	74	84	92	72	39	6,6
349	0	0	0	1	0	3520	32,5	115	71	83	114	54	32	6,1
350	0	0	0	1	0	3520	32,5	111	69	79	100	74	44	7,4
351	0	0	0	1	0	3660	65	115	78	87	107	61	36	6,6
352	0	0	0	1	0	3660	65	97	67	76	94	84	48	7,8
353	0	0	0	1	0	3490	37,5	109	83	92	99	58	36	5,8
354	0	0	0	1	0	3490	37,5	116	86	95	104	65	40	6,8
355	1	0	0	1	1	1765	15	110	76	84	100	57	38	5,7
356	1	0	0	1	1	1765	15	113	75	84	104	42	28	4,4
357	0	0	0	1	0	3395	40	110	73	83	88	116		10,2
358	0	0	0	1	0	3205	12,5	116	70	80	90	76		6,8
359	0	0	0	1	0	2670	22,5	124	76	88	101	76		7,7
360	0	0	0	1	0	2695	15	121	66	77	91	64		5,8
361	0	0	0	1	0	3555	42,5	98	65	75	119	56		6,6
362	0	0	0	1	0	3650	87,5	123	77	88	100	83		8,3

363	0	0	0	1	1	2560	17,5	114	80	89	93	109		10,2
364	0	0	0	1	0	2920	12,5	135	87	99	81	76		6,1
365	0	0	0	1	0	3060	12,5	94	66	76	92	69		6,3
366	0	0	0	1	1	3370	40	111	63	74	94	80		7,5
367	0	0	0	1	0	3815	67,5	126	78	89	79	110		8,7
368	0	0	0	1	0	2820	32,5	119	71	83	92	82		7,6
369	0	0	0	1	0	3165	32,5	102	68	79	92	79		7,3
370	0	0	0	1	1	3045	22,5	103	59	70	98	71		6,9
371	0	0	0	1	1	4215	95	125	77	88	114	77		8,9
372	0	0	0	1	0	3310	45	114	71	85	90	61		5,5
373	0	0	0	1	0	2995	17,5	102	74	80	95	58		5,5
374	0	0	0	1	1	3430	72,5	102	75	83	82	86		7
375	0	0	0	1	0	3755	85	134	84	98	98	64		6,2
376	0	0	0	1	0	3325	32,5	103	66	75	94	72		6,7
377	0	0	0	1	0	3060	80	117	85	93	88	85		7,5
378	0	0	0	1	0	3965	77,5	84	69	75	112	65		7,3
379	0	0	0	1	0	3490	77,5	117	79	89	98	79		7,7
380	0	0	0	1	1	2995	42,5	115	83	92	115	69		7,9
381	0	0	0	1	0	4005	90	117	81	90	101	94		9,5
382	0	0	0	1	1	3680	90	121	73	83	84	134		11,2
383	0	0	0	1	1	3560	60	138	97	106	77	77		5,9
384	0	0	0	1	1	2655	12,5	123	80	90	96	73		6,9
385	0	0	0	2	0	3430	57,5	118	80	90	104	66	41	6,9
386	0	0	0	2	0	3430	57,5	123	82	93	93	60	38	5,6
387	0	0	0	2	0	3980	72,5	120	66	81	90	94	53	8,5
388	0	0	0	2	0	3980	72,5	106	68	76	77	91	52	7,1
389	0	0	0	2	1	2980	12,5	128	72	86	89	76	43	6,7
390	0	0	0	2	1	2980	12,5	114	67	78	81	86	48	6,9
391	0	0	0	2	1	3370	42,5	102	66	76	132	72	39	9,6
392	0	0	0	2	1	3370	42,5	105	61	71	98	87	49	8,5
393	0	0	1	2	0	3525	55	106	73	82	123	51	32	6,2
394	0	0	1	2	0	3525	55	101	75	83	116	51	33	6
395	0	0	0	2	1	3015	57,5	119	84	95	130	55	30	7,2

396	0	0	0	2	1	3015	57,5	124	87	97	117	67	37	7,9
397	0	0	0	2	0	3975	80	93	67	73	89	77	43	6,9
398	0	0	0	2	0	3975	80	118	86	95	105	73	41	7,6
399	0	0	0	2	1	3345	52,5	115	80	89	93	76	44	7,2
400	0	0	0	2	1	3345	52,5	107	69	79	96	80	45	7,7
401	0	0	0	2	1	3435	47,5	128	75	89	81	104	51	8,4
402	0	0	0	2	0	3765	75	125	83	93	108	93	46	10,1
403	0	0	0	2	0	3765	75	136	78	92	95	99	51	9,4
404	0	0	0	2	1	2965	50	105	70	80	102	88	51	8,9
405	0	0	0	2	0	3066	12,5	130	76	87	100	64		6,4
406	0	0	0	2	0	3400	20	108	74	83	89	64		5,7
407	0	0	0	2	0	3680	80	124	78	90	105	70		7,3
408	0	0	0	2	0	3405	60	117	72	83	74	122		9
409	0	0	0	2	0	3510	52,5	133	82	94	113	72		8,1
410	0	0	0	2	0	2940	27,5	91	58	67	96	72		6,9
411	0	0	0	2	1	3365	52,5	96	70	79	93	75		7
412	0	0	0	2	1	3575	62,5	114	77	83	88	93		8,1
413	0	0	0	2	1	3055	15	108	78	86	83	75		6,2
414	0	0	0	2	0	3556	42,5	125	72	85	90	66		5,9
415	1	0	0	2	0	2680	55	105	65	75	95	64		6,1
416	0	1	0	2	0	1400	35	109	83	90	94	60		5,6
417	0	0		2	0	3430	30	109	77	86	98	78	42	7,6
418	0	0	0	1	1	3200	35	102	65	75	82	87	56	7,1
419	0	0	0	1	1	3200	35	97	70	78	80	73	49	5,8
420	0	0	0	4	0	3060	22,5	109	69	78	90	65		5,8
421	0	0	0	4	0	3570	45	113	69	79	92	84	50	7,7
422	0	0	0	4	0	3570	45	109	75	83	95	74	45	7
423	0	0	0	4	0	3345	65	110	84	91	79	111		8,7
424	0	0	0	4	1	2775	20	101	63	73	81	82	50	6,7
425	0	0	0	4	1	2775	20							
426	0	0	1	4	0	2550	15	109	70	80	87	82		7,1
427	0	0	0	4	0	3065	57,5	111	74	85	81	70		5,7
428	0	0	0	4	0	3200	22,5	100	59	70	77	70		5,4

429	0	0	0	4	0	3280	47,5	113	73	83	95	69		6,6
430	0	0	0	4	1	3250	27,5	100	65	74	87	111	60	9,7
431	0	0	0	4	1	3250	27,5	93	64	72	99	105	56	10,3
432	0	0	0	4	1	3535	82,5	115	80	90	95	74		7
433	0	0	0	4	0	2945	15	121	74	85	90	91	51	8,2
434	0	0	0	4	0	2945	15	122	81	90	91	88	50	8
435	0	0	0	4	1	3690	72,5	132	80	86	94	104		9,7
436	0	0	0	4	1	2995	42,5	102	74	81	111	46		5,1
437	0	0	0	4	1	4225	95	94	66	75	92	73		6,7
438	0	0	0	4	1	4370	97,5	101	67	78	107	100	50	10,6
439	0	0	0	4	1	4370	97,5	132	77	92	101	93	49	9,5
440	0	0	0	4	0	3365	17,5	109	76	86	85	93	43	7,9
441	0	0	0	4	0	3365	17,5	100	62	72	77	162	75	12,4
442	0	0	0	4	0	4290	92,5	124	84	95	89	91	50	8,2
443	0	0	0	4	0	4290	92,5	118	77	87	86	97	51	8,3
444	0	0	0	4	0	3260	15	103	74	83	88	66	39	5,8
445	0	0	0	4	0	3260	15	100	77	83	91	59	36	5,4
446	0	0	1	4	0	4120	97,5	146	82	95	84	128	56	10,7
447	0	0	1	4	0	4120	97,5	140	75	91	98	101	44	9,9
448	0	0	0	4	0	3575	57,5	118	79	90	88	90		7,9
449	0	0	0	4	0	3720	57,5	116	76	87	90	92	51	8,4
450	0	0	0	4	0	3670	67,5	118	75	86	112	78	45	8,7
451	0	0	0	4	0	3670	67,5	123	75	84	96	66	40	6,4
452	0	0	0	4	1	3340	50	117	68	80	82	48		4
453	0	0	0	4	0	3700	57,5	109	67	78	104	95	55	9,8
454	0	0	0	4	0	3700	57,5	113	73	83	91	78	46	7,1
455	0	0	0	4	1	3460	62,5	124	82	93	122	72	40	8,8
456	0	0	0	4	1	3460	62,5	135	86	96	120	65	36	7,9
457	0	0	0	4	1	3605	72,5	116	78	87	111	95	47	10,5
458	0	0	0	4	1	3680	70	119	81	91	116	74		8,6
459	0	0	0	4	1	3560	70	111	74	83	97	81	46	7,8
460	0	0	0	4	1	3560	70	124	79	89	88	72	43	6,4
461	0	0	0	4	1	3075	40	103	67	77	80	86	51	6,9

462	0	0	0	4	1	3075	40	97	63	72	80	84	50	6,7
463	0	0	0	4	0	2840	17,5	122	69	81	107	58	38	6,2
464	0	0	0	4	0	2840	17,5	103	62	71	85	62	41	5,3
465	0	0	1	4	0	4050	82,5	108	68	78	95	115	57	10,9
466	0	0	1	4	0	4050	82,5	109	66	77	87	122	59	10,6
467	0	0		4	0	4770	97,5							
468	0	0	1	4	0	3280	27,5	103	67	76	84	101	55	8,5
469	0	0	1	4	0	3280	27,5	109	73	82	88	75	42	6,7
470	0	0		4	0	3235	25	110	78	87	85	88	49	7,5
471	0	0		4	0	3235	25	117	77	88	92	97	52	8,9
472	0	0		4	0	3120	65	123	77	87	94	82	49	7,8
473	0	0		4	0	3120	65	108	77	86	96	64	40	6,2
474	0	0	0	0	0	3050	12,5	93	68	75	102	45	29	4,6
475	0	0	0	0	0	3050	12,5	94	64	73	86	63	38	5,4
476	0	0	0	0	1	2790	12,5	111	68	78	83	72	48	5,9
477	0	0	1	0	0	2740	12,5	121	87	96	97	70	40	6,8
478	0	0	1	0	0	2740	12,5	123	86	93	95	88	49	8,3
479	0	0	0	0	1	2790	12,5	101	66	76	87	67	45	5,9
480	0	0	0	0	0	3160	12,5	110	70	80	77	87		6,6
481	0	0	0	0	1	2945	15	107	72	82	100	72	47	7,2
482	0	0	0	0	1	3185	15	103	70	79	97	69	42	6,7
483	0	0	0	0	1	2945	15	109	71	81	96	65	43	6,3
484	0	0	0	0	1	2825	15	103	72	81	96	68		6,5
485	0	0	0	0	1	2970	15	116	71	82	84	72	46	6,1
486	0	0	0	0	0	3065	15	92	60	68	108	61		6,6
487	0	0	0	0	1	2970	15	117	68	76	77	92	57	7,1
488	0	0	0	0	0	3175	17,5	134	90	103	119	48	32	5,8
489	0	0	0	0	0	3175	17,5	117	83	93	105	59	39	6,2
490	0	0	0	0	0	3005	17,5	135	79	92	96	84	47	8
491	0	0	0	0	0	3005	17,5	131	76	89	95	99	54	9,5
492	0	0	0	0	0	3125	17,5	121	65	78	94	93	51	8,7
493	0	0	0	0	0	3125	17,5	118	65	76	86	90	50	7,8
494	0	0	0	0	1	3285	22,5	122	88	98	101	66	42	6,8

495	0	0	0	0	1	3055	22,5	99	66	74	91	88	50	8
496	0	0	0	0	0	3220	22,5	110	71	81	100	63	38	6,3
497	0	0	0	0	1	3075	22,5							
498	0	0	0	0	1	3285	22,5	122	77	88	111	52	34	5,8
499	0	0	0	0	0	3220	22,5	100	68	77	100	76	45	7,6
500	0	0	0	0	1	3055	22,5	97	66	75	93	78	46	7,3
501	0	0	0	0	1	3075	22,5	113	69	79	99	63	38	6,3
502	0	0	0	0	1	2835	25	123	82	93	107	66	42	7
503	0	0	0	0	0	3260	25	113	79	89	92	84	49	7,7
504	0	0	0	0	0	3260	25	107	76	85	97	75	43	7,3
505	0	0	0	0	1	2835	25	132	87	99	112	78	49	8,7
506	0	0	2	0	1	3110	25	103	64	73	76	72	44	5,4
507	0	0	2	0	1	3110	25	105	56	68	90	59	35	5,2
508	0	0	0	0	0	3110	27,5	119	81	92	101	67	39	6,7
509	0	0	0	0	0	3110	27,5	97	68	76	89	68	40	6,1
510	0	0	1	0	0	2935	27,5	107	79	87	99	65		6,4
511	0	0	0	0	1	2985	27,5	123	67	80	59	130	69	7,7
512	0	0	0	0	1	2985	27,5	125	73	87	75	122	64	9,1
513	0	0	0	0	1	3250	30	119	67	79	108	60	33	6,5
514	0	0	0	0	1	3250	30	116	77	86	107	63	35	6,7
515	0	0	0	0	0	3290	30	127	72	90	85	110	60	9,3
516	0	0	0	0	0	3290	30	113	76	86	91	102	54	9,2
517	0	0		0	1	3170	32,5	105	72	80	99	54		5,4
518	0	0	0	0	0	3015	32,5	109	75	85	104	74	41	7,7
519	0	0	0	0	0	2920	32,5	114	68	79	74	95	52	7
520	0	0	0	0	0	2920	32,5	117	77	85	75	100	55	7,5
521	0	0	0	0	1	3300	35	114	76	86	101	59	38	6
522	0	0	0	0	1	3300	35	97	66	75	98	81	50	8
523	0	0	0	0	1	3290	35	111	68	79	97	73	45	7,1
524	0	0	0	0	1	3290	35	101	74	82	95	78	47	7,4
525	0	0	0	0	0	3490	37,5	111	81	85	95	65	42	6,2
526	0	0	0	0	0	3180	37,5	111	71	79	97	92	50	8,9
527	0	0	0	0	1	3355	37,5	117	74	86	139	57		7,9

528	0	0	0	0	0	3490	37,5	114	68	78	85	81	51	6,9
529	0	0	0	0	0	3180	37,5	107	70	79	109	81	46	8,9
530	0	0	0	0	1	3015	42,5	115	81	91	89	83	50	7,4
531	0	0	0	0	1	3400	42,5	102	69	77	92	59	38	5,4
532	0	0	0	0	1	3015	42,5	115	77	87	91	94	57	8,5
533	0	0	0	0	1	3400	42,5	103	75	84	101	49	32	5
534	0	0	0	0	0	3445	45	131	85	96	116	75	42	8,7
535	0	0	0	0	1	3290	45	107	74	84	85	89	46	7,5
536	0	0	0	0	1	3290	45	105	72	81	87	98	50	8,5
537	0	0	0	0	0	3310	45	100	74	82	92	60		5,5
538	0	0	0	0	1	3470	47,5	109	67	78	93	70	42	6,5
539	0	0	0	0	0	3470	47,5	118	87	96	83	63		5,2
540	0	0	0	0	1	3470	47,5	123	74	85	107	78	46	8,3
541	0	0	0	0	0	3505	50	115	76	86	107	61		6,5
542	0	0	0	0	1	3200	50	101	79	86	83	61		5,1
543	0	0	0	0	1	3360	52,5	133	79	93	90	86	53	7,8
544	0	0	0	0	1	3360	52,5	115	77	87	88	74	46	6,5
545	0	0	0	0	0	3160	55	99	70	79	97	65	42	6,3
546	0	0	0	0	0	3160	55	117	75	85	101	63	40	6,3
547	0	0	1	0	0	3030	55	107	73	83	84	65		5,4
548	0	0	0	0	0	3780	55	119	76	88	74	105		7,8
549	0	0	0	0	0	3830	57,5	105	70	79	92	68		6,2
550	0	0	0	0	0	3605	60	110	74	84	113	64	36	7,2
551	0	0	0	0	1	3280	60	100	71	79	107	50		5,3
552	0	0	0	0	0	3605	60	125	76	87	100	73	42	7,3
553	0	0	0	0	0	3765	62,5	119	77	87	95	70	35	6,7
554	0	0	0	0	0	3765	62,5	121	80	90	82	101	51	8,3
555	0	0	0	0	0	3765	62,5	107	79	86	109	68	40	7,5
556	0	0	0	0	0	3765	62,5	125	105	112	92	96	54	8,8
557	0	0	0	0	0	3490	65	109	75	84	112	77		8,6
558	0	0	0	0	1	3640	65	117	76	83	101	56	32	5,6
559	0	0	0	0	1	3485	65	114	79	88	80	85	50	6,7
560	0	0	0	0	1	3485	65	116	74	84	84	92	53	7,7

561	0	0	0	0	0	3465	65	133	84	95	89	87	51	7,7
562	0	0	0	0	0	3465	65	107	76	86	92	106	61	9,8
563	0	0	0	0	1	3750	67,5	107	84	91	110	92	45	10,1
564	0	0	0	0	1	3750	67,5	127	78	87	96	94	47	8,9
565	0	0	0	0	0	3490	67,5	124	81	91	108	69	42	7,4
566	0	0	0	0	1	3500	67,5	106	58	69	99	92	51	9,1
567	0	0	0	0	0	3490	67,5	108	75	84	95	66	41	6,2
568	0	0	0	0	1	3500	67,5	113	72	83	88	86	47	7,5
569	0	0	0	0	0	3490	67,5	111	71	82	80	77		6,1
570	0	0	0	0	0	3880	70	124	73	84	100	108	58	10,8
571	0	0	0	0	0	3535	70	99	74	83	108	79		8,5
572	0	0	0	0	0	3880	70	133	78	93	106	89	49	9,4
573	0	0	1	0	1	3390	70	98	69	78	77	67		5,1
574	0	0	0	0	0	4005	72,5	109	65	76	114	44	29	5
575	0	0	0	0	1	3800	72,5	102	73	81	93	76	44	7,1
576	0	0	0	0	0	4005	72,5	105	62	72	111	64	41	7,1
577	0	0	0	0	1	3585	72,5	111	69	81	96	65	46	6,3
578	0	0	0	0	1	3800	72,5	101	75	83	95	72	43	6,9
579	0	0	0	0	1	3565	72,5	110	72	82	100	71	44	7,1
580	0	0	0	0	1	3565	72,5	115	76	85	87	76	47	6,7
581	0	0	0	0	1	3585	72,5	115	72	82	104	78	53	8,2
582	0	0	0	0	0	3730	72,5	117	74	84	89	67	41	6
583	0	0	0	0	0	3730	72,5	135	85	97	106	54	34	5,8
584	0	0	0	0	1	3615	75	122	85	95	99	82	42	8
585	0	0	0	0	1	3615	75	118	83	93	91	94	51	8,5
586	0	0	0	0	1	3615	75	121	66	77	93	86	46	8
587	0	0	0	0	1	3500	77,5	103	76	85	103	67	41	6,9
588	0	0	0	0	1	3500	77,5	100	72	80	97	71	43	6,9
589	0	0	0	0	1	3900	80	93	66	74	105	61	41	6,4
590	0	0	0	0	0	4010	80	111	70	80	113	60	35	6,7
591	0	0	0	0	0	4010	80	109	77	85	91	82	47	7,5
592	0	0	0	0	0	3665	80	108	76	85	90	73	42	6,6
593	0	0	0	0	0	3675	80	94	65	73	86	94	50	8,1

594	0	0	0	0	1	3330	80	100	57	65	86	88	49	7,6
595	0	0	0	0	1	3330	80	98	63	72	89	80	45	7,2
596	0	0	0	0	0	3985	80	124	86	96	80	88	44	7,1
597	0	0	0	0	1	3900	80	102	68	77	102	62	42	6,4
598	0	0	0	0	0	3665	80	119	71	81	89	81	45	7,2
599	0	0	0	0	1	3830	80	97	66	75	100	90	49	9
600	0	0	0	0	0	3675	80	92	74	80	92	89	49	8,2
601	0	0	0	0	1	3695	80	144	80	92	90	127	57	11,4
602	0	0	0	0	1	3830	80	102	62	71	76	100	56	7,6
603	0	0	0	0	1	3695	80	122	75	87	88	135	60	11,8
604	0	0	0	0	0	3705	82,5	124	74	85	122	60	35	7,3
605	0	0	0	0	0	3875	82,5	122	75	87	76	78	45	5,9
606	0	0	0	0	0	3860	82,5	111	80	88	96	74	43	7,1
607	0	0	0	0	0	3705	82,5	110	73	83	104	70	42	7,3
608	0	0	0	0	0	3875	82,5	118	84	94	88	63	38	5,5
609	0	0	0	0	0	3730	82,5	119	80	90	99	85		8,4
610	0	0	0	0	0	3860	82,5	131	84	95	86	69	41	5,9
611	0	0	0	0	1	3960	85	134	88	99	93	86	46	8
612	0	0	0	0	1	3960	85	126	79	89	109	90	47	9,7
613	0	0	1	0	1	3905	87,5	117	76	87	98	83	45	8,2
614	0	0	1	0	1	3905	87,5	114	75	85	90	77	43	6,9
615	0	0	0	0	0	3545	90	120	81	90	82	93	46	7,6
616	0	0	0	0	1	4115	90	102	71	79	98	77	37	7,6
617	0	0	0	0	0	4035	90	122	94	103	101	59	33	6
618	0	0	0	0	0	4035	90	92	66	74	96	71	39	6,8
619	0	0	0	0	0	3545	90	138	88	100	82	79	40	6,5
620	0	0	0	0	0	3990	90	102	70	78	84	78	48	6,6
621	0	0	0	0	0	4115	92,5	122	83	93	121	70	37	8,5
622	0	0	0	0	0	3420	92,5	99	69	78	110	81	43	8,8
623	0	0	0	0	0	4115	92,5	123	86	96	109	82	43	9
624	0	0	0	0	1	3805	92,5	124	79	90	89	130	68	11,6
625	0	0	0	0	1	3805	92,5	147	88	101	84	112	59	9,5
626	0	0	0	0	1	4120	92,5	141	81	97	106	103		10,8

627	0	0	0	0	0	3420	92,5	121	71	81	106	81	44	8,5
628	0	0	0	0	0	3895	95	119	84	93	104	77	34	8
629	0	0	0	0	1	4040	95	124	69	86	90	93	52	8,4
630	0	0	0	0	0	4340	95	134	76	92	97	98		9,5
631	0	0	0	0	1	4040	95	115	73	83	91	82	48	7,5
632	0	0	0	0	0	3895	95	129	92	101	96	76	34	7,3
633	0	0	0	0	0	3895	95	156	92	109	98	81	37	7,9
634	0	0	0	0	1	3855	95	130	73	86	77	102	51	7,9
635	0	0	3	0	0	4030	95	91	66	73	96	72	45	7
636	0	0	0	0	1	3855	95	131	74	85	94	83	43	7,8
637	0	0	0	0	0	4190	97,5	122	75	85	106	112	55	11,8
638	0	0	0	0	0	4190	97,5	130	79	92	97	89	44	8,6
639	0	0	0	0	0	4445	97,5							
640	0	0	0	0	1	4270	97,5	113	77	86	104	84	48	8,7
641	0	0	0	0	0	4445	97,5	158	109	120	91	109	50	10
642	0	0	0	0	1	4270	97,5	115	77	86	94	82	48	7,7
643	0	0	0	0	0	4135	97,5	119	79	88	90	80		7,1
644	0	0	0	0	1	2845	15	130	86	97	101	89	50	9
645	0	0	0	0	1	2845	15	140	91	102	105	82	46	8,6
646	0	0	0	0	1	3325	62,5	118	72	82	105	82	46	8,6
647	0	0	0	0	1	3325	62,5	109	71	81	97	92	51	8,8
648	0	0	0	0	1	3845	82,5	110	66	77	80	93	51	7,4
649	0	0	0	0	1	3845	82,5	106	69	79	75	86	48	6,4
650	0	0	0	0	0	3145	12,5	123	72	83	78	97		7,6
651	0	0	0	0	1	3690	80	118	80	90	114	73	42	8,4
652	0	0	0	0	1	3360	67,5	150	88	101	102	87	47	8,9
653	0	0	0	0	1	3360	67,5	132	89	100	110	70	38	7,8
654	0	0	0	0	0	3455	62,5	108	78	88	99	71	44	7
655	0	0	0	0	0	3455	62,5	127	81	94	93	54	35	5
656	0	0	0	0	0	3535	60	118	79	89	123	72		8,9
657	0	0	0	0	0	3860	70	105	73	82	86	65	41	5,6
658	0	0	0	0	0	3295	30	113	82	91	92	64	41	5,9
659	0	0	0	0	0	3295	30	109	74	84	87	69	44	6

660	0	0	0	0	0	3750	60	101	81	88	104	77		8
661	0	0	0	0	0	4235	97,5	105	64	74	99	87	47	8,6
662	0	0	0	0	0	4235	97,5	90	67	74	96	75	42	7,1
663	0	0	0	0	1	3520	55	108	73	83	111	75	40	8,2
664	0	0	0	0	1	3520	55	124	79	90	99	75	41	7,2
665	0	0	0	0	1	3810	87,5	130	68	80	82	95		7,8
666	0	0	0	0	0	3675	67,5	121	73	84	88	111	60	9,8
667	0	0	0	0	0	3675	67,5	114	77	87	93	104	56	9,6
668	0	0	0	0	0	3720	72,5	125	74	86	88	111	60	9,7
669	0	0	0	0	0	3720	72,5	119	71	82	92	86	48	7,9
670	0	0	0	0	1	3400	55	123	81	90	97	94		9,1
671	0	0	2	0	0	4070	82,5	100	74	82	102	65	38	6,6
672	0	0	2	0	0	4070	82,5	102	71	80	117	66	38	7,7
673	0	0	0	0	1	3650	67,5	123	81	91	73	95		6,9
674	0	0	0	0	1	3840	87,5	111	80	89	95	68	39	6,4
675	0	0	0	0	1	3840	87,5	122	79	89	94	58	34	5,4
676	0	0	0	0	0	3670	67,5	115	76	86	87	72	42	6,3
677	0	0	0	0	0	3570	72,5	122	73	84	72	91	53	6,6
678	0	0	2	0	1	4010	87,5	103	75	83	90	84	45	7,6
679	0	0	2	0	1	4010	87,5	109	73	84	93	87	46	8,1
680	0	0	0	0	1	3810	92,5	123	73	86	72	99		7,2
681	0	0	0	0	1	3270	60	135	90	102	102	77	41	7,8
682	0	0	0	0	1	4570	97,5	117	79	89	108	93		10
683	0	0	0	0	0	3505	50	109	72	81	93	90	53	8,4
684	0	0	0	0	0	3505	50	115	70	82	74	112	65	8,3
685	0	0	0	0	1	3625	92,5	108	68	78	84	88	45	7,4
686	0	0	0	0	1	3625	92,5	93	66	74	84	75	39	6,2
687	0	0	0	0	1	3335	47,5	105	70	79	92	72	41	6,6
688	0	0	0	0	1	3335	47,5	111	80	88	99	60	34	5,9
689	0	0	0	0	0	3760	75	119	79	89	100	69		7
690	0	0	0	0	1	3190	60	113	78	88	87	63	40	5,5
691	0	0	0	0	1	3190	60	97	55	65	111	64	40	7,1
692	0	0	1	0	0	3620	75	111	76	86	84	99	50	8,3

693	0	0	1	0	0	3620	75	107	76	85	83	97	52	8,1
694	0	0	1	0	0	3620	75	116	81	90	83	94	51	7,8
695	0	0	1	0	1	3120	17,5	106	73	82	91	73		6,7
696	0	0	0	0	1	3035	32,5	102	75	83	91	57		5,2
697	0	0	1	0	1	3550	82,5	109	77	86	86	73	40	6,2
698	0	0	0	0	1	3195	62,5	122	80	91	91	80	47	7,2
699	0	0	0	0	1	3195	62,5	116	84	94	95	59	36	5,6
700	0	0	2	0	0	3530	55	106	68	79	87	79	47	6,8
701	0	0	2	0	0	3530	55	103	74	83	82	69	42	5,6
702	0	0	0	0	0	3420	57,5	98	67	75	91	63		5,7
703	0	0	0	0	0	3840	67,5	103	72	81	90	82		7,4
704	0	0	0	0	1	2645	12,5	122	68	80	84	123		10,3
705	0	0	0	0	1	2945	15	128	81	93	96	100		9,5
706	0	0	0	0	0	3105	25	118	80	91	91	99		9
707	0	0	0	0	0	3370	27,5	108	78	86	98	108		10,6
708	0	0	0	0	1	3360	27,5	101	68	77	86	72		6,2
709	0	0	0	0	1	2885	30	117	81	91	88	74		6,5
710	0	0	0	0	0	3280	30	109	73	82	90	61		5,5
711	0	0	0	0	0	3315	32,5	110	75	84	102	68		6,9
712	0	0	0	0	1	3060	35	100	67	76	91	65		5,9
713	0	0	0	0	0	3335	35	116	68	77	98	68		6,7
714	0	0	0	0	1	3425	47,5	111	74	85	98	67		6,5
715	0	0	0	0	0	3105	50	105	75	83	77	68		5,2
716	0	0	0	0	0	3560	57,5	128	85	95	85	74		6,3
717	0	0	0	0	1	3560	60	132	81	95	99	74	42	7,3
718	0	0	0	0	1	3560	60	131	74	89	89	93	52	8,2
719	0	0	0	0	1	3380	67,5	113	75	85	93	59		5,5
720	0	0	0	0	0	3700	70	109	77	86	114	89		10,1
721	0	0	0	0	0	3380	70	108	71	81	106	69		7,4
722	0	0	0	0	0	3560	70	114	80	89	100	75		7,5
723	0	0	0	0	1	3750	75	114	75	84	112	82		9,2
724	0	0	0	0	0	3165	77,5	110	71	79	95	75		7,1
725	0	0	0	0	0	3840	80	128	92	103	139	45		6,2

726	0	0	0	0	0	4000	80	90	60	69	105	48		5
727	0	0	2	0	1	3375	80	132	75	86	107	80		8,5
728	0	0	1	0	0	3665	87,5	116	80	89	107	55		5,9
729	0	0	0	0	0	4140	87,5	103	77	85	78	94		7,3
730	0	0	0	0	1	4085	90	102	68	77	87	86		7,5
731	0	0	0	0	0	3995	95	113	83	93	82	81		6,7
732	0	0	0	0	1	3375	70	124	80	91	77	70		5,4
733	0	0	0	0	0	3155	32,5	98	55	67	104	82	48	8,4
734	0	0	0	0	0	3155	32,5	115	74	85	103	68	41	7
735	0	0	0	0	0	4050	75	122	71	83	87	86	49	7,5
736	0	0	0	0	0	4050	75	117	77	88	93	89	51	8,3
737	0	0	0	0	1	2715	32,5	121	81	91	87	58		5,1
738	0	0	0	0	1	2985	17,5	103	69	78	86	73	45	6,3
739	0	0	0	0	1	3300	45	119	82	92	97	72	43	7
740	0	0	0	0	1	3300	45	127	79	92	93	67	41	6,2
741	0	0	0	0	1	2980	60	123	87	97	102	55		5,6
742	0	0	0	0	1	3340	37,5	122	83	93	93	98	52	9,1
743	0	0	0	0	1	3340	37,5	117	83	93	100	87	47	8,6
744	0	0	0	0	1	3935	87,5	103	75	83	104	90		9,3
745	0	0	0	0	1	3780	80	153	80	96	94	102		9,5
746	0	0	0	0	0	3985	72,5	152	83	94	90	78		7
747	0	0	0	0	0	3635	77,5	107	67	77	80	110	59	8,8
748	0	0	0	0	0	3635	77,5	116	66	78	90	100	54	9
749	0	0	0	0	0	3710	82,5	93	58	69	95	89	55	8,4
750	0	0	0	0	0	3710	82,5	92	65	72	114	70	44	8,1
751	0	0	0	0	1	2860	22,5	116	72	83	113	53		5,9
752	0	0	0	0	0	3600	60	103	71	80	83	71		5,9
753	0	0	0	0	0	4670	97,5	118	86	91	108	68		7,4
754	0	0	0	0	1	3145	47,5	121	83	92	127	65		8,3
755	0	0	0	0	0	3995	80	142	83	96	97	102		9,9
756	0	0	0	0	1	3275	42,5	115	76	86	105	68	39	7,1
757	0	0	0	0	1	3275	42,5	103	67	77	110	58	34	6,3
758	0	0	0	0	0	3430	45	134	87	99	100	73	41	7,3

759	0	0	0	0	0	3430	45	118	82	92	103	65	36	6,7
760	0	0	0	0	0	3310	62,5	124	73	85	82	101	52	8,3
761	0	0	0	0	0	3310	62,5	103	67	75	86	107	51	9,2
762	0	0	0	0	1	2825	45	106	72	81	82	102	57	8,4
763	0	0	0	0	1	2825	45	107	68	80	77	96	54	7,4
764	0	0	0	0	0	3440	45	109	75	82	100	71	41	7,1
765	0	0	0	0	0	3440	45	103	67	76	86	97	54	8,3
766	0	0	0	0	0	3895	85	111	79	88	132	65	34	8,5
767	0	0	0	0	0	3895	85	126	74	89	114	57	30	6,5
768	0	0	0	0	0	3950	97,5	124	80	90	112	91	41	10,2
769	0	0	0	0	0	3950	97,5	117	92	98	122	75	34	9,2
770	0	0	0	0	1	3450	75	105	75	83	97	72	42	7
771	0	0	0	0	1	3450	75	113	72	82	93	74	44	6,9
772	0	0	0	0	1	3145	27,5	108	73	83	112	70	46	7,9
773	0	0	0	0	1	3145	27,5	105	67	76	125	72	45	8,9
774	0	0	0	0	0	3880	72,5	116	65	77	104	66	42	6,9
775	0	0	0	0	0	3880	72,5	105	72	82	107	56	37	6
776	0	0	0	0	0	3240	25	109	78	88	109	52		5,7
777	0	0	0	0	1	3405	42,5	131	81	94	101	71		7,1
778	0	0	0	0	0	2875	20	107	73	82	90	63		5,7
779	0	0	0	0	1	3180	22,5	135	82	92	97	159	75	15,5
780	0	0	0	0	1	3180	22,5	117	81	87	100	118	56	11,8
781	0	0	0	0	1	3215	25	119	76	87	99	75	41	7,4
782	0	0	0	0	1	3215	25	113	76	86	106	74	40	7,9
783	0	0	0	0	1	3220	37,5	124	82	93	115	62	37	7,1
784	0	0	0	0	1	3220	37,5	122	75	86	87	85	52	7,4
785	0	0	0	0	1	3220	37,5	116	75	85	96	79	48	7,6
786	0	0	2	0	0	3050	45	103	65	74	89	85	51	7,6
787	0	0	2	0	0	3050	45	110	69	78	90	75	46	6,7
788	0	0	0	0	1	3145	45	136	93	104	91	116		10,5
789	0	0	0	0	0	3630	62,5	118	80	89	116	73	41	8,4
790	0	0	0	0	0	3630	62,5	106	80	88	104	64	37	6,7
791	0	0	0	0	0	3380	70	118	71	82	90	86	48	7,7

792	0	0	0	0	0	3380	70	106	68	78	90	78	45	7
793	0	0	0	0	0	3960	70	119	83	89	101	78		7,9
794	0	0	0	0	0	3430	72,5	116	74	84	81	78	46	6,3
795	0	0	0	0	0	3430	72,5	113	77	87	79	73	44	5,8
796	0	0	0	0	0	3635	77,5	121	85	94	91	72	46	6,6
797	0	0	0	0	0	3635	77,5	125	95	103	114	46	30	5,2
798	0	0	0	0	1	4000	90	107	66	75	88	93	48	8,2
799	0	0	0	0	1	4000	90	111	75	84	98	74	38	7,3
800	0	0	0	0	0	4735	97,5	106	71	79	100	92	49	9,2
801	0	0	0	0	0	4735	97,5	100	69	78	88	87	48	7,7
802	0	0	0	0	1	2775	15	115	77	87	86	75	44	6,4
803	0	0	0	0	1	2775	15	101	72	81	84	74	45	6,2
804	0	0	0	0	1	3330	67,5	97	63	73	100	82	49	8,2
805	0	0	0	0	1	3330	67,5	116	67	79	91	76	47	7
806	0	0	0	0	0	3475	37,5	114	73	82	102	87	44	8,8
807	0	0	0	0	0	3475	37,5	126	75	88	89	103	53	9,2
808	0	0	1	0	1	3015	30	108	77	87	109	60		6,5
809	0	0	1	0	1	3845	87,5	133	78	90	110	65	40	7,1
810	0	0	1	0	1	3845	87,5	123	81	91	134	48	31	6,4
811	0	0	0	0	0	4025	82,5	114	74	86	92	87	51	8
812	0	0	0	0	0	4025	82,5	122	78	90	91	74	45	6,8
813	0	0	2	0	1	3515	65	127	84	96	87	77		6,7
814	0	0	0	0	1	3650	87,5	111	75	85	86	92	49	8
815	0	0	0	0	1	3650	87,5	123	75	88	99	97	51	9,6
816	0	0	0	0	0	3075	22,5	115	73	83	97	61	39	5,9
817	0	0	0	0	1	3285	45	109	86	91	85	93	54	7,9
818	0	0	0	0	1	3285	45	127	82	95	78	83	50	6,4
819	0	0	0	0	0	3580	75	119	77	86	106	64	40	6,8
820	0	0	0	0	1	3200	35	106	67	78	80	82	48	6,6
821	0	0	0	0	1	3200	35	102	67	77	86	68	41	5,9
822	0	0	0	0	0	3970	92,5	143	89	106	136	69		9,3
823	0	0	0	0	0	3550	70	121	62	80	72	100		7,2
824	0	0	0	0	1	3185	15	103	72	81	114	68	40	7,8

825	0	0	0	0	1	3795	72,5	124	82	91	91	90	48	8,2
826	0	0	0	0	1	3795	72,5	121	78	89	83	96	55	7,9
827	0	0	0	0	1	3795	72,5	111	83	91	92	72	42	6,7
828	0	0	0	0	1	3595	72,5	113	80	90	83	89		7,4
829	0	0	0	0	0	3185	37,5	111	74	84	103	51	32	5,3
830	0	0	0	0	1	3335	80	119	83	93	111	55		6
831	0	0	0	0	0	3815	57,5	102	69	78	83	74	42	6,1
832	0	0	0	0	0	3815	57,5	114	68	78	91	75	43	6,8
833	0	0	0	0	0	3785	77,5	109	66	76	82	83	54	6,8
834	0	0	0	0	0	3785	77,5	109	71	80	85	70	47	6
835	0	0	0	0	0	3530	55	115	75	84	96	48		4,6
836	0	0	0	0	0	3620	50	139	80	97	109	91		9,9
837	0	0	0	0	1	3015	12,5	98	67	76	99	66	39	6,6
838	0	0	0	0	1	3015	12,5	111	74	84	95	65	40	6,2
839	0	0	0	0	0	3990	72,5	110	72	82	96	80	44	7,7
840	0	0	0	0	0	3990	72,5	107	77	86	114	71	39	8,2
841	0	0	0	0	0	3240	72,5	124	78	88	91	77	42	7,1
842	0	0	0	0	0	3240	72,5	124	80	90	78	80	43	6,2
843	0	0	0	0	1	4130	92,5	124	81	91	109	71	37	7,8
844	0	0	0	0	1	4130	92,5	113	85	94	94	94	48	8,9
845	0	0	1	0	0	3455	27,5	134	81	94	90	102	51	9,2
846	0	0	1	0	0	3455	27,5	141	85	98	79	104	52	8,2
847	0	0	0	0	1	3380	70	109	66	76	96	83	44	7,9
848	0	0	0	0	1	3380	70	94	60	64	78	113	58	8,8
849	0	0	0	0	1	3120	40	113	85	94	98	57	33	5,6
850	0	0	0	0	1	3120	40	107	78	86	91	71	41	6,5
851	0	0	0	0	0	3285	60	117	80	91	96	76	46	7,3
852	0	0	0	0	0	3285	60	123	91	101	97	68	42	6,6
853	0	0	0	0	0	4170	90	105	69	78	86	79		6,8
854	0	0	0	0	1	3260	60	110	81	89	97	76	40	7,4
855	0	0	0	0	1	3260	60	108	78	87	87	103	53	8,9
856	0	0	0	0	1	3190	35	144	88	101	101	74		7,4
857	0	0	0	0	1	3035	22,5	135	85	95	113	62	40	7

858	0	0	0	0	1	3035	22,5	114	79	88	101	63	42	6,4
859	0	0	0	0	1	4030	87,5	105	73	82	116	55	34	6,4
860	0	0	0	0	1	4030	87,5	97	71	79	121	51	30	6,2
861	0	0	0	0	0	3505	40	146	89	104	117	76	38	8,8
862	0	0	0	0	0	3505	40	153	93	106	108	92	45	9,9
863	0	0	0	0	0	3505	40	139	82	100	97	84	42	8,2
864	0	0	3	0	0	3730	90	84	47	56	71	103	56	7,3
865	0	0	3	0	0	3730	90	88	56	64	88	78	43	6,8
866	0	0	0	0	0	4170	90	115	66	78	96	123	53	11,9
867	0	0	0	0	0	4170	90	123	73	82	94	134	57	12,6
868	0	0	0	0	1	3595	75	99	66	75	89	95	54	8,4
869	0	0	0	0	1	3595	75	99	66	75	89	95	54	8,4
870	0	0	0	0	0	4675	97,5	124	69	83	93	75	45	7
871	0	0	0	0	0	4675	97,5	98	63	72	95	58	36	5,4
872	0	0	0	0	1	3455	75	114	76	86	105	67	35	7,1
873	0	0	0	0	1	3455	75	124	79	88	106	61	32	6,5
874	0	0	0	0	1	2870	27,5	97	67	76	105	76	46	8
875	0	0	0	0	1	2870	27,5	105	73	82	99	74	45	7,3
876	0	0	0	0	1	3145	20	111	71	81	105	65		6,8
877	0	0	0	0	0	3400	55	93	66	75	80	92	52	7,3
878	0	0	0	0	0	3400	55	102	66	76	90	60	34	5,4
879	0	0	0	0	0	3310	45	133	89	99	97	68	37	6,7
880	0	0	0	0	0	3310	45	121	88	97	91	76	41	6,9
881	0	0	1	0	0	4070	82,5	124	91	100	80	96	43	7,8
882	0	0	1	0	0	4070	82,5	138	90	106	91	89	40	8,1
883	0	0	0	0	0	4210	85	97	71	79	98	68	36	6,6
884	0	0	0	0	0	4210	85	116	67	81	92	77	41	7
885	0	0	0	0	0	3650	65	101	69	78	77	99	55	7,6
886	0	0	0	0	0	3650	65	99	70	79	92	79	45	7,2
887	0	0	0	0	0	3580	72,5	102	77	85	117	56		6,6
888	0	0	0	0	0	3920	65	108	66	76	75	118	67	8,8
889	0	0	0	0	0	3920	65	99	67	77	83	88	51	7,3
890	0	0	1	0	0	3805	77,5	105	62	70	105	77	43	8

891	0	0	0	0	0	3680	67,5	121	75	88	93	123	55	11,5
892	0	0	0	0	0	3680	67,5	117	75	86	88	108	49	9,5
893	0	0	0	0	0	3125	27,5	122	82	93	109	56		6,1
894	0	0	0	0	1	3975	97,5	124	89	99	117	71	33	8,3
895	0	0	0	0	1	3975	97,5	115	85	94	97	78	36	7,6
896	0	0	1	0	0	4435	97,5	118	77	88	81	81		6,5
897	0	0	0	0	1	3020	20	102	66	76	99	77	44	7,6
898	0	0	0	0	1	3020	20	91	63	70	102	75	42	7,7
899	0	0	0	0	0	2830	32,5	100	74	82	100	63	40	6,3
900	0	0	0	0	0	2830	32,5	105	76	84	89	50	33	4,4
901	0	0	0	0	1	3275	32,5	121	77	87	82	66		5,4
902	0	0	0	0	0	3425	57,5	121	74	82	103	58	35	6,1
903	0	0	0	0	0	3425	57,5	130	82	94	99	80	47	7,9
904	0	0	0	0	0	4095	80	110	76	86	94	80		7,5
905	0	0	0	0	1	3705	90	102	73	81	112	70	37	7,8
906	0	0	0	0	1	3705	90	114	67	78	97	109	56	10,5
907	0	0	0	0	0	3780	77,5	96	65	73	100	74	44	7,5
908	0	0	0	0	0	3780	77,5	103	72	80	123	58	35	7,1
909	0	0	0	0	1	4070	92,5	142	91	102	87	106		9,3
910	0	0	0	0	1	2450	42,5	124	78	89	86	83		7,1
911	0	0	0	0	1	3485	50	106	79	87	91	79		7,2
912	0	0	0	0	1	3790	77,5	113	74	85	102	73		7,4
913	0	0	0	0	1	3430	72,5	111	71	83	79	106	57	8,4
914	0	0	0	0	1	3430	72,5	113	75	85	82	80	45	6,6
915	0	0	2	0	0	3070	12,5	119	78	89	98	85	42	8,2
916	0	0	2	0	0	3070	12,5	122	83	93	93	76	38	7
917	0	0	0	0	1	3390	32,5	123	75	87	107	50		5,4
918	0	0	0	0	0	3010	40	146	95	106	124	66	34	8,2
919	0	0	0	0	0	3010	40	129	88	97	121	68	37	8,3
920	0	0	0	0	1	3275	32,5	124	75	86	118	69		8,1
921	0	0	0	0	1	3770	92,5	106	68	80	90	88		7,8
922	0	0	0	0	0	3250	27,5	119	77	89	95	51	36	4,8
923	0	0	0	0	0	3250	27,5	109	78	87	96	48	33	4,6

924	0	0	1	0	0	3395	40	113	72	81	104	87		9
925	0	0	0	0	1	3125	45	111	72	81	95	83	46	7,9
926	0	0	0	0	1	3125	45	128	86	97	99	62	35	6,1
927	0	0	0	0	1	3070	25	108	63	72	85	84	49	7,1
928	0	0	0	0	1	3070	25	131	74	82	84	71	43	6
929	0	0	0	0	1	2985	12,5	107	62	73	71	92	54	6,5
930	0	0	0	0	1	2985	12,5	131	75	88	69	94	56	6,4
931	0	0	1	0	1	2875	22,5	108	71	79	86	82	47	7,1
932	0	0	0	0	0	4270	97,5	117	76	86	103	84	42	8,7
933	0	0	0	0	0	4270	97,5	136	85	95	102	70	36	7,2
934	0	0	0	0	1	3420	57,5	122	85	94	96	65	36	6,2
935	0	0	0	0	1	3420	57,5	116	76	87	89	87	48	7,8
936	0	0	0	0	0	3785	77,5	115	79	88	125	80	37	10
937	0	0	0	0	0	3785	77,5	109	66	75	104	96	45	10
938	0	0	0	0	0	3535	40	131	82	92	108	68	39	7,3
939	0	0	0	0	0	3535	40	131	82	94	86	80	46	6,9
940	0	0	0	0	1	3330	65	98	69	78	100	87	45	8,7
941	0	0	0	0	1	3330	65	100	75	84	91	82	42	7,5
942	0	0	0	0	0	3680	67,5	133	83	93	119	90	45	10,7
943	0	0	0	0	0	3680	67,5	126	76	87	104	82	42	8,5
944	0	0	0	0	1	3480	52,5	116	77	85	78	76		6
945	0	0	0	0	0	3385	70	114	73	83	104	79	46	8,2
946	0	0	0	0	0	3385	70	119	83	93	113	66	39	7,4
947	0	0	0	0	1	2880	27,5	119	75	86	93	78	45	7,2
948	0	0	0	0	0	3640	77,5	113	78	87	104	96	47	9,9
949	0	0	0	0	0	3640	77,5	109	66	77	100	91	46	9,1
950	0	0	2	0	1	3755	85	109	73	82	104	105	53	10,9
951	0	0	2	0	1	3755	85	124	78	89	123	75	39	9,3
952	0	0	0	0	1	3910	87,5	121	72	86	98	76		7,5
953	0	0	0	0	1	4140	92,5	141	81	90	98	77	42	7,5
954	0	0	0	0	1	3430	45	109	75	83	99	86	50	8,5
955	0	0	0	0	1	3430	45	99	70	79	95	71	42	6,7
956	0	0	0	0	0	4160	95	115	75	86	88	127	65	11,2

957	0	0	0	0	0	4160	95	121	87	96	98	100	53	9,8
958	0	0	0	0	1	3295	22,5	130	74	90	100	77	44	7,7
959	0	0	0	0	0	4260	97,5	121	74	86	101	97	52	9,8
960	0	0	0	0	0	4260	97,5	119	73	85	101	71	40	7,2
961	0	0	0	0	0	2995	50	115	81	89	116	60	31	6,9
962	0	0	0	0	0	2995	50	125	82	93	110	59	30	6,5
963	0	0	0	0	1	3870	95	118	82	92	95	78	43	7,3
964	0	0	0	0	1	3870	95	121	82	92	95	74	40	7
965	0	0	0	0	1	4070	97,5	102	72	79	111	69	38	7,7
966	0	0	0	0	1	4070	97,5	105	70	79	94	82	46	7,7
967	0	0	0	0	1	4070	97,5	110	72	82	93	79	45	7,3
968	0	0	0	0	0	3140	17,5	109	73	83	86	67	40	5,7
969	0	0	0	0	0	3140	17,5	111	72	81	90	73	44	6,6
970	0	0	1	0	1	3245	40	143	81	93	96	89	45	8,6
971	0	0	1	0	1	3245	40	124	83	92	86	93	47	8
972	0	0	0	0	1	3230	37,5	123	84	96	134	50		6,7
973	0	0	0	0	0	3730	60	100	83	89	103	71		7,3
974	0	0	0	0	0	2990	17,5	125	85	97	95	62	38	6
975	0	0	0	0	0	2990	17,5	133	82	94	94	77	45	7,2
976	0	0	0	0	1	3640	52,5	102	73	82	104	129	59	13,4
977	0	0	0	0	1	3640	52,5	110	68	78	98	117	55	11,4
978	0	0	0	0	0	3420	32,5	121	76	84	109	63		6,9
979	0	0	0	0	1	3500	55	110	80	87	104	60	38	6,2
980	0	0	0	0	1	3065	22,5	98	63	73	101	62		6,2
981	0	0	0	0	1	3005	55	142	91	102	92	105	54	9,7
982	0	0	0	0	1	3005	55	142	94	107	99	77	41	7,7
983	0	0	0	0	1	3055	22,5	118	80	90	103	81	46	8,3
984	0	0	0	0	1	3055	22,5	121	83	91	92	72	42	6,7
985	0	0	0	0	0	3405	22,5	116	76	86	87	117	64	10,1
986	0	0	0	0	0	3405	22,5	116	70	81	92	102	56	9,4
987	0	0	0	0	0	3600	47,5	110	76	86	121	53	33	6,4
988	0	0	0	0	0	3600	47,5	109	70	83	125	59	36	7,4
989	0	0	1	0	1	3745	67,5	123	81	91	84	102	60	8,6

990	0	0	1	0	1	3745	67,5	124	54	69	91	110	64	9,9
991	0	0	0	0	1	2275	30	123	80	89	92	84	46	7,7
992	0	0	0	0	1	2275	30	114	71	81	90	98	54	8,8
993	0	0	0	0	1	3130	80	123	90	99	104	75	41	7,8
994	0	0	0	0	1	3215	72,5	115	84	93	90	88	44	7,9
995	0	0	0	0	1	3215	72,5	133	89	98	105	71	35	7,5
996	0	0	0	0	1	3215	72,5	130	87	98	102	76	38	7,7
997	0	0	0	0	0	3515	40	119	81	91	104	84	44	8,8
998	0	0	0	0	0	3515	40	125	82	94	113	74	40	8,4
999	0	0	0	0	0	3840	90	106	75	84	99	74		7,3
1000	0	0	0	0	0	3410	57,5	110	69	79	90	59		5,3
1001	0	0	0	0	1	3400	70	110	71	82	90	95		8,5
1002	0	0	0	0	0	4495	95	133	94	103	117	110		12,9
1003	0	0	0	0	1	3810	87,5	125	85	95	104	128	53	13,2
1004	0	0	0	0	1	3810	87,5	119	68	82	98	131	56	12,8
1005	0	0	0	0	1	3810	87,5	110	80	88	94	138	59	12,9
1006	0	0	0	0	0	3670	80	119	72	82	89	139	62	12,4
1007	0	0	0	0	0	3670	80	102	69	76	94	110	50	10,4
1008	0	0	0	0	0	3670	80	110	78	86	100	135	63	13,4
1009	0	0	0	0	0	3675	42,5	117	76	86	108	86	46	9,3
1010	0	0	0	0	0	3675	42,5	128	82	93	105	95	50	10
1011	0	0	0	0	0	3915	75	132	80	90	90	105	51	9,5
1012	0	0	0	0	0	3915	75	140	82	92	96	100	48	9,6
1013	0	0	0	0	1	4035	95	108	72	82	76	110	61	8,4
1014	0	0	0	0	1	4035	95	124	74	86	89	86	49	7,6
1015	0	0	0	0	0	2490	37,5	103	80	85	101	76		7,7
1016	0	0	0	0	1	1600	65	115	85	94	106	68	46	7,2
1017	0	0	0	0	1	1600	65	113	78	88	115	59	41	6,8
1018	0	0	0	0	1	2970	50	114	69	80	97	60		5,8
1019	0	0	0	0	0	3395	30	121	84	93	112	66	40	7,4
1020	0	0	0	0	0	3395	30	119	82	92	110	59	37	6,5
1021	0	0	1	0	0	3260	42,5	108	79	88	93	65		6,1
1022	0	0	0	0	0	3410	30	136	82	96	104	73	38	7,5

1023	0	0	0	0	0	3410	30	116	77	87	97	87	45	8,5
1024	0	0	1	0	0	3475	67,5	116	71	84	82	100	47	8,2
1025	0	0	0	0	1	3740	82,5	114	71	82	97	70		6,7
1026	0	0	0	0	0	3180	20	124	72	87	98	67		6,6
1027	0	0	0	0	1	3325	37,5	98	64	72	68	94	60	6,4
1028	0	0	0	0	1	3325	37,5	100	67	76	69	93	59	6,4
1029	0	0	0	0	0	3510	40	117	77	86	102	69	40	7
1030	0	0	0	0	0	3510	40	130	92	101	93	64	37	5,9
1031	0	0	0	0	0	3545	55	118	87	96	97	66	35	6,3
1032	0	0	0	0	0	3545	55	132	85	97	113	58	31	6,5
1033	0	0	0	0	0	3545	55	131	81	95	105	58	31	6,1
1034	0	0	0	0	1	3235	75	138	86	98	103	81	39	8,4
1035	0	0	0	0	1	3235	75	144	82	101	82	105	52	8,6
1036	0	0	0	0	0	3980	77,5	166	96	110	98	84	42	8,2
1037	0	0	0	0	0	4130	87,5	100	72	81	106	82	42	8,7
1038	0	0	0	0	0	2975	17,5	130	78	89	85	89		7,5
1039	0	0	0	0	1	3190	35	143	89	98	106	84	43	8,9
1040	0	0	0	0	1	3190	35	140	91	103	104	78	41	8,1
1041	0	0	1	0	1	3180	60	128	90	101	107	65	37	6,9
1042	0	0	1	0	1	3180	60	133	90	101	120	70	40	8,4
1043	0	0	0	0	1	2765	15	102	71	80	99	84	47	8,3
1044	0	0	0	0	1	2765	15	106	74	83	97	70	40	6,8
1045	0	0	0	0	1	4385	97,5	115	78	87	96	88	42	8,5
1046	0	0	0	0	1	4385	97,5	109	75	84	89	86	43	7,6
1047	0	0	0	0	1	4385	97,5	130	72	85	97	95	47	9,2
1048	0	0	0	0	1	4950	100	111	70	80	94	104		9,8
1049	0	0	0	0	0	3295	30	121	64	79	67	88	56	5,9
1050	0	0	0	0	0	3295	30	100	62	71	85	78	48	6,6
1051	0	0	0	0	0	4080	92,5	111	73	81	97	83		8
1052	0	0	0	0	1	3535	57,5	98	66	73	92	72	49	6,7
1053	0	0	0	0	1	3535	57,5	106	73	83	102	57	40	5,8
1054	0	0	2	0	1	2830	12,5	108	73	83	103	58	37	6
1055	0	0	2	0	1	2830	12,5	96	66	74	104	62	39	6,4

1056	0	0	0	0	1	2965	15	134	90	101	115	58		6,6
1057	0	0	0	0	0	3000	52,5	121	79	90	93	83	43	7,8
1058	0	0	0	0	0	3000	52,5	130	77	94	82	90	47	7,4
1059	0	0	0	0	1	3600	12	131	84	97	103	61	36	6,3
1060	0	0	0	0	1	3600	62,5	98	70	79	97	75	41	7,2
1061	0	0	0	0	1	3600	62,5	124	89	99	119	57	32	6,8
1062	0	0	0	0	0	3250	42,5	117	75	87	88	102	53	8,9
1063	0	0	0	0	0	3250	42,5	131	77	87	73	94	49	6,9
1064	0	0	1	0	0	3475	67,5	113	81	91	103	88	41	9,1
1065	0	0	0	0	0	3535	72,5	109	62	75	95	82	46	7,8
1066	0	0	0	0	0	3535	72,5	127	89	100	105	66	38	6,9
1067	0	0	0	0	0	3535	72,5	125	84	95	98	78	44	7,6
1068	0	0	0	0	1	3430	35	110	77	86	127	56	32	7
1069	0	0	0	0	1	3430	35	107	77	87	120	53	30	6,4
1070	0	0	0	0	1	3430	35	114	83	92	120	59	34	7
1071	0	0	0	0	1	3455	62,5	126	87	95	107	67	38	7,1
1072	0	0	0	0	1	3455	62,5	101	66	76	114	70	39	8
1073	0	0	0	0	1	3180	22,5	117	83	93	92	67		6,1
1074	0	0	0	0	0	3935	85	110	77	87	79	70		5,5
1075	0	0	0	0	1	3465	62,5	117	70	81	76	88	46	6,6
1076	0	0	0	0	1	3465	62,5	100	61	72	75	104	54	7,8
1077	0	0	0	0	1	3080	17,5	101	65	75	89	62		5,5
1078	0	0	0	0	1	3790	92,5	106	68	76	103	63	36	6,5
1079	0	0	0	0	0	4300	97,5	130	78	91	111	107	47	11,9
1080	0	0	0	0	0	4300	97,5	127	81	94	100	104	46	10,4
1081	0	0	1	0	1	4060	92,5	136	86	96	103	82		8,4
1082	0	0	0	0	0	2960	15	108	67	78	94	81	45	7,6
1083	0	0	0	0	0	2960	15	99	69	78	88	74	42	6,5
1084	0	0	0	0	0	3880	62,5	105	71	80	93	86	48	8
1085	0	0	0	0	0	3880	62,5	109	72	82	83	96	53	8
1086	0	0	0	0	0	3455	32,5	122	85	96	103	78	42	8
1087	0	0	0	0	0	3455	32,5	118	85	95	104	93	50	9,7
1088	0	0	0	0	0	3815	80	105	68	78	79	72	48	5,7

1089	0	0	0	0	0	2940	35	122	80	90	80	104		8,4
1090	0	0	0	0	1	3160	15	100	68	77	106	41	28	4,4
1091	0	0	0	0	1	3160	15	99	70	79	117	41	28	4,8
1092	0	0	0	0	1	3945	92,5	159	79	92	126	82		10,4
1093	0	0	0	0	0	3550	55	106	74	84	107	42	31	4,5
1094	0	0	0	0	1	4130	97,5	106	79	88	96	47		4,5
1095	0	0	0	0	0	3185	37,5	111	74	84	98	91	49	8,9
1096	0	0	0	0	0	3185	37,5	107	76	86	102	90	49	9,2
1097	0	0	0	0	0	3145	17,5	130	60	77	100	95	48	9,6
1098	0	0	0	0	0	3145	17,5	122	66	77	98	113	59	11,1
1099	0	0	0	0	1	3510	80	134	88	101	110	80	42	8,8
1100	0	0	0	0	1	3510	80	141	83	95	116	68	37	7,8
1101	0	0	0	0	1	3510	80	127	82	93	94	75	41	7,1
1102	0	0	0	0	0	3340	72,5	118	86	96	97	73		7
1103	0	0	1	0	1	3120	45	110	67	77	78	125	64	9,8
1104	0	0	1	0	1	3120	45	117	71	77	78	129	68	10,1
1105	0	0	1	0	1	3120	45	119	66	79	66	120	64	7,9
1106	0	0	0	0	0	3430	30	100	71	78	89	72	43	6,4
1107	0	0	0	0	0	3430	30	117	78	88	111	55	33	6,2
1108	0	0	0	0	0	3750	85	110	66	76	111	69	37	7,6
1109	0	0	0	0	0	3750	85	111	64	77	87	63	34	5,5
1110	0	0	3	0	1	2515	17,5	124	80	90	101	83	45	8,4
1111	0	0	3	0	1	2515	17,5	116	70	83	86	101	55	8,7
1112	0	0	3	0	1	2515	17,5	128	73	88	88	90	50	7,9
1113	0	0	0	0	0	3520	40	121	76	89	91	57		5,2
1114	0	0	0	0	1	4135	97,5	99	61	71	80	120	64	9,6
1115	0	0	0	0	1	4135	97,5	105	69	79	83	81	45	6,7
1116	0	0	0	0	0	3640	65	100	72	80	99	74		7,3
1117	0	0	0	0	1	3755	92,5	124	79	89	97	85	43	8,3
1118	0	0	0	0	1	3755	92,5	136	75	86	109	76	37	8,4
1119	0	0	0	0	1	3755	92,5	129	80	90	103	77	38	8
1120	0	0	0	0	0	3340	50	113	73	83	100	52		5,2
1121	0	0	0	0	1	3745	85	100	69	78	112	70	42	7,9

1122	0	0	0	0	1	3745	85	122	64	78	121	65	40	7,8
1123	0	0	0	0	1	3240	57,5	124	85	95	81	103	48	8,3
1124	0	0	0	0	1	3240	57,5	149	80	97	97	94	44	9,2
1125	0	0	1	0	1	2545	17,5	108	70	79	99	69	46	6,9
1126	0	0	1	0	1	2545	17,5	98	68	77	83	69	46	5,7
1127	0	0	0	0	0	3105	17,5	118	83	93	124	56		7
1128	0	0	0	0	0	3140	17,5	126	81	93	89	104	50	9,2
1129	0	0	0	0	1	2985	17,5	130	74	90	90	78		7
1130	0	0	0	0	0	3140	17,5	130	87	98	83	100	49	8,3
1131	0	0	0	0	1	2820	22,5	127	73	84	112	56	33	6,3
1132	0	0	0	0	0	3215	22,5	121	73	79	85	70	45	6
1133	0	0	0	0	0	3215	22,5	139	74	90	108	76	47	8,3
1134	0	0	0	0	1	2820	22,5	114	67	74	87	73	44	6,3
1135	0	0	0	0	0	3330	25	109	50	62	97	90	49	8,8
1136	0	0	0	0	0	3330	25	99	61	70	89	91	51	8,1
1137	0	0	0	0	0	3330	25	108	63	72	91	87	49	7,9
1138	0	0	0	0	1	3205	25	115	66	77	86	89	52	7,7
1139	0	0	0	0	1	3205	25	110	68	77	82	79	48	6,5
1140	0	0	0	0	1	2975	25	117	76	87	89	81		7,2
1141	0	0	0	0	1	3255	27,5	118	69	79	99	77	44	7,6
1142	0	0	0	0	0	3425	27,5	108	78	87	99	72	41	7,1
1143	0	0	0	0	1	3110	27,5	110	63	75	89	79	46	7
1144	0	0	0	0	1	3105	27,5	114	73	82	83	66	41	5,4
1145	0	0	0	0	0	3425	27,5	98	69	77	99	77	43	7,6
1146	0	0	0	0	1	2990	27,5	107	61	76	87	102	53	8,8
1147	0	0	0	0	1	3105	27,5	109	66	76	84	87	52	7,3
1148	0	0	0	0	1	2990	27,5	117	78	88	81	105	55	8,5
1149	0	0	0	0	1	3325	37,5	119	83	92	114	78	44	9
1150	0	0	0	0	1	3325	37,5	117	84	93	111	77	44	8,5
1151	0	0	0	0	1	3085	37,5	123	84	95	87	72	44	6,3
1152	0	0	0	0	1	3085	37,5	116	76	85	99	75	45	7,4
1153	0	0	0	0	0	2975	37,5	107	71	81	98	66	41	6,4
1154	0	0	0	0	1	2770	40	110	81	86	93	45		4,2

1155	0	0	0	0	1	3275	42,5	101	70	79	102	78	44	8
1156	0	0	0	0	1	3370	42,5	107	72	79	101	61	35	6,2
1157	0	0	0	0	1	3275	42,5	126	73	85	102	63	36	6,5
1158	0	0	0	0	1	3370	42,5	107	68	78	85	75	43	6,4
1159	0	0	0	0	1	3190	50	132	84	95	97	112	61	10,9
1160	0	0	0	0	1	3190	50	118	74	84	103	87	48	9,1
1161	0	0	0	0	1	3195	50	119	81	93	115	55		6,4
1162	0	0	0	0	1	3465	52,5	114	76	86	111	67	40	7,4
1163	0	0	0	0	1	3465	52,5	117	78	88	114	69	40	7,9
1164	0	0	0	0	0	3380	52,5	123	71	84	107	72		7,7
1165	0	0	0	0	0	3510	52,5	116	78	88	104	60		6,3
1166	0	0	0	0	0	3555	55	108	70	80	80	86		6,9
1167	0	0	0	0	0	3145	55	123	83	93	86	71	46	6,1
1168	0	0	0	0	1	3250	57,5	125	83	95	75	105		7,9
1169	0	0	0	0	0	3720	57,5	130	87	98	95	80	44	7,5
1170	0	0	0	0	0	3720	57,5	141	90	103	92	80	45	7,3
1171	0	0	0	0	0	3610	60	125	82	92	107	81	45	8,6
1172	0	0	0	0	0	3280	60	111	72	82	85	87	50	7,5
1173	0	0	0	0	0	3610	60	121	84	94	100	66	38	6,6
1174	0	0	0	0	0	3280	60	109	70	79	88	70	40	6,2
1175	0	0	0	0	1	3585	62,5	118	69	81	97	89	48	8,6
1176	0	0	0	0	0	3880	62,5	115	68	78	101	95	50	9,7
1177	0	0	0	0	1	3305	62,5	115	78	89	88	71	44	6,3
1178	0	0	0	0	1	3305	62,5	110	70	82	84	81	50	6,8
1179	0	0	0	0	1	3585	62,5	91	66	72	100	78	43	7,9
1180	0	0	0	0	0	3485	65	116	80	89	91	84		7,5
1181	0	0		0	0	3685	67,5	124	77	87	93	95	50	8,8
1182	0	0		0	0	3685	67,5	129	86	96	89	88	48	7,9
1183	0	0	0	0	1	3390	70	107	76	85	99	72	42	7,1
1184	0	0	0	0	0	3300	70	110	74	84	95	75	40	7,1
1185	0	0	0	0	1	3410	70	114	76	85	97	74	44	7,2
1186	0	0	0	0	1	3390	70	127	75	87	113	60	36	6,8
1187	0	0	0	0	1	2725	70	117	76	87	112	102	51	11,5

1188	0	0	0	0	0	3300	70	112	75	84	115	56	31	6,5
1189	0	0	0	0	1	3675	70	93	58	66	96	56	37	5,4
1190	0	0	0	0	1	2725	70	125	80	87	91	121	62	11,1
1191	0	0	0	0	1	3410	70	114	76	85	97	74	44	7,2
1192	0	0	0	0	1	3410	72,5	123	84	94	92	100	49	9,2
1193	0	0	0	0	0	3875	72,5	108	72	80	85	95	49	8
1194	0	0	0	0	1	3410	72,5	130	71	80	108	90	44	9,7
1195	0	0	0	0	0	3875	72,5	107	72	82	78	94	50	7,3
1196	0	0	0	0	1	3410	72,5	107	66	79	94	104	51	9,7
1197	0	0	0	0	0	3760	75	101	67	77	85	80		6,8
1198	0	0	0	0	1	3460	75	115	77	86	80	74	44	6
1199	0	0	0	0	1	3460	75	131	78	90	88	76	45	6,7
1200	0	0	0	0	0	3950	77,5	123	80	89	94	73		6,9
1201	0	0	0	0	0	3650	77,5	141	90	103	100	96		9,6
1202	0	0	0	0	1	3930	82,5	133	72	86	105	91		9,5
1203	0	0	1	0	0	4030	82,5	132	90	100	142	55	29	7,8
1204	0	0	1	0	0	4030	82,5	124	84	93	131	47	25	6,3
1205	0	0	1	0	0	4030	82,5	121	77	84	131	49	28	6,5
1206	0	0	0	0	1	3845	82,5	104	64	72	83	103	57	8,6
1207	0	0	0	0	1	3845	82,5	109	69	78	93	84	48	7,8
1208	0	0	0	0	1	2870	85	108	77	86	114	64	39	7,2
1209	0	0	0	0	1	3865	85	114	64	75	104	50	35	5,2
1210	0	0	0	0	1	3765	85	118	78	87	114	70	36	7,9
1211	0	0	0	0	1	3865	85	103	73	83	104	52	37	5,3
1212	0	0	0	0	0	3260	87,5	119	83	92	113	64		7,3
1213	0	0	0	0	0	3915	90	110	76	86	107	81	43	8,7
1214	0	0	0	0	0	4195	90	128	74	87	114	72	40	8,2
1215	0	0	0	0	0	4180	90	100	66	75	104	82	43	8,5
1216	0	0	0	0	0	3915	90	121	71	81	100	66	36	6,6
1217	0	0	0	0	0	4215	90	110	68	78	102	56		5,7
1218	0	0	0	0	0	4180	90	111	71	80	103	74	39	7,6
1219	0	0	0	0	0	4005	90	112	80	87	82	86	44	7,1
1220	0	0	0	0	0	4195	90	121	71	82	111	85	47	9,4

1221	0	0	1	0	1	4405	92,5	122	70	81	108	96	44	10,3
1222	0	0	0	0	0	4265	92,5	116	67	77	116	81		9,4
1223	0	0	1	0	1	4405	92,5	116	78	87	108	70	33	7,6
1224	0	0	0	0	1	4065	92,5	121	74	85	108	54	27	5,9
1225	0	0	0	0	0	4085	92,5	119	81	91	106	86		9,1
1226	0	0	0	0	1	4065	92,5	143	84	95	97	84	43	8,2
1227	0	0	0	0	0	4110	95	124	72	84	107	64	38	6,8
1228	0	0	0	0	0	4475	97,5	116	69	78	79	109	57	8,7
1229	0	0	0	0	0	4475	97,5	123	73	83	82	92	49	7,5
1230	0	0	0	0	0	3430	45	98	65	75	125	38		4,8
1231	0	0	0	0	0	3795	77,5							
1232	0	0	0	0	0	3795	77,5	129	84	95	98	62	33	6,1
1233	0	0	0	0	0	3715	57,5	113	76	86	101	72		7,3
1234	0	0	0	0	1	2950	15	98	72	80	90	52		4,7
1235	0	0	2	0	0	3430	62,5	131	77	89	88	115	58	10,1
1236	0	0	2	0	0	3430	62,5	127	81	91	88	77	39	6,8
1237	0	0	1	0	1	3625	87,5	121	76	86	101	77	42	7,8
1238	0	0	1	0	1	3625	87,5	114	68	74	89	91	48	8,1
1239	0	0	0	0	1	3070	40	109	65	73	111	77	45	8,6
1240	0	0	0	0	1	3070	40	126	73	84	108	68	40	7,3
1241	0	0	0	0	1	3000	20	101	72	80	93	78		7,2
1242	0	0	0	0	0	3560	45	102	68	78	99	83	47	8,2
1243	0	0	0	0	0	3560	45	131	96	104	81	73	42	5,9
1244	0	0	0	0	0	3675	87,5	132	76	91	90	86		7,8
1245	0	0	0	0	1	3050	35	117	74	85	86	86		7,4
1246	0	0	0	0	0	3400	40	127	81	92	94	81	46	7,5
1247	0	0	0	0	0	3400	40	132	77	92	96	92	49	8,8
1248	0	0	0	0	0	3780	87,5	103	72	80	98	80		7,8
1249	0	0	0	0	0	2930	32,5	133	81	91	95	77	46	7,4
1250	0	0	1	0	0	2600	17,5	109	76	83	78	67	43	5,1
1251	0	0	1	0	0	2600	17,5	124	73	83	84	53	35	4,4
1252	0	0	0	0	0	3265	42,5	112	71	80	89	104	55	9,2
1253	0	0	0	0	0	3265	42,5	118	81	90	115	77	42	8,9

1254	0	0	0	0	0	3850	70	110	68	76	96	83	43	8
1255	0	0	0	0	0	3850	70	103	66	75	86	93	49	8
1256	0	0	0	0	0	3150	30	113	74	84	107	61		6,5
1257	0	0	0	0	0	3550	42,5	116	77	87	82	94	50	7,7
1258	0	0	0	0	0	3550	42,5	113	74	84	88	94	50	8,3
1259	0	0	0	0	1	4100	97,5	111	83	92	101	55		5,6
1260	0	0	0	0	0	3030	12,5	104	65	74	80	82	49	6,6
1261	0	0	0	0	0	3030	12,5	98	61	69	76	84	51	6,4
1262	0	0	0	0	0	3570	72,5	119	76	88	114	78		8,8
1263	0	0	0	0	0	3535	55	134	84	95	82	97		7,9
1264	0	0	0	0	1	2845	20	123	84	90	110	113	45	12,4
1265	0	0	0	0	1	2845	20	133	83	95	93	152	61	14,1
1266	0	0	0	0	0	3910	75	98	60	67	81	94	48	7,7
1267	0	0	0	0	1	3710	95	128	84	94	119	80	41	9,5
1268	0	0	0	0	1	3710	95	130	90	100	107	78	41	8,2
1269	0	0	0	0	1	4165	95	128	69	83	100	83		8,3
1270	0	0	0	0	0	3560	35	110	73	84	103	57		5,9
1271	0	0	0	0	0	3870	82,5	153	96	111	106	76		8
1272	0	0	0	0	0	3430	57,5	91	59	68	89	92	49	8,2
1273	0	0	0	0	0	3430	57,5	107	71	80	95	89	48	8,4
1274	0	0	0	0	0	3165	20	107	62	72	92	71	42	6,6
1275	0	0	0	0	0	3165	20	110	77	85	94	62	38	5,8
1276	0	0	2	0	0	4030	82,5	111	72	83	69	92		6,3
1277	0	0	0	0	1	3140	42,5	99	71	80	97	52	33	5,1
1278	0	0	0	0	1	3140	42,5	115	79	88	98	49	32	4,8
1279	0	0	0	0	0	3770	62,5	116	77	86	84	115	59	9,7
1280	0	0	0	0	0	3770	62,5	99	61	69	115	77	41	8,9
1281	0	0	0	0	0	3770	62,5	125	81	92	75	93	50	7
1282	0	0	1	0	0	4530	95	97	67	76	82	80	47	6,6
1283	0	0	1	0	0	4530	95	88	64	71	93	65	39	6
1284	0	0	0	0	1	3145	30	117	73	84	96	88		8,4
1285	0	0	0	0	0	3260	25	126	75	85	106	88	51	9,3
1286	0	0	0	0	0	3260	25	124	75	86	101	72	42	7,3

1287	0	0	0	0	0	3420	42,5	101	70	78	75	105	53	7,9
1288	0	0	0	0	0	3420	42,5	87	59	68	71	100	53	7,1
1289	0	0	0	0	1	3365	52,5	97	75	82	110	61	36	6,8
1290	0	0	0	0	1	3365	52,5	96	70	78	105	73	42	7,6
1291	0	0	0	0	0	3300	45	134	89	100	110	65	38	7,1
1292	0	0	0	0	0	3300	45	132	79	93	95	74	45	7
1293	0	0	0	0	0	3300	45	123	91	99	115	47	29	5,4
1294	0	0	0	0	0	3860	82,5	98	66	76	81	81	47	6,5
1295	0	0	0	0	0	3860	82,5	108	71	79	88	70	41	6,2
1296	0	0	0	0	0	3910	95	130	82	93	87	100		8,7
1297	0	0	0	0	0	2960	15	107	67	79	92	85		7,8
1298	0	0	0	0	1	2860	22,5	123	76	87	87	75	45	6,5
1299	0	0	0	0	1	2860	22,5	109	75	85	93	61	37	5,6
1300	0	0	0	0	0	3380	70	127	86	97	97	81		7,9
1301	0	0	0	0	0	3685	80	106	64	75	84	57		4,8
1302	0	0	0	0	0	3635	40	126	81	87	91	104	53	9,5
1303	0	0	0	0	0	3635	40	119	77	88	98	87	45	8,5
1304	0	0	0	0	0	3850	95	142	79	96	74	74	47	5,4
1305	0	0	0	0	0	3850	95	121	63	72	69	92	57	6,4
1306	0	0	0	0	0	3025	32,5	111	83	92	104	66	36	6,9
1307	0	0	0	0	0	3025	32,5	108	78	87	89	71	40	6,3
1308	0	0	0	0	0	3025	32,5	110	76	84	81	67	39	5,5
1309	0	0	0	0	0	3150	45	121	87	96	105	82	38	8,6
1310	0	0	0	0	0	3150	45	114	84	93	106	82	39	8,7
1311	0	0	0	0	1	3415	57,5	114	82	91	91	78		7,2
1312	0	0	0	0	0	3605	75	134	83	94	108	73	40	7,9
1313	0	0	0	0	0	3605	75	131	83	91	113	65	35	7,3
1314	0	0	0	0	0	3965	70	122	79	91	86	106	50	9,2
1315	0	0	0	0	0	3965	70	140	80	94	95	79	37	7,6
1316	0	0	0	0	0	3740	62,5	108	79	85	111	72	38	8
1317	0	0	0	0	0	3740	62,5	131	81	93	94	83	44	7,7
1318	0	0	0	0	1	3615	65	140	89	100	114	68	34	7,7
1319	0	0	0	0	1	3615	65	140	93	104	104	77	40	8

1320	0	0	0	0	0	4285	97,5	136	71	81	96	104	52	10
1321	0	0	0	0	0	4285	97,5	116	72	83	90	95	50	8,6
1322	0	0	0	0	1	3330	37,5	116	79	88	74	79	47	5,8
1323	0	0	0	0	0	3750	60	97	73	80	83	70	42	5,8
1324	0	0	0	0	0	3265	17,5	103	67	74	96	74	43	7,1
1325	0	0	0	0	0	3265	17,5	120	78	86	107	61	36	6,5
1326	0	0	0	0	1	3595	75	101	62	74	72	90	52	6,5
1327	0	0	0	0	1	3595	75	105	61	71	72	82	48	5,9
1328	0	0	0	0	0	3545	55	126	84	96	82	101		8,2
1329	0	0	0	0	0	3825	57,5	131	80	94	100	79		7,9
1330	0	0	0	0	0	3740	75	102	67	78	95	93		8,8
1331	0	0	0	0	1	3685	80	108	74	83	87	73		6,3
1332	0	0	0	0	0	3335	25	106	73	83	110	57	36	6,3
1333	0	0	0	0	0	3335	25	114	75	83	101	73	45	7,4
1334	0	0		0	0	3270	45	115	73	84	99	93		9,3
1335	0	0	0	0	0	3010	12,5	108	67	76	99	81	52	8
1336	0	0	0	0	0	3010	12,5	100	66	76	94	71	46	6,7
1337	0	0	0	0	0	3115	17,5	105	73	81	97	69	37	6,7
1338	0	0	0	0	0	3115	17,5	102	76	84	93	68	37	6,3
1339	0	0	0	0	0	4255	92,5	109	66	77	95	92	48	8,8
1340	0	0	0	0	0	4255	92,5	99	69	78	105	90	46	9,4
1341	0	0	0	0	0	3615	75	91	54	62	77	61	42	4,7
1342	0	0	0	0	0	3615	75	98	62	69	92	49	35	4,5
1343	0	0	0	0	0	3585	75	103	66	74	100	61	36	6,2
1344	0	0	0	0	0	3585	75	106	61	71	104	51	30	5,4
1345	0	0	0	0	1	3340	40	127	77	87	110	55	32	6,1
1346	0	0	0	0	1	3340	40	132	81	92	108	51	30	5,5
1347	0	0	1	0	0	3780	77,5	119	70	85	80	85	50	6,9
1348	0	0	1	0	0	3780	77,5	112	78	86	85	60	36	5,1
1349	0	0	1	0	1	3350	52,5	135	90	100	108	88	44	9,5
1350	0	0	0	0	1	3285	45	123	86	96	105	72	43	7,5
1351	0	0	0	0	1	3285	45	121	80	90	97	95	55	9,2
1352	0	0	0	0	0	4090	85	110	79	88	93	57	35	5,3

1353	0	0	0	0	0	4090	85	134	83	93	94	68	39	6,5
1354	0	0	0	0	0	4090	85	118	77	87	92	68	41	6,3
1355	0	0	0	0	0	3585	60	119	80	89	95	83	47	7,9
1356	0	0	0	0	0	3585	60	120	77	87	95	80	46	7,6
1357	0	0	0	0	0	3585	60	125	82	94	88	71	43	6,3
1358	0	0	0	0	1	3175	22,5	122	78	89	83	85	44	7,1
1359	0	0	0	0	1	3175	22,5	103	73	81	91	86	45	7,8
1360	0	0	0	0	0	4195	90	115	68	77	97	67	37	6,5
1361	0	0	0	0	0	4195	90	115	77	87	91	89	47	8,1
1362	0	0	0	0	1	3580	72,5	115	75	85	85	79		6,7
1363	0	0	0	0	0	3390	27,5	107	75	84	106	58	36	6,1
1364	0	0	0	0	0	3390	27,5	111	71	82	99	70	43	6,9
1365	0	0	0	0	0	2880	27,5	101	67	76	101	67	40	6,7
1366	0	0	0	0	0	2880	27,5	106	61	71	91	66	40	6
1367	0	0	0	0	0	3875	82,5	119	71	81	97	87	45	8,4
1368	0	0	0	0	0	3875	82,5	113	73	82	82	108	55	9
1369	0	0	0	0	0	3655	52,5	132	80	90	112	78	44	8,8
1370	0	0	0	0	0	3655	52,5	140	90	101	119	59	34	7,1
1371	0	0	0	0	0	3270	30	130	86	97	92	82		7,5
1372	0	0	0	0	0	3255	17,5	109	77	86	92	75	46	6,9
1373	0	0	0	0	0	3255	17,5	121	81	91	83	89	53	7,4
1374	0	0	2	0	1	3940	87,5	97	69	77	81	85	47	6,9
1375	0	0	2	0	1	3940	87,5	107	69	78	89	99	53	8,8
1376	0	0	0	0	1	3120	17,5	115	67	78	92	81	47	7,5
1377	0	0	0	0	1	3120	17,5	116	70	80	89	74	43	6,6
1378	0	0	0	0	1	3270	60	102	70	79	120	61	39	7,3
1379	0	0	0	0	1	3270	60	124	65	85	105	60	38	6,3
1380	0	0	0	0	0	4785	97,5	124	80	90	105	75	42	7,9
1381	0	0	0	0	0	3235	25	97	70	78	92	74		6,8
1382	0	0	3	0	0	3175	67,5	105	71	80	98	72	41	7,1
1383	0	0	3	0	0	3175	67,5	104	67	74	80	84	49	6,8
1384	0	0	0	0	0	3805	77,5	110	72	81	100	74	41	7,5
1385	0	0	0	0	0	3805	77,5	123	74	84	115	75	41	8,7

1386	0	0	0	0	1	3536	57,5	115	78	88	102	110	60	11,2
1387	0	0	0	0	1	3536	57,5	123	80	90	101	97	55	9,8
1388	0	0	0	0	1	3536	57,5	115	79	88	100	75	43	7,5
1389	0	0	0	0	0	3340	65	112	70	80	97	70	36	6,9
1390	0	0	0	0	0	3340	65	122	76	86	96	83	42	7,9
1391	0	0	0	0	0	3075	22,5	150	95	109	110	65	33	7,1
1392	0	0	0	0	0	3075	22,5	159	95	112	92	73	37	6,7
1393	0	0	0	0	0	3390	27,5	111	67	80	95	79	43	7,5
1394	0	0	0	0	0	3390	27,5	106	70	80	83	86	49	7,2
1395	0	0	0	0	0	3710	82,5	130	87	98	117	86		10
1396	0	0	0	0	1	4005	95	117	78	87	104	72	39	7,5
1397	0	0	0	0	1	4005	95	135	87	100	115	84	44	9,6
1398	0	0	0	0	1	4005	95	128	61	76	102	68	37	6,9
1399	0	0	0	0	0	3240	42,5	117	70	80	106	63	37	6,7
1400	0	0	0	0	0	3240	42,5	101	68	77	95	69	42	6,5
1401	0	0	0	0	1	2995	17,5	128	79	92	103	60		6,2
1402	0	0	0	0	1	2900	42,5	101	70	80	99	62	37	6,1
1403	0	0	0	0	1	2900	42,5	105	68	78	99	65	39	6,4
1404	0	0	0	0	0	3125	27,5	105	71	81	78	80		6,2
1405	0	0	0	0	1	3570	90	105	72	82	98	67		6,6
1406	0	0	0	0	0	3325	32,5	113	79	88	98	72		7
1407	0	0	0	0	1	3095	37,5	117	78	88	119	75	40	8,9
1408	0	0	0	0	1	3095	37,5	124	81	91	104	87	47	9
1409	0	0	0	0	0	3600	60	119	84	94	82	87	51	7,1
1410	0	0	0	0	0	3600	60	111	70	81	76	98	56	7,5
1411	0	0	0	0	1	3020	82,5	107	77	86	106	50		5,4
1412	0	0	0	0	0	3540	72,5	93	65	73	92	94	55	8,6
1413	0	0	0	0	0	3540	72,5	94	66	74	101	76	46	7,6
1414	0	0	0	0	0	3345	72,5	110	74	83	100	64	38	6,4
1415	0	0	0	0	0	3345	72,5	106	72	82	93	77	44	7,2
1416	0	0	0	0	0	4315	97,5	130	88	99	87	81	45	7
1417	0	0	0	0	0	3195	57,5	108	75	85	102	54	32	5,5
1418	0	0	0	0	0	3195	57,5	110	74	84	91	76	45	6,9

1419	0	0	0	0	1	3120	72,5	134	85	97	116	74		8,6
1420	1	0	0	0	0	2490	65	110	71	81	71	92	55	6,5
1421	1	0	0	0	0	2580	52,5	127	90	101	87	85		7,4
1422	1	0	0	0	0	2585	32,5	128	90	101	97	76	47	7,4
1423	1	0	0	0	0	2585	32,5	124	81	91	92	77	47	7,1
1424	1	0	0	0	1	2090	45	102	75	84	114	60	36	6,9
1425	1	0	0	0	1	2090	45	111	71	81	96	82	48	7,9
1426	1	0	0	0	1	2835	55	103	79	87	112	45	28	5,1
1427	1	0	0	0	1	2835	55	128	95	105	128	39	25	5
1428	1	0	0	0	0	2765	50	111	72	81	95	83	44	7,8
1429	1	0	0	0	0	2765	50	123	74	85	95	77	42	7,3
1430	1	0	0	0	1	2175	50	118	80	90	90	76	47	6,7
1431	1	0	0	0	1	2175	50	121	72	83	98	80	49	7,7
1432	1	0	3	0	1	2880	85	117	80	90	106	73	37	7,7
1433	1	0	3	0	1	2880	85	109	78	87	99	78	40	7,7
1434	1	0	0	0	1	2435	30	105	77	85	99	89	49	8,8
1435	1	0	0	0	1	2435	30	111	74	84	82	107	58	8,9
1436	1	0	0	0	1	3030	85	121	74	86	110	68	37	7,5
1437	1	0	0	0	1	3030	85	131	81	93	114	70	38	8
1438	1	0	0	0	1	3030	85	124	73	81	119	64	35	7,6
1439	1	0	2	0	0	3230	90	124	77	88	93	97	57	9
1440	1	0	2	0	0	3230	90	107	76	85	95	91	52	8,6
1441	1	0	0	0	1	2625	45	109	73	82	88	80	49	7
1442	1	0	0	0	1	2625	45	117	72	82	77	103	61	7,9
1443	1	0	0	0	0	2725	37,5	103	69	77	79	83	49	6,6
1444	1	0	0	0	0	2725	37,5	102	75	83	79	80	47	6,3
1445	1	0	2	0	1	3020	82,5	117	73	84	91	105	52	9,5
1446	1	0	2	0	1	3020	82,5	122	69	82	78	111	56	8,6
1447	1	0	0	0	1	2915	75	105	73	80	102	62	39	6,3
1448	1	0	0	0	1	2915	75	119	66	74	113	56	35	6,3
1449	1	0	0	0	1	2975	77,5	127	73	83	79	108	57	8,5
1450	1	0	0	0	1	2975	77,5	130	82	91	94	92	49	8,6
1451	1	0	1	0	0	3070	85	110	73	83	121	57	37	6,8

1452	1	0	1	0	0	3070	85	122	83	92	114	73	46	8,3
1453	0	1	0	0	0	2345	77,5	107	71	81	86	77		6,6
1454	0	1	0	0	1	1850	100	128	88	99	95	94	48	8,9
1455	0	1	0	0	0	1836	17,5	121	84	95	100	87	46	8,7
1456	0	1	0	0	0	1836	17,5	116	77	87	139	56	30	7,8
1457	0	1	0	0	0	1836	17,5	127	80	91	97	106	56	10,3
1458	0	1	0	0	1	3200	52,5	135	81	92	114	72	46	8,1
1459	0	1	0	0	1	3200	52,5	126	84	95	100	58	38	5,8
1460	0	1	0	0	1	1744	80	111	77	87	101	62	37	6,3
1461	0	1	0	0	1	1744	80	106	75	84	92	66	39	6,1
1462	0	1	0	0	0	2010	57,5	107	68	78	104	76	48	7,9
1463	0	1	1	0	0	2375	82,5	108	74	83	90	72	41	6,5
1464	0	1	1	0	0	2375	82,5	114	71	81	100	72	41	7,3
1465	0	1	0	0	0	2935	82,5	121	76	87	84	72	43	6
1466	0	1	0	0	0	2935	82,5	101	66	74	92	66	39	6
1467	0	1	0	0	1	1875	57,5	114	74	85	78	94	53	7,3
1468	0	1	0	0	1	1875	57,5	105	69	79	81	88	51	7,1
1469	0	1	0	0	1	1875	57,5	123	69	86	103	67	39	7
1470	0	0		0	1	927	12,5	147	85	95	91	70		6,3
1471	0	0		0	0	2835	17,5	142	102	112	99	54		5,3
1472	0	0		0	0	3830	80	134	94	105	95	77		7,3
1473	0	0		0		3595	50	122	84	95	73	103		7,6
1474	0	0			1	2775	15	116	80	89	105	71	40	7,5
1475	0	0			0	3650	52,5	124	75	87	100	105	56	10,5
1476	0	0			0	3650	52,5	124	74	86	105	98	53	10,3
1477	0	0			0	4840	100	117	73	84	92	96	51	8,8
1478	0	0			0	3235	25	103	69	78	91	85	51	7,7
1479	0	0			1	2840	25	124	84	96	87	97	52	8,4
1480	0	0			0	3235	25	105	72	79	91	73	46	6,6
1481	0	0			1	3245	57,5	109	70	81	98	72	42	7,1
1482	0	0			1	3245	57,5	103	73	81	89	71	42	6,4
1483	0	0			1	3295	62,5	108	74	83	94	69	40	6,5
1484	0	0			1	3295	62,5	106	75	84	98	71	41	7

1485	0	0			0	3305	50	124	81	92	100	61	35	6,1
1486	0	0			0	3505	52,5	101	64	73	105	69	44	7,2
1487	0	0			0	3505	52,5	106	63	74	103	75	47	7,8
1488	0	0			1	3155	32,5	92	62	70	103	77	50	8
1489	0	0			1	3740	67,5	118	81	91	108	75	39	8,1
1490	0	0			0	3640	52,5	134	67	86	96	61	38	5,9
1491	0	0			0	3640	52,5	115	80	90	94	61	37	5,7
1492	0	0			0	3015	12,5	136	76	91	84	98	55	8,3
1493	0	0			0	3150	12,5	121	74	84	93	66	40	6,1
1494	0	0			0	3150	12,5	121	76	87	79	74	45	5,8
1495	0	0			0	3015	12,5	111	76	86	83	103	58	8,4
1496	0	0			1	2735	12,5	109	61	72	89	76	45	6,8
1497	0	0			1	2735	12,5	123	73	83	97	84	48	8,1
1498	0	0			1	2880	12,5	111	74	84	87	74	45	6,4
1499	0	0			1	2880	12,5	126	76	88	95	86	50	8,1
1500	0	0			0	2805	15	106	68	78	112	77	43	8,7
1501	0	0			1	2775	15	124	82	92	88	90	50	7,9
1502	0	0			0	2805	15	116	80	89	116	69	38	8
1503	0	0			1	2755	17,5	107	73	82	105	102	53	10,7
1504	0	0			1	2975	17,5	114	81	90	95	58	37	5,5
1505	0	0			1	2755	17,5	99	69	79	111	79	41	8,7
1506	0	0			0	2955	17,5	103	70	79	89	73	45	6,5
1507	0	0			0	2955	17,5	92	62	70	82	84	51	6,9
1508	0	0			1	3165	20	113	72	82	103	68	40	7
1509	0	0			1	3045	20	118	79	90	100	83	45	8,3
1510	0	0			1	3035	20	123	85	95	87	88	49	7,6
1511	0	0			1	3045	20	126	78	90	90	83	46	7,5
1512	0	0			1	3165	20	109	75	85	132	60	35	7,8
1513	0	0			1	2790	22,5	113	75	85	90	91	47	8,2
1514	0	0			0	3220	22,5	128	82	91	86	77	45	6,6
1515	0	0			0	3220	22,5	131	79	91	81	85	50	6,9
1516	0	0			0	2890	22,5	118	73	83	87	91	51	8
1517	0	0			1	2790	22,5	123	68	80	83	104	52	8,6

1518	0	0			0	2890	22,5	118	73	85	84	97	54	8,2
1519	0	0			1	3205	22,5	125	86	96	108	71	38	7,7
1520	0	0			0	3360	25	114	74	84	85	71	42	6
1521	0	0			0	3360	25	109	76	84	88	82	47	7,2
1522	0	0			0	3260	27,5	115	76	86	89	77	39	6,8
1523	0	0			1	2535	27,5	105	73	82	106	74	42	7,8
1524	0	0			1	2535	27,5	99	69	77	89	70	41	6,2
1525	0	0			0	3415	32,5	108	73	83	94	61	39	5,7
1526	0	0			0	3025	32,5	108	76	86	122	56	33	6,8
1527	0	0			0	3415	32,5	106	64	77	103	84	52	8,7
1528	0	0			0	3025	32,5	123	75	86	99	69	42	6,9
1529	0	0			1	3270	32,5	118	73	80	91	76	45	6,9
1530	0	0			1	3270	32,5							
1531	0	0			1	3155	32,5	113	63	73	92	89	57	8,1
1532	0	0			1	3200	35	126	76	88	95	86	50	8,1
1533	0	0			1	3200	35	106	69	80	87	89	52	7,7
1534	0	0			1	3095	37,5	118	70	84	97	70	42	6,8
1535	0	0			1	3095	37,5	131	75	88	97	81	48	7,8
1536	0	0			0	3395	40	139	82	97	125	76	37	9,5
1537	0	0			0	3210	40	116	78	88	116	64	35	7,4
1538	0	0			0	3210	40	114	79	89	112	76	43	8,6
1539	0	0			0	3230	40	117	82	92	103	60	36	6,2
1540	0	0			1	3340	40	127	73	89	106	95	48	10,1
1541	0	0			1	3340	40	131	72	81	89	110	55	9,7
1542	0	0			0	3230	40	132	89	101	96	54	33	5,2
1543	0	0			0	3395	40	125	85	96	122	79	39	9,7
1544	0	0			0	3275	42,5	116	68	81	103	78	48	7,9
1545	0	0			1	3255	42,5	130	79	90	112	61	37	6,8
1546	0	0			0	3275	42,5	111	74	85	81	89	56	7,2
1547	0	0			1	3440	45	106	74	83	93	71	44	6,6
1548	0	0			1	3440	45	115	80	87	105	67	43	7
1549	0	0			0	3440	45	116	84	93	99	74	41	7,3
1550	0	0			0	3125	45	106	71	80	101	61	35	6,1

1551	0	0			0	3125	45	124	78	90	98	63	37	6,1
1552	0	0			1	3180	47,5	123	79	90	125	58	33	7,2
1553	0	0			1	3180	47,5	117	76	86	97	82	45	8
1554	0	0			1	3460	47,5	138	87	98	102	79	44	8
1555	0	0			1	3460	47,5	118	80	90	114	79	43	8,9
1556	0	0			0	3600	47,5	115	79	89	107	64	37	6,8
1557	0	0			1	2655	47,5	122	71	85	76	97	56	7,4
1558	0	0			1	2655	47,5	133	79	95	92	74	43	6,8
1559	0	0			0	3600	47,5	114	72	83	108	73	41	7,9
1560	0	0			0	3305	50	121	83	94	107	62	34	6,6
1561	0	0			0	3495	50	124	90	100	103	60	38	6,1
1562	0	0			1	3165	50	124	84	95	91	79	44	7,2
1563	0	0			1	3165	50	113	76	86	98	58	34	5,7
1564	0	0			0	3495	50	118	84	94	112	57	36	6,4
1565	0	0			0	3490	50	111	77	86	90	71	39	6,4
1566	0	0			0	3490	50	111	77	86	90	71	39	6,4
1567	0	0			0	3485	50	107	76	85	93	67	35	6,2
1568	0	0			0	3485	50	98	71	79	88	62	34	5,5
1569	0	0			0	3320	50	100	68	77	88	57	32	5
1570	0	0			1	3230	52,5	119	66	75	139	53	31	7,4
1571	0	0			0	3635	52,5	108	75	84	97	74	42	7,1
1572	0	0			1	2985	52,5	109	76	86	106	64	38	6,8
1573	0	0			0	3515	52,5	124	80	91	113	72	41	8,1
1574	0	0			1	3230	52,5	108	56	66	122	68	39	8,3
1575	0	0			1	2985	52,5	135	95	106	98	60	36	5,9
1576	0	0			0	3635	52,5	121	74	84	103	75	41	7,6
1577	0	0			0	3515	52,5	116	74	86	98	91	53	8,9
1578	0	0			1	3405	55	128	73	81	90	88	42	7,9
1579	0	0			1	3405	55	121	77	88	82	95	46	7,6
1580	0	0			1	2590	57,5	123	84	93	107	89	43	9,5
1581	0	0			1	2590	57,5	115	73	84	93	106	50	9,8
1582	0	0			0	3725	57,5	111	78	88	105	66	35	6,9
1583	0	0			1	3400	57,5	106	75	84	96	69	40	6,6

1584	0	0			1	3400	57,5	108	78	86	96	72	43	6,9
1585	0	0			0	3205	57,5	87	66	71	76	81	49	6,1
1586	0	0			0	3725	57,5	119	77	89	91	76	42	6,9
1587	0	0			0	3205	57,5	90	60	69	75	68	43	5,1
1588	0	0			0	3605	60	117	77	87	108	82	50	8,8
1589	0	0			0	3605	60	124	71	83	112	61	37	6,8
1590	0	0			1	3280	60	125	70	86	69	95	53	6,6
1591	0	0			1	3280	60	109	66	76	70	111	60	7,7
1592	0	0			0	3460	62,5	108	76	86	102	83	46	8,4
1593	0	0			0	3460	62,5	114	79	89	97	91	49	8,8
1594	0	0			0	3775	62,5	108	69	79	85	71	46	6
1595	0	0			0	3775	62,5	99	68	77	96	84	53	8,1
1596	0	0			0	3460	65	100	68	77	100	58	33	5,8
1597	0	0			1	3740	67,5	116	80	90	117	72	37	8,5
1598	0	0			0	3410	70	114	74	85	104	58	39	6,1
1599	0	0			0	3690	70	102	73	81	119	63	38	7,4
1600	0	0			0	3690	70	102	69	79	110	67	40	7,4
1601	0	0			1	3380	70	117	72	86	90	95	53	8,6
1602	0	0			1	3380	70	115	81	90	94	66	39	6,2
1603	0	0			0	3410	70	108	77	86	94	71	46	6,7
1604	0	0			1	3560	72,5	142	93	106	98	91	40	8,9
1605	0	0			0	3735	72,5	141	91	102	93	68	35	6,3
1606	0	0			0	2870	72,5	117	81	89	105	74	38	7,8
1607	0	0			1	3420	72,5	132	82	96	120	99	47	11,9
1608	0	0			0	3735	72,5	126	82	95	94	91	46	8,6
1609	0	0			1	3695	72,5	103	69	78	92	60	36	5,6
1610	0	0			1	3585	72,5	121	90	99	102	73	41	7,4
1611	0	0			1	3585	72,5	123	85	95	102	73	41	7,4
1612	0	0			1	3410	72,5	100	69	78	85	85	50	7,2
1613	0	0			0	2870	72,5	123	81	90	99	71	36	7
1614	0	0			1	3695	72,5	105	71	81	83	60	36	4,9
1615	0	0			1	3420	72,5	131	86	96	113	89	42	10,1
1616	0	0			1	3410	72,5	106	70	80	91	75	45	6,8

1617	0	0			0	3610	75	144	75	87	107	65	40	7
1618	0	0			0	3610	75	130	79	89	103	78	47	8
1619	0	0			0	3935	75	122	84	94	95	71	39	6,7
1620	0	0			0	3935	75	109	79	88	106	56	32	5,9
1621	0	0			0	3600	75	133	85	97	80	89	46	7,1
1622	0	0			0	3600	75	126	81	92	80	99	51	7,9
1623	0	0			1	3885	77,5	117	70	79	100	74	40	7,4
1624	0	0			0	3495	77,5	123	78	89	95	68	42	6,4
1625	0	0			1	3885	77,5	118	68	79	102	82	44	8,4
1626	0	0			1	3670	80	101	69	78	92	82	46	7,5
1627	0	0			1	3705	80	111	75	85	92	69	42	6,3
1628	0	0			1	3670	80	109	75	84	100	78	44	7,7
1629	0	0			1	3560	82,5	135	88	99	97	75	46	7,2
1630	0	0			1	3560	82,5	116	78	88	99	72	44	7,1
1631	0	0			0	3890	82,5	105	76	85	102	66	36	6,7
1632	0	0			1	3860	82,5	96	67	76	101	79	43	7,9
1633	0	0			1	3860	82,5	100	74	82	110	68	37	7,5
1634	0	0			1	3230	82,5	108	68	79	87	74	44	6,4
1635	0	0			0	3890	82,5	123	76	88	86	96	50	8,2
1636	0	0			1	3230	82,5	107	70	80	91	65	40	5,9
1637	0	0			0	3985	87,5	109	75	84	98	64	41	6,3
1638	0	0			1	3940	87,5	121	73	86	95	94	54	8,9
1639	0	0			1	3940	87,5	141	65	76	106	90	50	9,6
1640	0	0			0	4015	90	122	82	91	134	59	36	7,9
1641	0	0			1	4050	90	103	66	76	92	79	46	7,2
1642	0	0			0	4015	90	115	75	84	126	65	39	8,2
1643	0	0			1	3890	92,5	106	74	83	98	80	45	7,9
1644	0	0			0	4100	92,5	116	78	88	133	65	33	8,6
1645	0	0			0	4100	92,5	117	80	90	129	59	30	7,6
1646	0	0			1	3890	92,5	132	61	79	90	82	47	7,4
1647	0	0			1	4100	95	92	65	73	87	91	51	8
1648	0	0			1	4100	95	97	65	74	79	84	50	6,6
1649	0	0			0	4195	95	142	87	98	114	71	43	8

1650	0	0			0	4195	95	128	87	98	86	90	57	7,7
1651	0	0			0	4040	95	109	74	83	98	70	38	6,9
1652	0	0			0	4040	95	110	74	84	102	77	42	7,8
1653	0	0			0	4235	97,5	118	79	89	93	90	41	8,3
1654	0	0				4295	97,5	105	70	78	121	66	36	7,9
1655	0	0			1	4260	97,5	133	84	96	93	101	54	9,4
1656	0	0			1	4345	97,5	125	72	82	119	73	39	8,7
1657	0	0				4295	97,5	97	65	74	99	66	37	6,6
1658	0	0			1	4260	97,5	142	95	105	108	65	35	7
1659	0	0			1	4345	97,5	130	78	93	102	88	47	8,9
1660	0	0			0	4235	97,5	124	81	93	105	74	34	7,8
1661	0	0			1	4105	97,5	127	81	91	99	74	39	7,3
1662	0	0			1	4905	100	101	72	80	91	83	44	7,6
1663	0	0			1	4905	100	107	69	79	107	85	44	9
1664	0	0			1	3440	60	119	73	82	103	109	53	11,2
1665	0	0			1	3440	60	110	65	76	105	95	46	9,9
1666	0	0			0	3265	17,5	121	77	88	89	106	54	9,6
1667	0	0			0	3265	17,5	133	81	92	79	81	43	6,4
1668	0	0			0	3830	67,5	122	81	92	75	79	43	5,9
1669	0	0			0	3830	67,5	108	72	82	72	92	50	6,6
1670	0	0			1	3105	40	105	62	72	77	74	43	5,7
1671	0	0			1	3105	40	99	60	70	65	88	50	5,7
1672	0	0			1	3535	82,5	109	71	83	129	48	30	6,3
1673	0	0			1	3535	82,5	110	68	78	97	61	38	5,9
1674	0	0			0	3370	52,5	143	72	88	102	89	45	9,2
1675	0	0			0	3370	52,5	132	79	90	118	86	42	10,1
1676	0	0			1	3445	60	113	76	86	115	68	38	7,8
1677	0	0			0	3590	60	109	80	89	93	85	48	7,9
1678	0	0			0	3590	60	97	70	78	91	98	55	8,9
1679	0	0			0	3840	80	125	80	88	91	94	47	8,6
1680	0	0			0	3840	80	119	78	88	103	86	43	8,8
1681	0	0			0	2920	15	111	77	87	96	65	44	6,3
1682	0	0			0	2920	15	114	76	85	101	56	38	5,6

1683	0	0			1	3620	65	93	66	74	98	81	48	7,9
1684	0	0			1	3620	65	102	69	79	106	74	45	7,9
1685	0	0			0	3190	37,5	117	74	85	100	82	46	8,2
1686	0	0			0	3190	37,5	107	73	83	98	92	50	9
1687	0	0			1	3275	20	99	64	74	85	92	56	7,8
1688	0	0			1	3275	20	101	66	76	81	81	50	6,6
1689	0	0			1	3580	72,5	125	91	101	107	97	47	10,4
1690	0	0			1	3580	72,5	124	74	87	111	82	40	9,2
1691	0	0			1	4040	90	118	70	82	90	82	47	7,4
1692	0	0			1	4040	90	105	73	79	113	77	44	8,7
1693	0	0			1	2880	12,5	144	85	101	114	95	52	10,8
1694	0	0			1	2880	12,5	127	81	94	102	84	46	8,5
1695	0	0			1	3605	65	122	68	79	98	87	48	8,5
1696	0	0			1	3605	65	93	61	70	80	100	57	8
1697	0	0			0	3635	90	110	71	82	116	68	34	7,9
1698	0	0			1	3140	30	131	95	106	118	63	37	7,4
1699	0	0			1	3140	30	148	89	98	101	64	38	6,5
1700	0	0			1	1140	0	135	89	101	77	63	35	4,8
1701	0	0			1	380	0	138	97	108	98	67	30	6,6
1702	0	0			1	1302	2,5	149	105	115	110	55	30	6,1
1703	0	0			0	524	2,5	139	85	97	86	80	50	7
1704	0	0			1	1092	5	164	95	110	72	128	57	9,3
1705	0	0			1	440	7,5	149	87	102	95	91	46	8,6
1706	0	0			1	660	10	118	84	94	70	84	48	5,9
1707	0	0			1	770	10	150	97	108	115	57	29	6,5
1708	0	0			0	1078	17,5	149	93	105	79	66	34	5,2
1709	0	0			1	1335	17,5	136	109	114	98	76	39	7,4
1710	0	0			1	1410	17,5	139	101	109	106	69	42	7,3
1711	0	0			0	1770	20	137	87	97	114	64	33	7,3
1712	0	0			0	1057	20	168	109	121	93	73	37	6,8
1713	0	0			0	856	22,5	121	90	99	105	84	44	8,9
1714	0	0			0	1333	27,5	139	93	104	76	65	32	5
1715	0	0			0	923	30	177	101	122	58	114	55	6,6

1716	0	0			1	905	32,5	153	110	114	106	79	37	8,3
1717	0	0			1	1435	32,5	150	100	112	86	63	36	5,4
1718	0	0			0	1485	37,5	159	93	108	65	141	65	9,2
1719	0	0			1	1780	47,5	169	108	124	88	67	36	5,9
1720	0	0			0	1652	57,5	164	98	117	99	61	31	6,1
1721	0	0			0	2127	60	141	98	109	97	88	45	8,6
1722	0	0			1	1542	75	136	85	95	106	85	47	9
1723	0	0			1	1560	90	132	85	98	116	70	43	8,1
1724	0	0			1	741	25	155	94	110	101	94	46	9,6
1725	0	0			1	1783	30	163	110	123	90	38	23	3,4
1726	0	0			1	1800	50	136	85	97	80	111	59	8,9
1727	0	0			1	1370	30	119	85	94	84	45	30	3,8
1728	0	0			0	2185	27,5	143	105	116	116	55	26	6,3
1729	0	0			0	923	12,5	166	107	117	74	76	38	5,6
1730	0	0			1	1130	2,5	132	92	102	106	49	27	5,2
1731	0	0			1	510	2,5	141	98	110	94	72	41	6,7
1732	0	0			0	1460	2,5	138	97	108	109	65	35	7,1
1733	0	0			0	1460	2,5	124	90	100	96	67	36	6,4
1734	0	0			1	1670	15	164	101	119	85	55	34	4,7
1735	0	0			0	992	45	110	77	87	84	98		8,2
1736	0	0			1	1125	17,5	144	77	91	75	68	38	5
1737	0	0			1	1580	10	117	83	93	94	91	45	8,6
1738	0	0			1	1580	10	122	81	91	116	73	38	8,4
1739	0	0			1	1580	10	109	76	86	107	83	43	8,9
1740	0	0			1	1845	52,5	135	90	100	87	108	51	9,3
1741	0	0			1	1845	52,5	156	101	110	79	101	49	7,9
1742	0	0			1	1845	52,5	152	100	110	75	94	47	7
1743	0	0			0	2900	97,5	123	83	92	80	86	47	6,9
1744	0	0			0	2764	95	141	97	107	147	109	40	16,1
1745	0	0			1	680	12,5	159	101	112	59	110	58	6,5
1746	0	0			0	500	0	115	83	92	116	60	33	6,9
1747	0	0			0	500	0	163	121	133	95	68	35	6,4
1748	0	0			1	1914	57,5	153	102	115	106	63	30	6,7

1749	0	0			1	994	32,5	172	113	128	90	63	37	5,7
1750	0	0			1	1280	7,5	171	97	107	87	63	35	5,5
1751	0	0		2	1	1300	2,5	149	82	102	79	90	50	7,1
1752	0	0		2	1	1300	2,5	157	113	124	82	74	42	6,1
1753	0	0	0	1	0	1477	7,5	110	71	83	87	70		6,1
1754	0	0	0	0	1	595	5	180	115	132	102	53	30	5,4
1755	0	0	0	0	1	595	5	141	104	115	102	61	36	6,2
1756	0	0		0	1	1850	95	188	129	143	110	59	31	6,5
1757	0	0		0	1	1850	95	174	114	129	135	51	26	7
1758	0	0		0	0	1477	2,5	155	102	114	96	62		5,9
1759	0	0		0	0	600	2,5	172	105	122	102	71		7,2
1760	0	0		0	1	440	2,5	141	100	111	88	72		6,3
1761	0	0		0	1	1333	5	132	90	101	80	83		6,6
1762	0	0		0	1	1374	5	158	103	117	77	105		8,1
1763	0	0		0	0	1295	5	152	96	112	93	67		6,2
1764	0	0		0	0	630	7,5	146	108	116	98	109		10,7
1765	0	0		0	0	1425	10	168	110	122	107	90		9,5
1766	0	0		0	1	1465	10	157	108	121	96	64		6,1
1767	0	0		0	1	1185	10	131	88	99	64	110		7,1
1768	0	0		0	0	1175	12,5	153	104	117	94	75		7
1769	0	0		0	1	1610	25	148	100	112	99	65		6,4
1770	0	0		0	0	1930	30	148	102	113	114	81		9,2
1771	0	0		0	0	1920	32,5	134	98	108	87	86		7,5
1772	0	0		0	1	747	35	185	104	127	78	67		5,2
1773	0	0		0	1	1603	37,5	164	101	118	79	136		10,8
1774	0	0		0	0	992	45	146	92	105	67	99		6,7
1775	0	0		0	1	843	57,5	173	110	126	106	68		7,2
1776	0	0		0	1	1334	62,5	139	85	99	90	76		6,9
1777	0	0		0	1	2010	65	166	108	122	99	63		6,2
1778	0	0		0	1	1150	87,5	172	118	130	77	67		5,2
1779	0	0		0		320	0	180	114	126	92	80		7,3
1780	0	0		0	0	1920	30	159	97	113	113	71		8,1
1781	0	0		0	0	1730	30	177	121	136	104	75		7,8

1782	0	0		0	1	1360	22,5	173	104	119	101	90		9
1783	0	0		1	1	720	22,5	166	103	118	87	91		7,9
1784	0	0		1	1	1439	50	142	96	109	93	121		11,1
1785	0	0			1	550	7,5	115	67	76	86	93	49	7,9
1786	0	0			1	550	7,5	122	76	88	95	86	45	8,1
1787	0	0			0	1480	2,5	124	88	94	87	81	46	7,1
1788	0	0			0	2240	2,5	167	125	136	99	64	33	6,4
1789	0	0			1	1489	2,5	135	98	108	85	62	35	5,2
1790	0	0			0	1760	2,5	145	98	110	84	78	43	6,5
1791	0	0			1	2145	2,5	141	98	109	82	89	44	7,3
1792	0	0			1	2505	2,5	135	99	110	69	75	43	5,2
1793	0	0			1	2750	5	155	100	113	81	77	40	6,3
1794	0	0			0	2355	5	149	96	112	87	77	41	6,7
1795	0	0			1	1760	5	135	96	107	92	74	40	6,8
1796	0	0			1	2770	5	184	115	131	78	116	54	9,1
1797	0	0			1	2790	7,5	117	85	94	101	61	35	6,2
1798	0	0			0	2355	7,5	132	96	105	93	54	33	5
1799	0	0			0	2235	10	163	108	123	88	52	33	4,6
1800	0	0			1	2920	12,5	142	90	98	81	81	47	6,5
1801	0	0			0	2730	12,5	147	92	105	90	107	54	9,6
1802	0	0			1	2930	15	127	92	101	98	58	34	5,7
1803	0	0			1	2740	17,5	130	90	100	92	88	45	8,1
1804	0	0			1	2915	20	165	108	122	78	91	49	7
1805	0	0			1	2350	20	128	89	100	93	76	44	7
1806	0	0			0	2865	20	155	102	113	70	86	44	6,1
1807	0	0			0	2640	20	158	98	112	90	99	48	8,9
1808	0	0			1	2800	22,5	167	108	122	93	73	41	6,8
1809	0	0			1	2320	32,5	130	93	101	68	131	74	9
1810	0	0			0	3370	37,5	136	98	109	91	70	40	6,4
1811	0	0			0	3250	42,5	139	103	113	112	61	34	6,9
1812	0	0			1	3140	42,5	167	115	127	84	136	62	11,5
1813	0	0			1	3160	45	135	95	106	86	83	48	7,1
1814	0	0			1	3055	47,5	156	86	105	104	56	29	5,9

1815	0	0			0	3080	47,5	138	94	105	84	84	45	7,1
1816	0	0			1	2860	50	166	111	124	90	43	29	3,9
1817	0	0			0	3230	55	136	84	95	112	79	43	8,8
1818	0	0			0	3445	60	144	97	110	82	67	39	5,5
1819	0	0			0	3510	67,5	116	83	91	102	86	44	8,7
1820	0	0			1	3475	77,5	165	117	129	99	77	37	7,6
1821	0	0			1	3155	77,5	152	103	116	84	101	48	8,5
1822	0	0			0	3495	77,5	135	95	104	91	105	53	9,6
1823	0	0			0	3365	82,5	134	89	102	110	66	38	7,3
1824	0	0			0	2965	82,5	143	93	107	84	73	41	6,1
1825	0	0			0	3615	85	143	89	103	112	68	35	7,6
1826	0	0			0	3615	92,5	140	88	99	116	66	33	7,7
1827	0	0			0	3310	92,5	145	96	106	98	58	32	5,7
1828	0	0			0	3930	92,5	219	103	141	100	99	42	10
1829	0	0			1	3990	97,5	186	105	120	107	61	26	6,5
1830	0	0			1	4030	97,5	143	93	104	87	97	50	8,3
1831	0	0			1	4575	100	156	92	102	114	71	34	8,1
1832	0	0			1	2780	62,5	150	94	106	102	72	37	7,3
1833	0	0	0		0	3220	72,5	127	84	95	122	78	38	9,5
1834	0	0	0		0	3220	72,5	127	73	91	102	109	53	11
1835	0	0			0	4060	97,5	135	95	106	123	53	28	6,5
1836	0	0			0	3395	55	156	103	114	115	53	29	6,1
1837	0	0			1	1850	20	106	78	86	102	65	34	6,7
1838	0	0			0	3040	55	152	103	115	75	104	56	7,7
1839	0	0			0	3310	62,5	149	104	115	112	61	30	6,9
1840	0	0			0	3310	62,5	132	92	101	99	73	36	7,3
1841	0	0			1	2680	15	124	92	101	87	89	49	7,7
1842	0	0			0	3110	50	140	103	113	101	74	40	7,5
1843	0	0			0	3110	50	140	96	107	94	66	38	6,3
1844	0	0			0	2250	32,5	153	115	125	100	84	46	8,4
1845	0	0			1	1680	10	147	103	114	91	74	35	6,7
1846	0	0			1	3550	90	139	102	113	107	85	38	9
1847	0	0			1	2770	40	128	96	104	99	61	34	6

1848	0	0			0	2825	22,5	118	76	86	106	82	42	8,7
1849	0	0			0	2825	22,5	140	91	103	83	88	44	7,3
1850	0	0			0	2595	10	139	97	108	99	55	31	5,5
1851	0	0			0	2595	10	130	88	98	110	48	29	5,2
1852	0	0			0	2005	2,5	140	93	104	110	80	43	8,9
1853	0	0			0	4168	87,5	169	105	123	78	131	60	10,3
1854	0	0			0	3310	45	113	86	94	88	71	43	6,3
1855	0	0			0	2350	7,5	148	100	111	101	70	38	7,1
1856	0	0			1	2010	40	125	88	98	88	67	42	5,8
1857	0	0			0	2280	37,5	153	110	124	89	91	44	8,1
1858	0	0			0	3215	52,5	134	94	104	85	95	48	8,1
1859	0	0			0	3785	97,5	158	103	116	109	59	33	6,5
1860	0	0			0	3785	97,5	121	90	100	90	75	43	6,8
1861	0	0			0	3785	97,5	131	86	99	89	68	40	6
1862	0	0			1	1865	5	153	100	115	77	57	33	4,3
1863	0	0			1	2910	30	143	103	114	109	61	37	6,7
1864	0	0			0	3520	40	158	98	116	104	76	35	7,8
1865	0	0			1	1920	2,5	125	87	97	99	78	44	7,7
1866	0	0			1	3680	80	177	109	124	88	78	39	6,9
1867	0	0			1	2405	20	130	85	96	103	72	43	7,4
1868	0	0			1	2980	60	143	91	103	119	58	30	6,9
1869	0	0			1	2511	32,5	164	113	123	106	63	33	6,6
1870	0	0			1	2910	55	177	116	131	94	119	63	11,3
1871	0	0			1	3110	72,5	108	77	86	128	62	32	7,9
1872	0	0			1	3445	60	140	95	107	89	92	44	8,2
1873	0	0			1	2825	45	185	110	126	127	83	36	10,6
1874	0	0			1	1642	10	144	91	103	85	82	44	7
1875	0	0			1	2640	62,5	124	95	104	100	58	33	5,7
1876	0	0			1	2640	62,5	113	75	85	91	76	46	6,9
1877	0	0			1	2640	62,5	129	79	89	69	92	58	6,3
1878	0	0			0	3750	85	172	102	115	116	104	42	12
1879	0	0			0	2545	22,5	160	109	121	87	85	44	7,4
1880	0	0			0	2570	15	174	100	111	99	78	45	7,7

1881	0	0			0	2445	10	137	85	97	73	109	55	8
1882	0	0			1	3185	70							
1883	0	0			1	3445	75	151	92	106	86	94	44	8
1884	0	0			1	2745	35	152	92	109	96	83	39	7,9
1885	0	0			0	2231	2,5	187	113	130	68	68	41	4,6
1886	0	0	0		0	3445	60	131	83	93	93	102		9,5
1887	0	0			1	3360	67,5	151	97	110	112	76	35	8,6
1888	0	0			0	4366	100	114	89	95	84	68		5,7
1889	0	0	0		1	1945	30	134	84	97	83	64	39	5,3
1890	0	0	0		1	1945	30	160	110	121	84	68	39	5,7
1891	0	0	0		1	1945	30	131	80	90	83	66	40	5,5
1892	0	0	0	1	1	2925	32,5	157	98	110	105	107		11,2
1893	0	0	0	1	1	3285	90	106	74	82	83	88		7,3
1894	0	0	1	0	0	3350	37,5	111	81	89	105	67	41	7
1895	0	0	1	0	0	3350	37,5	126	83	94	95	72	41	6,8
1896	0	0	1	0	0	3350	37,5	111	83	91	94	62	39	5,8
1897	0	0	0	0	1	3735	90	126	77	89	108	71	37	7,7
1898	0	0	0	0	1	3735	90	126	88	98	91	76	40	6,8
1899	0	0	0	0	1	3865	90	125	92	103	108	82	41	8,8
1900	0	0	0	0	1	3865	90	114	77	86	99	87	46	8,6
1901	0	0	0	0	1	3865	90	111	79	88	101	89	47	9
1902	0	0	1	0	1	2720	32,5	118	76	86	102	62	35	6,3
1903	0	0	1	0	1	2720	32,5	119	80	90	101	55	32	5,5
1904	0	0	1	0	1	2720	32,5	115	76	85	96	59	35	5,7
1905	0	0	2	0	1	2235	5	134	97	107	100	57		5,7
1906	0	0	0	0	1	2390	25	123	87	98	76	80	46	6,1
1907	0	0	0	0	1	2390	25	131	89	99	82	76	43	6,2
1908	0	0	0	0	1	2390	25	180	121	136	82	79	42	6,4
1909	0	0		0	1	2760	30	142	98	110	99	59	33	5,8
1910	0	0		0	1	2760	30	148	92	106	94	67	42	6,3
1911	0	0		0	1	2760	30	141	92	104	88	77	47	6,8
1912	0	0		0	0	2540	20	151	102	114	104	61	33	6,4
1913	0	0		0	0	2540	20	144	111	116	93	60	31	5,6

1914	0	0		0	0	2540	20	145	100	110	97	65	37	6,3
1915	0	0	0	0	1	2660	7,5	100	64	74	98	62	43	6,1
1916	0	0	0	0	1	2645	20	146	97	111	91	69	37	6,3
1917	0	0	0	0	1	2645	20	115	83	92	76	85	48	6,4
1918	0	0	0	0	1	2645	20	105	72	81	76	78	44	5,9
1919	0	0	0	0	1	2970	77,5	126	73	87	91	116		10,5
1920	0	0	0	0	1	2790	65	109	69	79	92	83	48	7,6
1921	0	0	0	0	1	2790	65	103	73	81	84	74	45	6,2
1922	0	0	0	0	0	4285	100	127	79	90	90	131	59	11,8
1923	0	0	0	0	0	2980	37,5	122	85	96	117	87	40	10,2
1924	0	0	0	0	0	2980	37,5	156	101	116	101	102	48	10,3
1925	0	0	0	0	0	2980	37,5	123	86	97	87	102	50	8,9
1926	0	0	1	0	0	3425	72,5	156	82	96	102	85	39	8,7
1927	0	0	1	0	0	3425	72,5	140	81	91	93	83	40	7,8
1928	0	0	0	0	1	3645	77,5	117	82	91	89	74	38	6,6
1929	0	0	0	0	1	3645	77,5	117	82	91	89	74	38	6,6
1930	0	0	1	0	1	2420	42,5	171	108	122	83	98	52	8,2
1931	0	0	1	0	1	2420	42,5	155	100	115	73	109	57	7,9
1932	0	0	1	0	1	2420	42,5	165	104	121	84	146	76	12,3
1933	0	0	1	0	0	3785	77,5	124	78	89	85	72		6,1
1934	0	0	0	0	1	2105	15	122	88	98	100	84		8,3
1935	0	1	0	0	0	3355	82,5	118	79	90	80	93		7,5
1936	0	0		0	1	3920	100	151	91	107	85	139		11,9
1937	0	0		0	0	2620	35	136	93	103	76	106		8,1
1938	0	0		0	0	2940	27,5	153	97	111	113	71		8,1
1939	0	0		0	1	2015	2,5	133	93	103	80	86		6,9
1940	0	0		0	1	1537	2,5	153	114	120	94	70		6,5
1941	0	0		0	1	2645	5	183	108	120	94	88		8,3
1942	0	0		0	0	1795	5	133	91	102	82	74		6
1943	0	0		0	0	2225	5	149	95	108	103	59		6,2
1944	0	0		0	0	2200	7,5	136	99	110	97	62		6
1945	0	0		0	1	2385	10	144	97	107	94	69		6,5
1946	0	0		0	0	2700	10	138	92	102	86	98		8,4

1947	0	0		0	1	1943	10	186	126	141	76	109		8,3
1948	0	0		0	0	2640	12,5	157	102	114	98	80		7,9
1949	0	0		0	0	2730	12,5	131	94	104	90	67		6
1950	0	0		0	0	3085	12,5	143	96	108	91	106		9,6
1951	0	0		0	0	1855	12,5	139	90	102	80	93		7,3
1952	0	0		0	1	1802	17,5	180	119	133	76	82		6,2
1953	0	0		0	1	2755	17,5	166	109	121	94	51		4,7
1954	0	0		0	1	2930	20	143	102	113	81	61		4,9
1955	0	0		0	0	2865	20	158	100	110	82	73		6
1956	0	0		0	0	3260	25	128	93	103	85	115		9,9
1957	0	0		0	0	2520	25	190	109	130	88	128		11,2
1958	0	0		0	0	3000	30	127	92	102	84	103		8,6
1959	0	0		0	1	2465	30	150	109	119	78	97		7,6
1960	0	0		0	1	2925	32,5	196	112	132	125	84		10,5
1961	0	0		0	0	2465	35	149	86	102	120	83		10
1962	0	0		0	0	3355	35	131	90	102	93	57		5,3
1963	0	0		0	1	3240	40	134	92	103	113	71		8
1964	0	0		0	1	3260	42,5	172	98	114	104	97		10,1
1965	0	0		0	0	2720	45	199	119	141	106	37		3,9
1966	0	0		0	1	2345	50	151	97	111	98	44		4,4
1967	0	0		0	1	2875	60	165	101	116	89	159		14,2
1968	0	0		0	1	2235	62,5	150	92	107	101	88		8,9
1969	0	0		0	0	3305	62,5	166	102	117	77	138		10,6
1970	0	0		0	0	3705	70	160	107	120	73	91		6,6
1971	0	0		0	0	3440	72,5	143	98	110	87	87		7,5
1972	0	0		0	0	3640	77,5	168	103	119	104	91		9,5
1973	0	0		0	1	2970	77,5	146	97	108	94	126		11,8
1974	0	0		0	1	3715	80	161	105	121	97	124		12,1
1975	0	0		0	0	3670	80	153	111	120	109	119		13
1976	0	0		0	0	4175	90	168	104	118	83	88		7,3
1977	0	0		0	1	3940	92,5	124	84	95	90	114		10,2
1978	0	0		0	0	3780	92,5	180	122	134	100	84		8,4
1979	0	0		0	0	3535	95	127	96	105	93	96		9

1980	0	0		0	0	3885	95	151	101	115	107	86		9,2
1981	0	0		0	1	3735	100	168	113	127	82	110		9,1
1982	0	0		0	1	2870	50	133	98	108	76	76		5,7
1983	0	0		0	1	1650	2,5	124	90	100	93	99		9,2
1984	0	0		0	1	2175	22,5	188	124	139	84	79		6,6
1985	0	0		0	1	2855	70	150	97	112	90	91		8,2
1986	0	0		1	0	1858	12,5	157	99	113	101	60		6,1
1987	0	0		1	0	3060	22,5	164	114	127	103	82		8,4
1988	0	0		1	0	2535	27,5	142	103	114	81	101		8,2
1989	1	0			1	2320	15	110	74	83	98	107	58	10,5
1990	1	0			1	2320	15	116	78	88	88	94	52	8,2
1991	0	0			0	3170	17,5	115	77	87	80	97	53	7,7
1992	0	0			0	3170	17,5	105	73	83	87	89	49	7,8
1993	0	0			1	3240	27,5	113	71	81	112	67	38	7,4
1994	0	0			1	3240	27,5	108	74	84	113	68	37	7,7
1995	0	0			0	3430	57,5	149	89	100	117	59	30	6,8
1996	0	0			0	3430	57,5	108	81	89	103	73	38	7,5
1997	0	0			0	3660	65	136	87	97	128	66	38	8,4
1998	0	0			1	3630	77,5	124	82	93	93	86	43	8
1999	0	0			1	3205	80	108	72	81	84	77	49	6,5
2000	0	0			1	3205	80	117	78	87	87	80	51	6,9
2001	0	0			1	3880	90	101	66	75	117	69	37	8,1
2002	0	0			1	3880	90	125	84	93	95	70	38	6,6
2003	0	0			0	2680	22,5	111	80	88	102	90	42	9,1
2004	0	0			0	2680	22,5	123	86	95	95	99	46	9,4
2005	0	0			0	495	0	152	104	117	99	58	34	5,7
2006	0	0			0	2100	2,5	158	109	121	104	61	39	6,4
2007	0	0			1	2515	2,5	126	84	96	122	86	42	10,4
2008	0	0			1	2515	2,5	146	91	106	109	91	42	9,9
2009	0	0			0	2530	5	144	98	110	94	56	36	5,2
2010	0	0			1	2575	5	138	101	112	78	88	48	6,9
2011	0	0			0	2275	7,5	132	91	101	109	53	32	5,8
2012	0	0			1	950	12,5	124	93	103	102	102	46	10,5

2013	0	0			1	2985	12,5	116	83	92	75	94	53	7
2014	0	0			1	2700	15	149	100	114	98	69	40	6,7
2015	0	0			1	2935	15	158	100	113	111	106	48	11,8
2016	0	0			1	2770	15	142	95	107	90	67	36	6,1
2017	0	0			1	2995	17,5	130	87	96	139	45	25	6,2
2018	0	0			0	2820	17,5	124	90	100	87	62	37	5,4
2019	0	0			1	2910	20	135	96	105	88	66	35	5,8
2020	0	0			1	2150	22,5	166	114	125	77	95	46	7,5
2021	0	0			1	3080	25	153	97	112	117	69	41	8,1
2022	0	0			1	2965	25	152	102	114	100	54	34	5,4
2023	0	0			0	2735	25	144	101	113	95	50	32	4,8
2024	0	0			1	3140	27,5	165	99	111	105	75	37	7,7
2025	0	0			1	2740	30	160	109	123	98	68	36	6,7
2026	0	0			1	2990	32,5	146	98	109	77	109	59	8,5
2027	0	0			0	3325	32,5	157	103	116	106	105	47	11,1
2028	0	0			0	2850	35	133	95	104	84	90	44	7,6
2029	0	0			1	3260	42,5	112	76	85	105	66	36	7
2030	0	0			0	3450	45	148	101	112	96	74	37	7,2
2031	0	0			0	3685	45	141	104	114	116	76	36	8,8
2032	0	0			1	3330	47,5	140	91	107	93	96	49	8,9
2033	0	0			1	2835	47,5	153	112	121	89	58	32	5,2
2034	0	0			1	2970	50	164	105	122	112	92	46	10,4
2035	0	0			1	3330	50	165	96	114	98	71	36	6,9
2036	0	0			0	3130	52,5							
2037	0	0			0	3130	52,5	109	77	86	98	122	49	12,1
2038	0	0			1	2745	60	136	90	101	112	53	32	6
2039	0	0			1	3215	65	136	98	108	97	57	29	5,6
2040	0	0			0	3660	65	147	92	102	141	40	19	5,6
2041	0	0			0	3560	70	158	108	121	99	70	31	7,1
2042	0	0			1	3695	80	146	89	101	125	55	24	7,6
2043	0	0			0	3840	80	141	94	105	97	102	47	9,9
2044	0	0			0	3520	90	141	93	106	90	72	36	6,5
2045	0	0			0	3685	90	135	95	104	91	72	43	6,6

2046	0	0			0	3825	95	161	105	119	105	101	44	10,5
2047	0	0			1	3845	95	198	124	142	87	83	38	7,3
2048	0	0			1	3875	95	157	107	119	89	99	50	8,8
2049	0	0			0	3825	95	124	86	95	97	102	48	10
2050	0	0			1	4070	97,5	169	115	129	111	109	43	12
2051	0	0			0	4170	97,5	172	121	131	100	101	43	10,2
2052	0	0			0	5090	100	143	98	109	92	95	44	8,8
2053	0	0	1		1	2860	27,5	128	76	89	105	82	38	8,6
2054	0	0	1		1	2860	27,5	135	91	102	110	92	43	10,1
2055	0	0			1	2285	17,5	146	102	112	112	75	38	8,5
2056	0	0			1	3535	70	118	86	95	126	84	35	10,6
2057	0	0			1	2730	12,5	156	92	106	111	60	30	6,7
2058	0	0			0	2755	7,5	139	94	106	97	66	38	6,4
2059	0	0			0	2905	15	124	80	89	117	66	36	7,6
2060	0	0			0	2905	15	122	73	83	85	81	47	6,9
2061	0	0			0	2905	15	127	77	87	88	72	41	6,4
2062	0	0			1	1190	2,5	126	95	102	79	64	35	5,1
2063	0	0			1	3715	90	121	82	92	83	80	40	6,6
2064	0	0			0	3220	22,5	138	94	105	85	89	47	7,5
2065	0	0			0	2750	47,5	144	94	105	92	84	45	7,7
2066	0	0			1	2925	27,5	154	107	117	94	53	30	5
2067	0	0			0	3485	50	157	95	110	71	82	46	5,8
2068	0	0			1	3470	85	164	105	116	124	96	46	11,9
2069	0	0			0	3280	77,5	139	100	111	115	51	28	5,8
2070	0	0			0	1585	5	144	95	108	70	73	37	5
2071	0	0			1	2640	20	140	100	111	92	64	38	6
2072	0	0			1	2425	7,5	142	88	104	102	60	35	6,1
2073	0	0			1	2425	7,5	144	94	106	96	87	48	8,4
2074	0	0			1	3460	62,5	148	96	110	94	78	34	7,3
2075	0	0			1	3930	92,5	136	96	106	122	74	35	9,1
2076	0	0			1	3345	52,5	120	81	90	126	48	25	6,1
2077	0	0	0		0	950	2,5	121	82	92	87	53		4,6
2078	0	0			0	2610	2,5	123	90	99	87	84	46	7,3

2079	0	0			0	2915	15	134	93	104	113	63	34	7,1
2080	0	0			0	3250	65	153	95	109	105	73	35	7,7
2081	0	0			1	2660	72,5	121	82	93	111	70	32	7,7
2082	0	0			0	2415	17,5	148	97	110	102	86	45	8,8
2083	0	0	0	1	0	3720	47,5	148	96	109	115	66	37	7,5
2084	0	0	0	1	0	3720	47,5	116	77	87	94	81	47	7,7
2085	0	0	0	1	0	3720	47,5	124	87	96	90	78	46	7
2086	0	0	1	1	1	3600	85	155	96	107	102	70	33	7,1
2087	0	0	0	2	0	3210	40	136	83	97	102	75	38	7,6
2088	0	0	0	2	0	3210	40	140	83	93	103	63	37	6,5
2089	0	0	0	2	0	3210	40	123	87	98	110	70	40	7,7
2090	0	0	0	4	1	3040	32,5	118	76	86	106	79	45	8,4
2091	0	0	0	4	1	3040	32,5	128	81	91	92	72	42	6,5
2092	0	0		0	1	3005	17,5	110	72	81	89	67		5,9
2093	0	0		0		3405	65	117	75	85	110	90	50	9,7
2094	0	0		0		3405	65	105	61	71	114	70	41	8
2095	0	0		0		3405	65	114	77	85	96	58	35	5,6
2096	0	0	2	0	0	3935	75	165	101	115	119	83	38	9,9
2097	0	0	2	0	0	3935	75	168	102	118	110	102	46	11,4
2098	0	0	0	0	0	3800	65	103	71	80	78	117	65	9,1
2099	0	0	0	0	0	3800	65	111	73	83	79	95	55	7,4
2100	0	0	0	0	1	2740	5	164	111	120	100	94	46	9,1
2101	0	0	0	0	1	2740	5	118	79	90	76	122	63	9,2
2102	0	0	0	0	1	2740	5	142	88	100	94	111	57	10,4
2103	0	0	0	0	0	3410	40	119	76	87	90	55		5
2104	0	0	1	0	1	3620	92,5	153	111	121	98	63		6,2
2105	0	0	0	0	0	3040	25	117	79	88	93	87		8,1
2106	0	0	1	0	1	2985	52,5	86	48	58	85	105	58	8,8
2107	0	0	1	0	1	2985	52,5	127	81	91	96	79	45	7,6
2108	0	0	1	0	1	2985	52,5	126	75	87	85	93	54	7,9
2109	0	0	0	0	1	4425	100	134	94	103	102	87		8,8
2110	0	0	0	0	0	4125	95	124	86	97	101	76		7,7
2111	0	0	0	0	0	3470	62,5	115	79	89	100	80	42	8

2112	0	0	0	0	0	3470	62,5	118	76	86	91	90	48	8,2
2113	0	0	1	0	0	3080	15	122	84	94	87	68	39	5,9
2114	0	0	1	0	0	3080	15	113	83	92	96	74	41	7,1
2115	0	0	1	0	0	3080	15	111	77	87	87	79	46	6,9
2116	0	0	0	0	1	3670	77,5	118	78	88	99	50		4,9
2117	0	0	0	0	0	2585	32,5	111	80	90	95	61	40	5,8
2118	0	0	0	0	0	2585	32,5	98	71	79	98	52	34	5,2
2119	0	0	0	0	0	2865	20	119	89	97	101	86	51	8,7
2120	0	0	0	0	0	2865	20	114	75	85	97	64	39	6,2
2121	0	0	0	0	0	3850	90	124	88	98	98	80	37	7,7
2122	0	0	0	0	0	3850	90	125	81	89	89	89	41	7,9
2123	0	0	1	0	0	3580	82,5	149	101	115	73	96	51	7
2124	0	0	1	0	0	3580	82,5	107	73	83	96	74	44	7,1
2125	0	0	1	0	0	3580	82,5	114	71	81	83	108	62	8,9
2126	0	0	0	0	1	3300	62,5	140	81	91	101	86	54	8,7
2127	0	0	0	0	1	3300	62,5	118	68	81	93	74	47	6,9
2128	0	0	0	0	1	2700	5	135	101	111	102	47		4,8
2129	0	0	0	0	0	3800	77,5	113	78	88	99	82	46	8,2
2130	0	0	0	0	0	3800	77,5	116	81	91	100	74	43	7,4
2131	0	0	2	0	1	2995	17,5	127	72	83	109	65	42	7,1
2132	0	0	2	0	1	2995	17,5	102	74	82	103	68	44	7
2133	0	0	0	0	1	3335	37,5	128	81	93	115	75	39	8,6
2134	0	0	0	0	1	3335	37,5	127	89	99	95	82	45	7,8
2135	0	0	0	0	1	3335	37,5	119	74	83	106	88	46	9,3
2136	0	0	0	0	0	3620	50	123	83	94	96	82		7,8
2137	0	0	0	0	0	3130	10	124	83	94	83	75	45	6,2
2138	0	0	0	0	0	3130	10	118	83	93	93	58	36	5,4
2139	0	0	0	0	1	3195	50	135	85	96	115	78	45	8,9
2140	0	0	0	0	1	3195	50	124	79	89	104	73	43	7,6
2141	1	0	0	0	0	2415	7,5	133	92	98	93	75	39	7
2142	1	0	0	0	0	2415	7,5	112	75	83	97	86	44	8,3
2143	1	0	0	0	1	3160	90	143	96	107	109	101		11,1
2144	0	1	0	0	0	2110	52,5							

2145	0	1	0	0	0	2110	52,5	141	85	95	115	59	35	6,8
2146	0	0		0	1	2575	2,5	152	102	117	108	76		8,1
2147	0	0		0	0	2450	2,5	123	92	100	111	52		5,8
2148	0	0		0	0	950	2,5	175	99	115	97	61		5,9
2149	0	0		0	0	680	2,5	175	101	118	73	101		7,4
2150	0	0		0	0	1345	5	125	91	102	86	76		6,5
2151	0	0		0	1	2395	10	151	91	107	93	83		7,7
2152	0	0		0	0	3100	15	156	105	118	87	84		7,3
2153	0	0		0	1	3005	17,5	153	96	112	92	53		4,8
2154	0	0		0	0	3270	20	144	110	118	104	73		7,6
2155	0	0		0	1	2960	25	181	114	130	76	93		7
2156	0	0		0	0	3310	30	161	101	118	88	86		7,6
2157	0	0		0	0	3220	37,5	139	98	108	97	89		8,6
2158	0	0		0	1	1160	40	143	98	110	104	82		8,5
2159	0	0		0	0	1980	40	140	94	104	83	78		6,5
2160	0	0		0	1	2615	42,5	153	96	109	111	70		7,8
2161	0	0		0	1	1850	45	127	92	102	90	66		5,9
2162	0	0		0	0	3620	50	147	93	104	104	68		7,1
2163	0	0		0	0	3545	55	149	104	114	91	111		10,2
2164	0	0		0	0	3020	55	160	105	118	111	91		10,1
2165	0	0		0	0	3570	57,5	152	98	111	93	61		5,7
2166	0	0		0	0	3605	60	149	103	116	94	60		5,6
2167	0	0		0	0	3495	65	149	104	116	93	146		13,5
2168	0	0		0	1	3235	67,5	123	88	97	95	99		9,4
2169	0	0		0	0	3815	67,5	113	93	100	93	76		7
2170	0	0		0	1	3670	77,5	136	91	102	102	62		6,3
2171	0	0		0	0	3635	77,5	141	88	102	87	90		7,9
2172	0	0		0	1	3510	80	124	92	101	93	119		11
2173	0	0		0	1	3505	87,5	164	94	109	100	77		7,7
2174	0	0		0	1	4000	97,5	138	101	111	89	80		7,1
2175	0	0		0	1	4420	97,5	131	88	98	102	99		10,2
2176	0	0		0	0	4125	97,5	143	101	113	98	94		9,2
2177	0	0		0	0	4425	100	134	90	100	94	97		9,1

2178	0	0		0	0	4260	97,5	175	113	125	106	88		9,4
2179	0	0		0	0	2720	5	168	104	124	89	99		8,7
2180	0	0		0	0	2570	47,5	128	86	98	100	76		7,6
2181	0	0			0	2630	2,5	127	90	101	83	73	43	6
2182	0	0			0	2630	2,5	128	88	99	92	60	35	5,5
2183	0	0			0	3155	12,5	109	79	88	95	61	39	5,8
2184	0	0			0	3155	12,5	117	85	94	91	55	35	5
2185	0	0			0	4165	90	109	74	82	89	99	57	8,8
2186	0	0			0	4165	90	123	78	88	93	83	49	7,7
2187	0	0			1	3445	75	132	90	102	86	94	47	8,1
2188	0	0			1	3445	75	127	90	101	85	129	65	11
2189	0	0			0	4075	85	168	91	111	114	88	43	10
2190	0	0			0	4075	85	164	101	116	103	121	57	12,4
2191	0	0			1	2880	12,5	122	86	94	86	91	47	7,8
2192	0	0			1	2880	12,5	130	86	97	88	92	48	8,1
2193	0	0			0	2630	2,5	141	98	109	89	76	39	6,8
2194	0	0			0	2630	2,5	173	115	130	80	83	44	6,7
2195	0	0			1	1890	50	163	95	112	78	154	70	12
2196	0	0			0	3757	75	113	76	86	98	105	48	10,3
2197	0	0			1	2465	15	143	102	113	98	96	47	9,5
2198	0	0			1	2465	15	131	82	92	107	107	53	11,5
2199	0	0			1	2491	32,5	115	76	87	114	60	33	6,8
2200	0	0			1	2491	32,5	115	73	83	96	76	43	7,3
2201	0	0			0	3670	80	117	85	95	92	67	38	6,2
2202	0	0			0	1860	2,5	130	85	96	105	64	38	6,7
2203	0	0			0	1860	2,5	138	94	104	110	58	34	6,4
2204	0	0			0	1490	2,5	140	102	113	81	84	43	6,8
2205	0	0			0	970	0	135	88	99	110	60	33	6,6
2206	0	0			1	2835	20	148	104	114	102	87	48	8,9
2207	0	0			1	3160	67,5	128	88	97	110	56	29	6,2
2208	0	0	1		1	4530	97,5	124	89	99	119	65	31	7,8
2209	0	0	1		1	4530	97,5	138	88	99	89	83	40	7,4
2210	0	0	1		1	3330	47,5	102	80	85	80	109		8,7

2211	0	0	1		1	120	0	148	100	113	90	76	40	6,8
2212	1	0			0	1700	2,5	88	55	65	86	108	66	9,3
2213	1	0			0	1700	2,5	122	76	88	76	106	65	8,1
2214	1	0			0	1700	2,5	109	73	83	80	87	55	6,9
2215	0	0	0	3	1	3075	25	146	99	111	94	121	67	11,4
2216	0	0	0	3	1	3075	25	124	89	101	89	79	45	7,1
2217	0	0	0	4	0	3395	70	124	84	94	112	80	41	8,9
2218	0	0	0	4	0	3395	70	118	84	94	92	85	45	7,8
2219	0	0	0	4	0	3395	70	122	82	92	84	95	49	7,9
2220	0	0	0	4	1	3085	50	127	83	93	105	72	41	7,6
2221	0	0	0	4	1	3085	50	114	83	92	105	59	34	6,1
2222	0	0	0	0	0	4015	82,5	121	83	93	92	102		9,3
2223	0	0		0	1	3125	27,5	101	74	81	94	68	41	6,3
2224	0	0		0	1	3125	27,5	106	79	88	93	66	41	6,2
2225	0	0		0	1	3125	27,5	106	81	90	100	48	31	4,8
2226	0	0	0	0	0	2985	50	128	90	99	102	78	46	8
2227	0	0	0	0	0	2985	50	122	93	100	103	67	41	6,9
2228	0	0	0	0	0	2985	50	130	96	106	103	59	36	6,1
2229	0	0		0	1	700	17,5	117	80	90	115	45	28	5,2
2230	0	0		0	1	700	17,5	166	108	121	74	59	35	4,3
2231	0	0	0	0	1	3925	97,5	133	82	94	84	115	54	9,6
2232	0	0	0	0	1	3925	97,5	134	87	98	101	84	43	8,5
2233	0	0	0	0	1	3205	37,5	134	90	103	92	73		6,6
2234	0	0	0	0	0	3755	75	153	108	119	92	79	43	7,3
2235	0	0	0	0	0	3755	75	176	118	132	93	76	43	7,1
2236	0	0	0	0	1	3415	55							
2237	0	0	0	0	1	3415	55	143	98	110	86	101	48	8,8
2238	0	0	0	0	0	3540	55	124	89	99	102	89	45	9,1
2239	0	0	0	0	0	3540	55	127	90	101	104	88	45	9,2
2240	0	0	0	0	0	3540	55	124	89	98	96	88	46	8,5
2241	0	0	0	0	0	3055	15	164	113	124	106	74	37	7,8
2242	1	0	0	0	0	3055	15	148	98	112	86	98	49	8,3
2243	0	0	0	0	1	4175	97,5	127	81	92	94	76		7,1

2244	0	0	0	0	0	3720	90	124	76	88	85	114	58	9,6
2245	0	0	0	0	0	3720	90	107	79	87	84	126	63	10,6
2246	0	0	0	0	0	3760	85	153	92	108	110	75	38	8,2
2247	0	0	0	0	0	3760	85	153	86	98	104	82	41	8,6
2248	0	0	0	0	0	3760	85	143	86	101	119	69	34	8,3
2249	0	0	0	0	0	3170	35	115	75	86	108	68		7,3
2250	0	0	0	0	0	3760	75	136	96	107	105	99		10,4
2251	0	0	0	0	0	2865	27,5	119	86	96	91	83	49	7,5
2252	0	0	0	0	0	3340	72,5	144	102	113	91	72		6,5
2253	0	0	0	0	1	3140	27,5	147	85	103	77	136		10,4
2254	0	0	0	0	0	2905	57,5	152	99	112	118	70	41	8,3
2255	0	0	0	0	0	2905	57,5	147	91	104	101	69	41	7,1
2256	0	0	0	0	0	2905	57,5	159	95	107	96	63	37	6,1
2257	0	0	0	0	1	2690	10	177	110	127	92	85		7,8
2258	0	0		0	0	3340	72,5	183	130	142	100	65		6,5
2259	0	0		0	1	3205	85	135	96	108	87	99		8,7
2260	0	0		1	1	2550	7,5	163	108	123	80	118		9,4
2261	0	0			0	3165	47,5	125	90	100	99	83	44	8,2
2262	0	0			0	3165	47,5	138	90	100	115	85	43	9,7
2263	0	0			1	2520	2,5	151	110	121	88	58	36	5,2
2264	0	0			1	2520	2,5	130	81	92	82	75	45	6,2
2265	0	0			1	2095	7,5	133	81	91	89	89	49	8
2266	0	0			1	2455	7,5	109	67	78	87	95	53	8,3
2267	0	0			1	2760	7,5	124	66	77	81	117	64	9,5
2268	0	0			1	3035	7,5	123	76	87	119	79		9,4
2269	0	0			1	2265	7,5	133	80	94	106	75	37	8
2270	0	0			0	2515	7,5	124	77	89	74	69	45	5,1
2271	0	0			1	2070	7,5	138	100	108	105	95	44	9,9
2272	0	0			1	1645	7,5	124	69	83	91	81	50	7,4
2273	0	0			1	2315	7,5	127	79	95	93	111	52	10,3
2274	0	0			0	2053	7,5	124	82	92	118	60	39	7
2275	0	0			1	2535	7,5	115	71	78	95	85	48	8,1
2276	0	0			1	2535	7,5	123	78	87	105	79	46	8,3

2277	0	0	0	3	0	2805	7,5	130	81	92	131	63	35	8,3
2278	0	0	0	3	0	2805	7,5	123	83	93	120	69	37	8,2
2279	0	0	0	1	1	2760	7,5	116	74	84	80	76	48	6,1
2280	0	0	0	1	1	2760	7,5	101	61	70	103	79	50	8,1
2281	0	0	1	1	0	2855	7,5	128	86	97	122	61		7,4
2282	0	0	0	1	1	3030	7,5	110	73	82	74	75		5,5
2283	0	0	0	4	0	2910	7,5	114	75	86	98	58	38	5,6
2284	0	0	0	4	0	2910	7,5	114	75	86	98	58	38	5,6
2285	0	0	0	4	1	2805	7,5	113	71	81	89	89	50	7,9
2286	0	0	0	4	1	2805	7,5	119	78	88	74	94	54	7
2287	0	0	0	0	1	2805	7,5	108	81	89	115	57	35	6,5
2288	0	0	0	0	1	2805	7,5	108	73	83	122	67	40	8,2
2289	0	0	ovulatie inductie	0	1	2100	7,5	108	75	84	87	64		5,6
2290	0	0	0	0	0	2940	7,5	103	67	76	77	89	57	6,8
2291	0	0	0	0	0	2940	7,5	109	72	83	74	96	63	7,1
2292	0	0	1	0	0	3040	7,5	101	68	77	100	66	42	6,6
2293	0	0	1	0	0	3040	7,5	99	72	80	103	55	36	5,7
2294	0	0	0	0	0	2615	7,5	107	68	78	90	97	55	8,8
2295	0	0	0	0	0	2615	7,5	114	72	81	89	102	58	9,1
2296	0	0	0	0	0	3135	7,5	103	70	79	99	80	47	7,9
2297	0	0	0	0	0	3135	7,5	101	74	82	93	68	41	6,3
2298	0	0	0	0	0	3180	7,5	114	74	85	75	98	60	7,3
2299	0	0	0	0	0	3180	7,5	115	74	86	74	99	59	7,3
2300	0	0	1	0	1	2905	7,5	143	93	109	97	57	34	5,5
2301	0	0	1	0	1	2905	7,5	124	85	96	105	66	39	6,9
2302	0	0	0	0	0	2785	7,5	122	86	96	112	75	40	8,4
2303	0	0	0	0	0	2785	7,5	116	75	86	107	83	44	8,8
2304	0	0	0	0	0	3025	7,5	124	67	81	91	86	47	7,8
2305	0	0	0	0	0	3025	7,5	132	77	88	92	104	56	9,5
2306	0	0	0	0	1	2555	7,5	114	73	83	75	68	45	5,1
2307	0	0	0	0	1	2555	7,5	113	74	83	86	64	41	5,5
2308	0	0	0	0	0	3040	7,5	111	77	87	80	80		6,4
2309	0	0	0	0	0	1955	7,5	119	83	92	118	40	25	4,7

2310	0	0	0	0	0	1955	7,5	103	70	79	101	71	44	7,2
2311	0	0	0	0	0	2945	7,5	96	71	79	85	51	33	4,4
2312	0	0	0	0	0	2945	7,5	104	70	78	107	36	23	3,9
2313	0	0	0	0	0	2935	7,5	118	70	83	68	103	60	7
2314	0	0	0	0	0	2935	7,5	121	70	79	63	97	56	6,1
2315	0	0	0	0	0	3050	7,5	128	85	97	109	74	38	8,1
2316	0	0	0	0	0	3050	7,5	119	79	88	109	76	40	8,2
2317	0	0	0	0	0	3050	7,5	112	74	83	98	69	36	6,7
2318	0	0	2	0	0	2945	7,5	119	72	82	93	128		11,9
2319	0	0			1	2690	7,5	142	86	98	97	79	43	7,7
2320	0	0			1	2690	7,5	130	88	99	96	77	42	7,3
2321	0	0			0	1995	7,5	108	79	87	107	57	35	6,1
2322	0	0			0	3180	7,5	105	72	82	93	68	41	6,3
2323	0	0			0	3180	7,5	108	67	78	94	74	43	7
2324	0	0			0	1995	7,5	117	71	81	91	96	54	8,7
2325	0	0			1	2630	5	122	75	86	94	85	46	8
2326	0	0			0	2845	5	151	96	108	103	59	30	6,1
2327	0	0			0	2840	5	172	77	94	100	87	47	8,7
2328	0	0			0	2690	5	159	89	106	114	59	31	6,8
2329	0	0			0	2450	5	114	83	92	100	72	46	7,2
2330	0	0			0	2255	5	152	94	106	101	77	42	7,8
2331	0	0			0	2890	5	120	90	97	93	53	32	4,9
2332	0	0	0		0	2630	5	119	81	90	93	87	47	8,1
2333	0	0	0		0	2630	5	113	73	83	93	102	54	9,5
2334	0	0			0	2180	5	118	79	89	76	60	39	4,6
2335	0	0	0	1	0	2435	5	113	66	77	93	95	59	8,9
2336	0	0	0	1	0	2435	5	101	69	79	96	85	53	8,1
2337	0	0	0	1	0	2535	5	121	75	85	68	75	52	5,1
2338	0	0	0	1	1	2550	5	103	61	73	87	67	45	5,8
2339	0	0	0	1	1	2550	5	100	56	66	75	87	57	6,5
2340	0	0	0	1	0	2455	5	113	73	82	106	75	45	7,9
2341	0	0	0	1	0	2455	5	109	73	80	112	76	47	8,5
2342	0	0	0	1	0	2950	5	122	81	91	106	60		6,4

2343	0	0	0	4	1	2210	5	134	76	92	82	92		7,4
2344	0	0	0	0	1	2585	5	117	79	90	104	65	36	6,7
2345	0	0	0	0	1	2480	5	106	73	83	111	65	36	7,2
2346	0	0	0	0	1	2585	5	102	74	83	85	78	43	6,6
2347	0	0	0	0	0	2860	5	99	61	71	101	68	40	6,9
2348	0	0	0	0	1	2480	5	118	78	88	98	72	41	7
2349	0	0	0	0	0	2710	5	101	47	62	82	88	52	7,2
2350	0	0	0	0	0	2860	5	100	59	68	90	86	49	7,7
2351	0	0	0	0	0	2710	5	106	66	76	79	102	58	8,1
2352	0	0	0	0	1	2290	5							
2353	0	0	0	0	1	2290	5	118	74	85	113	72	40	8,2
2354	0	0	0	0	1	2290	5	110	74	84	103	66	38	6,8
2355	0	0	0	0	0	2510	5	102	67	76	100	56		5,6
2356	0	0	0	0	1	2355	5	131	83	95	104	68	38	7
2357	0	0	0	0	1	2355	5	135	91	102	101	78	43	7,8
2358	0	0	0	0	1	2355	5	128	75	86	98	75	42	7,3
2359	0	0	0	0	0	2830	5	159	95	109	93	74	42	6,9
2360	0	0	0	0	0	2690	5	99	70	79	92	51	34	4,6
2361	0	0	0	0	0	2690	5	121	72	86	81	57	39	4,7
2362	0	0	1	0	0	2840	5	110	79	89	94	57	36	5,4
2363	0	0	1	0	0	2840	5	127	79	93	80	65	41	5,2
2364	0	0	0	0	1	2750	5	98	63	72	99	41	32	4,1
2365	0	0	0	0	1	2750	5	101	58	68	99	56	43	5,6
2366	0	0	0	0	0	2880	5	110	75	85	110	70	40	7,7
2367	0	0	0	0	0	2880	5	107	68	79	92	83	48	7,7
2368	0	0	0	0	0	2880	5	124	78	89	98	75	43	7,3
2369	0	0	0	0	0	2885	5	93	65	74	116	46	29	5,3
2370	0	0		0	1	1370	5	122	80	90	75	56		4,3
2371	0	0			0	2965	5	111	71	81	101	84	46	8,5
2372	0	0			0	2645	5	107	68	77	92	103	55	9,3
2373	0	0			0	2645	5	114	73	83	84	108	57	8,8
2374	0	0			0	2965	5	108	72	82	96	84	44	8
2375	0	0			0	2565	5	111	79	87	112	75	42	8,4

2376	0	0			0	2565	5	123	80	91	97	78	44	7,6
2377	0	0			1	1925	2,5	110	78	86	87	88	52	7,6
2378	0	0			1	1650	2,5	111	77	87	98	89	44	8,7
2379	0	0			0	673	2,5	114	71	82	81	86	46	7
2380	0	0			1	2420	2,5	127	88	98	99	47	29	4,7
2381	0	0			0	2115	2,5	130	74	88	78	96	54	7,5
2382	0	0			1	1635	2,5	108	68	79	93	75	44	7
2383	0	0			0	2020	2,5	135	87	97	93	96	54	9
2384	0	0			1	2470	2,5	100	81	87	83	99	54	8,2
2385	0	0			0	1235	2,5	135	86	98	119	51	29	6,1
2386	0	0			0	2750	2,5	99	65	74	96	79	54	7,6
2387	0	0			1	2290	2,5	144	91	106	92	66	34	6,1
2388	0	0			1	2440	2,5	120	81	91	88	69	39	6,1
2389	0	0			0	1250	2,5	131	81	93	81	81	44	6,6
2390	0	0			1	2625	2,5	139	94	106	99	72	40	7,1
2391	0	0			1	1255	2,5	128	93	103	97	70	38	6,8
2392	0	0			0	2140	2,5	113	66	77	101	81	48	8,2
2393	0	0			1	1680	2,5	136	74	84	110	96	47	10,5
2394	0	0			0	2510	2,5	114	78	87	104	80	43	8,3
2395	0	0			0	2750	2,5	94	60	70	70	90	62	6,3
2396	0	0			1	1695	2,5	117	64	77	72	88	54	6,4
2397	0	0			0	2660	2,5	130	85	94	121	71	39	8,6
2398	0	1			1	564	2,5	114	75	85	94	121	54	11,4
2399	0	0			1	2645	2,5	127	83	94	118	75	39	8,9
2400	0	0			1	500	2,5	108	76	86	105	79	41	8,3
2401	0	0	0		0	2890	2,5	114	70	81	81	79	50	6,4
2402	0	0	0		0	2890	2,5	101	70	80	89	60	39	5,3
2403	0	0			0	2140	2,5	138	91	103	94	64	33	6,1
2404	0	0	0	3	0	2600	2,5	105	64	73	102	81	52	8,3
2405	0	0	0	3	0	2600	2,5	114	68	78	106	85	54	9
2406	0	0	0	3	1	2220	2,5	109	79	87	95	78		7,4
2407	0	0	2	1	0	2715	2,5	121	74	87	89	97	54	8,6
2408	0	0	2	1	0	2715	2,5	133	69	85	91	91	53	8,3

2409	0	0	0	1	0	2900	2,5	105	69	78	91	73	45	6,6
2410	0	0	0	1	0	2900	2,5	114	71	81	86	79	49	6,8
2411	0	0	0	1	0	2110	2,5	135	83	97	110	84	43	9,2
2412	0	0	0	1	0	2110	2,5	138	91	103	95	102	53	9,7
2413	0	0	0	1	0	2110	2,5	141	77	88	95	86	45	8,1
2414	0	0	0	1	1	2085	2,5	107	60	71	82	73	48	6
2415	0	0	0	1	1	2085	2,5	112	73	81	96	60	39	5,8
2416	0	0	0	1	0	2450	2,5	114	62	74	97	65		6,3
2417	0	0	0	1	1	2440	2,5	107	71	80	108	64		6,9
2418	0	0	0	2	1	2345	2,5	100	70	79	96	59	39	5,6
2419	0	0	0	2	1	2445	2,5	106	70	80	97	74		7,2
2420	0	0	0	4	1	2410	2,5	124	80	91	100	77	45	7,7
2421	0	0	0	4	1	2410	2,5	116	78	88	120	62	35	7,4
2422	0	0	0	4	0	2570	2,5	124	76	86	92	77	40	7,1
2423	0	0	0	4	0	2570	2,5	122	80	91	80	79	43	6,3
2424	0	0	0	4	0	2795	2,5	113	73	84	102	78	45	7,9
2425	0	0	0	4	0	2795	2,5	122	78	90	111	74	44	8,3
2426	0	0	0	4	1	2065	2,5	111	75	85	102	69		7,1
2427	0	0	0	0	0	2725	2,5	118	86	96	85	74	43	6,3
2428	0	0	0	0	0	2725	2,5	121	81	91	94	67	41	6,3
2429	0	0	0	0	1	2665	2,5	119	78	88	113	66	38	7,4
2430	0	0	0	0	1	2665	2,5	100	73	81	107	64	37	6,8
2431	0	0	0	0	0	2725	2,5	125	83	93	91	71	43	6,5
2432	0	0	0	0	0	2555	2,5	108	75	85	90	67	43	6
2433	0	0	0	0	0	2555	2,5	115	77	88	110	53	33	5,8
2434	0	0	0	0	0	2560	2,5	93	67	75	76	64		4,9
2435	0	0	0	0	0	2790	2,5	122	78	88	100	71	41	7,1
2436	0	0	0	0	0	2790	2,5	121	80	90	107	59	36	6,3
2437	0	0	0	0	1	2240	2,5	103	72	79	118	74	38	8,7
2438	0	0	0	0	1	2240	2,5	127	79	90	106	84	43	8,9
2439	0	0	0	0	1	2440	2,5	108	71	81	91	68	40	6,2
2440	0	0	0	0	1	2440	2,5	107	71	81	89	58	35	5,2
2441	0	0	0	0	1	2545	2,5	113	71	81	102	52	33	5,4

2442	0	0	0	0	1	2545	2,5	122	69	81	79	77	47	6,1
2443	0	0	0	0	1	2395	2,5	107	70	79	89	68	39	6
2444	0	0	0	0	1	2395	2,5	124	83	94	80	79	46	6,2
2445	0	0	0	0	0	1425	2,5	130	89	101	108	79		8,5
2446	0	0	0	0	0	2650	2,5	100	71	79	104	53	35	5,5
2447	0	0	0	0	0	2650	2,5	100	71	80	102	43	29	4,4
2448	0	0	0	0	0	2550	2,5	103	74	83	101	70	43	7
2449	0	0	0	0	0	2550	2,5	99	70	79	91	86	52	7,9
2450	0	0	0	0	0	2890	2,5	119	79	90	94	100	57	9,4
2451	0	0	0	0	0	2890	2,5	98	65	74	97	90	53	8,8
2452	0	0	0	0	0	2875	2,5	119	80	91	93	66	40	6,1
2453	0	0	0	0	0	2875	2,5	122	76	87	95	90	53	8,5
2454	0	0		0	1	2170	2,5	153	99	113	119	52		6,2
2455	0	0		0	1	717	2,5	128	83	96	100	82		8,2
2456	0	0		1	0	1460	2,5	108	73	84	89	72		6,3
2457	0	0			0	2040	0	132	93	104	94	81	42	7,6
2458	0	0			1	80	0	115	70	80	66	96	56	6,3
2459	0	0			0	1716	0	110	79	88	115	44	28	4,9
2460	0	0			0	2125	0	125	74	83	93	104	54	9,6
2461	0	0	0	2	1	530	0	132	78	89	90	90	49	8,1
2462	0	0	0	2	1	530	0	109	69	80	77	103	56	7,9

ID Code	CI-st	Gestat asses	TBW	ECW%	ECW	ICW	ECW/ICW	BSA
1	3,7	36,86	35,85	44,85	16,08	19,77	0,813	1,791
2	4,4	39,86	38,76	43,85	17	21,76	0,781	2,127
3	3,5	33						
4	3,6	28,72						
5	4,2	32,28	36,65	45,94	16,84	19,81	0,85	1,829
6	4,8	31,14	30,52	39,67	12,11	18,41	0,657	1,823
7	3,9	30						
8	3,6	31,72	28,21	43,63	12,31	15,9	0,774	1,514
9	5,2	27,18	36,01	43,23	15,57	20,44	0,761	1,944
10	4,3	32,43	30,63	41,49	12,71	17,92	0,709	1,7
11	3,7	30,43	29,37	43,37	12,74	16,63	0,766	1,639
12	3,6	20,43						
13		38,43						
14	4,4	33	30,27	42,64	12,91	17,36	0,743	1,629
15	4,6	20,72	33,75	43,64	14,73	19,02	0,774	1,748
16	5	11,72	36,18	44,52	16,11	20,07	0,802	1,645
17	3,3	12,43	35,58	44,71	15,91	19,67	0,808	1,762
18	4,8	19,28	35,08	40,3	14,14	20,94	0,675	2,107
19	4,4	11,28	35,36	40,1	14,18	21,18	0,669	2,141
20	4,7	19,86	27,47	42,55	11,69	15,78	0,74	1,417
21	3,6	13,43	24,93	40,67	10,14	14,79	0,685	1,371
22		12						
23	3,9	12,43	31,32	43,42	13,6	17,72	0,767	1,788
24	4,5	13	31,01	42,53	13,19	17,82	0,74	1,808
25	4,1	21						
26	4,5	20,28	34,58	46,41	16,05	18,53	0,866	1,843
27	4	13,14	33,99	45,89	15,6	18,39	0,848	1,848
28	4	12	33,14	44,41	14,72	18,42	0,799	1,668
29	3,9	20	33,76	44,07	14,88	18,88	0,788	1,723
30	3,9	21,14	39,4	43,75	17,24	22,16	0,777	2,124
31	4,3	12,57	35,96	41,54	14,94	21,02	0,71	2,059
32		20,43	33,42	44,37	14,83	18,59	0,797	1,809

33	4,9	11,57	34,38	44,79	15,4	18,98	0,811	1,74
34		32,86						
35		37,28						
36		33,14						
37		37,43						
38	4,4	20	35,81	43,08	15,43	20,38	0,757	1,92
39	4,7	11,43	35,31	43,35	15,31	20	0,765	1,845
40	4,2	34,86	39,9	46,59	18,59	21,31	0,872	2,019
41	3,5	27,86						
42	3,4	36,28	34,48	45,38	15,65	18,83	0,831	1,699
43	3,9	31	41,89	45,95	19,25	22,64	0,85	2,042
44	4	30,43	28,83	40,92	11,8	17,03	0,692	1,567
45	4,7	30,43	34,06	44,68	15,22	18,84	0,807	1,782
46	4,1	33,72	34,67	42,97	14,9	19,77	0,753	1,88
47	5,1	33,57						
48	5	29,28						
49	4,1	19,57	32,09	43,59	13,99	18,1	0,772	1,725
50		11,72						
51	3,1	39,28						
52	3,5	33,28	37,09	46,29	17,17	19,92	0,961	1,858
53	3,7	31						
54	3,3	31,86	27,83	45,92	12,78	15,05	0,849	1,382
55	3,5	36,72						
56	3,4	38,43	43,83	46,38	20,33	23,5	0,865	2,121
57	5,8	20,72	40,19	44,21	17,77	22,42	0,792	2,05
58	3,3	30,72	29,57	42,74	12,64	16,93	0,746	1,549
59	4,1	31,72						
60	3,4	33,14	36,86	45,6	16,81	20,05	0,838	1,909
61	4,4	23,72	35,21	43,19	15,21	20	0,76	1,831
62	5,3	12,43	40,27	44,35	17,86	22,41	0,796	2,062
63		11						
64	3,7	21,86						
65	5,9	34,72						

66	3,8	28,14	33,72	43,35	14,62	19,1	0,765	1,688
67	4,4	30,57						
68	4,1	22,72	32,29	41,87	13,52	18,77	0,72	1,712
69	4,1	36,72	36,41	43,47	15,83	20,58	0,769	1,794
70	4,7	36,28	34,92	43,52	15,2	19,72	0,77	2,031
71	3,6	34	36,83	45,88	16,9	19,93	0,847	1,807
72	3,2	37,28						
73	4,6	26,57	32,03	43,74	14,01	18,02	0,777	1,691
74	4,2	40						
75	2,8	34,43	27,57	40,04	11,04	16,53	0,667	1,563
76	3,5	31,86	40,64	46,62	18,95	21,69	0,873	2,16
77	4,1	30	34,26	43,63	14,95	19,31	0,774	1,765
78	6,3	33	39,39	43,79	17,25	22,14	0,779	2,04
79	4,5	34,14						
80	4,1	13,43	35,54	43,78	15,56	19,98	0,778	1,78
81	4	25,14						
82	4,1	19,86	35,13	43,12	15,15	19,98	0,758	1,807
83	3,9	40,43						
84	3,7	20,86	41,21	44,84	18,48	22,73	0,813	2,13
85	3,9	12	40,06	44	17,63	22,43	0,786	2,14
86	4,6	28,86	35,97	44,28	15,93	20,04	0,794	1,892
87	4,9	30,28	31,64	41,52	13,14	18,5	0,71	1,741
88	4,6	33,72	31,14	39,21	12,21	18,93	0,645	1,788
89	4,5	34,72						
90	4,4	21,72						
91	5,1	29,28						
92	4,4	21,28						
93	4	41,14						
94	4,3	20,86	39,5	45,51	17,98	21,52	0,835	2,008
95	4,2	30,14	32,18	44,34	14,27	17,91	0,796	1,772
96	3,5	38,28						
97		37,43	46,77	48,85	22,85	23,92	0,955	2,224
98	4,1	39,14						

99	4,4	30,72	33,6	43,24	14,53	19,07	0,761	1,741
100	4,3	36,72	39,55	44,98	17,79	21,76	0,817	2,068
101	4,7	20,86	33,58	41,45	13,92	19,66	0,708	1,822
102	5,7	32,43	38,01	44,98	17,1	20,91	0,817	1,931
103	4,3	39						
104	3,7	35,43						
105	3	13,28	27,79	40,05	11,13	16,66	0,668	1,476
106	3,2	12,14	25,11	38,98	9,79	15,32	0,639	1,429
107	3,3	21,86	27,56	42,52	11,72	15,84	0,739	1,485
108	3,6	39	34,25	44,17	15,13	19,12	0,791	1,695
109	5	24,28	77,02	45,24	16,75	20,27	0,826	2,108
110	4,3	36,28	37,61	44,77	16,84	20,77	0,81	2,19
111	4,9	21	33,62	44,52	14,97	18,65	0,802	1,673
112	4	13,86	31,67	42,87	13,58	18,09	0,75	1,624
113	4,9	30,72						
114	4,1	25,43	35,43	43,63	15,46	19,97	0,774	1,791
115	4,1	29,72	31,74	43,69	13,87	17,87	0,776	1,7
116	3,7	30	35,15	45,37	15,95	19,2	0,83	1,862
117	3,6	23,57	35,91	45,22	16,24	19,67	0,825	1,776
118	4,7	35,14	44,15	47,74	21,08	23,07	0,913	2,053
119	3,8	29	37,7	44,29	16,7	21	0,795	2,014
120	5,5	32,28	37,96	46,91	17,81	20,15	0,883	2,003
121	4,5	32,57	33,71	42,8	14,43	19,28	0,748	1,84
122	4,1	21,57	41,01	43,79	17,96	23,05	0,779	2,238
123	4,5	31,72						
124	5	20,57	37,01	45,25	16,75	20,26	0,826	1,955
125		14,28						
126	4,2	29						
127	4,5	13						
128	4,4	21,14						
129	3,7	36,43						
130	3,5	12,57	33,89	41,45	14,05	19,84	0,708	1,918
131		12,86						

132	4,2	20,43	34,27	41,23	14,13	20,14	0,701	1,93
133	4,2	31,72	36,15	44,78	16,19	19,96	0,811	1,92
134	3,9	33,86						
135	4,7	37,43	34,38	45,81	15,75	18,63	0,845	1,683
136	3,7	37,43	43,77	48,34	21,16	22,61	0,935	2,091
137	4,4	33,14						
138	5,2	20,28	34,28	44,07	15,11	19,17	0,788	1,825
139	5,1	33,72	41,82	46,07	19,27	22,55	0,854	2,079
140	4	32,14	37,14	45,23	16,8	20,34	0,825	1,902
141	3,9	39,14						
142	4,4	25,57						
143	6,3	32,14	39,18	45,02	17,64	21,54	0,818	2,062
144	4,2	12,28	33,92	43,45	14,74	19,18	0,768	1,809
145	5,1	20,28	35,34	44,51	15,73	19,61	0,802	1,83
146		12,72						
147	5,2	32,28	34,87	42,58	14,85	20,02	0,741	1,897
148	5,6	34,72						
149	4,9	33,28	51,86	49,01	25,42	26,44	0,961	2,423
150	4,1	19,72						
151	4,8	40						
152	4,2	34,14	36,99	43,11	15,95	21,04	0,758	2,061
153	4,5	25	31,54	44,13	13,92	17,62	0,79	1,752
154	4,2	24	37,24	43,55	16,22	21,02	0,771	2,04
155	3,8	29,86	33,59	45,84	15,4	18,19	0,846	1,642
156	5,3	29,57	35,81	42,39	15,18	20,63	0,735	1,991
157		12,28						
158	4,5	21	28,93	42,37	12,26	16,67	0,735	1,567
159	4,2	13	28,11	41,51	11,67	16,44	0,709	1,529
160	4,7	13,43	32,11	42,22	13,56	18,55	0,73	1,715
161	4,7	18,86	33,95	43,32	14,71	19,24	0,764	1,741
162	3,7	20,14	36,53	42,78	15,63	20,9	0,747	1,938
163	3,9	12,28	35,77	42,4	15,17	20,6	0,736	1,886
164	5,3	20	39,32	46,41	18,25	21,07	0,866	2,056

165	4,6	11,57	38,29	44,99	17,23	21,06	0,818	2,048
166	3,4	28,72	35,69	43,79	15,63	20,06	0,779	2,003
167	3,4	20	34,62	43,67	15,12	19,5	0,775	1,942
168		11,72						
169	4,4	34,14						
170	4,8	20,14	30,91	42,89	13,26	17,65	0,751	1,577
171	3,5	12,86	29,93	41,95	12,45	17,48	0,712	1,534
172	4,3	25,43	36,55	43,55	15,92	20,63	0,771	1,909
173	4,6	18,86	37,43	44,24	16,56	20,87	0,793	1,956
174	4,9	25,28	34,42	46,07	15,86	18,56	0,854	1,73
175		13,14						
176		35,14						
177	4,3	25,86	29,1	43,6	12,69	16,41	0,773	1,604
178	4,5	13,14	33,15	40,87	13,55	19,6	0,691	1,778
179	4,4	30	34,9	41,77	14,58	20,32	0,72	
180	4,4	20	33,46	42,13	14,1	19,36	0,73	
181	4,2	30	32,97	43,88	14,47	18,5	0,782	1,901
182	4,1	15,43	33,88	44,71	15,15	18,73	0,808	1,722
183	4,2	26,57	29,14	41	11,95	17,19	0,695	1,618
184	4	36						
185	4,5	29,86						
186	4,4	27,57	40,36	46,38	18,72	21,64	0,865	2,021
187	4,1	34,86						
188	4,2	15,43	37,95	44,95	17,06	20,89	0,816	2,064
189	5,6	32,86	34,91	42,45	14,82	20,09	0,737	1,895
190	4,2	26,86						
191	3,2	33,43						
192	3	40,72	36,19	45,17	16,35	19,84	0,824	1,898
193		11,57						
194		11						
195	4,2	29,86						
196	3,6	11,86	28,13	41,69	11,73	16,4	0,715	1,505
197	4,6	20,43	28,78	42,18	12,14	16,64	0,729	1,548

198		32,28						
199	5,9	29,57	38,67	45,35	17,54	21,13	0,83	1,929
200	4,8	27,57						
201	4,3	33,43	35,43	42,95	15,22	20,21	0,753	1,924
202	3,7	19,86	33,93	42,29	14,35	19,58	0,732	1,823
203	3,8	12,86	33,57	42,18	14,16	19,41	0,729	1,783
204	3,4	32,43	47,67	49,25	23,48	24,19	0,97	2,224
205	4,3	27,57						
206		20,43	41,43	45,73	18,95	22,48	0,842	2,164
207	4,9	13	40,7	44,32	18,04	22,66	0,796	2,153
208	4,3	26,57	39,09	44,48	17,39	21,7	0,801	2,096
209	4,8	28,72	39,03	46,09	17,99	21,04	0,855	2,043
210	3,7	37,86	34,94	43,04	15,04	19,9	0,755	1,864
211	4,7	36,14						
212	4	28,28	37,71	44,41	16,75	20,96	0,799	2,003
213	3,3	31	33,25	44,75	14,88	18,37	0,81	1,781
214	4,3	26	32,54	44,74	14,56	17,98	0,809	1,766
215	4,9	14	39,97	45,55	18,21	21,76	0,836	2,097
216	5,9	20,28	41,84	46,41	19,42	22,42	0,866	2,151
217	3,7	33,28						
218	4,8	37,14	35,57	44,75	15,92	19,65	0,81	1,879
219	4,5	12,14	31,21	41,49	12,95	18,26	0,709	1,692
220	4,1	18,86	31,58	42,43	13,4	18,18	0,737	1,741
221	3,9	32,14						
222	3,6	34,14	43,16	45,34	19,57	23,59	0,829	2,263
223	5,7	25	35,31	44,29	15,64	19,67	0,795	1,795
224	3,9	34,72						
225	4,5	38,72						
226	3,6	34,86	34,43	42,89	14,77	10,66	0,751	1,77
227	4	35						
228	2,8	38,28	43,75	49,32	21,58	22,17	0,973	2,192
229	4,4	34						
230	4,8	24,14						

231	6,3	30,86	41,44	42,8	17,74	23,7	0,748	2,537
232	5,8	10,86	40,54	43,61	17,68	22,86	0,773	2,275
233	5,3	25,86	39,73	42,81	17,01	22,72	0,748	2,316
234		12,86						
235	4	32	55,23	58,15	32,12	23,11	1,389	2,235
236	5,9	31,72	44,54	47,21	21,03	23,51	0,894	2,203
237	4,3	20,43	49,61	46,92	23,28	26,33	0,884	2,589
238	3,7	12,43	52,27	44,21	23,11	29,16	0,792	2,612
239		11,57						
240	4,6	28	36,81	45,63	16,8	20,01	0,839	2,026
241	4,4	35,57	31,29	42,98	13,45	17,84	0,753	1,68
242	3,8	22	42,35	44,39	18,8	23,55	0,798	2,278
243	3,3	12	38,52	44,75	17,24	21,28	0,81	2,097
244	4,5	32,43						
245	3,8	27,43						
246	3,6	35,28						
247	5,5	35	42,48	47,59	20,22	22,26	0,908	2,055
248	5,3	23,43						
249	6	21,72	36,73	43,3	15,87	20,86	0,76	2,015
250	3,1	34,86	42,33	48,68	20,61	21,72	0,948	2,111
251	4,5	19,72	32,68	43,6	14,25	18,43	0,773	1,697
252	3,9	11,28	37,87	43,59	16,51	21,36	0,772	1,67
253	4,5	19,72	34,59	43,01	14,88	19,71	0,754	1,802
254	4,1	11,72	34,25	42,91	14,7	19,55	0,751	1,721
255	4,8	20,57	31,74	41,49	13,17	18,57	0,709	1,683
256	4	10,43	30,39	39,84	12,11	18,28	0,662	1,649
257	4,3	13,14	32,18	43,84	14,11	18,07	0,78	1,736
258	4,1	13	36,6	45,24	16,56	20,04	0,826	2,013
259	4,8	23,57						
260	3,8	32,28						
261	3,7	20,43						
262	4,7	23,72	34,95	45,89	16,04	18,91	0,848	1,732
263	4,3	21,14	34,63	42,7	14,78	19,85	0,744	1,932

264	3,6	12,86	35,16	43,65	15,35	19,81	0,774	1,92
265	3,9	11	38,17	44,53	17	21,17	0,803	2,044
266	3,9	21,28	32,81	43,58	14,3	18,51	0,772	1,7
267	3,5	12,43	32,38	42,8	13,86	18,52	0,748	1,68
268	4,1	12,57	37,7	46,89	17,68	20,02	0,883	1,834
269	4,4	19,72	38,52	47,09	18,14	20,38	0,89	1,859
270	3,6	20,57	33,39	45,37	15,15	18,24	0,83	1,678
271	3,7	11,72	30,33	42,49	12,89	17,44	0,739	1,609
272	3,8	21,72	34,19	42,81	14,64	19,55	0,748	1,876
273	3,9	12	32,77	42,35	13,88	18,89	0,734	1,812
274	4,7	20,72	30,2	43,9	13,26	16,94	0,782	1,577
275	4,2	12,14	28,85	43,05	12,42	16,43	0,755	1,52
276	3,5	20,28	33,6	43,24	14,53	19,07	0,761	1,861
277	4,1	12,28	32,85	43,65	14,34	18,51	0,774	1,763
278	5,2	20,43	39,39	46,58	18,35	21,04	0,872	1,967
279	4,7	12,28	35,75	44	15,73	20,02	0,785	1,881
280	4,7	12,28	37,33	45,19	16,87	20,46	0,824	1,881
281	5,9	20	38,61	45,63	17,62	20,99	0,839	1,955
282	3	21,28	40,41	46,69	18,87	21,54	0,876	2,228
283	3,5	12,28	37,94	45,54	17,28	20,66	0,836	2,125
284	4,3	22,14	34,37	42,24	14,52	19,85	0,731	1,926
285	4,9	11,57	32,87	41,8	13,74	19,13	0,718	1,852
286	3,9	12,28	38,5	43,81	16,87	21,63	0,779	1,883
287	4,2	20,28	39,56	43,85	17,35	22,21	0,781	1,903
288	3,7	20,43	35,11	46,93	16,48	18,63	0,884	1,745
289	3,7	13,57	34,51	47,2	16,29	18,22	0,894	1,712
290	4	20,72	35,99	43,73	15,74	20,25	0,777	1,997
291	3,8	11,57	33,45	42,18	14,11	19,34	0,729	1,926
292	3,7	12	32,08	43,7	14,02	18,06	0,776	1,67
293	4,6	20						
294	4,9	19,72	37,78	45,57	17,22	20,56	0,837	1,883
295	4,6	11,72	34,8	43,47	15,13	19,67	0,769	1,833
296	4,2	11,57	32,85	42,49	13,96	18,89	0,739	1,798

297	5	19,14	33,63	43,14	14,51	19,12	0,758	1,86
298	3,6	19,86	26,9	39,7	10,68	16,22	0,658	1,549
299	3,9	12,28	24,85	38,14	9,48	15,37	0,616	1,489
300	6,6	12,14	38,83	43,34	16,83	22	0,765	2,091
301	4,8	20	39,28	43,45	17,07	22,21	0,768	2,103
302	5,1	20,14	29,95	40,06	12	17,95	0,668	1,685
303	4,6	12,14	29,5	40,03	11,81	17,69	0,667	1,672
304	4,2	20,86	33,97	43,62	14,82	19,15	0,773	1,736
305	4,5	12,86	32,43	42,79	13,88	18,55	0,748	1,668
306	4	12	36	43,33	15,6	20,4	0,764	1,812
307	4,9	20	36,76	44,45	16,34	20,42	0,8	1,865
308	4,8	14,43	34,39	44,75	15,39	19	0,81	1,776
309	3,8	21,28	35,45	43,75	15,51	19,94	0,777	1,786
310	3,9	12,14	34,08	42,84	14,6	19,48	0,749	1,692
311	3,9	11,57	29,81	42,53	12,68	17,13	0,74	1,687
312	4,4	19,28	30,63	43,02	13,18	17,45	0,755	1,745
313	4,2	11,86	31,14	41,84	13,03	18,11	0,719	1,762
314	4,9	21,43						
315	3,5	20,14	32,62	42,61	13,9	18,72	0,742	1,829
316	4	12	31,47	42	13,22	18,25	0,724	1,778
317	3,7	12	37,43	45,15	16,9	20,53	0,823	1,877
318	3,7	21	38,8	46,23	17,94	20,86	0,86	1,889
319	4,2	13,72	43,11	46,27	19,95	23,16	0,861	2,318
320	3,7	18,28	43,16	46,58	20,1	23,05	0,872	2,318
321	3,8	11,28	32,29	42,55	13,74	18,55	0,74	1,647
322	4,5	20,57	30,19	42,19	12,74	17,45	0,73	1,58
323	4,1	11,57	30,54	42,43	12,96	17,58	0,737	1,552
324	4,7	20,72	38,53	43,83	16,89	21,64	0,78	2,015
325	3,8	10,72	36,98	42,88	15,86	21,12	0,75	1,979
326	4,9	13,14	35,87	44,68	16,03	19,84	0,807	1,8
327	4,5	20,28	34,66	44,14	15,3	19,36	0,79	1,768
328	3,9	13,43	33,85	43,92	14,87	18,98	0,783	1,703
329	3,4	12,43	31,91	41,89	13,37	18,54	0,721	1,673

330	5	21,43	31,07	44,54	13,84	17,23	0,803	1,652
331	3,8	12,72	30,9	44,3	13,69	17,21	0,795	1,593
332	5	30,86	33,53	44,61	14,96	18,57	0,805	1,811
333	3,5	12,14	29,99	42,28	12,68	17,31	0,732	1,644
334	6,3	20,14	37,39	44,15	16,51	20,88	0,79	1,985
335	4,9	12	38,49	45,44	17,49	21	0,832	1,985
336	4,1	20,14	34,56	44,87	15,51	19,05	0,814	1,776
337	4,2	11,57	34,01	43,98	14,96	19,05	0,785	1,776
338	4,1	12,14						
339	3,6	12,28	35,97	44,78	16,11	19,86	0,811	1,762
340	3,9	20,28	37,23	46,03	17,14	20,09	0,853	1,841
341	5,1	21,28	34,28	44,25	15,17	19,11	0,793	1,811
342	4,5	11,72	34,44	44,54	15,34	19,1	0,803	1,785
343	3,6	14,43	31,4	40,7	12,78	18,62	0,686	1,674
344	4,1	19,57	32,64	41,54	13,56	19,08	0,71	1,707
345	3,8	20,14	33,67	44,43	14,96	18,71	0,799	1,712
346	3,7	12,14	32,48	44,08	14,32	18,16	0,788	1,644
347	3,6	20,43						
348	3,6	12,86	35,28	42,65	15,05	20,23	0,743	1,839
349	3,7	11,57	32,75	42,59	13,95	18,8	0,742	1,702
350	4,4	19	31,62	41,99	13,28	18,34	0,724	1,72
351	3,9	13,28	31,46	40,94	12,88	18,58	0,693	1,697
352	4,5	20,28	32,69	42,45	13,88	18,81	0,737	1,724
353	3,6	12,43	29,04	41,25	11,98	17,06	0,702	1,611
354	4,2	19,28	29,42	41,12	12,1	17,32	0,698	1,611
355	3,8	20,28	29,62	42,8	12,68	16,94	0,748	1,526
356	2,9	13,28	28,18	41,8	11,78	16,4	0,718	1,501
357		13						
358		11,86						
359		10						
360		8,14						
361		12,43						
362		12						

363		12,43						
364		13,14						
365		12						
366		12,57						
367		11						
368		12,72						
369		11,57						
370		13,43						
371		13,72						
372		12						
373		12						
374		12,86						
375		12,28						
376		12,14						
377		12,72						
378		12						
379		11,14						
380		12,43						
381		11,86						
382		12,28						
383		11,28						
384		12,43						
385	4,3	20,28	32,31	43,57	14,08	18,23	0,772	1,602
386	3,5	12,14	35,24	44,49	15,68	19,56	0,801	1,557
387	4,7	21,28	36,34	46,28	16,82	19,52	0,861	1,811
388	4	12,57	33,68	44,77	15,08	18,6	0,81	1,76
389	3,8	12	33,68	42,33	14,26	19,42	0,734	1,775
390	3,9	20,72	34,96	42,73	14,94	20,02	0,746	1,789
391	5,2	20,28	36,55	46,07	16,84	19,71	0,854	1,878
392	4,7	12,86	36,36	45,43	16,52	19,84	0,832	1,822
393	3,9	20,28	30,71	43,63	13,4	17,31	0,774	1,612
394	3,8	12,28	30,44	43,79	13,33	17,11	0,779	1,585
395	3,9	13,14	35,65	44,57	15,89	19,76	0,804	1,838

396	4,3	20,14	37,14	46,17	17,15	19,99	0,857	1,82
397	3,9	19,86	32,55	43,62	14,2	18,35	0,773	1,844
398	4,3	12,86	32,67	42,82	13,99	18,68	0,748	1,82
399	4,2	12,43	33,13	44	14,58	18,55	0,785	1,733
400	4,3	20,43	34,64	45,09	15,62	19,02	0,821	1,798
401	4,2	12,43	43,03	48,08	20,69	22,34	0,926	2,106
402	5	19,57	40,94	47,19	19,32	21,62	0,893	2,067
403	4,9	11,86	38,58	46,24	17,84	20,74	0,86	1,979
404	5,2	14,14	34,93	43,88	15,33	19,6	0,782	1,751
405		11,43						
406		11,57						
407		12,72						
408		13,14						
409		13						
410		12,57						
411		12,43						
412		12,14						
413		9,86						
414		12,43						
415		12,14						
416		11,86						
417	4,1	37,14						
418	4,6	20	28,56	42,43	12,12	16,44	0,737	1,566
419	3,9	12	28,5	43,22	12,32	16,18	0,761	1,51
420		12,14						
421	4,6	19,57	29,16	40,53	11,82	17,34	0,681	1,678
422	4,3	11,72	29,3	40,71	11,93	17,37	0,686	1,658
423		12,86						
424	4,1	12,14	31,26	41,17	12,87	18,39	0,699	1,656
425		19,57	31,22	41,57	12,98	18,24	0,711	1,69
426		12						
427		11,57						
428		12,14						

429		12						
430	5,2	11,43	35,63	43,3	15,43	20,2	0,763	1,909
431	5,5	19	36,1	43,43	15,68	20,42	0,767	1,92
432		12,14						
433	4,5	20,14	37,01	45,42	16,81	20,2	0,832	1,812
434	4,6	11,72						
435		12						
436		12						
437		13,14						
438	5,4	20,28	39,59	45,94	18,19	21,4	0,85	2,061
439	5	12,57	35,42	44,38	15,72	19,7	0,797	1,948
440	3,7	12,14	38,58	45,87	17,7	20,88	0,847	2,261
441	5,7	20,28	39,98	47,92	19,16	20,82	0,92	2,271
442	4,4	12,43	36,1	43,43	15,68	20,42	0,767	1,828
443	4,4	20,72	36,75	43,29	15,91	20,84	0,763	1,907
444	3,4	20,28	31,17	43,37	13,52	17,65	0,766	1,727
445	3,3	11,43	29,67	41,35	12,33	17,34	0,711	1,662
446	4,7	12,72	46,67	44,54	20,79	25,88	0,803	2,37
447	4,3	20,72	45	47,02	21,16	23,84	0,887	2,4
448		12,28						
449	4,6	13,43	33,11	42,61	14,11	19	0,742	1,86
450	5	20,14	31,07	42,42	13,18	17,89	0,736	1,733
451	3,9	12,14	29,44	40,89	12,04	17,4	0,691	1,66
452		11,43						
453	5,7	18,86	33,86	45,15	15,29	18,57	0,823	1,738
454	4,2	12,28	32,63	43,57	14,22	18,41	0,772	1,712
455	4,9	19,86	30,7	39,9	12,25	18,45	0,663	1,861
456	4,4	11,43	30,92	40,07	12,39	18,53	0,668	1,831
457	5,2	13,86	38,25	44,39	16,98	21,27	0,798	2,044
458		11,72						
459	4,5	20,72	33,72	42,58	14,36	19,36	0,741	1,76
460	3,8	11,72	31,89	41,61	13,27	18,62	0,712	1,678
461	4,1	20,43						

462	4	13,57	32,03	43,64	13,98	18,05	0,774	1,686
463	4,1	20,28	31,32	43,64	13,67	17,65	0,774	1,632
464	3,5	10,86	30,43	40,91	12,45	17,98	0,692	1,505
465	5,4	12,43	39,01	44,55	17,38	21,63	0,803	2,032
466	5,2	18,72	41,8	46,22	19,32	22,48	0,859	2,068
467		11,56	41,96	44,51	18,68	23,28	0,802	2,16
468	4,7	20,14	38,45	45,64	17,55	20,9	0,839	1,852
469	3,7	12,14	36,88	44,95	16,58	20,3	0,816	1,813
470	4,1	12	34,01	42,78	14,55	19,46	0,747	1,823
471	4,8	19,86	33,63	42,69	14,36	19,27	0,745	1,862
472	4,6	19,86	29,38	40,02	11,76	17,62	0,667	1,626
473	3,8	12,28	29,41	40,15	11,81	17,6	0,671	1,582
474	2,9	12,14	28,4	41,33	11,74	16,66	0,704	1,563
475	3,3	20,14	29,99	42,68	12,8	17,19	0,744	1,648
476	4	11,43	28,15	41,98	11,82	16,33	0,723	1,475
477	3,9	12	33,74	44,72	15,09	18,65	0,809	1,826
478	4,6	20	34,68	45,47	15,77	18,91	0,833	1,861
479	3,9	19,43	28,27	41,77	11,81	16,46	0,717	1,49
480		11,86						
481	4,7	20,43	29,77	41,48	12,35	17,42	0,708	1,549
482	4	12,43	35,65	44,54	15,88	19,77	0,803	1,628
483	4,2	12,28	29,61	42,99	12,73	16,88	0,754	1,49
484		12,28						
485	3,8	11,57	28,44	40,47	11,51	16,93	0,679	1,578
486		11,14						
487	4,4	20,57	29,76	41,8	12,44	17,32	0,718	1,606
488	3,8	12,72	32,6	44,53	14,52	18,08	0,803	1,49
489	4,1	20,86	33,35	45,27	15,1	18,25	0,827	1,527
490	4,5	11,86						
491	5,1	19,86	35,2	42,99	15,13	20,07	0,753	1,866
492	4,8	18,28	32,59	41,91	13,66	18,93	0,721	1,831
493	4,3	12,57	32	41,21	13,19	18,81	0,701	1,805
494	4,3	19,57	29,71	42,37	12,59	17,12	0,735	1,581

495	4,6	20	33,25	44,27	14,72	18,53	0,794	1,767
496	3,8	12,14	30,8	40,87	12,59	18,21	0,691	1,64
497		21,86	34,23	43,55	14,91	19,32	0,771	1,765
498	3,8	13,72	28,38	41,22	11,7	11,68	0,701	1,539
499	4,6	21,14	31,62	41,42	13,1	18,52	0,707	1,668
500	4,3	12	32,02	43,59	13,96	18,06	0,772	1,728
501	3,7	11,86	33,33	43,53	14,51	18,82	0,77	1,692
502	4,5	11,28	30,48	43,33	13,21	17,27	0,764	1,558
503	4,5	12,43	32,22	43,32	13,96	18,26	0,764	1,76
504	4,1	20,43	32,57	43,75	14,25	18,32	0,777	1,772
505	5,5	20,28	29,45	42,3	12,46	16,99	0,733	1,586
506	3,3	12	31,93	44,22	14,12	17,81	0,792	1,646
507	3,1	20,57	31,68	43,87	13,9	17,78	0,781	1,674
508	4	20,14	32,71	42,03	13,75	18,96	0,725	1,7
509	3,6	12,14	31,68	41,28	13,08	18,6	0,703	1,7
510		12,28						
511	4,1	13,14	41,4	46,49	19,25	22,15	0,869	1,876
512	4,7	19,14	42,46	46,6	19,79	22,67	0,872	1,927
513	3,6	11,43	33,76	42,92	14,49	19,27	0,751	1,819
514	3,8	20,28	34,4	44,27	15,23	19,17	0,794	1,794
515	5	12,86	36,33	43,79	15,91	20,42	0,779	1,871
516	4,9	19,86	38,49	45,85	17,65	20,84	0,846	1,902
517		13,14						
518	4,3	12	34,55	42,92	14,83	19,72	0,752	1,802
519	3,8	19,43	32,79	42,93	14,08	18,71	0,752	1,845
520	4,1	12,43	33,63	43,5	14,63	19	0,77	1,833
521	3,8	12,43	31,74	43,25	13,73	18,01	0,762	1,586
522	4,9	19,14	31,35	43,03	13,49	17,86	0,755	1,627
523	4,4	11,57	31,5	42,47	13,38	18,12	0,738	1,632
524	4,5	19,57	32,68	43,17	14,11	18,57	0,759	1,659
525	4	11,72	28,4	40,77	11,58	16,82	0,688	1,563
526	4,8	20,72	34,56	42,59	14,72	19,84	0,741	1,834
527		13,72						

528	4,4	20,72	30,12	41,69	12,56	17,56	0,715	1,699
529	5	12,14	32,78	41,48	13,6	19,18	0,709	1,767
530	4,5	12,28	29,97	41,4	12,41	17,56	0,706	1,661
531	3,5	20,14	30,84	43,32	13,36	17,48	0,764	1,544
532	5,2	20,28	30,75	41,82	12,86	17,89	0,718	1,675
533	3,2	11,43	29,54	41,53	12,27	17,27	0,71	1,537
534	4,9	12	32,48	39,85	12,94	19,54	0,662	1,741
535	3,9	12	36,88	44,73	16,5	20,38	0,809	1,979
536	4,4	20,14	36,99	44,09	16,31	20,68	0,788	1,997
537		11,86						
538	3,9	12	31,54	41,34	13,04	18,5	0,704	1,673
539		12,14						
540	4,8	21,43						
541		12,57						
542		12,43						
543	4,7	20,28	31,43	43,07	13,54	17,89	0,756	1,649
544	4,1	12,43	30,57	42,55	13,01	17,56	0,74	1,587
545	4,1	13,28	28,38	42,14	11,96	16,42	0,728	1,552
546	4	20,28	28,82	42,15	12,15	16,67	0,728	1,607
547		13,14						
548		11,43						
549		12,57						
550	4,1	20	35,56	43,41	15,44	20,12	0,767	1,778
551		13,57						
552	4,2	12	34,59	43,07	14,9	19,69	0,756	1,731
553	3,4	12,43	37,93	44,1	16,73	21,2	0,789	2,026
554	4,1	21,43	40,08	45,5	18,24	21,84	0,835	2,05
555	4,4	12,43	31,53	41,19	12,99	18,54	0,7	1,723
556	5	20,43	33,93	43,32	14,7	19,23	0,764	1,789
557		12,28						
558	3,2	11	33,56	42,22	14,17	19,39	0,73	1,721
559	4	11,57	32,23	42,41	13,67	18,56	0,736	1,673
560	4,5	20,43	32,34	42,14	13,63	18,71	0,728	1,728

561	4,5	12,43	30,3	40,23	12,19	18,11	0,673	1,738
562	5,6	20,14	31,59	42,1	13,3	18,29	0,727	1,763
563	5	20,86	39,46	44,24	17,46	22	0,793	2,094
564	4,5	12,28						
565	4,6	19,86	29,86	40,99	12,24	17,62	0,694	1,616
566	5	18,43	37,67	45,07	16,98	20,69	0,82	1,854
567	3,9	11,86	29,9	41,27	12,34	17,56	0,702	1,587
568	4,1	13,28	37,95	45,21	17,16	20,79	0,825	1,854
569		12,43						
570	5,8	20,43	36,01	43,79	15,77	20,24	0,779	1,903
571		11,43						
572	5,2	13	33,54	42,03	14,1	19,44	0,726	1,842
573		11						
574	3,3	12,28	26,97	39,85	10,75	16,22	0,662	1,5
575	4,1	18,86	34,32	46,88	16,09	18,23	0,882	1,779
576	4,5	20,43	28,38	40,52	11,5	16,88	0,681	1,573
577	4,4	11,72	24,43	39,58	9,67	14,76	0,655	1,418
578	4,1	12,72	32,34	44,68	14,45	17,89	0,807	1,724
579	4,4	20,57	32,37	44,2	14,31	18,06	0,792	1,681
580	4,1	12,57	33,04	43,97	14,53	18,51	0,784	1,641
581	5,6	19,72	26,5	41,05	10,88	15,62	0,696	1,467
582	3,7	20,72	29,93	42,9	12,84	17,09	0,751	1,651
583	3,6	12,72	27,98	41,1	11,5	16,48	0,697	1,582
584	4,2	33,14	39,37	46,81	18,43	20,94	0,88	1,985
585	4,6	20	35,59	44,11	15,7	19,89	0,789	1,888
586	4,3	11,14	35,25	43,88	15,47	19,78	0,782	1,884
587	4,3	12,14	30,48	40,87	12,46	18,02	0,691	1,621
588	4,1	19,72	32,53	42,82	13,93	18,6	0,748	1,083
589	4,3	19,86	27,03	41,39	11,19	15,84	0,706	1,493
590	3,9	12,57	31,47	42,1	13,25	18,22	0,727	1,709
591	4,3	20	33,26	43,35	14,42	18,84	0,765	1,762
592	3,7	12,43	32,64	41,94	13,69	18,95	0,722	1,768
593	4,4	20,14	34,06	42,3	14,41	19,65	0,733	1,874

594	4,2	19,86	36,21	45,09	16,33	19,88	0,821	1,829
595	4,1	12,28	35,28	44,58	15,73	19,55	0,804	1,791
596	3,5	11,43	38,15	43,04	16,42	21,73	0,755	1,992
597	4,3	11,86	26,87	41,86	11,25	15,62	0,72	1,466
598	4	20	34,33	42,9	14,73	19,6	0,751	1,817
599	4,9	19,86	34,85	42,58	14,84	20,01	0,741	1,826
600	4,5	12,14	34,71	42,09	14,61	20,1	0,726	1,836
601	5,1	12,57	42,25	45,11	19,06	23,19	0,821	2,323
602	4,3	12,28	33,02	42,91	14,17	18,85	0,751	1,738
603	5,3	20,57	42,38	44,99	19,07	23,31	0,818	2,343
604	4,3	19,57	32,38	42,4	13,73	18,65	0,736	1,704
605	3,4	20,86	32,86	42,6	14	18,86	0,742	1,734
606	4,1	20	34,98	44,62	15,61	19,37	0,805	1,717
607	4,4	12,43	31,07	41,22	12,81	18,26	0,701	1,663
608	3,3	13,14	31,01	41,34	12,82	18,19	0,704	1,659
609		12,14						
610	3,5	11						
611	4,3	11,57	34,05	41,52	14,14	19,91	0,71	1,888
612	5,1	20,57	36,16	42,8	15,48	20,68	0,748	1,943
613	4,4	20	37,96	44,81	17,01	20,95	0,811	1,856
614	3,8	12	35,79	43,22	15,47	20,32	0,761	1,818
615	3,8	20,14	39,65	43,88	17,4	22,25	0,782	2,038
616	3,7	11	41,58	46,15	19,19	22,39	0,857	2,114
617	3,4	12	34,02	43,7	14,87	19,15	0,776	1,762
618	3,8	20	36,65	44,74	16,4	20,25	0,809	1,815
619	3,2	12,14	41,98	42,87	18	23,98	0,75	2,016
620	4	12,43	30,41	41,13	12,51	17,9	0,698	1,616
621	4,4	20	35,16	43,2	15,19	19,97	0,76	1,988
622	4,7	20	38,7	45,24	17,51	21,19	0,826	1,918
623	4,7	13	36,47	43,81	15,98	20,49	0,779	1,966
624	6	20,43	34,81	44,32	15,43	19,38	0,796	1,998
625	5	12,57	33,21	42,9	14,25	18,96	0,751	1,976
626		12,57						

627	4,6	11,14	36,69	44,04	16,16	20,53	0,787	1,876
628	3,5	35,14	43,65	44,94	19,62	24,03	0,816	2,378
629	4,7	20,28	34,18	43,68	14,43	19,25	0,775	1,831
630		11,72						
631	4,3	10,43	32,97	42,91	14,15	18,82	0,751	1,743
632	3,3	11,57	41,2	42,28	17,42	23,78	0,732	2,263
633	3,6	20,57	41,13	44,07	18,13	23	0,788	2,274
634	4	21,14	40,11	45,45	18,23	21,88	0,833	2,038
635	4,3	12,57	27,99	40,83	11,43	16,56	0,69	1,658
636	4,1	11,57	37,05	43,15	15,98	21,05	0,759	1,943
637	5,8	20,28	37	43,05	15,93	21,07	0,756	2,088
638	4,3	11,28	35,79	42,19	15,1	20,69	0,729	2,077
639		19	45,04	46,31	20,86	24,18	0,862	2,223
640	5	20,57	30,42	42,57	12,95	17,47	0,741	1,789
641	4,5	11,14	45,47	42,99	19,55	25,92	0,754	2,245
642	4,5	12,14	30,35	43,09	13,08	17,27	0,757	1,74
643		11,57						
644	5,1	19,72	35,01	44,64	15,63	19,38	0,806	1,819
645	4,8	12	34,31	42,87	14,71	19,6	0,75	1,819
646	4,8	12,72	32,91	41,93	13,8	19,11	0,722	1,811
647	4,9	20,86	33,95	42	14,26	19,69	0,724	1,861
648	4,1	20,28	36,2	44,25	16,02	20,18	0,793	1,82
649	3,6	12,28	36,17	43,79	15,84	20,33	0,779	1,781
650		12,28						
651	4,8	12,57	33,32	43,57	14,52	18,8	0,772	1,776
652	4,8	12,86	33,41	42,11	14,07	19,34	0,727	1,863
653	4,2	19,72	34,09	42,41	14,46	19,63	0,736	1,901
654	4,4	19,86	29,63	41,64	12,34	17,29	0,713	1,592
655	3,2	12,28	29,37	42,21	12,4	16,97	0,73	1,537
656		11,28						
657	3,6	12						
658	3,8	13,72	30,62	43,69	13,38	17,24	0,776	1,534
659	3,8	19,72	31,25	43,2	13,5	17,75	0,76	1,578

660		12,57						
661	4,6	20,72	37,69	45,79	17,26	20,43	0,844	1,879
662	4	12,72	36,93	44,97	16,61	20,32	0,817	1,816
663	4,4	19,86	37,45	45,2	16,93	20,52	0,825	1,906
664	3,9	13,14	35,04	43,4	15,21	19,83	0,767	1,843
665		12,28						
666	5,3	20	35,92	43,17	15,51	20,41	0,759	1,913
667	5,2	12,28	37,36	43,54	16,27	21,09	0,771	1,856
668	5,3	20,14	35,3	42,63	15,05	20,25	0,743	1,855
669	4,4	12,57	33,49	41,23	13,81	19,68	0,701	1,784
670		11,43						
671	3,9	12,28	30,56	41,78	12,77	17,79	0,717	1,678
672	4,4	20,43	30,8	41,33	12,73	18,07	0,704	1,753
673		11,43						
674	3,7	20,72						
675	3,2	12,72	34,66	44,43	15,4	19,26	0,799	1,683
676	3,7	12,28	31,94	41,85	13,37	18,57	0,719	1,693
677	3,8	12,28	35,09	45,19	15,86	19,23	0,824	1,714
678	4	11,72	34,5	43,39	14,97	19,53	0,766	1,914
679	4,2	20,57	34,37	43,29	14,88	19,49	0,763	1,95
680		12,14						
681	4,2	11,57	33,22	41,81	13,89	19,33	0,718	1,932
682		12,43						
683	4,9	11,57	33,79	43,62	14,74	19,05	0,773	1,71
684	4,8	19,57	34,14	44,02	15,03	19,11	0,786	1,749
685	3,8	20,86	36,99	46,01	17,02	19,97	0,852	2,001
686	3,3	12,57	34,61	44,32	15,34	19,27	0,796	1,961
687	3,7	22,72	36,73	44,4	16,31	20,42	0,798	1,762
688	3,4	13						
689		11,86						
690	3,4	11,86	30,48	42,19	12,86	17,62	0,729	1,605
691	4,4	19,72	31,17	42,6	13,28	17,89	0,742	1,605
692	4,2	33						

693	4,3	20,14						
694	4,3	13,57						
695		12,14						
696		12,86						
697	3,4	12,72	36,05	44,85	16,17	19,88	0,813	1,877
698	4,3	20,28	34,09	43,53	14,84	19,25	0,77	1,674
699	3,4	11,72	33,37	43,99	14,68	18,69	0,785	1,616
700	4	20,43	35,06	45,2	15,85	19,21	0,825	1,69
701	3,4	11,72	34,24	44,88	15,37	18,87	0,814	1,635
702		11,43						
703		11,72						
704		12,28						
705		11,72						
706		12,43						
707		11,43						
708		12,72						
709		12,86						
710		13,57						
711		12,57						
712		13						
713		13,72						
714		11,43						
715		14,14						
716		12,14						
717	4,2	13,86	30,19	41,5	12,53	17,66	0,709	1,781
718	4,6	20	30,36	41,17	12,5	17,86	0,699	1,813
719		11,43						
720		12						
721		10,57						
722		12,57						
723		13,14						
724		12						
725		12,28						

726		11,72						
727		12,43						
728		12						
729		11,86						
730		11,14						
731		12,72						
732		11,86						
733	4,9	19,43	33,13	42,74	14,16	18,97	0,746	1,709
734	4,2	11,43	32,12	42,09	13,52	18,6	0,726	1,654
735	4,3	19,14	33,84	44,17	14,95	18,89	0,791	1,808
736	4,8	12,14	35,97	44,89	16,15	19,82	0,814	1,789
737		12,43						
738	3,9	12,43	32,67	43,83	14,32	18,35	0,78	1,611
739	4,2	20,43	35,1	45,32	15,91	19,19	0,829	1,691
740	3,8	12,43	32,49	43,7	14,2	18,29	0,776	1,638
741		12,14						
742	4,8	20,14	36,01	43,12	15,53	20,48	0,758	1,92
743	4,6	12	35,04	42,43	14,87	20,17	0,737	1,883
744		13,28						
745		11,43						
746		11,86						
747	4,7	19,86	35,03	43,27	15,16	19,87	0,762	1,92
748	4,9	11,43	34,24	43,22	14,8	19,44	0,761	1,867
749	5,2	18,14	30,81	43,13	13,29	17,52	0,758	1,636
750	5,1	12,14	29,03	41,3	11,99	17,04	0,703	1,609
751		12,14						
752		11,57						
753		11,43						
754		13,43						
755		12,57						
756	4,1	21,14	31,92	42,88	13,69	18,23	0,75	1,765
757	3,7	12,43	31,15	42,18	13,14	18,01	0,729	1,699
758	4,1	12,57						

759	3,7	19,57	33,03	41,81	13,81	19,22	0,718	1,807
760	4,3	12,86	39,56	44,18	17,48	22,08	0,791	1,93
761	4,4	19,72	42,19	45,81	19,33	22,86	0,845	1,974
762	4,6	20,43	32,78	42,49	13,93	18,85	0,738	1,842
763	4,1	11,72	31,26	41,07	12,84	18,42	0,697	1,824
764	4,1	11,57	33,42	41,95	14,02	19,4	0,722	1,754
765	4,6	19,72	35,81	43,5	15,58	20,23	0,77	1,807
766	4,5	20,14	35,35	43,39	15,34	20,01	0,766	1,914
767	3,4	11,28	35,05	42,71	14,97	20,08	0,745	1,926
768	4,6	21,28	42,59	46,34	19,74	22,85	0,863	2,339
769	4,1	13						
770	4,1	19,86	31,62	40,82	12,91	18,71	0,69	1,726
771	4,1	12,86	29,36	38,89	11,42	17,94	0,636	1,678
772	5,1	12,86	31,48	45,01	14,17	17,31	0,818	1,548
773	5,7	19,28	31,47	45,12	14,2	17,27	0,822	1,59
774	4,4	19,86	29,82	42,99	12,82	17	0,754	1,572
775	3,9	12,43	29,23	42,35	12,38	16,85	0,734	1,537
776		11,57						
777		12,28						
778		13,14						
779	7,3	20,57	38,67	44,32	17,14	21,53	0,796	2,188
780	5,6	13	38,2	44,34	16,94	21,26	0,796	2,166
781	4	12,86	35,57	43,4	15,44	20,13	0,767	1,852
782	4,3	20	36,19	43,74	15,83	20,36	0,777	1,896
783	4,3	11,43	31,98	41,83	13,38	18,6	0,719	1,654
784	4,5	12,86	32,17	43,73	14,07	18,1	0,777	1,653
785	4,6	21,43	32,1	41,83	13,43	18,67	0,719	1,7
786	4,5	19,28	31,61	44,09	13,94	17,67	0,788	1,729
787	4,2	13,28	31,18	44,29	13,81	17,37	0,795	1,695
788		11,28						
789	4,8	21	33,65	42,88	14,43	19,22	0,75	1,768
790	3,9	13	32,22	41,68	13,43	18,79	0,714	1,707
791	4,3	19,72	34,53	42,57	14,7	19,83	0,741	1,791

792	4	12	33,95	42,2	14,33	19,62	0,73	1,751
793		13,28						
794	3,7	20	34,12	43,78	14,94	19,18	0,778	1,692
795	3,5	11,86	31,78	41,53	13,2	18,58	0,71	1,635
796	4,2	21	31,29	43,84	13,72	17,57	0,78	1,581
797	3,4	13,57	29,7	42,72	12,69	17,07	0,746	1,539
798	4,2	21,14	40,98	44,48	18,23	22,75	0,801	1,991
799	3,8	11,72	37,68	43,15	16,26	21,42	0,759	1,949
800	4,9	19,86	36,42	44,39	16,17	20,25	0,798	1,894
801	4,2	13,86						
802	3,8	20,86	31,2	41,47	12,94	18,26	0,708	1,682
803	3,7	13,14	31,02	41,97	13,02	18	0,723	1,654
804	4,8	20,57	31,74	43,41	13,78	17,96	0,767	1,712
805	4,3	11	30,57	42,13	12,88	17,69	0,728	1,646
806	4,5	19,86	37,64	45,51	17,13	20,51	0,835	2,045
807	4,7	11,86	37,08	44,84	16,63	20,45	0,813	2,023
808		12,43						
809	4,4	20,14	31,03	43,11	13,38	17,65	0,758	1,619
810	4,1	12,57	29,47	41,83	12,33	17,14	0,719	1,549
811	4,7	22	33,51	44,01	14,75	18,76	0,786	1,714
812	4,1	12	33,41	43,99	14,7	18,71	0,785	1,659
813		10,72						
814	4,3	12,14	38,08	44,11	16,8	21,28	0,789	1,906
815	5,1	19,86	38,15	44,63	17,03	21,12	0,806	1,902
816	3,8	11,72	28,53	40,44	11,54	16,99	0,679	1,543
817	4,6	19,86	35,29	44,99	15,88	19,41	0,818	1,736
818	3,9	11	32,36	43,32	14,02	18,34	0,764	1,683
819	4,3	28	31,17	43,08	13,43	17,74	0,757	1,591
820	3,9	18,43	34,51	45,08	15,56	18,95	0,821	1,715
821	3,5	12	31,57	42,57	13,44	18,13	0,741	1,667
822		13						
823		13,14						
824	4,6	19,43	36,04	43,84	15,8	20,24	0,78	1,688

825	4,4	34,72	35,75	45,79	16,37	19,38	0,844	1,943
826	4,5	20,57	32,2	43,38	13,97	18,23	0,766	1,788
827	3,9	11,72	32,49	43,05	13,99	18,5	0,756	1,788
828		11,28						
829	3,3	12,14	31,43	42,25	13,28	18,15	0,731	1,601
830		11,14						
831	3,5	12,57	34,69	44,04	15,28	19,41	0,787	1,765
832	3,9	17,86	33,26	43,47	14,46	18,8	0,769	1,784
833	4,4	19,43	29,87	42,95	12,83	17,04	0,752	1,571
834	4	11,43	28,1	41,7	11,72	16,38	0,715	1,508
835		11,72						
836		12,57						
837	3,9	20,86	33,22	43,91	14,59	18,63	0,783	1,718
838	3,8	12,57	31,71	43,23	13,71	18	0,761	1,651
839	4,3	11,43	33,41	41,93	14,01	19,4	0,722	1,793
840	4,5	20,43	33,5	42,08	14,1	19,4	0,726	1,843
841	3,8	19	34,35	43,81	15,05	19,3	0,779	1,865
842	3,4	12	34,69	44,01	15,27	19,42	0,786	1,872
843	4	12,57	35,22	41,11	14,48	20,74	0,698	1,941
844	4,5	19,86	35,29	41,25	14,56	20,73	0,702	1,962
845	4,6	21,14	40,87	45,92	18,77	22,1	0,849	2,066
846	4,1	12	39,82	46,2	18,4	21,42	0,859	2,049
847	4,2	12,43	37,19	43,18	16,06	21,13	0,76	1,881
848	4,5	21,43	39,24	43,8	17,19	22,05	0,779	1,947
849	3,3	13,43	31,01	40,34	12,51	18,5	0,676	1,711
850	3,8	20,14	32,32	41,24	13,33	18,99	0,701	1,751
851	4,4	21	32,65	44,28	14,46	18,19	0,794	1,705
852	4,1	11,57	29,88	41,96	12,54	17,34	0,723	1,667
853		12,14						
854	3,9	10,57	36,03	42,85	15,44	20,59	0,749	1,873
855	4,6	20	37,39	43,67	16,33	21,06	0,775	1,925
856		7,72						
857	4,5	20,57	29,62	42,33	12,54	17,08	0,734	1,553

858	4,2	12,57	29,69	42,74	12,69	17	0,746	1,517
859	4	10,43	30,19	42,03	12,69	17,5	0,725	1,615
860	3,7	19,57	31,39	42,97	13,49	17,9	0,753	1,697
861	4,5	12,86	38,54	45,48	17,53	21,01	0,834	2,047
862	4,9	28,14	39,51	45,93	18,15	21,36	0,849	2,097
863	4,1	21,14	39,02	45,95	17,93	21,09	0,85	2,064
864	4	19,72	36,94	44,15	16,31	20,63	0,79	1,834
865	3,8	12,72	37,3	44,02	16,42	20,88	0,786	1,801
866	5,1	12						
867	5,4	20,57	56,3	49,92	28,11	28,19	0,997	2,442
868	4,8	20,57	33,55	43,6	14,63	18,92	0,773	1,856
869	4,8	12,14	33,56	43,77	14,69	18,87	0,778	1,795
870	4,2	20,43	30,28	41,8	12,66	17,62	0,718	1,673
871	3,4	11,28	29,31	41,28	12,1	17,21	0,703	1,591
872	3,7	20,57	32,96	40,01	13,19	19,77	0,667	1,927
873	3,4	11,57	33,73	40,67	13,72	20,01	0,685	1,914
874	4,8	20,14	31,58	42,65	13,47	18,11	0,743	1,659
875	4,5	12,86	32,02	42,62	13,65	18,37	0,743	1,632
876		12,28						
877	4,1	19,86	35,04	44,17	15,48	19,56	0,791	1,778
878	3,1	12,28	34,22	43,83	15	19,22	0,78	1,751
879	3,6	19,86	33,52	44,15	14,8	18,72	0,79	1,866
880	3,7	12,86	32,47	44,22	14,36	18,11	0,792	1,803
881	3,5	12	47,61	47,82	22,77	24,84	0,916	2,272
882	3,7	20	45,84	46,48	21,31	24,53	0,868	2,226
883	3,5	19,86	35,05	43,82	15,36	19,69	0,78	1,883
884	3,7	11,86	33,45	41,88	14,01	19,44	0,72	1,883
885	4,2	20,14	39,64	45,2	17,92	21,72	0,825	1,802
886	4,1	12,43	37,11	43,97	16,32	20,79	0,784	1,734
887		12						
888	5	20,86	34,05	44,28	15,08	18,97	0,794	1,785
889	4,3	13,28	34,51	44,59	15,39	19,12	0,804	1,733
890	4,5	13,14	32,14	42,9	13,79	18,35	0,751	1,832

891	5,1	20,86	48,84	47,78	23,34	25,5	0,915	2,33
892	4,3	12	52,71	46,32	24,42	28,29	0,863	2,299
893		13						
894	3,8	20,14	42,84	45,51	19,5	23,34	0,835	2,212
895	3,5	10,72	43,26	45,05	19,49	23,77	0,819	2,234
896		11,86						
897	4,3	12,28	32,7	42,17	13,79	18,91	0,729	1,751
898	4,3	19,86	34,94	44,41	15,52	19,42	0,799	1,791
899	4	20,14	28,42	41,62	11,83	16,59	0,713	1,554
900	2,9	11,57	28,38	41,75	11,85	16,53	0,716	1,495
901		13,86						
902	3,7	12,57	31,57	43,01	13,58	17,99	0,754	1,661
903	4,6	21,43	33,28	43,93	14,62	18,66	0,783	1,722
904		11,43						
905	4,1	13,86	37,25	43,38	16,16	21,09	0,766	1,884
906	5,4	20,28	39,36	44,58	17,55	21,81	0,804	1,949
907	4,5	11,28	30,85	41,58	12,83	18,02	0,711	1,668
908	4,3	20,28	30,7	41,33	12,69	18,01	0,704	1,668
909		10,86						
910		11,14						
911		13,14						
912		11,86						
913	4,5	20,28	34,43	41,88	14,42	20,01	0,72	1,847
914	3,7	11,72	33,22	41,57	13,81	19,41	0,711	1,771
915	4,1	19,72	37,78	44,81	16,93	20,85	0,811	2,059
916	3,6	11,43	35,25	43,09	15,19	20,06	0,757	2,037
917		12,14						
918	4,2	23,72						
919	4,5	12,72						
920		12						
921		12,86						
922	3,4	13,72	27,86	43,43	12,1	15,76	0,767	1,438
923	3,2	20,28	27,57	43,38	11,96	15,61	0,766	1,452

924		12,86						
925	4,4	27,72	37,82	46,35	17,53	20,29	0,863	1,856
926	3,4	11	35,58	44,32	15,77	19,81	0,796	1,838
927	4,2	21,28	33,3	43	14,32	18,98	0,754	1,687
928	3,6	11,28	30,65	40,84	12,52	18,13	0,69	1,63
929	3,8	20,43	32,8	41,98	13,77	19,03	0,723	1,697
930	3,8	12,86	32,21	41,41	13,34	18,87	0,706	1,669
931	4,1	12,72	34,86	44,52	15,52	19,34	0,802	1,736
932	4,3	20,43						
933	3,6	11,43	39,88	44,5	17,75	22,13	0,802	1,962
934	3,5	11	33,82	43,31	14,65	19,17	0,764	1,811
935	4,3	20	32,33	43,61	14,1	18,23	0,773	1,707
936	4,7	20,28						
937	4,6	12,28	43,14	44,92	19,38	23,76	0,815	2,241
938	4,2	20,28	34,67	47,27	16,39	18,28	0,896	1,837
939	4	12,28	32,61	45,35	14,79	17,82	0,829	1,791
940	4,5	20,72	36,9	43,44	16,03	20,87	0,768	1,985
941	3,8	11,14	37,03	43,42	16,08	20,95	0,767	1,997
942	5,4	20,28	36,99	42,57	15,75	21,24	0,741	1,992
943	4,3	12,28						
944		13,57						
945	4,7	24	31,49	40,93	12,89	18,6	0,693	1,741
946	4,4	12,28	31,08	41,11	12,78	18,3	0,698	1,694
947	4,1	12,86	31,04	42,2	13,1	17,94	0,73	1,802
948	4,9	19,86	38,76	46,51	18,03	20,73	0,869	2,126
949	4,6	11,86	37,22	45,35	16,88	20,34	0,829	2,094
950	5,5	20,57	35,19	41,94	14,76	20,43	0,722	2,003
951	4,8	12,86	33,87	41,39	14,02	19,85	0,706	1,956
952		12,28						
953	4,1	12,57	36	43,22	15,56	20,44	0,761	1,833
954	4,9	20,72	31,5	41,2	12,98	18,52	0,7	1,721
955	4	12,57	29,53	39,65	11,71	17,82	0,657	1,671
956	5,7	21,14	34,68	42,7	14,81	19,87	0,745	2,028

957	5,2	13,43	32,33	40,89	13,22	19,11	0,691	1,961
958	4,3	11,86	33,25	42,37	14,09	19,16	0,735	1,773
959	5,3	20,28	35,46	44,1	15,64	19,82	0,789	1,854
960	4	13	31,12	41,19	12,82	18,3	0,7	1,775
961	3,6	20,43	41,04	44,88	18,42	22,62	0,814	2,018
962	3,3	11,72	39,1	44,09	17,24	21,86	0,788	2,018
963	4,1	12,28	33,58	40,64	13,65	19,93	0,684	1,777
964	3,8	20,28	36,02	42,55	15,33	20,69	0,74	1,844
965	4,2	33	35,18	44,31	15,59	19,59	0,795	1,879
966	4,4	19,28	33,18	43,94	14,58	18,6	0,783	1,82
967	4,1	11,28	31,08	41,24	12,82	18,26	0,702	1,807
968	3,4	11,28	32,53	43,03	14	18,53	0,755	1,673
969	3,9	19,28	32,48	43,34	14,08	18,4	0,765	1,673
970	4,3	19,43	39,56	45,52	18,01	21,55	0,835	2,021
971	4	14,28	40,04	46,05	18,44	21,6	0,853	1,997
972		10,57						
973		13,28						
974	3,6	12,57	30,06	42,08	12,65	17,41	0,726	1,689
975	4,3	20	31	42,38	13,14	17,86	0,735	1,727
976	6,2	20,57	40,51	43,54	17,64	22,87	0,771	2,243
977	5,4	12,57	37,99	42,82	16,27	21,72	0,749	2,19
978		12,57						
979	3,9	10,28	27,76	39,73	11,03	16,73	0,659	1,577
980		12,86						
981	4,9	26,14						
982	4,1	10,86						
983	4,7	25,86						
984	3,9	12	34,29	44,47	15,25	19,04	0,8	1,7
985	5,5	20,14						
986	5,2	13,14	34,08	43,77	14,92	19,16	0,778	1,861
987	4	11,18	29,99	40,78	12,23	17,76	0,688	1,554
988	4,5	20,14	30,81	41,05	12,65	18,16	0,696	1,602
989	5,1	12,43	33,41	44,32	14,81	18,6	0,796	1,729

990	5,8	20,43	35,02	45,6	15,97	19,05	0,838	1,766
991	4,2	10,86	33,71	42,09	14,19	19,52	0,726	1,851
992	4,8	20,43	32,95	41,45	13,66	19,29	0,708	1,844
993	4,3	12,43	34,44	44,51	15,33	19,11	0,802	1,897
994	4	21	39,59	44,6	17,66	21,93	0,805	2,038
995	3,7	30,14	40,32	46,77	18,86	21,46	0,787	2,062
996	3,9	13,72	37,23	43,32	16,13	21,1	0,764	2,032
997	4,6	20,28	36,84	45,05	16,6	20,24	0,82	1,95
998	4,5	13,86	35,6	43,82	15,6	20	0,78	1,914
999		12						
1000		12,28						
1001		12,14						
1002		12						
1003	5,5	33,14	50,53	47,04	23,77	26,76	0,888	2,558
1004	5,5	20,28	49,46	48,84	24,16	25,3	0,954	2,5
1005	5,6	13,14	45,37	46,59	21,14	24,23	0,872	2,467
1006	5,5	30,43	46,49	46,28	21,52	24,97	0,861	2,294
1007	4,7	20,43	44,28	45,14	19,99	24,29	0,822	2,222
1008	6,3	12,72	43,87	45,11	19,79	24,08	0,821	2,144
1009	5	12,72	35,6	45,19	16,09	19,51	0,824	1,943
1010	5,2	19,72	35,83	44,48	15,94	19,89	0,801	1,983
1011	4,6	12,86	38,64	43,89	16,96	21,68	0,782	2,091
1012	4,6	21,28	51,57	48,32	24,92	26,65	0,935	2,103
1013	4,6	20,72	33,41	42,53	14,21	19,2	0,74	1,837
1014	4,3	11,28	32,02	42,09	13,48	18,54	0,727	1,787
1015		12,14						
1016	4,8	19,86	27,73	42,33	11,74	15,99	0,734	1,491
1017	4,7	12,86	26,57	41,13	10,93	15,64	0,698	1,462
1018		11						
1019	4,5	20,14	28,77	41,04	11,81	16,96	0,696	1,634
1020	4,1	12,28	27,13	39,43	10,7	16,43	0,651	1,592
1021		12,43						
1022	3,9	11,43	37,43	41,62	15,58	21,85	0,713	1,902

1023	4,3	20,43	37,43	41,35	15,48	21,95	0,705	1,947
1024	3,8	12,57	36,57	43,15	15,78	20,79	0,759	2,22
1025		12						
1026		12,14						
1027	4,1	21,43	31,72	44,82	14,22	17,5	0,812	1,605
1028	4,1	12,28	30,89	44,12	13,63	17,26	0,789	1,563
1029	4	20						
1030	3,4	12,57	30,78	40,77	12,55	18,23	0,688	1,723
1031	3,4	32,86	36	46,13	16,61	19,39	0,856	1,941
1032	3,5	19,86	35,25	44,93	15,84	19,41	0,816	1,93
1033	3,3	12,72	35,53	45,73	16,25	19,28	0,842	1,919
1034	4,1	19,72	38,73	42,7	16,54	22,19	0,745	2,091
1035	4,2	10,72	38,85	42,9	16,67	22,18	0,751	2,061
1036	4,1	12	35,81	42,41	15,19	20,62	0,736	2,096
1037	4,4	13,57	41,93	44,97	18,86	23,07	0,817	1,981
1038		12						
1039	4,6	20,14	33,87	39,82	13,49	20,38	0,661	1,972
1040	4,3	12,57	33,4	39,49	13,19	20,21	0,652	1,922
1041	4	12	32,34	40,69	13,16	19,18	0,686	1,702
1042	4,8	20	33,19	41,45	13,76	19,43	0,708	1,744
1043	4,7	19,72	34,47	43,6	15,03	19,44	0,773	1,798
1044	3,9	12,14	33,29	42,5	14,15	19,14	0,739	1,772
1045	4,1	36,72	40,05	44,64	17,88	22,17	0,806	2,145
1046	3,8	12,28	37,15	43,17	16,04	21,11	0,759	2,05
1047	4,6	22,72	37,88	43,21	16,37	21,51	0,761	2,061
1048		14,28						
1049	3,7	11,86	29,5	43,69	12,89	16,61	0,776	1,593
1050	4,1	20,14	33,84	44,73	15,14	18,7	0,809	1,62
1051		12,28						
1052	4,5	19,72	28,06	42,19	11,84	16,22	0,729	1,462
1053	4,1	11,72	27,74	42,6	11,82	15,92	0,742	1,407
1054	3,8	11,43	31,01	43,92	13,62	17,39	0,783	1,609
1055	4	20,43	31,78	44,33	14,09	17,69	0,796	1,622

1056		11,57						
1057	4	20,43	35,36	42,61	15,07	20,29	0,742	1,967
1058	3,9	12	34,68	41,83	14,51	20,17	0,719	1,914
1059	3,7	11,57	33,65	43,95	14,79	18,86	0,784	1,738
1060	3,9	32,86	38,71	46,44	17,98	20,73	0,867	1,92
1061	3,8	20,14	35,73	45,25	16,17	19,56	0,826	1,825
1062	4,6	20,14	39,24	45,41	17,82	21,43	0,831	1,996
1063	3,6	12,28	37,39	44,85	16,77	20,62	0,813	1,962
1064	4,3	20	35,8	42,73	15,3	20,5	0,746	2,22
1065	4,4	29,14	33,25	44,24	14,75	18,54	0,793	1,831
1066	4	12,43	34,97	45,38	15,87	19,1	0,83	1,784
1067	4,3	20,57	36,32	45,89	16,67	19,65	0,848	1,844
1068	4	30,14	32,7	46,05	15,06	17,64	0,853	1,82
1069	3,6	20,14	32,71	45,52	14,89	17,82	0,835	1,831
1070	4,1	12,14	32,24	44,75	14,43	17,81	0,81	1,807
1071	4	11,72	32,41	43,44	14,08	18,33	0,768	1,858
1072	4,5	19,72	34,16	44,49	15,2	18,96	0,801	1,844
1073		12,14						
1074		11,57						
1075	3,5	11,86	38,05	45,44	17,29	20,76	0,832	1,899
1076	4,1	19,72	39,43	46,91	18,5	20,93	0,883	1,912
1077		12,57						
1078	3,7	10,86	33,21	42,78	14,21	19	0,747	1,723
1079	5,2	20,43	45,72	46,08	21,07	24,65	0,854	2,342
1080	4,6	14	44,91	45,98	20,65	24,26	0,851	2,321
1081		11,43						
1082	4,2	21,14	33,92	44,22	15	18,92	0,792	1,799
1083	3,7	10,86	32,19	42,87	13,8	18,39	0,75	1,746
1084	4,4	20,14	31,92	41,13	13,13	18,79	0,698	1,842
1085	4,4	11,14	33,06	41,22	13,63	19,43	0,701	1,855
1086	4,3	12,28	35,57	44,02	15,66	19,91	0,786	1,957
1087	5,2	20,43	48,06	52,28	25,13	22,93	1,095	1,957
1088	3,8	11,72	29,69	44,12	13,1	16,59	0,789	1,483

1089		12,72						
1090	2,9	20,72	26,59	40,2	10,69	15,9	0,672	1,475
1091	3,3	10,28	24,89	38,16	9,5	15,39	0,617	1,468
1092		11,43						
1093	3,3	12	25,94	41,82	10,85	15,09	0,719	1,356
1094		11,43						
1095	4,8	21	35,16	43,37	15,25	19,91	0,765	1,833
1096	5	12,28	36,58	44,45	16,26	20,32	0,8	1,806
1097	4,9	9,86	35,76	40,4	14,45	21,31	0,678	1,985
1098	5,7	19,57						
1099	4,6	32,43	37,59	44,98	16,91	20,68	0,817	1,951
1100	4,3	21,28	34,86	43,31	15,1	19,76	0,764	1,864
1101	3,9	11,14	34,63	43,54	15,08	19,55	0,771	1,83
1102		12,28						
1103	5	30	36,38	44,61	16,23	20,15	0,805	1,991
1104	5,3	20,72	34,24	43,34	14,84	19,4	0,764	1,931
1105	4,2	10,57	34,77	44,2	15,37	19,4	0,792	1,881
1106	3,8	21,14	32,67	43,52	14,22	18,45	0,77	1,697
1107	3,7	11,28	32,46	42,42	13,77	18,69	0,736	1,67
1108	4,1	20,72	37,3	46,4	17,31	19,99	0,865	1,913
1109	3	11,72	38,33	46,15	17,69	20,64	0,857	1,913
1110	4,5	32,14	36,11	42,53	15,36	20,75	0,74	1,852
1111	4,8	21,43	36,3	43,91	15,94	20,36	0,782	1,839
1112	4,4	12,43	34	42,2	14,35	19,65	0,73	1,786
1113		13,28						
1114	5,1	21,72	35,51	43,64	15,5	20,01	0,774	1,891
1115	3,7	12,14	34	41,82	14,22	19,78	0,718	1,82
1116		12,72						
1117	4,1	11,28	36,59	43,2	15,81	20,78	0,76	2,037
1118	4	28,28	38,97	44,29	17,26	21,71	0,795	2,152
1119	4	19,28	42,31	44,05	18,64	23,67	0,787	2,083
1120		12,57						
1121	4,8	20,86	35,06	45,23	15,86	19,2	0,826	1,67

1122	4,8	12,28	32,71	43,25	14,15	18,56	0,762	1,615
1123	3,9	13,14	41,78	44,63	18,65	23,13	0,806	2,167
1124	4,3	20,14	42	44,71	18,78	23,22	0,808	2,19
1125	4,5	19,43	27,9	41,43	11,56	16,34	0,707	1,532
1126	3,9	12	26,56	40,28	10,7	15,86	0,674	1,481
1127		12,14						
1128	4,4	20,28	40,3	45,13	18,19	22,11	0,822	2,151
1129		12,72						
1130	4	12,28	39,74	44,81	17,81	21,93	0,812	2,118
1131	3,8	20,28	30,82	42,18	13	17,82	0,729	1,673
1132	3,8	12	30,48	42,15	12,85	17,63	0,728	1,554
1133	5,1	21,14	30,78	42,69	13,14	17,64	0,744	1,601
1134	3,8	12,43	31,72	40,54	12,86	18,86	0,681	1,659
1135	4,8	30,57	33,49	44,19	14,8	18,69	0,791	1,873
1136	4,5	20,28	31,82	41,57	13,23	18,59	0,711	1,833
1137	4,4	11,86	31,84	42,43	13,51	18,33	0,737	1,814
1138	4,5	20,28	34,79	46,42	16,15	18,64	0,866	1,712
1139	3,9	11,28	38,13	45,05	17,18	20,95	0,82	1,678
1140		11,43						
1141	4,3	12,72	35,18	44,54	15,67	19,51	0,803	1,772
1142	4	12,72	34,04	43,24	14,72	19,32	0,761	1,778
1143	4,1	12						
1144	3,4	13	29,99	41,34	12,4	17,59	0,704	1,578
1145	4,3	19,14	35,73	44,58	15,93	19,8	0,804	1,791
1146	4,6	20,86	36,46	43,58	15,89	20,57	0,772	1,911
1147	4,4	20,72	31,46	41,73	13,13	18,33	0,716	1,65
1148	4,5	12,43	35,48	42,81	15,19	20,29	0,748	1,911
1149	5	19,72	35,85	43,73	15,68	20,17	0,777	1,783
1150	4,9	12,14	35,32	43,71	15,44	19,88	0,776	1,721
1151	3,8	13,14	32,43	42,89	13,91	18,52	0,751	1,638
1152	4,4	20	31,5	41,42	13,05	18,45	0,707	1,65
1153	4	13	29,01	41,5	12,04	16,97	0,709	1,605
1154		12,72						

1155	4,5	19,86	33,27	42,35	14,09	19,18	0,734	1,777
1156	3,5	11,86	34,42	44,47	15,31	19,11	0,801	1,749
1157	3,7	12,28	30,81	40,18	12,38	18,43	0,671	1,733
1158	3,6	19,43	34,81	45,33	15,78	19,03	0,829	1,762
1159	5,9	20,28	35,67	44,18	15,76	19,91	0,791	1,869
1160	5	12,86	33,32	42,43	14,14	19,18	0,737	1,818
1161		11,28						
1162	4,4	20,57	31,61	41,5	13,12	18,49	0,709	1,714
1163	4,6	12,43	30,72	40,16	12,34	18,38	0,671	1,741
1164		11,86						
1165		12,14						
1166		13						
1167	3,9	11,86	29,65	41,68	12,36	17,29	0,714	1,539
1168		11,43						
1169	4,2	20,43	34,56	42,5	14,69	19,87	0,739	1,794
1170	4,2	11,72	33,85	42,51	14,39	19,46	0,739	1,739
1171	4,7	21,43	38,47	44,78	17,23	21,24	0,811	1,818
1172	4,2	19,72	33,82	45,38	15,35	18,47	0,831	1,788
1173	3,8	13,43	36,73	44,05	16,18	20,55	0,787	1,758
1174	3,6	12,28	32,52	44,15	14,36	18,16	0,79	1,751
1175	4,6	20,86	35,15	44,21	15,54	19,61	0,792	1,926
1176	5,1	11,86	36,02	43,33	15,61	20,41	0,764	1,938
1177	3,9	12,72	30,29	42,48	12,87	17,42	0,738	1,632
1178	4,2	20,14	30,82	43,47	13,4	17,42	0,769	
1179	4,3	13,28	34,26	43,89	15,04	19,22	0,782	1,882
1180		12,14						
1181	4,7	20,43	41,24	45,92	18,94	22,3	0,849	1,892
1182	4,3	12,57	34,91	42,53	14,85	20,06	0,74	1,82
1183	4,1	20,86	33,46	43,15	14,44	19,02	0,759	1,709
1184	3,9	21,28	35,8	43,51	15,58	20,22	0,77	1,87
1185	4,2	12,57	32,63	42,78	13,96	18,67	0,747	1,702
1186	4,1	12,86	32,68	43,14	14,1	18,58	0,758	1,64
1187	5,7	21,14	39,17	44,31	17,36	21,81	0,795	2,072

1188	3,6	11,72	34,4	42,93	14,77	19,63	0,752	1,807
1189	3,5	13,14	24,69	38,88	9,6	15,09	0,636	1,5
1190	5,6	13,14	37,65	43,85	16,51	21,14	0,78	2,003
1191	4,2	20	34,55	44,51	15,38	19,17	0,802	1,736
1192	4,5	36,14	40,78	45,95	18,74	22,04	0,85	2,05
1193	4,2	20,57	35,45	42,42	15,04	20,41	0,736	1,923
1194	4,7	12,72	37,96	42,67	16,2	21,76	0,744	2,062
1195	3,9	13	34,43	41,47	14,28	20,15	0,708	1,87
1196	4,8	19,86	41,01	45,15	18,52	22,49	0,823	2,042
1197		11,86						
1198	3,5	12,28	32,25	43,34	13,98	18,27	0,765	1,704
1199	3,9	19,28	32,55	43,34	14,11	18,44	0,765	1,743
1200		13,14						
1201		11,86						
1202		11,86						
1203	4,1	33,86	36,27	45,05	16,34	19,93	0,819	1,988
1204	3,4	27,86	34,49	44,12	15,22	19,27	0,789	1,885
1205	3,7	13,14	33,39	44,41	14,83	18,56	0,799	1,788
1206	4,8	19,86	33,77	43,61	14,73	19,04	0,773	1,813
1207	4,4	12,86	31,79	41,8	13,29	18,5	0,718	1,768
1208	4,5	11,43	32,37	43,71	14,15	18,22	0,776	1,632
1209	3,6	20,86	27,53	41,95	11,55	15,98	0,722	1,434
1210	4,1	12,86	35,76	42,39	15,16	20,6	0,735	1,909
1211	3,8	13,14	27,81	43,86	12,2	15,61	0,789	1,391
1212		12,43						
1213	4,6	19,14	36,23	44,99	16,3	19,93	0,817	1,941
1214	4,6	12,14	32,81	39,98	13,12	19,69	0,666	1,818
1215	4,4	20,43	39,13	45,79	17,92	21,21	0,844	1,939
1216	3,6	13,72	34,84	43,48	15,15	19,69	0,769	1,885
1217		12,28						
1218	4	10,57	37,88	45,24	17,14	20,74	0,826	1,902
1219	3,6	11,57	37,09	44,18	16,39	20,7	0,791	1,972
1220	5,2	20,72	32,01	41,08	13,15	18,86	0,697	1,862

1221	4,7	20,57	40,75	44,09	17,97	22,78	0,788	2,265
1222		12,28						
1223	3,5	12,57	39,59	43,49	17,22	22,37	0,769	2,188
1224	3	20,57	37,12	43,69	16,22	20,9	0,776	2,02
1225		12						
1226	4,2	12,57	37,51	44,14	16,56	20,95	0,79	1,979
1227	4	12,86	33,28	43,29	14,41	18,87	0,763	1,699
1228	4,5	22	36,78	43,82	16,12	20,66	0,78	1,93
1229	4	13,14	35,34	42,84	15,14	20,2	0,749	1,867
1230		11,86						
1231		20,57	36,62	45,24	16,57	20,05	0,826	1,909
1232	3,3	13,72	34,82	45,08	15,7	19,12	0,821	1,885
1233		12						
1234		11,86						
1235	5,1	19,14	37,75	45,11	17,03	20,72	0,821	2,042
1236	3,5	12,72	36,55	44,35	16,21	20,34	0,796	2,014
1237	4,2	12	35,06	42,95	15,06	20	0,753	1,828
1238	4,3	21	37,14	44,02	16,35	20,79	0,786	1,894
1239	5	19,86						
1240	4,3	12	34,31	45,2	15,51	18,8	0,825	1,725
1241		13,14						
1242	4,7	21,28	33,37	44,71	14,92	18,45	0,808	1,768
1243	3,4	10,28	32,32	43,75	14,14	18,18	0,777	1,721
1244		11,86						
1245		12,72						
1246	4,3	11,57	34,33	43,78	15,03	19,3	0,778	1,765
1247	4,7	20,43	36,3	44,82	16,27	20,03	0,812	1,881
1248		11,86						
1249	4,4	14,18	31,55	42,37	13,37	18,18	0,735	1,685
1250	3,4	20,28	29,89	43,29	12,94	16,95	0,763	1,513
1251	2,9	12,28	29,18	42,8	12,49	16,69	0,748	1,484
1252	4,9	20,14	35,59	41,69	14,84	20,75	0,715	1,905
1253	4,8	12,43	33,21	40,98	13,61	19,6	0,694	1,854

1254	4,2	20,14	37,13	43,38	16,11	21,02	0,766	1,914
1255	4,2	12,14	35,65	42,49	15,15	20,5	0,739	1,876
1256		12						
1257	4,1	11,57	36,71	45,32	16,64	20,07	0,829	1,949
1258	4,4	19,57	36,71	45,54	16,72	19,99	0,836	1,949
1259		12						
1260	3,9	20,14	35,19	42,99	15,13	20,06	0,754	1,699
1261	3,8	9,72	33,73	43,01	14,51	19,22	0,754	1,678
1262		12						
1263		13,86						
1264	4,9	20,86	42,9	42,44	18,21	24,69	0,737	2,687
1265	5,6	11,86	46,17	40,97	18,92	27,25	0,694	2,67
1266	3,9	11,14	37,45	44,32	16,6	20,85	0,796	2,02
1267	4,9	21	38,41	44,64	17,15	21,26	0,806	1,991
1268	4,4	13	35,78	43,01	15,39	20,39	0,754	1,906
1269		12						
1270		12						
1271		14,72						
1272	4,4	19,57	36,79	42,89	15,78	21,01	0,751	1,862
1273	4,5	12,57	35,97	42,31	15,22	20,75	0,733	1,834
1274	3,9	20,14	30,51	42,21	12,88	17,63	0,73	1,694
1275	3,6	11,72	29,89	41,95	12,54	17,35	0,722	1,606
1276		11,72						
1277	3,3	20	30,88	43,84	13,54	17,34	0,78	1,562
1278	3,2	13	29,24	43,05	12,59	16,65	0,756	1,513
1279	5	30,72	39,26	45,95	18,04	21,22	0,85	1,936
1280	4,7	20,18	40,27	45,81	18,45	21,82	0,845	1,865
1281	3,8	11,72	35,62	43,23	15,4	20,22	0,761	1,852
1282	3,8	20	32,55	42,51	13,84	18,71	0,739	1,706
1283	3,7	13	31,11	41,49	12,91	18,2	0,709	1,635
1284		11,43						
1285	5,4	20	32,18	43,07	13,86	18,32	0,756	1,721
1286	4,2	11,43	31,04	41,14	12,77	18,27	0,698	1,714

1287	4	20,28	39,13	45,54	17,82	21,31	0,836	2,003
1288	3,7	10,72	36,41	43,77	15,94	20,47	0,778	1,911
1289	4	12,43	30,75	40,78	12,54	18,21	0,688	1,659
1290	4,4	21,28	33,13	42,25	14	19,13	0,731	1,714
1291	4,1	32,72	36,15	46,91	16,96	19,19	0,883	1,72
1292	4,3	22,72	31,92	43,2	13,79	18,13	0,76	1,628
1293	3,4	10,72	28,78	40,79	11,74	17,04	0,688	1,575
1294	3,8	20	32,38	43,69	14,15	18,23	0,776	1,737
1295	3,7	12,14	32,34	43,59	14,1	18,24	0,773	1,693
1296		12,72						
1297		12,28						
1298	3,9	20,43	30,36	42,49	12,9	17,46	0,738	1,689
1299	3,4	12,43	28,55	40,63	11,6	16,95	0,684	1,662
1300		12						
1301		11,43						
1302	4,8	21,86	35,17	42,87	15,08	20,09	0,75	1,991
1303	4,4	12,43	34,96	42,59	14,89	20,07	0,741	1,967
1304	3,4	12,28	33,51	41,39	13,87	19,64	0,706	1,553
1305	3,9	21,28	33,73	44,17	14,9	18,83	0,791	1,597
1306	3,7	35,28	37,35	45,19	16,88	20,47	0,824	1,87
1307	3,6	19,86	35,71	43,37	15,49	20,22	0,766	1,781
1308	3,1	12	34,38	43,6	14,99	19,39	0,773	1,733
1309	4	20,57	42,13	44,24	18,64	23,49	0,793	2,179
1310	4,1	12,57	39,82	42,84	17,06	22,76	0,749	2,144
1311		12,43						
1312	4,3	20,86	33,89	41,39	14,03	19,86	0,706	1,831
1313	4	10,86	33,06	40,83	13,5	19,56	0,69	1,843
1314	4,3	19,43	37,23	43,54	16,21	21,02	0,771	2,232
1315	3,5	12,72	37,18	43,67	16,24	20,94	0,775	2,214
1316	4,2	19,72	36,5	43,61	15,92	20,58	0,773	1,892
1317	4,1	13,28	34,52	41,85	14,45	20,07	0,719	1,886
1318	3,9	18,14	38,58	45,12	17,41	21,17	0,822	2,014
1319	4,1	12,14	36,96	44,53	16,46	20,5	0,802	1,977

1320	5	20,28	39,1	44,34	17,34	21,76	0,796	2,024
1321	4,5	13,28	37,42	44,28	16,57	20,85	0,794	1,936
1322	3,5	13	31,24	43,69	13,65	17,59	0,776	1,685
1323	3,5	13	30,38	42,49	12,91	17,47	0,738	1,675
1324	4,2	20,14	35,19	45,18	15,9	19,29	0,824	1,723
1325	3,9	11,14	32,9	44,13	14,51	18,37	0,789	1,677
1326	3,7	20,14	35,36	45,3	16,02	19,34	0,828	1,747
1327	3,4	12,14	34,17	44,22	15,11	19,06	0,792	1,716
1328		10,57						
1329		12,28						
1330		12,28						
1331		12,43						
1332	4	11,43	28,26	43,02	12,16	16,1	0,755	1,584
1333	4,5	20,14	29,4	43,94	12,92	16,48	0,783	1,644
1334		13,14						
1335	5,1	20,72	30	44,3	13,29	16,71	0,795	1,576
1336	4,3	12,86	29,41	43,48	12,79	16,62	0,769	1,576
1337	3,6	20	35,04	43,8	15,35	19,69	0,779	1,881
1338	3,5	12,28	33,9	43,95	14,9	19	0,784	1,818
1339	4,5	12,86	39,65	43,65	17,31	22,34	0,774	1,952
1340	4,8	20,86	40,16	43,89	17,63	22,53	0,782	1,977
1341	3,2	19,72	26,55	40,97	10,88	15,67	0,694	1,446
1342	3,2	11,72	26,65	42,92	11,44	15,21	0,752	1,376
1343	3,6	18,43	32,18	43,94	14,14	18,04	0,783	1,685
1344	3,2	13	32,14	43,34	13,93	18,21	0,764	1,672
1345	3,6	21	30,78	41,22	12,69	18,09	0,701	1,707
1346	3,3	12	31,37	39,52	12,4	18,97	0,653	1,672
1347	4	19,43	31,63	41,98	13,28	18,35	0,723	1,695
1348	3,1	10,72	30,13	40,88	12,32	17,81	0,691	1,643
1349	4,7	32,14	38,95	44,82	17,46	21,49	0,812	2,111
1350	4,5	12,28	28,69	40,92	11,74	16,95	0,692	1,709
1351	5,4	19,57	30,33	41,44	12,57	17,76	0,707	1,759
1352	3,3	12,43						

1353	3,6	32						
1354	3,8	19,86						
1355	4,5	30,57						
1356	4,4	24,28						
1357	3,8	11						
1358	3,7	12,57	37,01	43,74	16,19	20,82	0,777	1,955
1359	4,1	20,43	37,56	44,62	16,76	20,8	0,805	1,943
1360	3,6	12,28	34,31	42,11	14,45	19,86	0,727	1,806
1361	4,3	19,72	35,77	42,94	15,36	20,41	0,752	1,904
1362		12,28						
1363	3,8	12,28	31,42	43,12	13,55	17,87	0,758	1,587
1364	4,2	20,28	32,08	43,17	13,85	18,23	0,759	1,63
1365	4	20,86	32,64	42,98	14,03	18,61	0,753	1,68
1366	3,6	12,86	31,46	41,73	13,13	18,33	0,716	1,644
1367	4,3	12,14	38,23	43,94	16,8	21,43	0,783	1,954
1368	4,5	20,14	38,78	44,55	17,28	21,5	0,803	1,978
1369	5	19,86	31,96	41,77	13,35	18,61	0,717	1,77
1370	4,1	12						
1371		11,72						
1372	4,3	11,72	30,14	42,7	12,87	17,27	0,745	1,657
1373	4,4	21,72	30,2	42,74	12,91	17,29	0,746	1,696
1374	3,8	12,43	35,88	44,62	16,01	19,87	0,805	1,859
1375	4,7	20,57	38,57	46,74	18,03	20,54	0,877	1,908
1376	4,3	20,14						
1377	3,9	12,14	34,83	43,58	15,18	19,65	0,772	1,7
1378	4,7	20,14	30,56	42,76	13,07	17,49	0,747	1,585
1379	4	12,14	30,39	43,27	13,15	17,24	0,762	1,564
1380	4,4	12,57	34,66	43,24	14,99	19,67	0,762	1,823
1381		12,43						
1382	4	20,43	34,72	42,88	14,89	19,83	0,75	1,739
1383	3,9	12,86	33,77	41,72	14,09	19,68	0,715	1,707
1384	4,1	13	35,39	44,22	15,65	19,74	0,792	1,822
1385	4,8	18,86	37,31	45,99	17,16	20,15	0,851	1,859

1386	6,1	30,43						
1387	5,6	20,72						
1388	4,4	12,43						
1389	3,5	11,86	43,89	44,11	19,36	24,53	0,789	1,991
1390	4	20,28	37,1	44,17	16,39	20,71	0,791	1,991
1391	3,6	21,57	36,14	45,01	16,27	19,87	0,818	2,052
1392	3,4	11,57	33,15	42,14	13,97	19,18	0,728	2,04
1393	4,1	21,14	35,57	44,75	15,92	19,65	0,81	1,855
1394	4,1	12,14	34,47	43,98	15,16	19,31	0,785	1,767
1395		12,14						
1396	4	20,43	36,98	45,02	16,65	20,33	0,818	1,907
1397	5,1	32,86	39,65	46,93	18,61	21,04	0,884	1,966
1398	3,7	12,43	35,99	44,92	16,17	19,82	0,815	1,876
1399	4	20	31,2	43,71	13,64	17,56	0,776	1,708
1400	3,9	12,43	31,32	43,86	13,74	17,58	0,781	1,689
1401		11,28						
1402	3,7	20,43	32,54	45,32	14,75	17,79	0,829	1,714
1403	3,9	12,57	31,5	44,85	14,13	17,37	0,813	1,665
1404		12,14						
1405		11,14						
1406		12,14						
1407	4,7	19,43	34,93	42,68	14,91	20,02	0,744	1,914
1408	4,9	11,72	35,05	42,68	14,96	20,09	0,744	1,878
1409	4,2	12,28	30,89	41,34	12,77	18,12	0,704	1,709
1410	4,3	20,86	32,92	43,71	14,39	18,53	0,776	1,762
1411		11,86						
1412	5	20,28	32,02	43,94	14,07	17,95	0,783	1,704
1413	4,6	12,57	30,78	42,88	13,2	17,58	0,75	1,671
1414	3,8	11,72	31,24	41,19	12,87	18,37	0,7	1,687
1415	4,1	21,28	33,22	43,07	14,31	18,91	0,756	1,741
1416	3,9	12,72	33,98	42,28	14,37	19,61	0,732	1,778
1417	3,3	13	30,21	42,99	12,99	17,22	0,754	1,712
1418	4,1	20	30,8	44,02	13,56	17,24	0,786	1,758

1419		13						
1420	4	10,86	33,16	44,24	14,67	18,49	0,793	1,645
1421		11,43						
1422	4,5	12,14	30,72	43,03	13,22	17,5	0,755	1,669
1423	4,3	20,14	31,61	43,94	13,89	17,72	0,783	1,694
1424	4,1	13,57	31,45	41,52	13,06	18,39	0,71	1,669
1425	4,6	21,57	33,18	43,03	14,28	18,9	0,755	1,724
1426	3,1	20,72	29,42	43,09	12,68	16,74	0,757	1,636
1427	3,2	9,72	26,72	42,62	11,39	15,33	0,742	1,541
1428	4,2	19,72	35,56	44,15	15,7	19,86	0,79	1,907
1429	4	12,28	35,21	43,85	15,44	19,77	0,78	1,87
1430	4,1	13,72						
1431	4,7	19,86	32,66	44,3	14,47	18,19	0,795	1,648
1432	3,9	20,86	38,98	45,38	17,69	21,29	0,83	2,037
1433	4	12,57	36,98	44,29	16,38	20,6	0,795	1,979
1434	4,9	19,57	33,66	45	15,15	18,51	0,818	1,839
1435	4,8	12,57	34,17	44,89	15,34	18,83	0,814	1,858
1436	4	26,28	32,83	41,94	13,77	19,06	0,722	1,938
1437	4,3	12,57	34,69	43,87	15,22	19,47	0,781	1,938
1438	4,1	28,57	32,45	41,72	13,54	18,91	0,716	1,938
1439	5,2	12,43	29,85	43,35	12,94	16,91	0,765	1,785
1440	4,9	19,86	29,36	42,67	12,53	16,83	0,744	1,83
1441	4,3	11	31,26	44,01	13,76	17,5	0,786	1,64
1442	4,7	21	32,53	44,85	14,59	17,94	0,813	1,682
1443	3,8	20,43	34,8	44,13	15,36	19,44	0,79	1,721
1444	3,7	12,43	34,21	43,87	15,01	19,2	0,781	1,687
1445	4,7	22	38,91	45,05	17,53	21,38	0,819	2,068
1446	4,4	12	37,19	44,04	16,38	20,81	0,787	2,009
1447	3,9	13,43	29,92	41,77	12,5	17,42	0,717	1,573
1448	3,9	21	31,2	42,59	13,29	17,91	0,742	1,616
1449	4,5	20,14	35,4	43,02	15,23	20,17	0,755	1,917
1450	4,6	12,14	33,9	42,12	14,28	19,62	0,727	1,885
1451	4,4	12,28	27,9	41,82	11,67	16,23	0,719	1,561

1452	5,2	20,28	29,01	42,6	12,36	16,65	0,742	1,615
1453		12,28						
1454	4,5	11,14	37,41	43,59	16,31	21,1	0,772	2,031
1455	4,6	32	38,19	46,92	17,92	20,27	0,884	1,938
1456	4,2	13,86	35,06	43,55	15,27	19,79	0,771	1,866
1457	5,5	20	36,81	45,88	16,89	19,92	0,847	1,926
1458	5,2	19,86	30,88	42,97	13,27	17,61	0,753	1,563
1459	3,8	10,86						
1460	3,7	12,28	31,72	42,93	13,62	18,1	0,752	1,64
1461	3,6	18,14	32,09	43,31	13,9	18,19	0,764	1,697
1462	4,9	12,72	31,43	42,6	13,39	18,04	0,742	1,578
1463	3,7	12,86	32,9	43,31	14,25	18,65	0,764	1,801
1464	4,1	20,14	33,88	44,51	15,08	18,8	0,802	1,795
1465	3,6	20,28	32,47	43,68	13,86	18,61	0,744	1,739
1466	3,5	11,28	31,68	42,55	13,48	18,2	0,74	1,685
1467	4,1	30,86	35,19	44,52	15,67	19,52	0,802	1,782
1468	4,1	21,14	34,44	44,04	15,17	19,27	0,787	1,728
1469	4,1	14,14	35,22	43,55	15,34	19,88	0,771	1,7
1470		28						
1471		31,57						
1472		40,14						
1473		39,28						
1474	4,2	12,14	33,71	42,71	14,4	19,31	0,745	1,762
1475	5,6	20,57	36,44	44,78	16,32	20,12	0,811	1,902
1476	5,6	13,14	35,19	44,1	15,52	19,67	0,789	1,866
1477	4,7	11,72	37,86	46,01	17,42	20,44	0,852	1,955
1478	4,7	20	32,08	45,48	14,59	17,49	0,834	1,669
1479	4,5	12,72	32,65	41,28	13,48	19,17	0,703	1,87
1480	4,2	12	29,99	43,64	13,09	16,9	0,774	1,617
1481	4,1	19,72	34,83	45,65	15,9	18,93	0,839	1,763
1482	3,7	12,72	33,31	43,96	14,64	18,67	0,784	1,732
1483	3,8	13,14	32,77	45,13	14,79	17,98	0,822	1,747
1484	4,1	20,14	33,32	45,25	15,08	18,24	0,826	1,772

1485	3,5	13	35,65	44,88	16	19,65	0,814	1,788
1486	4,6	12,28	30,46	42,28	12,88	17,58	0,732	1,549
1487	4,9	18,72	30,77	42,11	12,96	17,81	0,727	1,591
1488	5,2	12,14	29,41	41,58	12,23	17,18	0,711	1,544
1489	4,2	12,43	34,35	42,64	14,65	19,7	0,743	1,968
1490	3,7	11,57	31,77	43,68	13,88	17,89	0,775	1,619
1491	3,5	21,14	32,08	43,79	14,05	18,03	0,779	1,659
1492	4,6	12,28	32,86	44,12	14,5	18,36	0,789	1,825
1493	3,8	13	32,3	42,97	13,88	18,42	0,753	1,611
1494	3,5	20,57	31,31	41,32	12,94	18,37	0,704	1,654
1495	4,7	20,28	33,85	44,84	15,18	18,67	0,813	1,825
1496	4	10,72	31,04	43,07	13,37	17,67	0,756	1,743
1497	4,6	20,57	32,65	44,41	14,5	18,15	0,798	1,814
1498	3,9	12,43	30,81	42,74	13,17	17,64	0,746	1,673
1499	4,7	20,86						
1500	4,9	13,14	32,88	43	14,14	18,74	0,754	1,824
1501	4,4	20,14	36,38	43,95	15,99	20,39	0,784	1,815
1502	4,4	20,57	34,06	43,95	14,97	19,09	0,784	1,873
1503	5,5	21,28	38,23	44,02	16,83	21,4	0,786	1,938
1504	3,5	12	30,38	43,18	13,12	17,26	0,76	1,563
1505	4,6	12,28	38,01	43,04	16,36	21,65	0,755	1,912
1506	4	12,28	28,99	41,73	12,1	16,89	0,716	1,62
1507	4,2	21,28	29,82	42,28	12,61	17,21	0,732	1,648
1508	4,1	13,57	30,98	41,28	12,79	18,19	0,703	1,677
1509	4,5	20,14						
1510	4,2	12,14	36,8	44,61	16,42	20,38	0,805	1,807
1511	4,2	11,14	34,06	41,77	14,23	19,83	0,717	1,794
1512	4,6	20,57	30,71	41,22	12,66	18,05	0,701	1,69
1513	4,2	11,43	36,24	42,52	15,41	20,83	0,739	1,956
1514	3,9	20	31,9	41,37	13,2	18,7	0,705	1,731
1515	4,1	11,86	30,99	40,36	12,51	18,48	0,676	1,688
1516	4,4	12	33,11	42,94	14,22	18,89	0,752	1,85
1517	4,3	21,43	39,34	43,95	17,29	22,05	0,784	2,019

1518	4,6	21	32,48	42,98	13,96	18,52	0,753	1,826
1519	4,2	12,57	36,41	42,84	15,6	20,81	0,749	1,852
1520	3,5	11,86	33,14	42,15	13,97	19,17	0,728	1,712
1521	4,1	18,86	34,68	43,88	15,22	19,46	0,782	1,76
1522	3,5	12,28	39,97	45,15	18,05	21,92	0,823	1,997
1523	4,4	20	33,57	41,34	13,88	19,69	0,704	1,732
1524	3,6	12	32,94	42,1	13,87	19,07	0,727	1,673
1525	3,6	11	29,39	42,32	12,44	16,95	0,733	1,563
1526	4,1	19,72	31,78	43,54	13,84	17,94	0,771	1,712
1527	5,4	19	30,09	42,77	12,85	17,24	0,745	1,606
1528	4,2	11,72	30,71	41,97	12,89	17,82	0,723	1,738
1529	4,1	11,43	34,99	44,01	15,4	19,59	0,786	1,669
1530		20,43	36,97	45,14	16,69	20,28	0,822	1,712
1531	5,2	19,72	26,69	42,2	12,53	17,16	0,73	1,581
1532	4,7	19,57	34,32	45,51	15,62	18,7	0,835	1,754
1533	4,5	12,57	33,52	45,22	15,16	18,36	0,825	1,728
1534	4,1	12,14	30,35	40,82	12,39	17,96	0,689	1,64
1535	4,7	20,14	32,15	41,74	13,42	18,73	0,716	1,654
1536	4,6	12,43	37,35	42,67	15,94	21,41	0,744	2,116
1537	4,1	19,57	34,47	43,48	14,99	19,48	0,769	1,841
1538	4,8	10	33,84	43,2	14,62	19,22	0,76	1,818
1539	3,7	19,57	32,17	43,54	14,01	18,16	0,771	1,669
1540	5,2	11,28	35,14	42,11	14,8	20,34	0,727	1,965
1541	4,9	20,28	36,53	43,2	15,78	20,74	0,76	2,026
1542	3,2		30,14	41,77	12,59	17,55	0,717	1,611
1543	4,7	22	38,65	42,92	16,59	22,06	0,752	2,149
1544	4,9	20	28,65	41,01	11,75	16,9	0,695	1,624
1545	4,1	13,14	31,29	42,79	13,39	17,9	0,748	1,635
1546	4,6	12	26,85	39,36	10,57	16,28	0,649	1,59
1547	4,1	20	28,84	41,99	12,11	16,73	0,723	1,539
1548	4,5	12,28	28,08	40,7	11,43	16,65	0,686	1,567
1549	4	11,28	34,66	42	14,56	20,1	0,724	1,796
1550	3,6	19,86	33,02	42,15	13,92	19,1	0,728	1,728

1551	3,6	11,86	33,31	41,81	13,93	19,38	0,718	1,687
1552	4,1	13	34,21	41,82	14,31	19,9	0,719	1,768
1553	4,4	20	36,64	43,28	15,86	20,78	0,763	1,796
1554	4,5	11,57	36,43	45,92	16,73	19,7	0,849	1,825
1555	4,9	19,57	36,62	45,49	16,66	19,96	0,834	1,861
1556	3,9	11,86	32,18	41,79	13,45	18,73	0,718	1,721
1557	4,2	19,86	33,57	43,13	14,48	19,09	0,758	1,741
1558	4	12,28	31,78	41,59	13,22	18,56	0,712	1,725
1559	4,4	20,43	33,59	41,7	14,01	19,58	0,715	1,775
1560	3,6	21	34,88	44,35	15,47	19,41	0,797	1,861
1561	3,9	13,14	29,33	41,11	12,06	17,27	0,698	1,572
1562	4	20,86	35,22	44	15,5	19,72	0,786	1,791
1563	3,3	12,28	34,73	43,88	15,24	19,49	0,781	1,724
1564	4	19,14	30,87	42,3	13,06	17,81	0,733	1,587
1565	3,5	20,57	39,05	44,12	17,23	21,82	0,789	1,899
1566	3,5	11,72	37,56	44,19	16,6	20,96	0,791	1,833
1567	3,2	22	39,61	46,45	18,4	21,21	0,867	1,956
1568	3	12,43	37,72	46,07	17,38	20,34	0,854	1,845
1569	2,8	12,43	32,88	42,18	13,87	19,01	0,729	1,741
1570	4,3	14	30,23	40,72	12,31	17,92	0,686	1,751
1571	4	11,57	36,46	44,37	16,18	20,28	0,797	1,778
1572	4,1	21	32,41	42,73	13,85	18,56	0,746	1,668
1573	4,7	12,28	32,7	42,93	14,04	18,66	0,752	1,688
1574	4,8	20,57	31,04	41,55	12,9	18,14	0,711	1,776
1575	3,5	10	32,9	43,19	14,21	18,69	0,76	1,64
1576	4,2	19,57	34,58	43,2	14,94	19,64	0,76	1,818
1577	5,2	20,28	33,12	43,84	14,52	18,6	0,78	1,741
1578	3,7	21,14	39,95	44,6	17,82	22,13	0,805	1,918
1579	3,7	13,14	38,28	42,58	16,3	21,98	0,741	2,109
1580	4,6	20,43	38,94	43,47	16,93	22,01	0,769	2,151
1581	4,6	12,72	39,08	43,42	16,97	22,11	0,767	2,173
1582	3,7	20,28	37,02	45,62	16,89	20,13	0,839	1,895
1583	3,8	20,57	33,65	42,97	14,46	19,19	0,753	1,724

1584	4,1	11,57	33,35	42,81	14,28	19,07	0,748	1,683
1585	3,7	20,28	34,01	45,95	15,63	18,38	0,85	1,675
1586	3,8	13,57	36,73	45,65	16,77	19,96	0,84	1,833
1587	3,3	12,57	32,89	45,75	15,05	17,84	0,843	1,567
1588	5,5	11,86	30,45	42,69	13	17,45	0,744	1,656
1589	4,1	19,86	30,68	42,5	13,04	17,64	0,739	1,694
1590	3,7	12,72	34,22	40,38	13,82	20,4	0,677	1,769
1591	4,1	20,14	36,43	41,88	15,26	21,17	0,72	1,852
1592	4,7	12,28	37,13	44,54	16,54	20,59	0,803	1,82
1593	4,8	20,14	37,82	44,71	16,91	20,91	0,808	1,87
1594	3,9	12,28	29,64	41,9	12,42	17,22	0,721	1,563
1595	5	20,28	31,22	42,79	13,36	17,86	0,748	1,605
1596	3,3	12	30,55	40,52	12,38	18,17	0,681	1,738
1597	4,3	19,86	34,56	42,7	14,76	19,8	0,745	1,991
1598	4	14,28	28,24	42,17	11,91	16,33	0,729	1,51
1599	4,5	11,72	32,35	41,05	13,28	19,07	0,696	1,644
1600	4,3	20,72	33,26	42,63	14,18	19,08	0,743	1,712
1601	4,8	20,14	34,18	42,94	14,68	19,5	0,753	1,812
1602	3,6	10,72	32,68	42,59	13,92	18,76	0,742	1,706
1603	4,3	19,72	27,83	41,75	11,62	16,21	0,716	1,539
1604	3,9	12,43	44,06	45,77	20,17	23,89	0,844	2,374
1605	3,2	12,57	36,74	43,08	15,83	20,91	0,757	2,045
1606	4	20,86	36,61	44,08	16,14	20,47	0,788	1,973
1607	5,6	20,14	38,28	43,67	16,72	21,56	0,775	2,155
1608	4,3	20,14	36,95	44,49	16,44	20,51	0,801	2,067
1609	3,3	20,43	35,34	44,28	15,65	19,69	0,794	1,683
1610	4,1	20,72	35,95	44,81	16,11	19,84	0,811	1,837
1611	4,2	12,72	36,02	44,69	16,1	19,92	0,808	1,812
1612	4,3	20,43	30,33	42,99	13,04	17,29	0,754	1,714
1613	3,6	12,86	37	44,4	16,43	20,57	0,798	1,979
1614	2,9	12,57	34,87	44,04	15,36	19,51	0,787	1,654
1615	4,8	13,57	38,66	43,35	16,76	21,9	0,765	2,155
1616	4,1	11,72	29,4	42,44	12,48	16,92	0,737	1,675

1617	4,3	12,28	32,9	43,67	14,37	18,53	0,775	1,615
1618	4,8	22,28	32,83	43,74	14,36	18,47	0,777	1,688
1619	3,7	22	33,33	42,3	14,1	19,23	0,733	1,805
1620	3,4	13,14	32,17	42,05	13,53	18,64	0,725	1,738
1621	3,7	11,72	36,79	45,01	16,56	20,23	0,818	1,95
1622	4,1	20,28	36,97	45,79	16,93	20,04	0,844	1,996
1623	4	12,28	35,66	44,61	15,91	19,75	0,805	1,858
1624	3,9	12,28	29,06	40,98	11,91	17,15	0,694	1,567
1625	4,5	21,72	37,46	44,95	16,84	20,62	0,816	1,883
1626	4,2	20,86	32,81	41,84	13,73	19,08	0,719	1,802
1627	3,8	11,86	31,46	43,48	13,68	17,78	0,769	1,665
1628	4,4	11,86	33,06	42,8	14,15	18,91	0,748	1,789
1629	4,4	21,14	30,63	42,47	13,01	17,62	0,738	1,646
1630	4,4	13,14	30,48	42,65	13	17,48	0,743	1,619
1631	3,7	11,57	34,86	44,14	15,39	19,47	0,79	1,823
1632	4,3	20,28	32,2	40,71	13,11	19,09	0,686	1,836
1633	4,1	12,28	31,26	39,79	12,44	18,82	0,66	1,823
1634	3,9	19,86	31,57	41,74	13,18	18,39	0,716	1,645
1635	4,3	20,57	38,9	45,98	17,89	21,01	0,851	1,924
1636	3,6	11,86	30,78	41,09	12,65	18,13	0,697	1,616
1637	4	12,57	28,36	40,55	11,5	16,86	0,682	1,557
1638	5,1	12	33,15	44,52	14,76	18,39	0,802	1,772
1639	5,3	22	33,1	44,22	14,64	18,46	0,793	1,844
1640	4,8	12,57	29,95	41,13	12,32	17,63	0,698	1,663
1641	4,2	12,15	34,16	44,43	15,18	18,98	0,799	1,726
1642	4,8	19,57	31,93	42,87	13,69	18,24	0,75	1,69
1643	4,4	20,43	33,4	44,67	14,92	18,48	0,807	1,838
1644	4,4	20,72	37,19	44,44	16,53	20,66	0,8	2,002
1645	3,9	12,57	36,12	43,79	15,82	20,3	0,779	2,002
1646	4,2	12,57	32,75	44,94	14,72	18,03	0,816	1,802
1647	4,5	20,14	33,3	44,47	14,81	18,49	0,8	1,826
1648	3,9	12,14	31,62	43,95	13,9	17,72	0,784	1,727
1649	4,9	21,72	30,13	43,31	13,05	17,08	0,764	1,672

1650	4,8	12,28	28,27	42,02	11,88	16,69	0,724	1,607
1651	3,7	19,72	34,8	42,35	14,74	20,06	0,734	1,872
1652	4,3	11,86	34,24	41,85	14,33	19,91	0,719	1,834
1653	3,8	20,28	41,48	43,82	18,18	23,3	0,78	2,236
1654	4,4	20	35,22	42,75	15,06	20,16	0,747	1,815
1655	5	20,28	34,47	42,64	14,7	19,77	0,743	1,932
1656	4,7	13,57	36	42,72	15,38	20,62	0,745	1,865
1657	3,7	11,86	36,64	43,8	16,05	20,59	0,779	1,802
1658	3,8	12,28	33,77	42,4	14,32	19,45	0,736	1,925
1659	4,8	19,57	35,4	42,51	15,05	20,35	0,739	1,878
1660	3,6	12,28	41,32	44,04	18,2	23,12	0,787	2,218
1661	3,9	12,86	35,02	43,91	15,38	19,64	0,783	1,914
1662	4	11,57	37,77	44,55	16,83	20,94	0,803	1,886
1663	4,7	19,28	37,74	44,35	16,74	21	0,797	1,927
1664	5,4	19,86	37,78	42	15,87	21,91	0,724	2,129
1665	4,8	11,86	37,51	41,77	15,67	21,84	0,717	2,14
1666	4,9	20,14	39,85	46,19	18,41	21,44	0,858	1,965
1667	3,4	12,14	37,89	44,44	16,84	21,05	0,8	1,889
1668	3,3	12,86	34,48	42,48	14,65	19,83	0,738	1,806
1669	3,6	19,72	35,65	43,14	15,38	20,27	0,758	1,833
1670	3,3	11,72	35,94	44,07	15,84	20,1	0,788	1,702
1671	3,2	21,72	36,07	43,85	15,82	20,25	0,781	1,758
1672	3,8	20,14	30,05	42,32	12,72	17,33	0,733	1,659
1673	3,7	11,72	30,24	41,83	12,65	17,59	0,719	1,646
1674	4,6	13,14	34,74	42,51	14,77	19,97	0,739	2,052
1675	5	21,14	38,86	45,05	17,51	21,35	0,82	2,095
1676	4,3	12,14	31,14	39,65	12,35	18,79	0,657	1,794
1677	4,5	12,57	32,69	42,7	13,96	18,73	0,745	1,754
1678	5	20,57	33,35	43,17	14,4	18,95	0,759	1,794
1679	4,3	12,14	37,54	44,45	16,69	20,85	0,8	2,048
1680	4,4	20	38,11	44,71	17,04	21,07	0,808	2,059
1681	4,2	20	29,65	43,6	12,93	16,72	0,773	1,481
1682	3,8	12,43	29,86	43,7	13,05	16,81	0,776	1,466

1683	4,7	21,28						
1684	4,8	12,86	29,83	41,43	12,36	17,47	0,707	1,646
1685	4,6	11,72	32,52	42,8	13,92	18,6	0,748	1,862
1686	4,9	19,72	36,47	44,03	16,06	20,41	0,786	1,926
1687	4,8	19,28	31,37	43,22	13,56	17,81	0,761	1,634
1688	4,1	12,72	31,97	43,44	13,89	18,08	0,768	1,625
1689	5	22,14	36	42,61	15,34	20,66	0,742	2,149
1690	4,4	12,14	34,87	41,12	14,34	20,53	0,698	2,138
1691	4,2	9,28	30,94	41,43	12,82	18,12	0,707	1,788
1692	5	20,28	29,75	40,13	11,94	17,81	0,67	1,795
1693	5,9	21,57	34,68	44,78	15,53	19,15	0,81	1,872
1694	4,7	12,72	34,36	45,6	15,67	18,69	0,838	1,836
1695	4,7	19,57	37,33	45,51	16,99	20,34	0,835	1,802
1696	4,6	13,14	36,2	44,3	16,04	20,16	0,795	1,749
1697	4	12	40,08	46,15	18,5	21,58	0,857	2,076
1698	4,3	20,28	33,62	44,22	14,87	18,75	0,793	1,714
1699	3,9	12,57	32,26	42,8	13,81	18,45	0,748	1,673
1700	2,7	26,14	35,19	44,67	15,72	19,47	0,807	1,818
1701	3	21,43						
1702	3,4	32,86	36,49	45,73	16,69	19,8	0,842	1,837
1703	4,3	26,43						
1704	4,1	30,86	54,12	53,82	29,13	24,99	1,165	2,33
1705	4,3	22,86	37,49	44,27	16,6	20,89	0,794	2,032
1706	3,3	21,28	35,27	44,34	15,64	19,63	0,796	1,78
1707	3,4	27,57	41,47	46,05	19,1	22,37	0,853	1,972
1708	2,7	29,43	40,27	46,61	18,77	21,5	0,873	1,967
1709	3,8	31,14	44,04	52,24	23,01	21,03	1,094	2,048
1710	4,4	31,28						
1711	3,7	20,86	37,88	43,5	16,48	21,4	0,77	
1712	3,4	26,72	39,23	45,75	17,95	21,28	0,843	2,002
1713	4,7	26,57						
1714	2,4	29,72						
1715	3,2	26,57	50,48	51,28	25,89	24,59	1,052	2,114

1716	3,9	27,28	47,23	49,05	23,17	24,06	0,963	2,176
1717	3,1	31,14						
1718	4,2	31						
1719	3,2	30,43	37,44	46,98	17,59	19,85	0,886	1,872
1720	3,1	31						
1721	4,4	32,86						
1722	5	30,14	39,84	47,84	19,06	20,78	0,917	1,899
1723	5	29,72	28,95	42,9	12,42	16,53	0,751	1,663
1724	4,7	25,14	49,92	46,29	23,11	26,81	0,86	
1725	2	29,57						
1726	4,8	30,43	37,85	46,28	17,52	20,33	0,861	1,907
1727	2,5	31,57						
1728	3	31,72	47,34	45,24	21,42	25,92	0,826	2,154
1729	2,8	28,28	44,24	50,18	22,2	22,04	1,007	2,014
1730	2,9	31,28						
1731	3,9	25,57						
1732	3,8	29,86	43,02	48,83	21,01	22,01	0,954	1,877
1733	3,5	22,86	39,09	45,91	17,95	21,14	0,849	1,865
1734	2,9	32,57						
1735		12,72						
1736	2,8	27,57	35,55	46,01	16,36	19,19	0,852	1,819
1737	4,2	30,57	38,54	45,45	17,52	21,02	0,833	2,072
1738	4,3	20,28	34,62	42,83	14,83	19,79	0,749	1,979
1739	4,6	13,28	35,13	42,89	15,07	20,06	0,751	1,955
1740	4,4	28,57	43,85	46,33	20,32	23,53	0,863	2,157
1741	3,9	20	40,94	43,62	17,86	23,08	0,77	
1742	3,5	13	38,72	43,2	16,73	21,99	0,76	
1743	3,8	34,28						
1744	5,9	32,72	67,23	55,89	37,58	29,65	1,267	2,915
1745	3,4	26,57	40,43	50,11	20,26	20,17	1,004	1,967
1746	3,8	12,28	35,79	43,72	15,65	20,14	0,777	1,854
1747	3,4	26,57	35,18	42,63	15	20,18	0,743	1,93
1748	3,2	32,86						

1749	3,4	28,57						
1750	3,1	32,72						
1751	3,9	20,86	34,46	44,57	15,36	19,1	0,804	1,834
1752	3,5	12,86	33,92	44,51	15,1	18,82	0,802	1,77
1753		10,72						
1754	3,1	26						
1755	3,7	14,57						
1756	3,4	13,28	37,6	43,72	16,44	21,16	0,78	
1757	3,5	21,28	42,93	44,37	19,05	23,88	0,8	
1758		32,57						
1759		26,57						
1760		24,14						
1761		31,72						
1762		33,28						
1763		32,57						
1764		26,14						
1765		31,72						
1766		32,72						
1767		30,43						
1768		31,28						
1769		33,14						
1770		32,86						
1771		33,57						
1772		25,28						
1773		31,28						
1774		27,57						
1775		27,28						
1776		32,14						
1777		32,86						
1778		27,28						
1779		22,14						
1780		33,57						
1781		32,28						

1782		31,14						
1783		26,72						
1784		30,28						
1785	4,2	13,43	33,63	43,02	14,47	19,16	0,755	1,96
1786	4,2	20,86	36,37	44,59	16,22	20,15	0,804	1,993
1787	4	34,28	35,23	44,05	15,52	19,71	0,787	1,774
1788	3,2	37,86	41,85	48,98	20,5	21,35	0,96	2,058
1789	2,9	33,28	36,15	45,72	16,53	19,62	0,842	1,792
1790	3,6	35,14	39,16	44,15	17,29	21,87	0,79	1,81
1791	3,6	36,28						
1792	3	38,57	33,09	43,78	14,49	18,6	0,779	1,756
1793	3,3	39,14	41,29	47,51	19,62	21,67	0,905	1,95
1794	3,5	34,43	38,99	46,49	18,13	20,86	0,869	1,902
1795	3,7	34,43	38,68	47,31	18,3	20,38	0,897	1,874
1796	4,2	39,43						
1797	3,6	34	33,73	45,24	15,26	18,47	0,826	1,754
1798	3,1	36,72	35,66	46,71	16,66	19	0,876	1,649
1799	2,9	36						
1800	3,8	39,43	36,52	44,93	16,41	20,11	0,816	1,709
1801	4,9	38	43,09	48,59	20,94	22,15	0,945	2,047
1802	3,3	32,43	34,7	47,49	16,48	18,22	0,904	1,76
1803	4,2	35						
1804	3,8	39,28	38,48	46,98	18,08	20,4	0,886	1,856
1805	4,1	33,72						
1806	3,1	37,28	43,06	48,65	20,95	22,11	0,947	1,979
1807	4,3	37						
1808	3,8	37,86	44,32	54,98	24,37	19,95	1,221	1,872
1809	5,1	35,43						
1810	3,6	39,72	41,7	50	20,85	20,85	1	1,789
1811	3,8	37,72	36,06	45,17	16,29	19,77	0,823	1,842
1812	5,3	29						
1813	4,1	39	33,46	43,6	14,59	18,87	0,773	1,755
1814	3,1	38,14	43,11	49,38	21,29	21,82	0,975	1,932

1815	3,8	37,57	38,3	48,19	18,46	19,84	0,93	1,876
1816	2,6	37,57						
1817	4,8	37,57	43,02	48,44	20,84	22,18	0,939	1,903
1818	3,2	38,72	37,74	47,72	18,01	19,73	0,912	1,717
1819	4,5	32,72	35,73	42,12	15,05	20,68	0,727	2,007
1820	3,6	38,57	47,36	52,42	24,83	22,53	1,102	2,155
1821	4	36,57						
1822	4,8	38,28	44,03	48,42	21,32	22,71	0,938	2,026
1823	4,1	27	31,78	41,12	13,07	18,71	0,698	1,776
1824	3,4	38,28						
1825	4	38						
1826	3,8	37,43						
1827	3,2	34,86						
1828	4,3	39,72						
1829	2,7	38,57						
1830	4,3	38,14						
1831	3,9	39,86						
1832	3,8	35,86						
1833	4,6	20,28	39,23	44,09	17,3	21,93	0,788	2,151
1834	5,4	13,28	40	44,62	17,85	22,15	0,805	2,119
1835	3,5	38,43						
1836	3,3	39,14	37,89	47,98	18,18	19,71	0,922	1,861
1837	3,5	33,72	37,15	45,92	17,06	20,09	0,849	1,911
1838	4,1	35,72	34,84	43,88	15,29	19,55	0,782	1,895
1839	3,4	13,72	39,62	43,23	17,13	22,49	0,76	
1840	3,5	20,72	39,03	43,14	16,84	22,19	0,76	
1841	4,3	19,43	32,91	44,02	14,49	18,42	0,786	1,849
1842	4,1	36,57	39,04	47,92	18,71	20,33	0,92	1,89
1843	3,6	22,14	37,8	47,61	18	19,8	0,909	1,778
1844	4,6	32,86	37,86	48,57	18,39	19,47	0,944	1,867
1845	3,2	34,14	51,44	49,65	25,54	25,9	0,986	2,301
1846	4	36,72	48,39	49,24	23,83	24,56	0,97	2,301
1847	3,4	37						

1848	4,4	27	40,85	45,75	18,69	22,16	0,843	1,977
1849	3,6	33,72	46,89	49,84	23,37	23,52	0,993	
1850	3,1	35	34,33	44,27	15,2	19,13	0,794	1,805
1851	3,1	21	30,83	42,97	13,25	17,58	0,75	
1852	4,8	23,72	33,68	42,22	14,22	19,46	0,73	
1853	4,7	40,43	54,26	54,99	29,84	24,42	1,221	2,239
1854	3,8	39,28	32,45	45,63	14,81	17,64	0,839	1,687
1855	3,9	36,86	38,7	45,94	17,78	20,92	0,849	1,851
1856	3,6	33,86	34,25	47,53	16,28	17,97	0,905	1,63
1857	3,9	34,43	45,52	50,48	22,98	22,54	1,019	2,138
1858	4	37,43						
1859	3,6	36,57	50,49	50,12	25,31	25,18	1,005	1,802
1860	3,8	24,43	40,28	46,17	18,6	21,68	0,857	1,748
1861	3,6	12,57	40,1	47,08	18,88	21,22	0,889	1,664
1862	2,5	35,28						
1863	4	38,57	33,96	45,87	15,58	18,38	0,847	1,712
1864	3,6	40						
1865	4,3	30,72	34,24	43,98	15,06	19,18	0,785	1,798
1866	3,4	39						
1867	4,4	24,28						
1868	3,5	37,28	43,43	50,25	21,82	21,61	1,009	1,99
1869	3,5	36	43,28	50,48	21,85	21,43	1,019	1,926
1870	5,9	37,57	55,39	46,45	25,73	29,66	0,867	1,938
1871	4,1	30,72	37,87	44,04	16,68	21,19	0,787	1,985
1872	3,9	38,86	46,04	46,85	21,57	24,47	0,881	2,137
1873	4,7	33,43						
1874	3,7	34	38,97	49,63	19,11	19,86	0,962	1,909
1875	3,3	34,14	36,89	40,98	15,12	21,77	0,694	1,76
1876	4,2	19,43	30,25	41,22	12,47	17,78	0,701	1,653
1877	4	10,86	29,73	41,43	12,32	17,41	0,71	
1878	4,9	38,86	58,22	51,78	30,15	28,07	1,074	2,58
1879	3,8	36,72	39,51	45,3	19,7	21,61	0,828	1,983
1880	4,5	36,57	34,23	44,02	15,07	19,16	0,786	1,728

1881	4	35						
1882		36,57	61,63	49,05	30,23	31,4	0,962	2,364
1883	3,7	39,14	47,11	50,64	23,86	23,25	1,026	2,194
1884	3,8	37	40,03	45,14	18,07	21,96	0,822	2,157
1885	2,7	22,14						
1886		10,43						
1887	3,9	38,57						
1888		11,72						
1889	3,2	19,86	31,36	43,04	13,5	17,86	0,755	1,638
1890	3,3	34,28	37,57	47,1	17,7	19,87	0,89	1,775
1891	3,4	10,86	30,76	42,19	12,98	17,78	0,73	1,648
1892		11,14						
1893		11,72						
1894	4,3	22,43	32,17	43,82	14,1	18,07	0,78	1,63
1895	3,9	39,86	40,76	49,68	20,25	20,51	0,987	1,791
1896	3,6	13,43	31,7	42,8	13,57	18,13	0,748	1,609
1897	4	20,72	39,73	46,33	18,41	21,32	0,863	1,973
1898	3,6	12,28	37,77	44,55	16,83	20,94	0,803	1,938
1899	4,3	40,43	37,67	43,69	16,46	21,21	0,776	2,062
1900	4,5	20,86	36,87	43,47	16,03	20,84	0,769	1,955
1901	4,8	12,86	34,46	42,19	14,54	19,92	0,729	1,905
1902	3,6	29	31,8	42,61	13,55	18,25	0,742	1,791
1903	3,2	11,72	30,78	41,97	12,92	17,86	0,723	1,712
1904	3,3	20,28	30,76	42,23	12,99	17,77	0,731	1,712
1905		10,86						
1906	3,5	11,72	33,39	43,33	14,47	18,92	0,764	1,74
1907	3,5	22,72	35,99	45,15	16,25	19,74	0,823	1,776
1908	3,4	36,57	44,7	51,85	23,18	21,52	1,077	1,947
1909	3,2	36,28	51,97	47,04	24,45	27,52	0,888	1,843
1910	3,9	11,14	30,52	42,72	13,04	17,48	0,745	1,596
1911	4,1	20,14	33,5	43,28	14,5	19	0,763	1,651
1912	3,5	24,14	36,35	44,29	16,1	20,25	0,795	1,844
1913	2,9	34,57	35,85	46,79	18,18	20,67	0,879	1,917

1914	3,6	10						
1915	4,2	11,57	24,4	39,5	9,64	14,76	0,653	1,428
1916	3,4	37,14	37,51	46,33	17,38	20,13	0,863	1,878
1917	3,7	21,14	33,82	44,08	14,91	18,91	0,788	1,778
1918	3,4	13,72						
1919		11,72						
1920	4,4	21	33,28	43,84	14,59	18,69	0,78	1,728
1921	3,8	11,57	31,74	42,5	13,49	18,25	0,739	1,661
1922	5,3	11,57	43,15	43,82	18,91	24,24	0,78	2,299
1923	4,7	32,14						
1924	4,9	20,72						
1925	4,4	12,28						
1926	3,9	38	49,02	49,12	24,08	24,94	0,965	2,271
1927	3,7	12,72	38,88	43,23	16,81	22,07	0,761	2,13
1928	3,4	20,43	39,8	45,87	18,26	21,54	0,847	2,014
1929	3,4	11,43	38,38	44,05	16,91	21,47	0,787	1,965
1930	4,3	12						
1931	4,2	19,28						
1932	6,4	32,14						
1933		11,43						
1934		12,86						
1935		12,72						
1936		35,86						
1937		36,72						
1938		38,72						
1939		36,57						
1940		33,86						
1941		39,28						
1942		35,57						
1943		36,86						
1944		36,57						
1945		37,14						
1946		38,43						

1947		33,72						
1948		37,43						
1949		37,86						
1950		39,72						
1951		33,57						
1952		34,86						
1953		38,57						
1954		38,86						
1955		38,14						
1956		40,57						
1957		35,72						
1958		38,28						
1959		36,28						
1960		37,72						
1961		33,72						
1962		40,28						
1963		36,72						
1964		40						
1965		35,86						
1966		33,72						
1967		35						
1968		32,72						
1969		38,28						
1970		39						
1971		38						
1972		38,14						
1973		36						
1974		40,43						
1975		39,14						
1976		40,43						
1977		40						
1978		37,57						
1979		35,57						

1980		37,72						
1981		35,72						
1982		37,57						
1983		30,57						
1984		34,86						
1985		36,28						
1986		34,28						
1987		38,86						
1988		36,14						
1989	5,7	20,72	35,82	43,99	15,76	20,06	0,785	1,878
1990	4,5	11,28	35,85	43,62	15,64	20,21	0,773	1,841
1991	4,2	22	34,08	42,89	14,62	19,46	0,751	1,879
1992	4,3	13	33,46	41,9	14,02	19,44	0,721	1,842
1993	4,2	11,43	33,08	42,53	14,07	19,01	0,74	1,783
1994	4,2	20						
1995	3,5	19,86	38,01	44,61	16,96	21,05	0,805	1,973
1996	3,9	11,14	37,29	43,84	16,35	20,94	0,78	1,973
1997	4,9	10,57	33,21	42,33	14,06	19,15	0,734	1,741
1998	4	12,28	39,35	44,11	17,36	21,99	0,789	2,066
1999	4,1	21,14	30,99	43,14	13,37	17,62	0,758	1,639
2000	4,3	13,86	31,57	43,49	13,73	17,84	0,769	1,598
2001	4,3	20,43	35,14	41,57	14,61	20,53	0,711	1,881
2002	3,6	12,43	35,71	42,22	15,08	20,63	0,73	1,835
2003	4,3	20,57	38,34	42,61	16,34	22	0,742	2,224
2004	4,4	11,86	38,45	42,41	16,31	22,14	0,736	2,224
2005	3,4	25,86	33,39	42,31	14,14	19,25	0,734	1,688
2006	4	35,57	27,08	39,18	10,61	16,47	0,644	1,578
2007	5	27,72						
2008	4,6	35						
2009	3,4	36,57	29,69	42,13	12,51	17,18	0,728	1,532
2010	3,7	38,86	35,68	44	15,7	19,98	0,785	1,858
2011	3,5	37						
2012	4,7	28,14	46,37	46,19	21,42	24,95	0,858	2,287

2013	3,9	40,72	32,49	43,89	14,26	18,23	0,782	1,838
2014	3,9	38,14						
2015	5,3	40,43						
2016	3,3	38,28	35,43	46,28	16,4	19,03	0,861	1,896
2017	3,5	40,43						
2018	3,2	37,72	30,76	42,06	12,94	17,82	0,826	1,699
2019	3	38	39,88	45,73	18,24	21,64	0,842	1,95
2020	3,6	35,86						
2021	4,9	36,72						
2022	3,3	39,14	32,49	44,75	14,54	17,95	0,81	1,632
2023	3,1	35,86						
2024	3,8	40	40,86	46,74	19,1	21,76	0,877	2,097
2025	3,6	33	36,17	46,33	16,76	19,41	0,863	1,909
2026	4,6	37,72	38,56	47,61	18,36	20,2	0,908	1,885
2027	4,9	36,86						
2028	3,7	36						
2029	3,8	37	36,03	44,21	15,93	20,1	0,792	1,866
2030	3,6	39,43	37,01	44,9	16,62	20,39	0,815	2,069
2031	4,2	39,72						
2032	4,5	37,86	42,42	50,02	21,22	21,2	1	2,032
2033	2,8	35,28						
2034	5,2	34,86						
2035	3,5	39,72						
2036		32,14						
2037	4,9	21,86						
2038	3,6	33,43	32,06	42,63	13,67	18,59	0,743	1,699
2039	2,8	35,28	41,36	46,08	19,06	22,3	0,854	2,003
2040	2,7	39,72						
2041	3,1	39						
2042	3,3	38,86						
2043	4,6	37,28						
2044	3,2	34,72	41,55	47,05	19,55	22	0,888	2,038
2045	3,9	38,43	32,03	43,7	14	18,03	0,776	1,667

2046	4,6	37,86	49,49	47,78	23,65	25,84	0,915	2,353
2047	3,3	38,28						
2048	4,5	38	41,46	49,15	20,38	21,08	0,966	2,028
2049	4,7	19,86						
2050	4,7	38	52,48	46,34	24,32	28,16	0,863	2,621
2051	4,3	38,86	58,42	47,55	27,78	30,64	0,906	2,435
2052	4,1	40,28						
2053	4	30,43	39,38	43,19	17,01	22,37	0,76	2,224
2054	4,8	24,43	40,32	44,19	17,82	22,5	0,792	2,203
2055	4,2	19,57	36,63	46,13	16,9	19,73	0,856	2,088
2056	4,3	34,86	47,41	45,89	21,76	25,65	0,848	2,611
2057	3,3	36,72	46,93	52,22	24,51	22,42	1,093	2,102
2058	3,7	32,14						
2059	4,1	34,14	36,01	44,7	16,1	19,91	0,808	1,865
2060	3,9	20	34,41	43,82	15,08	19,33	0,78	1,784
2061	3,7	12	33,16	43,24	14,34	18,82	0,761	1,749
2062	2,8	26,43						
2063	3,3	34,14	43,15	44,95	19,4	23,75	0,816	2,053
2064	4	35,14						
2065	4,2	33,57	36,02	44,19	15,92	20,1	0,792	1,87
2066	2,8	38						
2067	3,2	40,14	38,47	48,99	18,85	19,62	0,96	1,85
2068	5,7	35,28						
2069	3,2	35,28	39,08	49,12	19,2	19,88	0,965	1,861
2070	2,6	33,14						
2071	3,5	36,72	36,62	47,7	17,47	19,15	0,912	1,761
2072	3,6	19	31,51	41,57	13,1	18,41	0,711	1,694
2073	4,6	30	32,35	41,73	13,5	18,85	0,716	1,825
2074	3,2	40	47,94	47,78	22,91	25,03	0,915	2,34
2075	4,3	32,86	40,49	44,28	17,93	22,56	0,794	2,165
2076	3,1	37,28	39,07	45,94	17,95	21,12	0,849	1,996
2077		12,72						
2078	4	41						

2079	3,8	36,28	42,44	50,61	21,48	20,96	1,024	1,926
2080	3,7	24,86	43,35	48,28	20,93	22,42	0,933	2,134
2081	3,6	34,14	39,98	43,39	17,35	22,63	0,766	2,258
2082	4,6	20,86	36,99	43,3	16,02	20,97	0,763	1,881
2083	4,2	31,28	35,1	44,27	15,54	19,56	0,794	1,811
2084	4,4	18,57	32,14	42,75	13,74	18,4	0,746	1,767
2085	4,2	10,57	30,64	42,19	12,93	17,71	0,73	1,695
2086	3,3	12,14	39,84	44,52	17,74	22,1	0,802	2,15
2087	3,9	34,14						
2088	3,9	12,43	33,41	42,71	14,27	19,14	0,745	1,687
2089	4,4	20,43	34,69	43,35	15,04	19,65	0,765	1,768
2090	4,8	20,43	36,1	45,78	16,53	19,57	0,844	1,774
2091	3,8	11,43	33,26	43,68	14,53	18,73	0,775	1,71
2092		12,28						
2093	5,4	32,72	35,55	44,33	15,76	19,79	0,796	1,783
2094	4,7	19,57	32,51	42,23	13,73	18,78	0,731	1,688
2095	3,4	8	30,13	41,25	12,43	17,7	0,7	
2096	4,5	19,57	39,6	43,53	17,24	22,36	0,771	2,261
2097	5,2	11,57	39,94	43,28	17,29	22,65	0,763	2,258
2098	5,1	20,72	33,02	43,42	14,34	18,68	0,767	1,837
2099	4,3	12,72	32,23	43,25	13,94	18,29	0,762	1,736
2100	4,5	39,28	41,75	47,42	19,8	21,95	0,902	2,094
2101	4,7	21,57	38,84	47,28	18,84	21	0,897	1,985
2102	5,4	11,86	37,84	45,34	17,16	20,68	0,829	1,979
2103		13,14						
2104		12,72						
2105		11,43						
2106	4,9	33,43						
2107	4,3	18						
2108	4,6	12	33,81	44,48	15,04	18,77	0,801	1,731
2109		12,72						
2110		11,86						
2111	4,2	18,43	37,29	44,08	16,44	20,85	0,788	1,925

2112	4,4	13,57	35,43	42,42	15,03	20,4	0,736	1,901
2113	3,4	12,28	33,71	45,83	15,45	18,26	0,846	7,796
2114	4	33,28	34,75	46,18	16,05	18,7	0,858	1,859
2115	4	20	33,71	45,17	15,23	18,48	0,824	1,802
2116		12,14						
2117	3,7	12,28	28,28	41,05	11,61	16,67	0,696	1,549
2118	3,3	20,72	27,94	40,73	11,38	16,56	0,687	1,549
2119	5,1	20,43	32,09	44,99	14,44	17,65	0,818	1,707
2120	3,8	12,43	32,54	45,82	14,91	17,63	0,845	1,668
2121	3,6	20,57	38,94	42,91	16,71	22,23	0,751	2,269
2122	3,6	12,43	40,35	44,11	17,8	22,55	0,789	2,279
2123	3,7	38,57	39,48	48,12	19	20,48	0,927	1,936
2124	4,3	12,14	29,66	40,39	11,98	17,68	0,677	1,668
2125	5,1	21,86	31,78	41,44	13,17	18,61	0,707	1,749
2126	5,5	20,14	29,32	42,29	12,4	16,92	0,732	1,64
2127	4,3	12,72	30,13	43,51	13,11	17,02	0,77	1,621
2128		10,72						
2129	4,6	20,72	34,56	44,35	15,33	19,23	0,797	1,805
2130	4,3	11,72	33,59	43,61	14,65	18,94	0,773	1,767
2131	4,6	20,72	29,36	43,69	12,83	16,53	0,776	1,601
2132	4,5	13,14	27,91	42,63	11,9	16,01	0,743	1,583
2133	4,4	35,86	38,88	45,13	17,55	21,33	0,822	1,991
2134	4,2	13,86	35,16	43,11	15,16	20	0,758	1,863
2135	4,9	20,57	36,54	44,77	16,36	20,18	0,81	1,906
2136		11,14						
2137	3,7	19,14	32,04	42,88	13,74	18,3	0,75	1,678
2138	3,3	11,72	30,25	42,04	12,72	17,53	0,725	1,61
2139	5,2	19,28	32,06	42,07	13,49	18,57	0,726	1,716
2140	4,4	12,28	31,51	41,54	13,09	18,42	0,71	1,723
2141	3,6	11,86	39,83	46,29	18,44	21,39	0,862	1,973
2142	4,3	19,86	39,92	45,74	18,26	21,66	0,843	2,031
2143		12,28						
2144		20,43						

2145	4	11,86	30,88	40,77	12,59	18,29		0,688	1,687
2146		39,72							
2147		40							
2148		26,72							
2149		23,72							
2150		31							
2151		37							
2152		39,14							
2153		39,72							
2154		38,57							
2155		39							
2156		39,28							
2157		38							
2158		27,14							
2159		33,28							
2160		33,28							
2161		32,72							
2162		40							
2163		36,86							
2164		37,28							
2165		38							
2166		39,43							
2167		38,28							
2168		37							
2169		40,14							
2170		38							
2171		39,14							
2172		39,72							
2173		37,57							
2174		39,43							
2175		39,28							
2176		36,43							
2177		38,43							

2178		36						
2179		38,72						
2180		33						
2181	3,5	20,57	32,3	42,1	13,6	18,7	0,727	1,697
2182	3,3	11,57	32,41	41,99	13,61	18,8	0,723	1,669
2183	3,7	19,86	28,75	41	11,79	16,96	0,695	1,578
2184	3,2	11,86	27,93	40,42	11,29	16,64	0,678	1,563
2185	5	20,72	33,69	43,84	14,77	18,92	0,78	1,734
2186	4,5	12,72	32,05	42,12	13,5	18,55	0,727	1,678
2187	4,1	12,28	36,87	42,09	15,52	21,35	0,726	1,996
2188	5,5	20,14	37,19	42,51	15,81	21,38	0,739	2,008
2189	4,8	13,28	39,05	43,89	17,14	21,91	0,782	2,133
2190	5,9	19,86	40,53	44,85	18,18	22,35	0,813	2,188
2191	4,1	20,57	37,59	44,42	16,7	20,89	0,799	1,968
2192	4,2	12,57	37,01	44,17	16,35	20,66	0,791	1,937
2193	3,5	38,14						
2194	3,5	27,72						
2195	5,5	33,14						
2196	4,7	26,86	44,14	44,92	19,82	24,31	0,815	2,268
2197	4,6	34,86						
2198	5,7	24,14						
2199	3,8	32,14	35,81	44,01	15,76	20,05	0,786	1,818
2200	4,1	24,14	35,71	44,6	15,93	19,78	0,805	1,768
2201	3,5	38,43	38,88	48,32	18,79	20,09	0,935	1,832
2202	4	14,28	29,95	41,43	12,41	17,54	0,71	
2203	3,8	21	30,24	42,03	12,71	17,53	0,725	1,741
2204	3,5	35,57						
2205	3,6	26,72	36,65	44,69	16,38	20,27	0,808	1,878
2206	4,9	35,86	33,48	44,11	14,77	18,71	0,789	1,85
2207	3,2	15,28						
2208	3,7	35,43	39,74	43,78	17,4	22,34	0,778	2,144
2209	3,6	20,28	39,66	45,18	17,92	21,74	0,824	2,079
2210		12,14						

2211	3,6	11,28	36,41	45,5	16,57	19,84	0,835	1,906
2212	5,7	32,72	30,56	45,25	13,83	16,73	0,826	1,667
2213	5	20,72	30,36	44,2	13,42	16,94	0,792	1,67
2214	4,4	12,72	29,08	44,01	12,8	16,28	0,786	1,602
2215	6,3	30,57						
2216	4	12,14						
2217	4,5	28,14	36,36	45,18	16,43	19,93	0,824	2,029
2218	4,1	12,28	35,3	44,7	15,78	19,52	0,808	1,973
2219	4,1	19,43	36,19	45,53	16,48	19,71	0,836	1,996
2220	4,3	21,43	34,13	42,57	14,53	19,6	0,741	1,766
2221	3,5	9,86	34	42,5	14,45	19,55	0,739	1,719
2222		12,28						
2223	3,8	30,43	34,22	44,33	15,17	19,05	0,796	1,64
2224	3,8	21,14	31,13	43,27	13,47	17,66	0,762	1,611
2225	3,1	12,14	29,41	43,01	12,65	16,76	0,754	1,553
2226	4,7	33,72						
2227	4,2	20,57						
2228	3,7	13,72						
2229	3,2	19,72	30,34	43,21	13,11	17,23	0,76	1,657
2230	2,6	10,72	30,91	42,54	13,15	17,76	0,74	1,683
2231	4,5	38,28	47,01	49,47	23,26	23,75	0,979	2,245
2232	4,3	10,72	38,81	44,8	17,39	21,42	0,811	2,031
2233		12,57						
2234	4	21,43	34,25	43,29	14,83	19,42	0,763	1,852
2235	4	13,86	33,57	43,19	14,5	19,07	0,76	1,807
2236		19,86	41,81	44,55	18,63	23,18	0,803	2,213
2237	4,2	12,43	41,74	44,75	18,68	23,06	0,81	2,152
2238	4,5	31	38,41	43,21	16,6	21,81	0,761	2,05
2239	4,7	20	37,26	43,34	16,15	21,11	0,765	1,979
2240	4,4	13	37,22	43,33	16,13	21,09	0,764	1,973
2241	3,9	9,57	39,79	46,44	18,48	21,31	0,867	2,069
2242	4,2	20	40,12	46,23	18,55	21,57	0,859	2,08
2243		12,43						

2244	4,9	12,86	35,95	42,89	15,42	20,53		0,751	1,992
2245	5,3	19,57	37,28	43,19	16,37	20,91		0,782	2,044
2246	4,2	12	38,84	45,9	17,83	21,01		0,848	2,065
2247	4,3	30,72	42,4	46,1	19,55	21,85		0,855	2,147
2248	4,1	19	38,41	45,4	17,44	20,97		0,831	2,097
2249		10,86							
2250		12,72							
2251	4,5	24,14	30,66	41,84	12,83	17,83		0,719	1,699
2252		12,43							
2253		13,57							
2254	4,8	31,57							
2255	4,2	19,57							
2256	3,6	14							
2257		13,28							
2258		37,57							
2259		34,86							
2260		35							
2261	4,4	12,43	36,69	43,63	16,01	20,68		0,774	1,88
2262	4,9	20,43	37,48	43,59	16,34	21,14		0,772	2,015
2263	3,2	13,72	26,22	38,86	10,19	16,03		0,635	1,462
2264	3,6	20,86	27,72	39,68	11	16,72		0,657	1,674
2265	4,4	34,86	32,5	42,52	13,82	18,68		0,739	1,861
2266	4,6	35,72	33,68	42,93	14,46	19,22		0,752	1,816
2267	5,1	31							
2268		12,86							
2269	4	30,28							
2270	3,4	35,28							
2271	4,7	30	36,65	41,39	15,17	21,48		0,706	2,174
2272	4,6	26,14	32,35	43,8	14,17	18,18		0,779	1,618
2273	4,8	34,57	41,02	44,58	18,29	22,73		0,804	2,195
2274	4,5	35,43	28,64	41,79	11,97	16,67		0,718	1,57
2275	4,5	33							
2276	4,8	24							

2277	4,6	12,28	33,8	42,84	14,48	19,32		0,749	1,849
2278	4,4	20,14	35,17	43,84	15,42	19,75		0,78	1,891
2279	3,9	12	31,53	43,38	13,68	17,85		0,766	1,578
2280	5,1	22	30,96	42,63	13,2	17,76		0,743	1,599
2281		11,72							
2282		12,86							
2283	3,8	12,86	28,32	41,41	11,73	16,59		0,707	1,513
2284	3,8	20,86	29,15	42,09	12,27	16,88		0,726	1,576
2285	4,4	20,57	33,22	42,8	14,22	19		0,748	1,812
2286	4	12,57	32,66	42,62	13,92	18,74		0,742	1,746
2287	4	12,72	29,62	40,71	12,06	17,56		0,686	1,64
2288	4,8	19,86	34,72	42,74	14,84	19,88		0,746	1,695
2289		12							
2290	4,4	20	28,05	42,78	12	16,05		0,747	1,591
2291	4,7	12,57	26,53	41,31	10,96	15,57		0,703	1,537
2292	4,2	20,28	29,92	42,04	12,58	17,34		0,725	1,563
2293	3,7	11,72	29,77	42,15	12,55	17,22		0,728	1,534
2294	5	13	33,84	45,77	15,49	18,35		0,844	1,847
2295	5,1	20	33,64	45,6	15,34	18,3		0,838	1,858
2296	4,6	20,86	32,51	43,92	14,28	18,23		0,783	1,738
2297	3,8	12,86	31,45	43,68	13,74	17,71		0,775	1,686
2298	4,5	12	31,62	43,8	13,85	17,77		0,779	1,639
2299	4,4	21	33,12	44,74	14,82	18,3		0,809	1,667
2300	3,3	12	30,77	43,09	13,26	17,51		0,757	1,67
2301	4,1	20	32,42	44,44	14,41	18,01		0,8	1,683
2302	4,5	11,14	33,93	41,14	13,96	19,97		0,699	1,869
2303	4,7	20,72	36,82	44,29	16,31	20,51		0,795	1,906
2304	4,3	12,57	36,67	45,4	16,65	20,02		0,831	1,891
2305	5,1	20	36,89	45,37	16,74	20,15		0,83	1,926
2306	3,3	11,86	30,94	43,6	13,49	17,45		0,773	1,519
2307	3,6	19,86	31,22	43,81	13,68	17,54		0,779	1,519
2308		13,14							
2309	2,9	13,57	30	41,13	12,34	17,66		0,698	1,585

2310	4,4	20,57	30,96	41,63	12,89	18,07	0,713	1,62
2311	2,8	19	28,84	41,78	12,05	16,79	0,717	1,553
2312	2,5	12,86	28,55	41,89	11,96	16,59	0,72	1,51
2313	4,1	20,86	33,43	42,74	14,29	19,14	0,746	1,724
2314	3,5	11,28	33,9	42,53	14,42	19,48	0,74	1,711
2315	4,1	37,43	37,86	44,21	16,74	21,12	0,792	2,045
2316	4,3	20	37,32	44,39	16,57	20,75	0,798	1,961
2317	3,5	12,14	36,13	43,39	15,68	20,45	0,766	1,951
2318		11,86						
2319	4,1	19,86	35,28	43,73	15,43	19,85	0,777	1,909
2320	4	11,43	35,78	44,35	15,87	19,91	0,797	1,885
2321	3,7	12	32,74	43,15	14,13	18,61	0,759	1,626
2322	3,8	13,43	31,16	42,84	13,35	17,81	0,749	1,688
2323	4,1	21,43	32,99	44,28	14,61	18,38	0,794	1,728
2324	4,9	19,57	36,57	44,92	16,43	20,14	0,815	1,765
2325	4,3	33,14						
2326	3,1	40	42,34	50,28	21,29	21,05	1,011	2,041
2327	4,7	30,57	34,27	44,2	15,15	19,12	0,792	1,866
2328	3,5	34	37,26	44,49	16,58	20,68	0,801	1,944
2329	4,6	33,86	29,36	42,77	12,56	16,8	0,747	1,58
2330	4,3	37,43						
2331	3	34,86						
2332	4,4	12,28	35,19	44,1	15,52	19,67	0,789	1,876
2333	5	20,28	36,37	44,84	16,31	20,06	0,813	1,895
2334	3	32,57	27,42	43,69	11,98	15,44	0,775	1,501
2335	5,5	20	30,83	43,49	13,41	17,42	0,769	1,643
2336	5	12,43	31,23	43,41	13,56	17,67	0,767	1,656
2337	3,5	11						
2338	4	12,72	27,89	43,02	12	15,89	0,755	1,486
2339	4,3	22,57	29,36	44,38	13,03	16,33	0,797	1,541
2340	4,8	11,72	27,45	39,34	10,8	16,65	0,648	1,641
2341	5,2	20,72	28,2	40,81	11,51	16,69	0,689	1,634
2342		11,72						

2343		12,14							
2344	3,8	12,72	34,69	45,02	15,62	19,07		0,819	1,805
2345	4	20,28	36,05	43,63	15,73	20,32		0,774	1,786
2346	3,6	19,14	34,8	44,22	15,39	19,41		0,792	1,855
2347	4	11,86	33,72	43,65	14,72	19		0,774	1,735
2348	4	12,28	35,85	43,62	15,64	20,21		0,773	1,76
2349	4,2	12	32,01	42,54	13,62	18,39		0,74	1,683
2350	4,4	19,72	34,45	44,35	15,28	19,17		0,797	1,78
2351	4,6	20	38,49	45,44	17,49	21		0,832	1,985
2352		28,43	34,71	44,1	15,31	19,4		0,789	1,829
2353	4,6	20,14	33,93	42,44	14,4	19,53		0,737	1,807
2354	3,9	12,72	32,89	41,98	13,81	19,08		0,723	1,761
2355		11,28							
2356	3,9	11,86	33,43	42,08	14,07	19,36		0,726	1,816
2357	4,3	34,57	34,27	42,39	14,53	19,74		0,736	1,816
2358	4,1	19,86	34,38	43,13	14,83	19,55		0,758	1,791
2359	3,9	12	32,35	44,35	14,35	18		0,797	1,838
2360	3,1	19,72	28,94	43,91	12,71	16,23		0,783	1,501
2361	3,2	12,57	27,97	43,4	12,14	15,83		0,766	1,471
2362	3,4	12,86							
2363	3,3	19,86	30,18	43,17	13,03	17,15		0,759	1,591
2364	3,2	12,43	23,16	40,71	9,43	13,73		0,686	1,29
2365	4,2	20	23,38	41,06	9,6	13,78		0,696	1,329
2366	4,4	31,86	35,81	43,67	15,64	20,17		0,775	1,763
2367	4,4	22,86	35,24	43,5	15,33	19,91		0,769	1,724
2368	4,2	12,14	34,73	43,43	15,08	19,64		0,767	1,707
2369	3,4	12,57	32,17	44,48	14,31	17,86		0,801	1,563
2370		30,43							
2371	4,6	11,86	37,55	46,55	17,48	20,07		0,87	1,867
2372	4,9	11,28	37,57	43,91	16,5	21,07		0,783	1,902
2373	4,7	19,86	38,88	45,78	17,8	21,08		0,844	1,908
2374	4,3	20,86	38,08	47	17,9	20,18		0,887	1,949
2375	4,7	20,43	33,2	42,28	14,04	19,16		0,732	1,818

2376	4,2	12,43	32,84	42,38	13,92	18,92	0,735	1,812
2377	4,5	36,57	33,89	45,05	15,27	18,62	0,82	1,702
2378	4,3	33,86	40,11	43,72	17,54	22,57	0,777	2,026
2379	3,7	24,57	35,11	42,55	14,94	20,17	0,74	1,878
2380	2,8	34,28						
2381	4,2	30,72						
2382	4,1	31,14	34,47	44,47	15,33	19,14	0,8	1,725
2383	5,1	35						
2384	4,5	37,14						
2385	3,5	30						
2386	5,1	28,43	28,32	44,06	12,48	15,84	0,787	1,471
2387	3,2	30,86						
2388	3,4	38,72						
2389	3,6	30,28	36,03	45,12	16,26	19,77	0,822	1,861
2390	4	38,86						
2391	3,7	30,72						
2392	4,8	32,14	34,29	44,32	15,2	19,09	0,796	1,72
2393	5,1	33,72						
2394	4,5	25						
2395	4,4	22,43	27,47	43,72	12,01	15,46	0,776	1,442
2396	3,9	32,57	31,23	42,58	13,3	17,93	0,741	1,602
2397	4,8	32,28	36,11	45,41	16,4	19,71	0,832	1,849
2398	5,1	20,43	42,58	44,78	19,07	23,51	0,811	2,376
2399	4,6	35,86						
2400	4,3	24,14	42,73	46,54	19,89	22,84	0,87	2,026
2401	4,1	19,72	28,88	41,62	12,02	16,86	0,712	1,577
2402	3,5	11,86	28,5	41,33	11,78	16,72	0,704	1,527
2403	3,1	37,43						
2404	5,3	20,14	30,71	44,44	13,65	17,06	0,8	1,601
2405	5,7	12,57	31,75	45,29	14,38	17,37	0,827	1,614
2406		11,72						
2407	4,8	20,43	35	45,6	15,96	19,04	0,838	1,844
2408	4,8	12,43	34,42	44,94	15,47	18,95	0,816	1,77

2409	4,1	12,28	31,16	44,22	13,78	17,38		0,792	1,617
2410	4,3	20	30,82	43,34	13,36	17,46		0,765	1,617
2411	4,8	34,72	36,35	42,58	15,48	20,87		0,741	1,981
2412	5	20,72	37,02	42,78	15,84	21,18		0,747	1,985
2413	4,2	12,14	34,54	41,14	14,21	20,33		0,698	1,973
2414	4	11,86	30,53	43,39	13,25	17,28		0,766	1,494
2415	3,7	20,72	30,51	43,33	13,22	17,29		0,764	1,521
2416		11,43							
2417		10,57							
2418	3,7	11,43	29,07	41,83	12,16	16,91		0,719	1,505
2419		10,57							
2420	4,5	12,28	31,87	41,66	13,28	18,59		0,714	1,702
2421	4,2	21,14	32,66	41,51	13,56	19,1		0,709	1,762
2422	3,7	20,28	37,82	44,34	16,77	21,05		0,796	1,955
2423	3,5	12,28	33,96	42,52	14,44	19,52		0,739	1,841
2424	4,7	20,14							
2425	4,9	11,57	32,29	42,02	13,57	18,72		0,724	1,683
2426		12,57							
2427	3,6	30,72	32,85	43,13	14,17	18,68		0,758	1,765
2428	3,8	18,86	30,72	42,02	12,91	17,81		0,724	1,658
2429	4,3	12,72	33,32	42,58	14,19	19,13		0,742	1,728
2430	3,9	20,43	34,1	43,22	14,74	19,36		0,764	1,741
2431	3,9	11	30,9	42,45	13,12	17,78		0,737	1,644
2432	3,9	13,14	29,22	41,2	12,04	17,18		0,7	1,544
2433	3,7	20,14	30,11	41,87	12,61	17,5		0,72	1,587
2434		12,57							
2435	4,1	20	30,96	41,11	12,73	18,23		0,698	1,712
2436	3,8	12	29,77	40,07	11,93	17,84		0,668	1,658
2437	4,5	20,28	40,07	46,24	18,53	21,54		0,86	2,01
2438	4,6	12,28	37,82	44,55	16,85	20,97		0,803	2,026
2439	3,7	18,57	31,87	42,95	13,69	18,18		0,753	1,677
2440	3,1	10,72	30,88	42,03	12,98	17,9		0,725	1,635
2441	3,4	12,14	27,36	41,08	11,24	16,12		0,697	1,602

2442	3,7	23,43	28,94	41,81	12,1	16,84	0,718	1,667
2443	3,4	19,72	34,96	44,39	15,52	19,44	0,798	1,754
2444	3,7	13,14	33,76	44,07	14,88	18,88	0,788	1,687
2445		11,43						
2446	3,6	21	30,87	44,63	13,78	17,09	0,806	1,578
2447	3	12,72	28,63	43,9	12,57	16,06	0,782	1,495
2448	4,3	12,57	30,32	42,28	12,82	17,5	0,732	1,616
2449	4,8	21,72	30,51	42,31	12,91	17,6	0,733	1,644
2450	5,4	20,72	32,08	43,17	13,85	18,23	0,759	1,772
2451	5,1	13,14	31,11	42,2	13,13	17,98	0,73	1,733
2452	3,7	12,14	29,41	42,5	12,5	16,91	0,739	1,005
2453	5	22,14	33,31	44,01	14,66	18,65	0,786	1,704
2454		33,72						
2455		29,43						
2456		34,28						
2457	3,9	35,86	36,92	41,73	15,41	21,51	0,716	1,949
2458	3,6	11,86						
2459	3,2	30,57	31,29	43,63	13,66	17,63	0,774	1,541
2460	5	32,72	36,35	46,21	16,8	19,55	0,859	2
2461	4,4	19,86	36,01	44,59	16,06	19,95	0,805	1,876
2462	4,3	11,28	35,36	43,94	15,54	19,82	0,784	1,838
